# Solid-Phase Glycolipid Synthesis Expedites Liposome Functionalization

**DOI:** 10.1021/jacs.6c10089

**Published:** 2026-07-06

**Authors:** Marilet Sigler, Calvin Hon, Kai G. Pohl, Leif E. Sander, Manuel G. Ricardo, Peter H. Seeberger

**Affiliations:** † Department of Biomolecular Systems, Max-Planck-Institute of Colloids and Interfaces, Am Muehlenberg 1, 14476 Potsdam, Germany; ‡ Institute of Chemistry and Biochemistry, Freie Universitaet Berlin, Altensteinstraße 23a, 14195 Berlin, Germany; § Department of Infectious Diseases, Pulmonology and Intensive Care Medicine, Charité-University Berlin, Augustenburger Platz 1, 13353 Berlin, Germany

## Abstract

Glycolipids play important roles in immune modulation, which makes them attractive components for vaccine delivery systems. However, access to these molecules remains a major limitation as most synthetic approaches rely on arduous solution-phase strategies. Here, we report a unified solid-phase platform that combines automated glycan assembly and iterative amide couplings for the rapid on-resin generation of a variety of mannan- and Gb3-derived glycolipids. This modular method enables the systematic diversification of glycan and lipid-anchor domains within a single synthetic framework. The synthetic constructs were used to generate glyco-functionalized liposomal nanoparticles with potential for immune cell targeting. Preliminary biological studies demonstrate efficient cell-associated uptake by antigen-presenting cells and show that biological performance is governed not only by glycan identity but also by anchor architecture.

## Graphical abstract

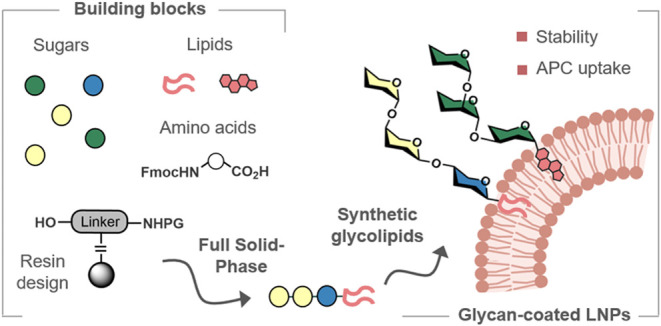

## Introduction

Liposomes have been widely explored as biodegradable and biocompatible nanocarriers for medical applications. Their highly tunable physicochemical properties allow for precise control over particle size, surface charge, and membrane composition, making vesicle customization a key strategy in vaccine development.^1–3^ Within the liposome architecture, the internal aqueous compartment is well suited for encapsulation of hydrophilic molecules (e.g., CpG),^4^ whereas the lipid bilayer can accommodate amphiphilic immunostimulatory molecules such as lipopeptides (e.g., Pam2Cys and Pam3Cys)^5^ and glycolipids (e.g., MPLA,^6,7^ and α-GalCer^8^). By incorporating these molecules into the lipid bilayer, functional moieties can be displayed on the nanoparticle surface to serve as ligands for specific protein receptors. This surface exposure is a critical determinant of cellular uptake and the subsequent activation of innate and adaptive immune pathways.^9^

Glycans play a crucial role in molecular recognition processes and are key mediators of immune cell signaling through interactions with lectins and other immune receptors.^10^ These interactions are intrinsically multivalent, as clustered glycan displays enhanced binding relative to monovalent ligands.^11,12^ Mimicking this mode of presentation using nanomaterials has become a suitable approach to modulate immune recognition.^13^ In this context, glycan-decorated lipid nanoparticles (GLNPs) have emerged as an attractive strategy for receptor-targeted delivery^14–17^ and immunomodulation.^18–24^ In such systems, exposed carbohydrate ligands engage specific immune receptors to modulate cellular uptake and downstream immune response.^25,26^ GLNPs have also addressed critical limitations of mRNA-based lipid nanoparticles (LNPs), including insufficient accumulation in antigen-presenting cells (APCs) and reduced efficacy upon repeated administration. Mannans and galactans that serve as high-affinity ligands for DC-SIGN have been incorporated onto the liposomal surface to facilitate receptor-mediated endocytosis and enhance mRNA delivery specifically into APCs.^15,27,28^ Replacement of PEG-lipids with maltose-derived glycolipids improved physicochemical properties and enhanced splenic accumulation.^29^ Similarly, sialoglycolipid-coated LNPs demonstrated superior mRNA transfection efficiency in spleen-targeted delivery compared to conventional PEG-LNPs.^30^ Rhamnose-coated particles further improved APC uptake through interactions with natural anti-Rha antibodies.^31^

Current strategies for liposomal glyco-functionalization generally fall into two categories: the coformulation of presynthesized glycolipids during particle assembly (Figure 1)^32,33^ or the post-functionalization of preformed liposomes via chemoselective ligation.^34–36^ Direct insertion during formulation is particularly advantageous due to its simplicity; the amphiphilic nature of glycolipids allows them to spontaneously integrate into the lipid bilayer without disrupting the nanoparticle architecture.

**Figure 1:**
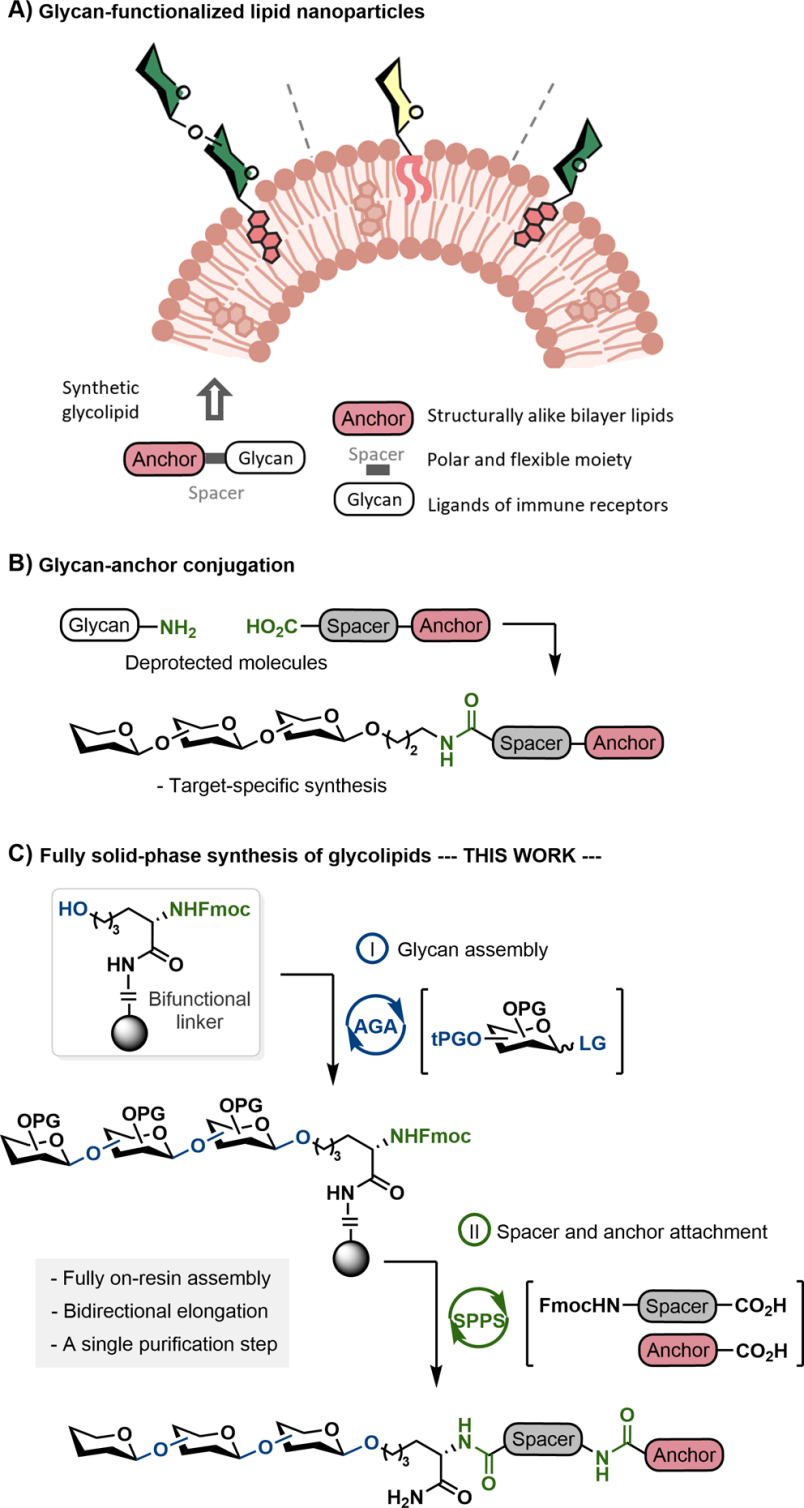
(A) Glycan-functionalized LNPs through lipid tail insertion. Chemical methods to prepare glycolipids: (B) Conventional target-specific approach based on late-stage chemoselective coupling of prefunctionalized glycan and anchor motifs. (C) Unified fully solid-phase strategy enables access to diverse glycolipids. A resin-bound linker is the basis for bidirectional assembly, combining Automated Glycan Assembly (AGA) for glycan elongation with Solid-Phase peptide Synthesis (SPPS) to introduce a spacer and construct the lipid anchor.

However, the structural complexity and inherent diversity of glycolipids present significant synthetic challenges. Isolation from natural sources is often hindered by heterogeneity and impurities, making chemical synthesis indispensable. Currently, most synthetic routes rely on late-stage coupling between prefunctionalized glycans and lipid anchors (Figure 1B). While effective, these solution-phase methods are typically target-specific, requiring intensive optimization for each conjugate and arduous purification steps. Despite the significant advances in solid-phase methods for glycan synthesis,^37^ unified methods capable of generating structurally diverse glycolipid collections remain limited.^38–40^

Here, we report a versatile solid-phase platform that enables systematic variation of each glycolipid structural component (Figure 1C). Central to this approach is the design of a resin-bound linker capable of withstanding the chemical transformations required to construct both the glycan headgroup and the lipid anchor. Automated glycan assembly (AGA) allows for glycan extension, while the lipid anchor is installed by solid-phase peptide synthesis (SPPS). Compared to previous efforts to integrate these solid-phase methodologies, the present approach enables the assembly of glycolipids entirely on solid support, without requiring prefunctionalized glycan building blocks^41,42^ and avoiding incompatibilities between glycan and peptide chemistry.^43,44^ Overall, this platform expedites access to a structurally diverse glycolipid collection, thereby facilitating the screening of customized glyco-liposomal formulations. Preliminary studies identify the role of glycan and lipid architecture in call-associated uptake across relevant APC subsets and suggest potential application as a vaccine delivery system.

## Results and Discussion

To implement this synthetic concept, we developed a combined AGA-SPPS workflow for the glycolipid assembly. The integration of these two on-resin techniques required the design and synthesis of a suitable resin-bound bifunctional linker. In addition to the attachment to the solid support, this linker needed two orthogonal functional groups: one to initiate the glycan elongation and another for the installation of the spacer and anchor.

### Bidirectional Linker for Fully On-Resin Glycolipid Assembly

The nonproteinogenic amino acid residue 6-hydroxynorleucine (Hle) was selected as the core linker since its side chain can be *O*-glycosylated and its primary amino group supports peptide elongation. Danishefsky et al. used Hle as a glycosyl acceptor in solution-phase syntheses of complex glycopeptides.^45,46^ Compared to the proteinogenic residues serine and threonine, Hle offers superior reactivity due to the increased accessibility of its primary hydroxyl group, and the extended alkyl chain avoids β-elimination.^47^ We modified Hle by placing a mildly acid-labile protecting group (PG) at the ε-hydroxyl position that upon removal enables acid-catalyzed glycosylation while preserving the Fmoc-N^α^-amino group for subsequent incorporation of the hydrophobic moiety by SPPS.

The selection of the solid support represented a key aspect of the methodology, given the distinct chemical requirements of AGA and SPPS. Wang and Methylbenzhydrylamine (MBHA) Rink amide resins both typically require a high concentration of trifluoroacetic acid (TFA> 50%) for final cleavage.^48^ Therefore, their lability under glycosylation conditions was assessed. Protected-Hle was attached to each support, and variation of the resin loading was monitored before and after acid cycles using standard Fmoc-based colorimetric procedure (see the Supporting Information).^49^ As anticipated, the standard AGA acid wash (1% trimethylsilyl triflate acid, TMSOTf, in CH_2_Cl_2_, *v/v*) caused a considerable decrease in loading of both resins. Wang resin was particularly affected, consistent with the higher lability of its ester linkage under acidic conditions, rendering it unsuitable for this application. MBHA Rink amide exhibited good stability throughout iterative acid treatments. Under optimized conditions, using 2% trichloroacetic acid (TCA) in CH_2_Cl_2_ for AGA acid wash and standard 0.15% triflic acid (TfOH) in CH_2_Cl_2_ for glycosylation, the MBHA Rink amide resin showed negligible cleavage (less than 0.1%), confirming the required resistance for a combined AGA-SPPS approach.

The bifunctional nature of the chosen linker enables bidirectional elongation of the glycolipid regardless of the order in which the building units are incorporated. However, to minimize possible interference of the peptide-derived functionalities during glycosylation, we selected a sequential workflow where the glycan portion was first constructed by AGA, followed by the installation of the spacer and lipid anchor by SPPS.

### Assembly of Mannose-Based Glycolipids

Mannose-containing glycolipids served as the initial, representative targets, motivated by the high expression of mannose receptors on antigen-presenting cells and the immunological relevance of oligomannosides. In particular, α-(1→2)-trimannose and α-(1→2)-α-(1→3)-trimannose motifs have been associated with immunostimulatory properties.^50^ Two mannan oligosaccharides were assembled on MBHA Rink amide resin equipped with Hle bifunctional linker **1** on a 0.015 mmol scale using a home-built synthesizer.^51^ Glycan elongation relied on iterative cycles of acid wash, glycosylation, and deprotection using 20% Et_3_N in dimethylformamide (DMF) (Figure 2A). Thioglycoside donors were activated *in situ* under controlled temperature conditions (−20 °C for 10 min, then warmed up to 0 °C for 20 min), unless otherwise stated for a specific glycosylation step, using six equivalents of the building block (BB) with an equimolar amount of *N*-Iodosuccinimide (NIS) and catalytic TfOH to ensure complete couplings.

**Figure 2:**
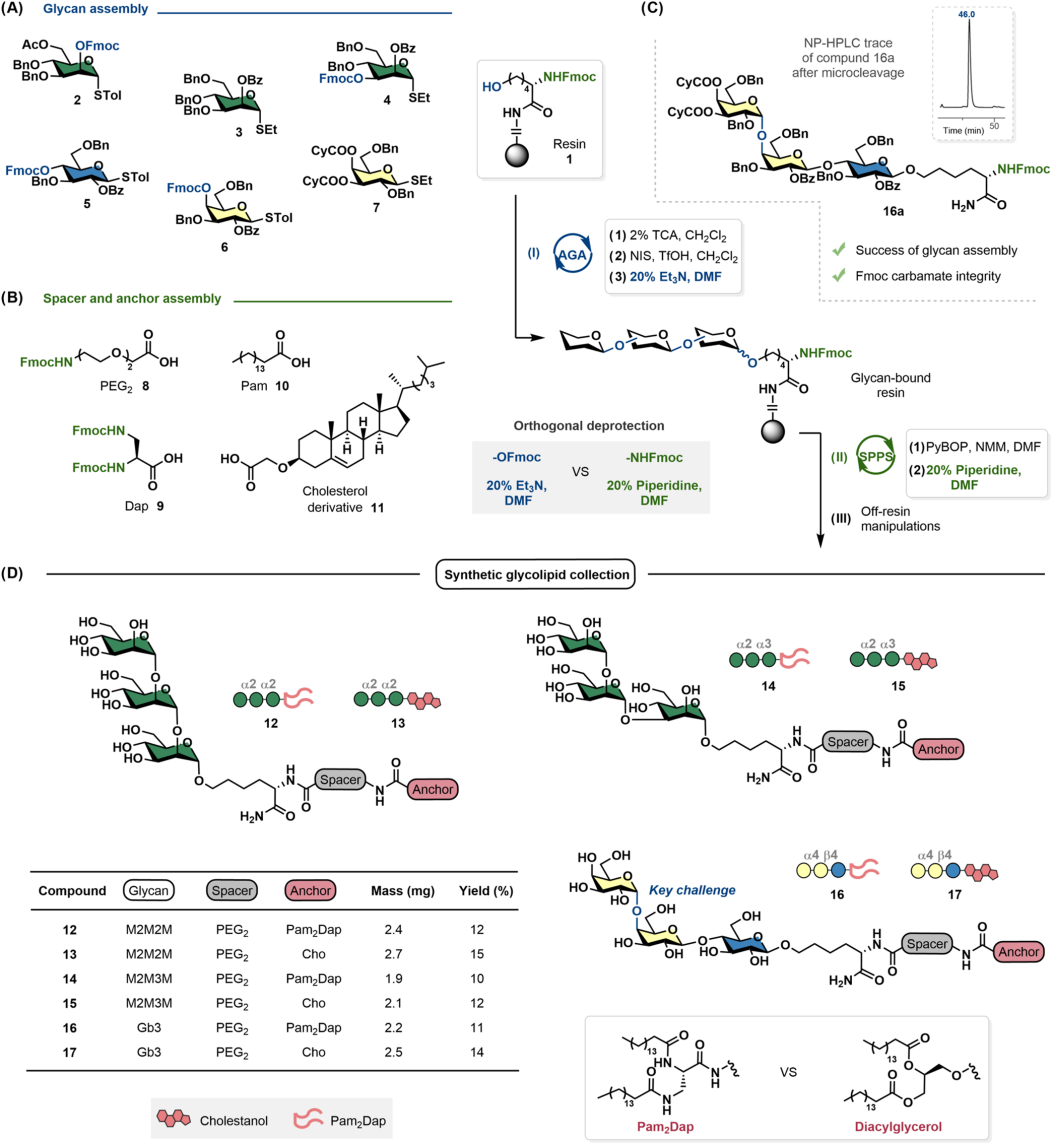
Synthetic glycolipids prepared using the solid-phase methodology. Platform versatility is demonstrated by systematic variation of the glycan headgroup and hydrophobic motif within the same synthetic framework. Stereoselective α-1,4-galactosylation required for Gb3 structures represents a key synthetic challenge addressed here. The Pam2 Dap anchor was incorporated as a diacylglycerol mimetic. Building blocks used for (A) glycan assembly and (B) incorporation of lipid motif. (C) Example of successful glycan assembly and integrity of Fmoc carbamate after AGA. (D) Synthetic glycolipid collection obtained by the developed solid-phase method.

The linear α-(1→2)-trimannose motif was assembled by sequential incorporation of mannosyl donor **2** two times, followed by capped donor **3** (Figure 2). The α-(1→2)-α-(1→3)-trimannose analogue was prepared similarly, employing donor **4**, bearing an Fmoc carbonate at C3-OH to enable the alternative extension. Successful glycan assembly was confirmed for each target by NP-HPLC and MALDI-TOF analysis following microcleavage from sampled resin beads (see the Supporting Information). This analysis was particularly important to verify that, after glycan elongation, the linker α-amino group remained intact and, upon deprotection with 20% piperidine in DMF, enabled smooth transition to SPPS. Orthogonality of glycan temporary protecting groups and the amino linker was essential for the sequential AGA-SPPS.

Spacer and lipid-anchor motifs were then introduced using standard peptide coupling cycles (benzotriazol-1-yloxy)­tripyrrolidinophosphonium hexafluorophosphate, PyBOP/*N*-methyl morpholine, NMM) and Fmoc carbamate deprotection (Figure 2B). A short poly­(ethylene glycol) PEG-based amino acid spacer was introduced to spatially separate the glycan and peptide motifs as previously demonstrated.^41,52,53^ We next explored distinct hydrophobic motifs relevant to liposomal formulation. A diacylglycerol-mimetic anchor was generated using diaminopimelic acid (Dap), where α- and side-chain amino groups enabled double palmitoylation (palmitic acid, Pam) after deprotection, affording the Pam_2_Dap motif. This architecture resembles phospholipid-derived diacyl lipids while avoiding a charged phosphate headgroup (Figure 2D). In parallel, a cholestanol-derived anchor was prepared from a carboxyl-functionalized cholesterol BB (see the Supporting Information), allowing for direct incorporation through the same SPPS protocol. Using this approach, fully protected mannose glycolipids bearing varied anchors were obtained smoothly, with no significant difference observed during solid-phase assembly.

### Assembly of Gb3-Containing Glycolipids

We next extended the scope of the platform through the synthesis of more chemically demanding globotriosyl (Gb3)-containing glycolipids. Gb3 ceramide stimulates immune responses in influenza models, rendering it an attractive target.^54^ The Gb3-trisaccharide headgroup consist of α-d-Gal-(1→4)-β-d-Gal-(1→4)-β-d-Glc. The formation of 1,2-*trans*-glycosidic linkages was efficiently achieved by neighboring participation of benzoyl (Bz) ester group at C2-OH of glucoside **5** and galactoside **6**. The installation of the terminal α-galactosidic linkage represented the major synthetic challenge because of the absence of anchimeric assistance. Since stereoselectivity is particularly critical in AGA to avoid anomeric mixtures that compromise the yield and complicate purification, building block **7** was specifically designed to address this transformation (see the Supporting Information). Among a series of esters with different degrees of steric demand placed at C3-OH and C4-OH, the cyclohexanecarbonyl (CyCO) group provided the highest selectivity for 1,2-*cis* linkage formation. This observation is consistent with literature reports describing remote participation in galactosylations.^55–58^ The steric bulk of the CyCO group might contribute by shielding the top side of the sugar ring during nucleophilic attack, promoting the α-isomer formation.^59^ Despite the increased synthetic complexity of the Gb3 sequence, AGA proceeded efficiently on the new solid support. Microcleavage and analysis of sampled resin beads after assembly confirmed successful glycan construction and preservation of the Fmoc carbamate handle (Figure 2C). Subsequent installation of Pam_2_Dap and cholestanol anchors using the optimized SPPS conditions afforded Gb3 glycolipids **16** and **17**, demonstrating that the platform tolerates more demanding oligosaccharide architectures without modification of the downstream synthetic sequence.

### Off-Resin Modifications

Following complete on-resin assembly, ester PGs, including the stereodirecting CyCO group, could be removed under the standard on-resin methanolysis conditions. Cleavage from the resin was then achieved under acidic conditions (up to 30% TFA) without detectable degradation of the glycosidic linkages. Final hydrogenolytic removal of benzyl protecting groups was performed directly on the crude material, avoiding intermediate purification steps that could be complicated by amphiphile solubility. It was anticipated that the likely reduction of the steroid double bond during the necessary hydrogenolysis step, resulting in the conversion of cholesterol to cholestanol (Cho), would not compromise its ability to coassemble within the lipid bilayer.

In contrast to the highly uniform solid-phase stage, final deprotection and purification displayed anchor-dependent behavior, primarily attributed to differences in glycolipid solubility. Efficient processing required solvent systems capable of maintaining both partially protected intermediates and fully deprotected amphiphilic products in solution, thereby minimizing precipitation or aggregation during heterogeneous transformations. Establishing a universal purification procedure for the glycolipid collection was not feasible. Successful purification required minimal use of water, addition of cosolvents such as MeOH or *i*PrOH during sample preparation, and HPLC column temperature up to 40–50 °C. Oligosaccharides bearing different anchors, ready for liposomal formulation, were successfully obtained in global yields ranging from 10 to 15% (Compound **12** to **17**) (Figure 2D). The high reproducibility and modularity of the method highlight the robustness of the platform and suggest broader applicability to diverse lipid-peptide-glycan conjugates.

### Liposomal Formulation of Synthetic Glycolipids

Glycan-functionalized liposomal nanoparticles (GLNPs) were prepared by thin-film hydration followed by extrusion (see the Supporting Information).^60^ The coformulation strategy enabled direct incorporation of the synthetic glycolipids together with 1,2-distearoyl-*sn*-glycero-3-phosphorylcholine (DSPC) and cholesterol during film formation, allowing for anchoring the hydrophobic tail into the lipid bilayer while exposing the glycan headgroup at the particle surface.

Formulation conditions were optimized using 5–15% of α-1,2-trimannose glycolipids, replacing either phospholipid or cholesterol components. Glycan coating efficiency (∼90%) was determined by RP-HPLC with ELSD detection. All formulations exhibited narrow hydrodynamic size distributions (< 200 nm) with low polydispersity (PDI < 0.2), consistent with homogeneous nanoparticle populations suitable for cellular uptake studies and sterile filtration (Figure 3).

**Figure 3:**
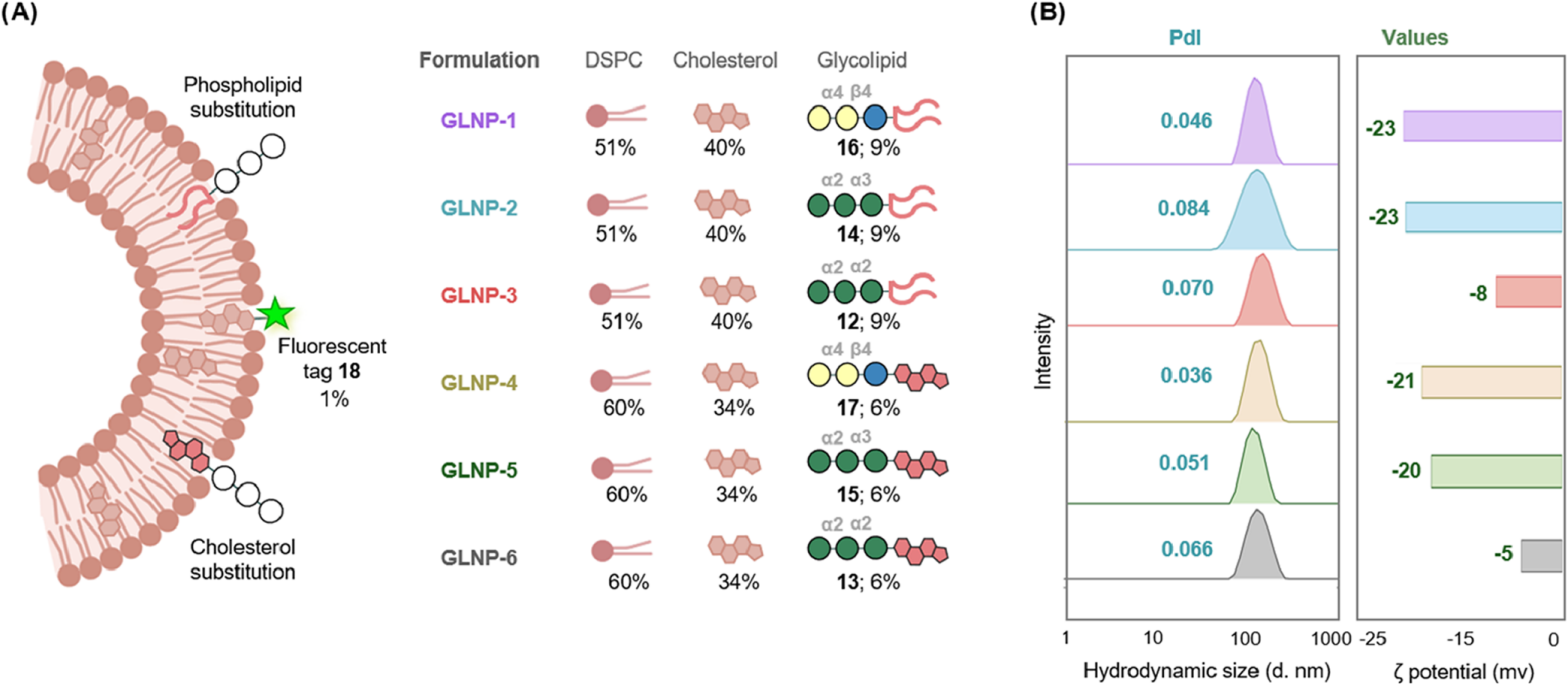
Formulation and physicochemical characterization of fluorescent-labeled glycan-functionalized lipid nanoparticles (GLNPs). (A) Synthetic glycolipids (GLs) were coassembled with conventional liposomal components. (B) Hydrodynamic diameter, size distribution, and ζ potential of GLNP formulations where 15% of either phospholipid or cholesterol content was substituted with a synthetic glycolipid.

Surface exposure of the mannose residues was confirmed by lectin-binding assays that induced time-dependent agglutination of mannosylated LNPs but not for the unmodified control.^34^ This observation was further supported by fluorescence microscopy using fluorescently labeled concanavalin A (ConA-FITC). Transmission electron microscopy (TEM) analysis revealed spherical vesicles of size less than 200 nm, consistent with DLS measurements. Glycolipid incorporation of up to 15% did not significantly affect the particle size or morphology, whereas higher loading (30%) led to structural disruption. Accordingly, a 15% substitution was selected to formulate the remaining glycolipids.

Across the glycolipid collection, all GLNPs showed sizes of 150–170 nm with low dispersity (PDI < 0.2). Functionalization of the particle with Gb3 and α-1,2-α-1,3-trimmanose- containing lipids exhibited higher changes in surface charge (ζ ≤ −20 mV) in comparison with those containing α-1,2-trimmanose lipids (ζ > −10 mV). This observation may be attributed to differences in the structural organization of the glycans used to coat the LNPs. Notably, glycan-coated formulations displayed enhanced colloidal stability, maintaining size and dispersity over 8 weeks at 4 °C, whereas unmodified liposomes showed increased aggregation over time. Incorporation of a fluorescent tag **18** (synthesis details in Supporting Information) for preliminary *in vitro* studies did not alter nanoparticle physicochemical properties.

### Cell-Associated Uptake of Glyco-Functionalized Liposomes

To evaluate the immunological relevance of the GLNPs as targeted delivery vehicles, their cell-associated uptake by APCs was assessed by flow cytometry using fluorescently labeled formulations incubated with human peripheral blood mononuclear cells (PBMCs, see the Supporting Information for experimental details). Quantification based on the fluorescent probe together with cell-surface protein markers defining APC subsets permitted assessment of particle association or uptake by each relevant APC subset (Figure 4).

**Figure 4:**
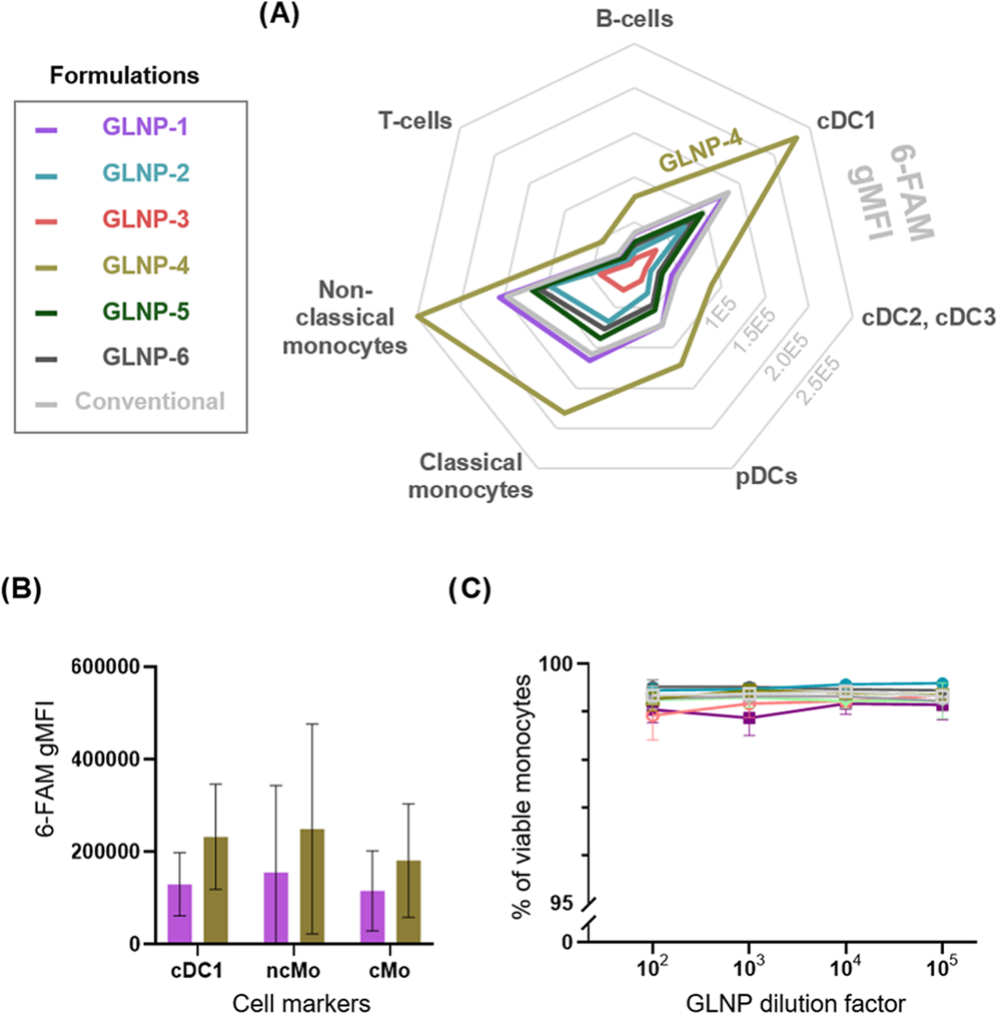
Cell-associated uptake of GLNPs by APCs. (A) Liposomal formulations showed predominant cell-associated signal by dendritic cells, classical and nonclassical monocytes, with GLNP containing 15% of GL **17** exhibiting enhanced APC-associated uptake. (B) GLNP formulated with 15% of GL **17** displayed higher cell-associated fluorescence uptake across relevant APC subsets compared to formulation containing 15% of GL **16**, suggesting stronger impact of cholestanol substitution on particle association/uptake. (C) No significant cytotoxicity toward monocytes was observed for any GLNP formulation following 18 h incubation.

Although the physicochemical properties of the α-1,2-mannosylated liposomes remained largely unchanged across formulations, APC uptake or association was strongly influenced by glycolipid surface density, with enhanced signal observed upon increasing the coating content from 5 to 15% (see the Supporting Information). Across all formulations containing 15% synthetic glycolipid, association/uptake was predominantly observed in dendritic cells (cDC1), classical monocytes (CD16−), and nonclassical monocytes (CD16+) (Figure 4A). Among the glycan motifs investigated, Gb3-functionalized liposomes exhibited the highest signal, with the cholestanol-anchored formulation **GLNP-4** (15% Cho-PEG_2_-Gb3 **17**) showing distinctly superior performance relative to **GLNP-1** containing the Pam_2_Dap-based analogue **16**. Cell-associated uptake was markedly dependent on the nature of the lipid anchor (Figure 4B).

This pronounced anchor-dependent effect suggests that lipid-anchor design plays a central role in controlling glycan presentation at the liposomal surface and, consequently, cellular uptake or association. The more rigid sterol framework of cholestanol may promote stronger bilayer insertion and more favorable glycan exposure compared to the phospholipid-like Pam_2_Dap anchor, thereby enhancing APC recognition.

Importantly, none of the glycan-coated LNPs exhibited detectable cytotoxicity under the conditions tested (Figure 4C), rendering them safe for the intended purpose when stimulated at the stated doses. Overall, these preliminary studies highlight both the intrinsic immunological relevance of the Gb3 motif and the critical importance of anchor design in tuning the biological performance of glycan-decorated liposomal nanocarriers for vaccine applications.

## Conclusions

We developed a unified solid-phase platform that integrates AGA and SPPS for the modular synthesis of structurally diverse glycolipids. Central to this strategy was the use of the nonproteinogenic amino acid Hle as a bifunctional resin-bound linker. The compatibility of the Hle-loaded MBHA Rink amide resin with both iterative glycosylation and peptide coupling cycles enabled the efficient access to relevant glycolipid scaffolds.

The developed methodology permitted systematic diversification of all major structural regions, including the glycan headgroup, spacer, and hydrophobic moiety. Mannose-containing glycolipids and Gb3 derivatives were successfully synthesized. In particular, stereoselective installation of the terminal α-galactosidic linkage in Gb3 was achieved by placing cyclohexanecarbonyl ester at both C3- and C4-OH.

Amide couplings proceeded efficiently to install PEG_2_ residues to distance the sugars from the hydrophobic anchor. The anchor design mimics conventional liposomal components, favoring coassembly of synthetic glycolipids for subsequent decoration of LNPs. The resulting formulations retained favorable physicochemical properties and colloidal stability, confirming the compatibility of the synthetic amphiphiles with the liposomal assembly.

Preliminary *in vitro* evaluations demonstrated efficient uptake (or association) of glycan-functionalized liposomes by antigen-presenting cells, with Cho-PEG_2_-Gb3-decorated nanoparticles exhibiting the best performance. These findings highlight that biological performance is governed not only by glycan identity but also by anchor architecture, underscoring the critical role of lipid anchor design in controlling ligand presentation and, consequently, cellular uptake.

This work establishes a versatile synthetic platform for the rapid generation of glycolipids to tune glyco-functionalized nanocarriers with relevant biological properties. Although only two selected glycolipid classes are presented herein, the modular nature of the platform is expected to be extended to a broader range of glycan architectures. Ongoing immunization studies will further evaluate the potential of Gb3-coated liposomes as targeted vaccine delivery systems. Positive outcomes will potentially lead to the use of Gb3-coated liposomes as a platform to deliver payloads such as antigenic protein-glycan conjugates.

## Supplementary Material



## Notes

Experimental section: all synthetic procedures, liposomal formulation and characterization details, *in vitro* assay procedures, and glycolipid characterization data, including HPLC, HR-MS, and NMR. (PDF)

## Abbreviations

AGA: automated glycan assembly
APC: antigen-presenting cells, BB, building block
Cho: cholestanol
Con-A: concanavalin A
CyCO: cyclohexanecarbonyl
Dap: diaminopimelic acid
DSPC: 1,2-distearoyl-*sn*-glycero-3-phosphorylcholine
FITC: fluorescein isothiocyanate
Gb3: globotriosyl, GLNP, glycan-functionalized lipid nanoparticles
Hle: 6-hydroxynorleucine
SPPS: solid-phase peptide synthesis
TCA: trichloroacetic acid
TfOH: triflic acid
TMSTf: trimethylsilyl triflate
NIS: *N*-iodosuccinimide
Pam: palmitic acid
PG: protecting group
