# Supplementary material for: Solid-Phase Glycolipid Synthesis Expedites Liposome Functionalization
Source: J Am Chem Soc. 2026 Jul 6;148(28):30486–93. doi: 10.1021/jacs.6c10089 (PMC13397566; doi:10.1021/jacs.6c10089)
Supplement: Supplementary file 1 [file ja6c10089_si_001.pdf]

Supporting information

Solid-Phase Glycolipid Synthesis Expedites Liposome Functionalization

Marilet Sigler,[⊥,§] Calvin Hon,[†] Kai G. Pohl,[†] Leif E. Sander,[†] Manuel G. Ricardo,*[,⊥] Peter H. Seeberger*[,⊥,§]

[⊥]Department of Biomolecular Systems, Max-Planck-Institute of Colloids and Interfaces, Am Muehlenberg 1, 14476 Potsdam, Germany

[§]Institute of Chemistry and Biochemistry, Freie Universitaet Berlin, Altensteinstr. 23a, 14195 Berlin, Germany

[†]Department of Infectious Diseases, Pulmonology and Intensive Care Medicine, Charité-University Berlin, Augustenburger Platz 1, 13353 Berlin, Germany

Email:

*peter.seeberger@mpikg.mpg.de

*manuel.garciaricardo@mpikg.mpg.de

Table of content

1. Abbreviations 1
2. General information 1
3. Building blocks synthesis 3
   - 3.1. Synthesis of non-natural amino acid linker 3
   - 3.2. Synthesis of monosaccharide building blocks 12
     - 3.2.1. Stereoselectivity study of designed galactose building blocks 47
   - 3.3. Synthesis of Cholesterol derivative 64
4. Resin Loading of Hle Linker 67
   - 4.1. Stability of resins towards acid conditions 67
5. Solid-phase synthesis of glycolipids 69
   - 5.1. Automated Glycan Assembly 69
     - 5.1.1. Materials and conditions for Automated Glycan Assembly 69
       - 5.1.1.1. Stock solutions 69
       - 5.1.1.2. Modules of Automated Glycan Assembly 69
     - 5.1.2. Post-Automated Glycan Assembly monitoring 71
     - 5.1.3. Glycan assembly of synthetic glycolipids 71
   - 5.2. Solid Phase Peptide Synthesis 75
     - 5.2.1. Materials and conditions for Solid Phase Peptide Synthesis 75
     - 5.2.2. Post-solid phase manipulations 75
       - 5.2.2.1. Analytical RP-HPLC and purification 76
     - 5.2.3. Constructing spacer and glycolipid anchor 77
6. Synthesis of fluorescent tag Cholesterol-K(FAM) 100
7. Materials and methods for liposomal formulation of synthetic glycolipids 103
   - 7.1. Physicochemical characterization of formulations 104
8. Liposomal formulations 105
   - 8.1. Conventional liposomes 105
   - 8.2. α-(1→2)-Man-LNPs 105
     - 8.2.1. ConA binding with mannosylated LNP 106
       - 8.2.1.1. Agglutination study of mannosylated LNP with ConA by DLS 106
       - 8.2.1.2. Qualitative determination of lectin binding with mannosylated LNP 107
   - 8.3. α-(1→2)- Man α-(1→3)-Man-LNPs 109

8.4. Gb3-LNPs 110

9. Materials and conditions for cell-associated uptake of lipid nanoparticles by antigen presenting cell subsets assays.. 112

9.1. Sample handling.. 112

9.2. Monocyte enrichment 112

9.3. Surface marker staining to evaluate liposome-induced activation of monocytes and associated cell toxicity.. 112

9.4. Monitoring kinetics of liposomal cell-associated uptake by APC subsets 112

9.5. Data analysis and statistics.. 113

10. Cell-associated uptake of lipid nanoparticles by antigen presenting cell subsets........ 113

10.1. Innate cell cytotoxicity.. 114

11. References 115

1. Abbreviations

AGA, automated glycan assembly; BB, building block; Bn, benzyl; Bz, benzoyl; Cho, cholestanol; CyCO, cyclohexanecarbonyl; DCM, dichloromethane; Diox, 1,4-dioxane; DIPEA, diisopropylethylamine; DMAP, dimethylaminopyridine; DMF, dimethylformamide; EtOAc, ethyl acetate; EDC, 1-ethyl-3-(3-dimethylaminopropyl)carbodiimide; ESI-MS, electrospray ionization mass spectrometry; Et_3N, triethylamine; FA, formic acid; Fmoc, fluorenylmethyloxycarbonyl; GL, glycolipid; GLNP, glycan-coated lipid nanoparticles; HR-MS, high resolution mass spectrometry; *i*BuCO, isobutyryl; IR, infrared; LNP, lipid nanoparticles; MeCN, acetonitrile; MeOH, methanol; MmtCl, 4-methoxytrityl chloride; PFP, pentafluorophenol; NP-HPLC, normal-phase high-performance liquid chromatography; *n*PrCO, propionyl; Py, pyridine; RP-HPLC, reversed-phase high-performance liquid chromatography; RT, room temperature; SPPS, solid-phase peptide synthesis; *t*Bu, tert-butyl; TCA, trichloroacetic acid; TFA, trifluoroacetic acid; ; MBHA, *p*-methylbenzhydrylamine; MBHA Rink amide, 4-(2′,4′-dimethoxyphenyl-Fmoc-aminomethyl)-phenoxyacetamido-norleucyl-MBHA resin; TfOH, trifluoromethanesulfonic acid; THF, tetrahydrofuran; TIPS, triisopropylsilane; TLC, thin layer chromatography; TMSOTf, trimethylsilyl trifluoromethanesulfonate.

2. General information

All chemicals were reagent grade and used as supplied unless stated otherwise. All solvents for chemical reactions were commercially purchased in p.a. quality. If stated, they were dried in a Solvent Dispensing System (J.C. Meyer). For HPLC and HR-MS spectrometry, solvents with corresponding quality were used. Water was used from a Milli-Q station from Millipore. Automated glycan syntheses were performed on a home-built synthesizer developed at the Max Planck Institute of Colloids and Interfaces.[1] Analytical TLC was performed on Macherey-Nagel silica gel 60 G-25 F254 plates (0.25 mm). Compounds were visualized by UV irradiation or/and dipping the silica plate in a staining solution (sugar stain: 10% H_2SO_4 in EtOH; CAM: 48g/L ammonium molybdate, 60 g/L ceric ammonium molybdate in 6% H_2SO_4 aq sol). Flash column chromatography was carried out by using forced flow of the indicated solvent on Fluka Kieselgel 60 M (0.04 – 0.063 mm). Analysis and purification by normal and reverse phase HPLC were performed by using an Agilent 1200 series. Products were lyophilized using a Christ Alpha 2-4 LD plus freeze dryer. Global yields of solid-phase syntheses were calculated based on the resin

initial loading. Palladium on carbon was removed from reaction mixtures by filtration with Rotilabo syringe filters (Roth), PTFE filters (pore size: 0.45 μm). ^{1}H, ^{13}C and HSQC NMR spectra were recorded on a Varian 400-MR (400 MHz) or Bruker Biospin AVANCE700 (700 MHz) spectrometer. Spectra were recorded in $CDCl_3$ by using the solvent residual peak chemical shift as the internal standard ($CDCl_3$: 7.26 ppm ^{1}H, 77.0 ppm ^{13}C) or co-solvents by locking one of the deuterated solvents used and using the solvent residual peak chemical shift as the internal standard (D_2O in ^{1}H NMR (D_2O: 4.79 ppm ^{1}H); (MeOD: 3.35; 4.78 ppm ^{1}H, 49.3 ppm ^{13}C); (CD_3CN: 1.93 ppm ^{1}H, 1.3; 117.9 ppm ^{13}C). High resolution mass spectra were obtained using a 6210 ESI-TOF mass spectrometer (Agilent) and a MALDI-TOF autoflexTM (Bruker). MALDI and ESI mass spectra were run on IonSpec Ultima instruments. Size and surface charge of lipid nanoparticles were measured using disposable folded capillary tubes (DTS1070; Malvern Panalytical, UK) in a Malvern Instruments Nano-ZS Zetasizer equipped with a 632.8 nm, 4 mW HeNe laser (Malvern Panalytical, UK). Images of lipid nanoparticles were obtained in a JEOL JEM-ARM200F and a Leica SP8 FALCON microscope equipped with a 63× 1.2 NA water immersion objective (Leica, Germany) for fluorescent particles. Flow cytometry was measured on a Beckman Coulter CytoFLEX flow cytometer using CytExpert software. Flow cytometry data was analyzed with FlowJo v10 software. Graphical presentation and statistical analyses were performed using Microsoft Excel, Origin and Graph Prism 10.

3. Building blocks synthesis

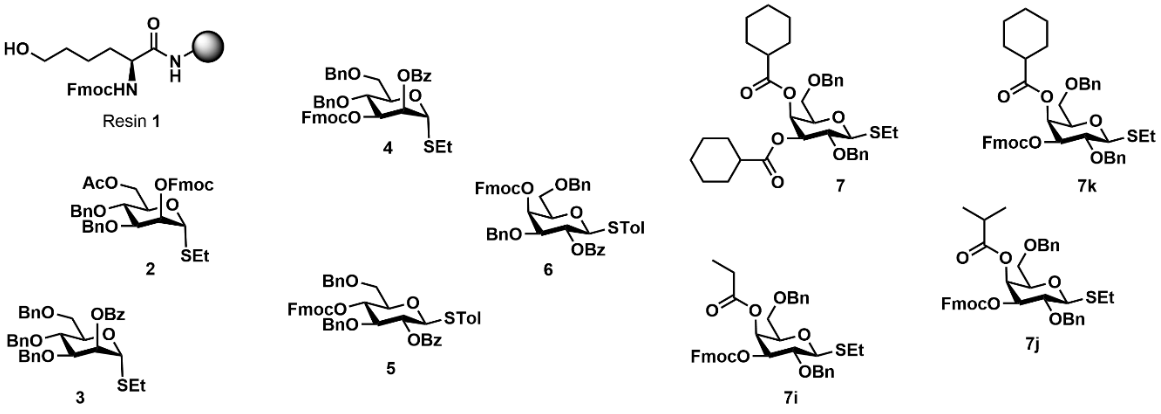

Building blocks (BBs) **2**, **5** and **6** were purchased from GlycoUniverse GmbH & CO KGaA (Germany) while the rest of the BBs were synthesized as described below.

3.1. Synthesis of non-natural amino acid linker

N-α-(9H-fluoren-9-ylmethoxy)carbonyl]-N-ε-(tert-butyloxycarbonyl)- L-lysine allyl ester **(1b)**

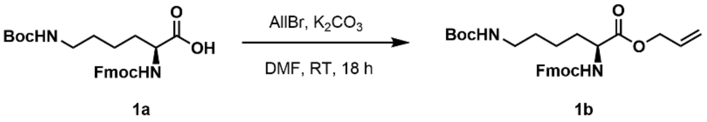

To a solution of **1a** (10.0g, 21.3 mmol) and allyl bromide (4.08 mL, 42.6 mmol, 2.0 equiv) in DMF (30 mL), K_2CO_3 (4.42 g, 31.9 mmol, 1.5 equiv) was added at room temperature and the reaction was stirred for 16 h. After completion, the mixture was diluted with EtOAc (200 mL) and water was added. The two phases were separated, and the organic layer was washed with brine (50 mL), dried over Na_2SO_4, filtered and concentrated. The resulting crude was purified by column chromatography (Hex/EtOAc 2:1, *v/v*) to get the pure compound **1b** (10.1 g, 19.8 mmol, 93%) as a white solid R*f* (Hex/EtOAc 2:1, *v/v*) = 0.35. HR-MS (QTOF) m/z = 531.2493 [M+Na]+, calcd for $C_{29}H_{36}N_2O_6Na$: 531.2471. ^{1}H NMR (400 MHz, $CDCl_3$) δ 7.77 (d, *J* = 7.5 Hz, 2H), 7.63 – 7.56 (m, 2H), 7.40 (t, *J* = 7.5 Hz, 2H), 7.32 (tt, *J* = 7.5, 1.3 Hz, 2H), 5.92 (ddd, *J* = 17.2, 10.6, 5.8 Hz, 1H), 5.41 (d, *J* = 8.3 Hz, 1H), 5.36 (d, *J* = 1.5 Hz, 1H), 5.32 (d, *J* = 1.4 Hz, 1H), 5.27 (dd, *J* = 10.4, 1.3 Hz, 1H), 4.65 (dd, *J* = 5.8, 1.2 Hz, 2H), 4.43 – 4.36 (m, 3H), 4.23 (t, *J* = 7.1 Hz, 1H), 3.11 (t, *J* = 6.9 Hz, 2H), 1.89 (dt, *J* = 13.9, 5.1 Hz, 1H), 1.86 – 1.68 (m, 1H), 1.54 – 1.48 (m, 2H), 1.44 (s, 9H), 1.39 – 1.31 (m, 2H). ^{13}C NMR (101 MHz, $CDCl_3$) δ 171.96, 155.79, 143.55, 141.12, 131.30, 127.52, 126.88, 124.92, 119.79, 118.87, 66.83, 65.82, 53.57, 46.97, 32.00, 29.41, 28.22, 22.16.

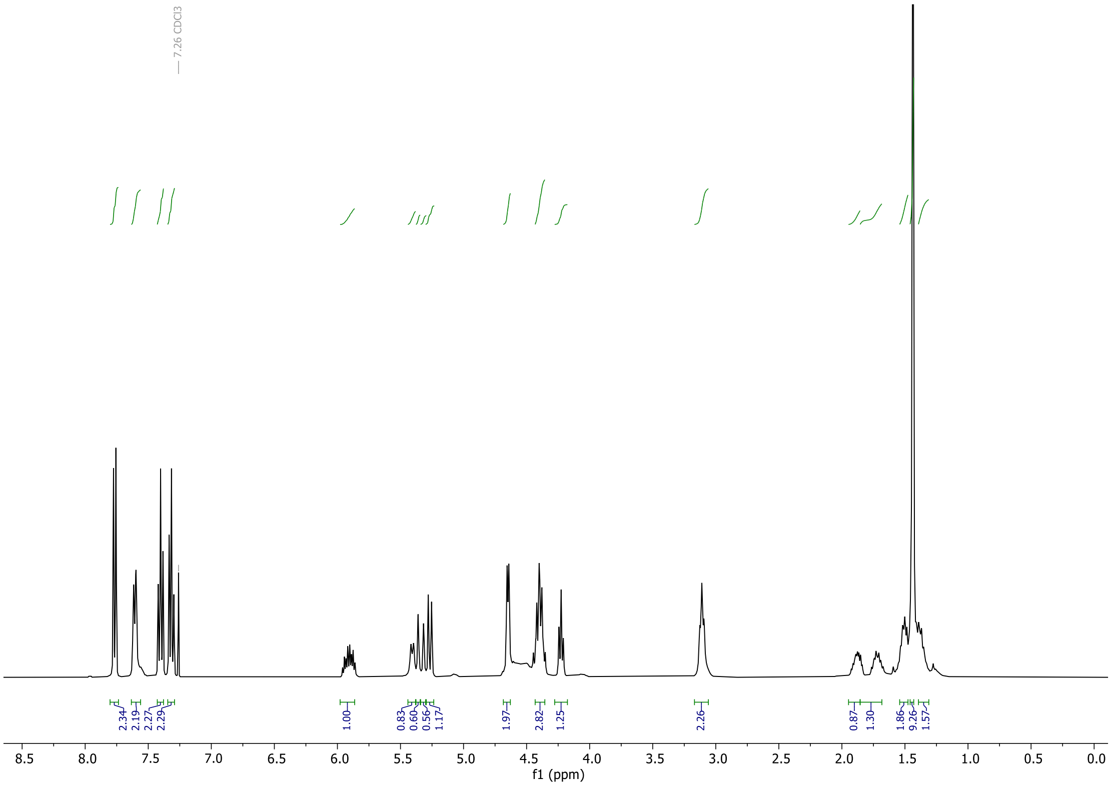

Figure 1. ^{1}H NMR (400 MHz, $CDCl_3$) spectrum of **1b.**

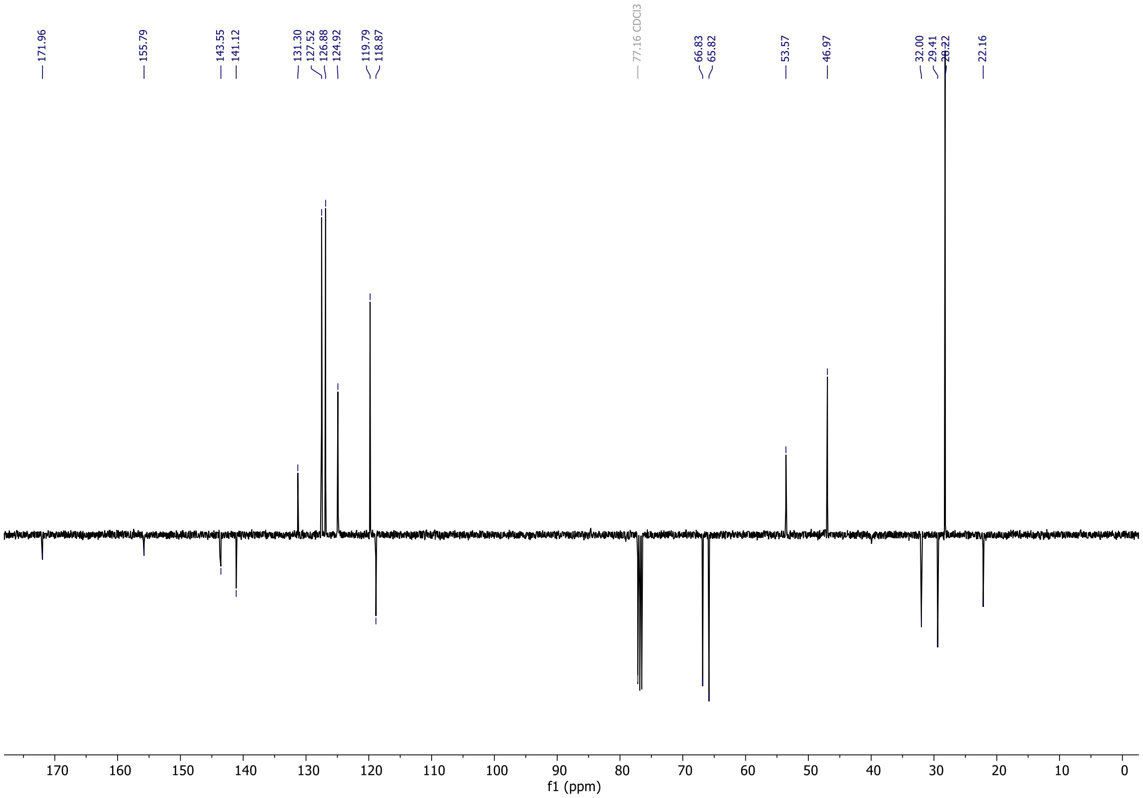

Figure 2. ^{13}C APT NMR (101 MHz, $CDCl_3$) spectrum of **1b.**

N-[(9H-fluoren-9-ylmethoxy)carbonyl]-6-hydroxy-L-norleucine allyl ester **(1c)**

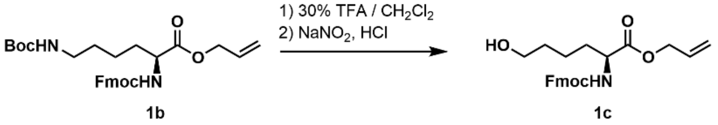

To a solution of **1b** (2.00 g, 3.83 mmol) in CH_2Cl_2 (30 mL), TFA (15 mL) is added. The reaction was stirred at room temperature for 3 h. The reaction was concentrated; CH_2Cl_2 (3×20 mL) and Et_2O (1×20 mL) were sequentially added to the crude and evaporated at reduced pressure to remove TFA. Next, the crude intermediate (3.83 mmol, 1.0 equiv) was dissolved in THF (20 mL) and H_2O (5 mL). Then, conc. HCl (0.350 mL, 11.5 mmol, 3.0 equiv) was added and the mixture was cooled to -5 °C. A solution of $NaNO_2$ (0.79 g, 11.5 mmol, 3.0 equiv) in water (5 mL) was added dropwise. The reaction was stirred at 0 °C for 4 h; monitoring it by TLC ($CH_2Cl_2/CH_3OH/(CH_3)_3N$ 20:1:0.1). After completion, the mixture was diluted with EtOAc (100 mL) and washed with water (50 mL) and brine (50 mL), dried over Na_2SO_4 and evaporated at reduced pressure. The residue was purified by column chromatography (CH_2Cl_2/EtOAc, (gradient from 20:1 to 4:1, v/v) to afford **1c** (0.76 g, 1.85 mmol, 48%) as a pale-yellow solid. R*f* (CH_2Cl_2/EtOAc 4:1, v/v) = 0.4. HR-MS (QTOF) m/z =432.1795 [M+Na]+, calcd for $C_{24}H_{27}NO_5Na$: 432.1787. ^{1}H NMR (400 MHz, $CDCl_3$) δ 7.77 (d, *J* = 7.5 Hz, 2H), 7.60 (dd, *J* = 7.5, 2.6 Hz, 2H), 7.40 (t, *J* = 7.4 Hz, 2H), 7.32 (t, *J* = 7.4 Hz, 2H), 5.91 (ddt, *J* = 16.5, 11.0, 5.8 Hz, 1H), 5.39 (s, 1H), 5.34 (d, *J* = 17.6 Hz, 1H), 5.27 (d, *J* = 10.4 Hz, 1H), 4.65 (d, *J* = 5.8 Hz, 2H), 4.42 (ddd, *J* = 9.7, 7.2, 3.4 Hz, 3H), 4.23 (t, *J* = 7.0 Hz, 1H), 3.64 (t, *J* = 6.4 Hz, 2H), 1.90 (ddt, *J* = 15.1, 10.6, 5.6 Hz, 1H), 1.74 (ddd, *J* = 14.6, 9.6, 6.2 Hz, 1H), 1.59 (dq, *J* = 12.6, 6.2 Hz, 2H), 1.52 – 1.40 (m, 2H). ^{13}C NMR (101 MHz, $CDCl_3$) δ 172.68, 156.42, 144.32, 144.17, 141.75, 131.93, 128.17, 127.52, 125.54, 120.45, 120.43, 119.47, 67.45, 66.48, 62.88, 54.23, 47.59, 32.91, 32.47, 21.94.

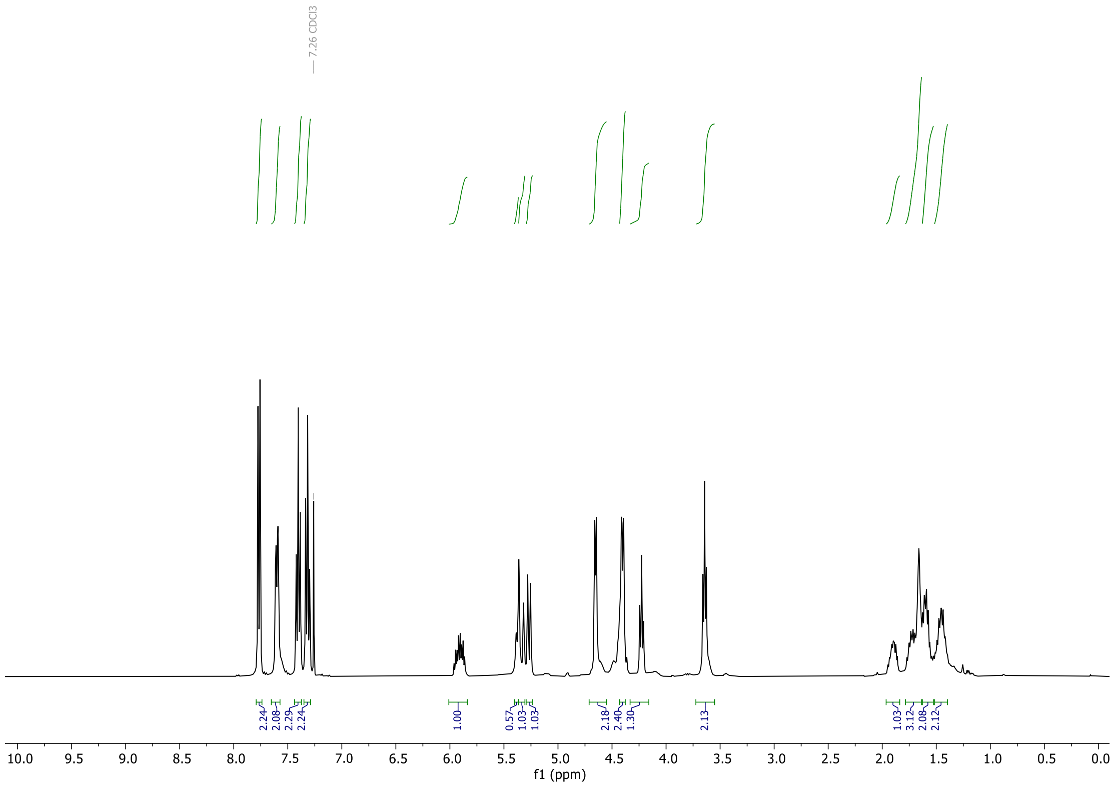

Figure 3. ^{1}H NMR (400 MHz, $CDCl_3$) spectrum of **1c.**

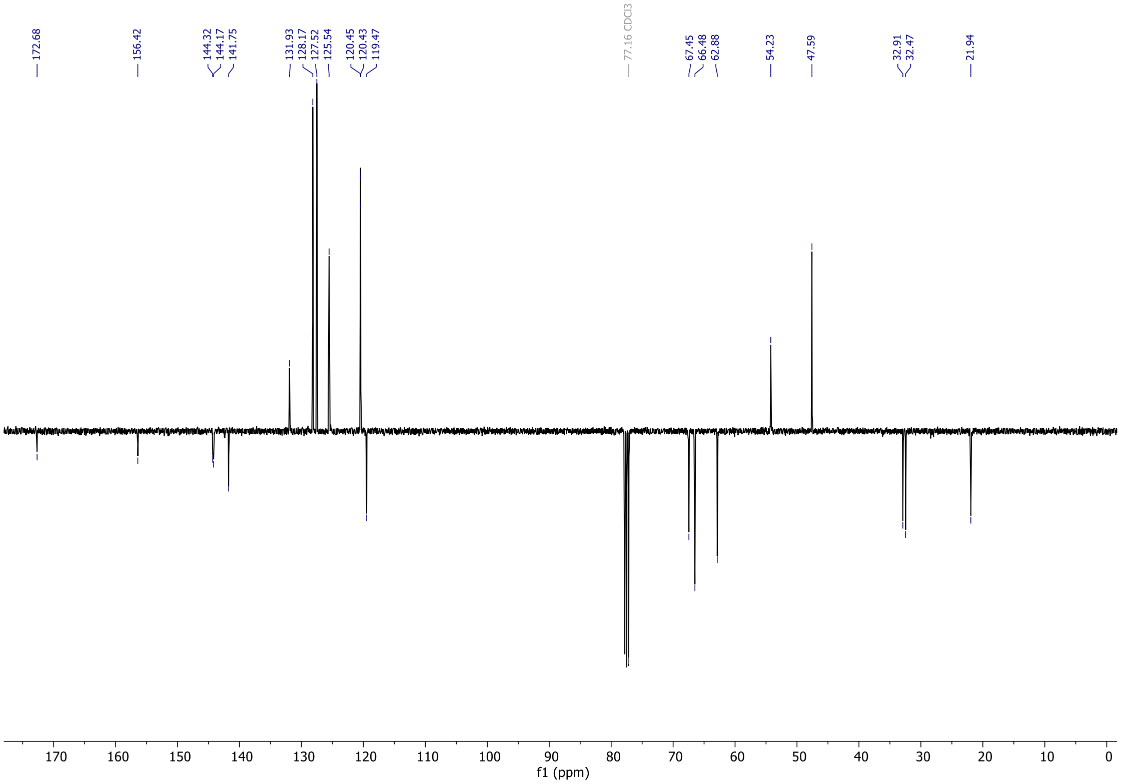

Figure 4. ^{13}C APT NMR (101 MHz, $CDCl_3$) spectrum of **1c.**

N-[(9H-fluoren-9-ylmethoxy)carbonyl]-6-hydroxy-(4-methoxytrityl)-L-norleucine allyl ester **(1d)**

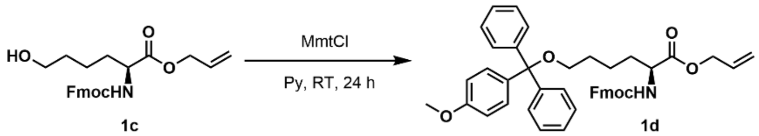

To a solution of **1c** (3.00 g, 7.33 mmol) in pyridine (20 mL), 4-methoxytrityl chloride (1.23 g, 4.4 mmol, 2.0 equiv) was added. The reaction was stirred for 24 h at room temperature. After completion, the reaction was concentrated until dryness at reduced pressure to remove pyridine. The residue was purified by column chromatography (Hex/EtOAc, gradient from 20:1 to 4:1, *v/v*) to afford **1d** (1.02 g, 1.56 mmol, 71%) as a white foam. R*f* (Hex/EtOAc 4:1, *v/v*) = 0.5. HR-MS (QTOF) m/z =704.2987 $[M+Na]^+$, calcd for $C_{44}H_{43}NO_6Na$: 704.2988. ^1H NMR (400 MHz, $CDCl_3$) δ 7.81 – 7.72 (m, 2H), 7.59 (d, *J* = 7.5 Hz, 2H), 7.44 (dd, *J* = 4.5, 2.6 Hz, 2H), 7.41 – 7.35 (m, 2H), 7.34 – 7.26 (m, 10H), 7.24 – 7.14 (m, 2H), 6.85 – 6.77 (m, 2H), 5.95 – 5.82 (m, 1H), 5.38 – 5.31 (m, 1H), 5.29 (d, *J* = 1.5 Hz, 1H), 5.28 – 5.24 (m, 1H), 5.24 – 5.19 (m, 1H), 4.68 – 4.57 (m, 2H), 4.39 (qd, *J* = 7.0, 2.8 Hz, 2H), 4.22 (q, *J* = 7.2 Hz, 1H), 3.79 (d, *J* = 8.9 Hz, 3H), 3.05 (t, *J* = 6.5 Hz, 2H), 1.97 – 1.78 (m, 1H), 1.69 – 1.57 (m, 3H), 1.48 – 1.38 (m, 2H). ^{13}C NMR (101 MHz, $CDCl_3$) δ 172.67, 158.87, 156.29, 145.25, 144.19, 141.72, 136.51, 131.95, 130.70, 129.65, 128.82, 128.27, 128.14, 128.11, 127.54, 127.48, 127.16, 125.50, 120.37, 119.32, 113.61, 113.43, 86.49, 67.39, 66.33, 63.36, 55.59, 54.32, 47.61, 32.93, 29.99, 22.48.

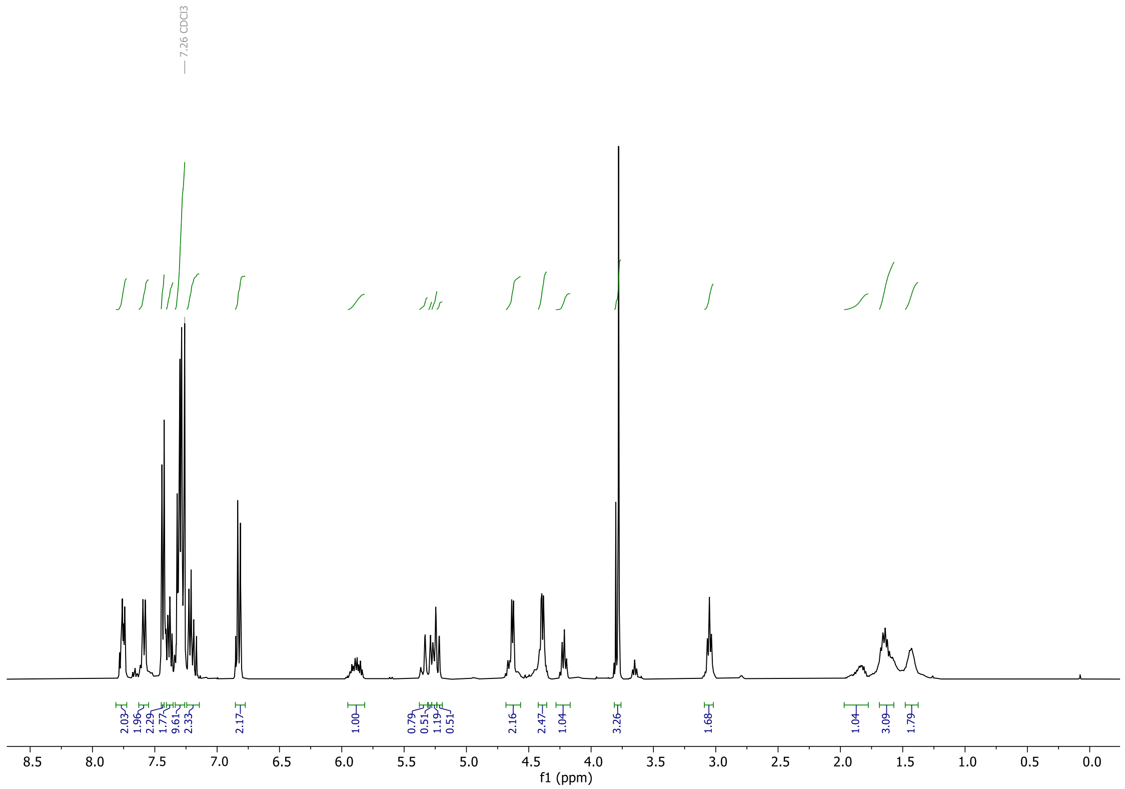

Figure 5. ^{1}H NMR (400 MHz, $CDCl_3$) spectrum of **1d**.

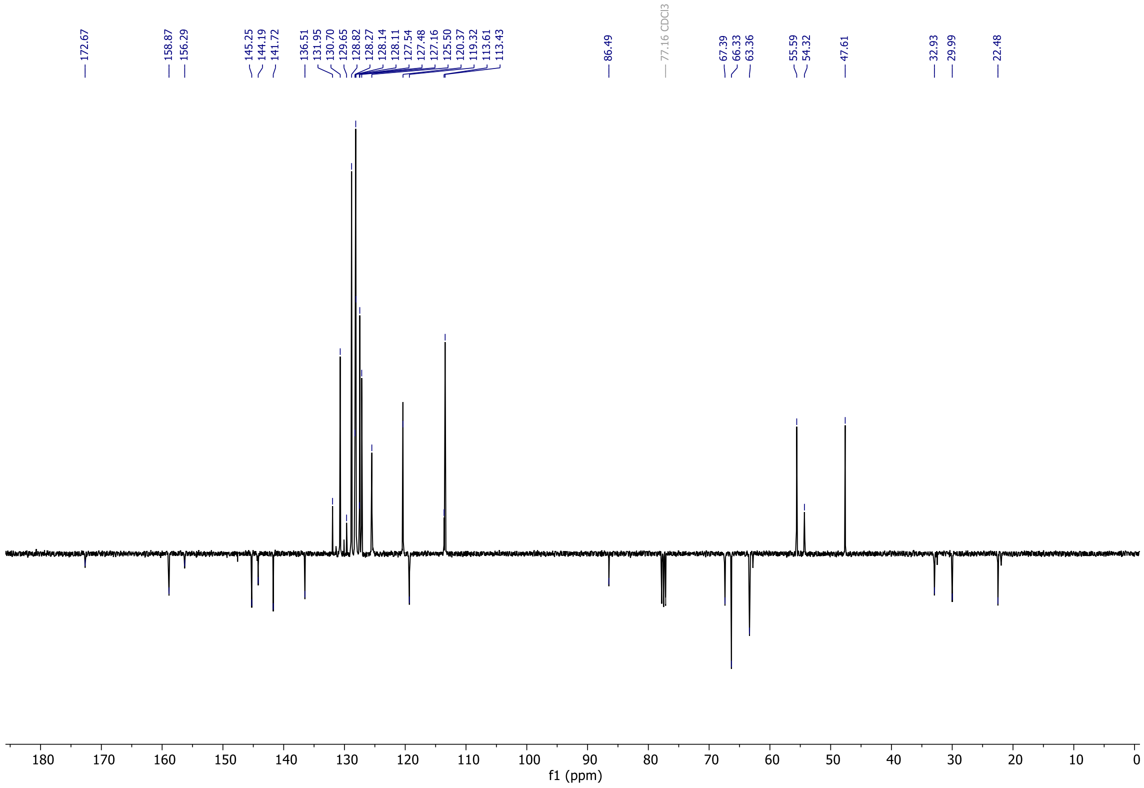

Figure 6. ^{13}C APT NMR (101 MHz, $CDCl_3$) spectrum of **1d.**

N-[(9H-fluoren-9-ylmethoxy)carbonyl]-6-hydroxy-(4-methoxytrityl)-L-norleucine pentafluorophenyl ester **(1e)**

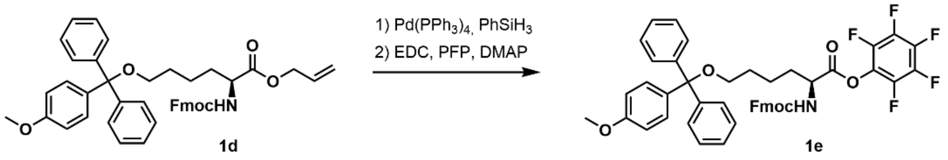

To a solution of **1d** (1.00 g, 1.46 mmol) in CH_2Cl_2 (20 mL), protected from light, tetrakis-(triphenylphosphine) palladium (0.168 g, 0.146 mmol, 0.1 equiv) was added at room temperature. Then, phenylsilane (0.545 mL, 0.438 mmol, 3.0 equiv) was added and the reaction was stirred for 5 h. After the consumption of the starting material, EDC (0.335 g, 1.75 mmol, 1.2 equiv) was added and the mixture was stirred at 0 °C for 15 min. Next, PFP (0.322 g, 1.75 mmol, 1.2 equiv) and 4-dimethylaminopyridine (0.018 g, 0.146 mmol, 0.1 equiv) were added. The reaction was stirred at room temperature for 16 hours. After completion, the crude was diluted with CH_2Cl_2 (100 mL). The organic layer was sequentially washed with sat sol of $NaHCO_3$ (50 mL) follow by 10% of citric acid (50 mL, *w/v*) and brine (50 mL). Then, the crude solution was dried over Na_2SO_4, concentrated under reduced pressure and purified by column chromatography Hex/EtOAc (gradient from 10:1 to 4:1, *v/v*) to afford **1e** (0.81 g, 0.83 mmol, 68% over two steps) as a white foam. R*f* (Hex/EtOAc 4:1, *v/v*) = 0.45. HR-MS (QTOF) m/z =830.2512 $[M+Na]^+$, calcd for $C_{47}H_{38}F_5NO_6Na$: 830.2517. ^1H NMR (400 MHz, $CDCl_3$) δ 7.76 (dd, *J* = 7.5, 2.9 Hz, 2H), 7.58 (d, *J* = 7.6 Hz, 2H), 7.45 – 7.43 (m, 2H), 7.39 (td, *J* = 7.4, 3.9 Hz, 2H), 7.35 – 7.27 (m, 8H), 7.24 – 7.16 (m, 2H), 6.87 – 6.81 (m, 2H), 5.23 (d, *J* = 8.4 Hz, 1H), 4.70 (td, *J* = 8.1, 5.1 Hz, 1H), 4.46 (dt, *J* = 6.7, 3.4 Hz, 2H), 4.23 (t, *J* = 6.8 Hz, 1H), 3.79 (d, *J* = 10.9 Hz, 3H), 3.11 (t, *J* = 6.3 Hz, 2H), 1.99 (ddd, *J* = 13.6, 8.7, 5.7 Hz, 1H), 1.91 – 1.76 (m, 1H), 1.74 – 1.65 (m, 2H), 1.55 (q, *J* = 7.6 Hz, 2H), 1.28 (d, *J* = 11.9 Hz, 2H). ^{13}C NMR (101 MHz, $CDCl_3$) δ 169.37, 159.14, 147.52, 144.18, 144.04, 141.80, 139.65, 130.73, 129.68, 128.92, 128.83, 128.36, 128.29, 128.24, 127.64, 127.55, 127.23, 125.46, 120.50, 113.66, 67.68, 62.80, 55.72, 54.16, 47.57, 32.54, 32.34, 21.98. ^{19}F NMR (376 MHz, $CDCl_3$) δ -152.26 (d, *J* = 20.3 Hz), -157.04, -161.76 (d, *J* = 21.0 Hz).

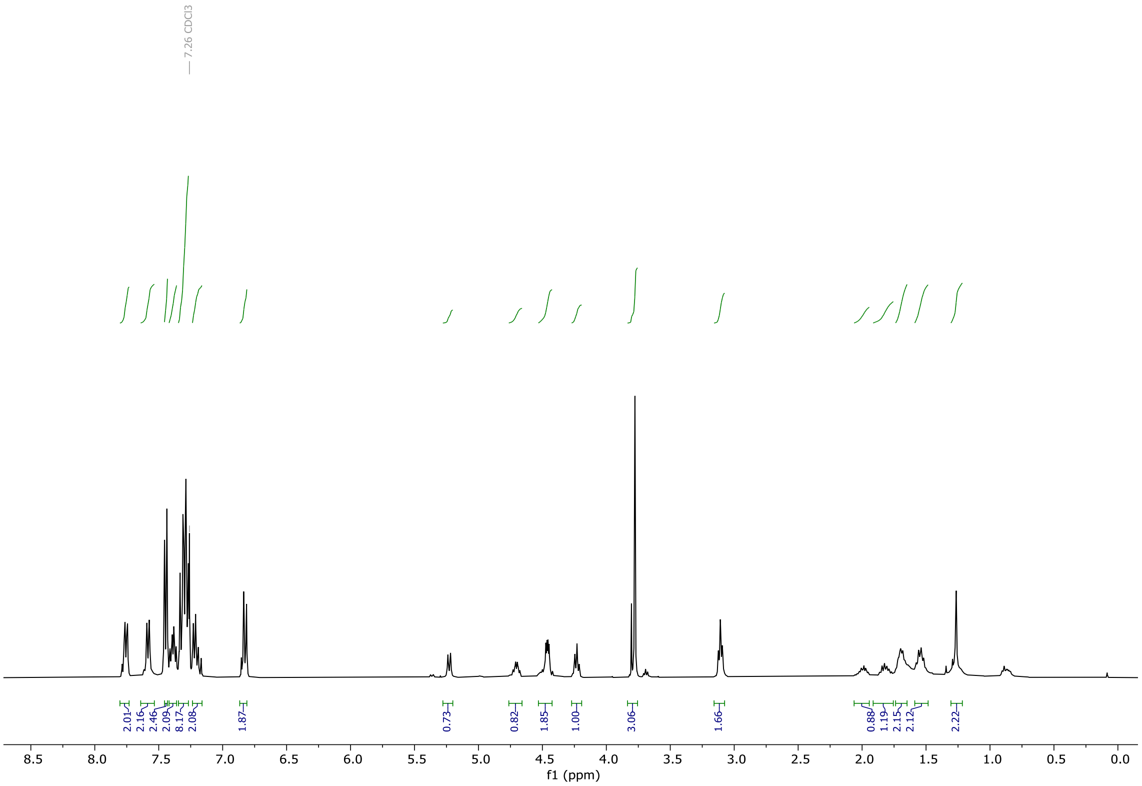

Figure 7. ^{1}H NMR (400 MHz, $CDCl_3$) spectrum of **1e**.

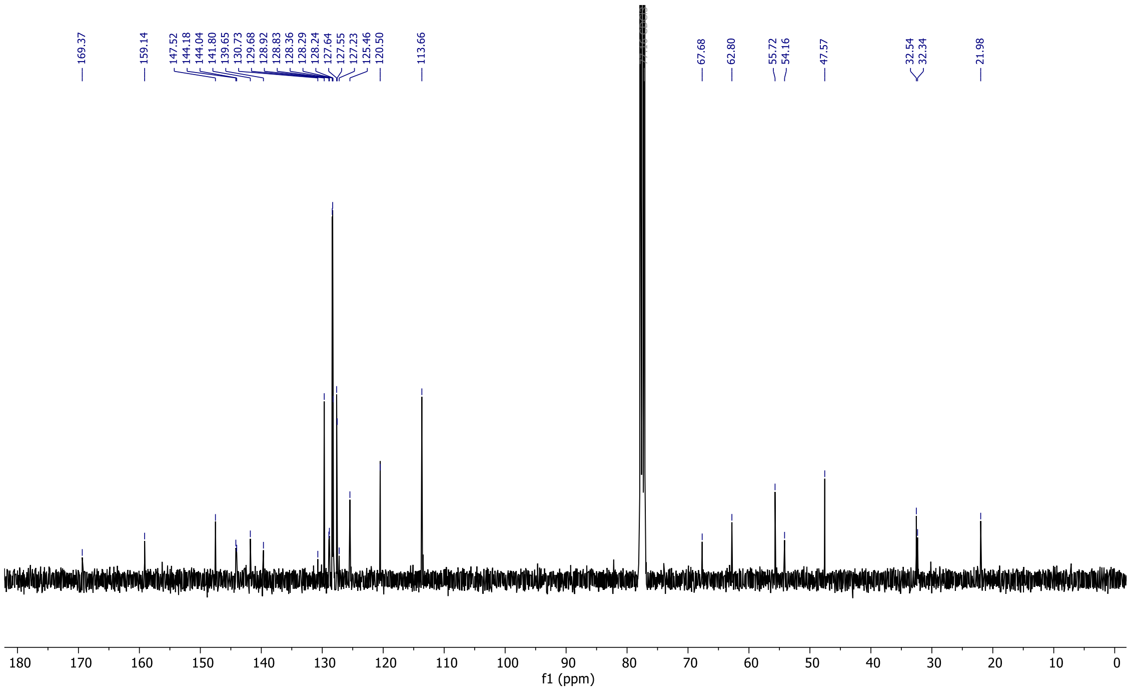

Figure 8. ^{13}C NMR (101 MHz, $CDCl_3$) spectrum of **1e.**

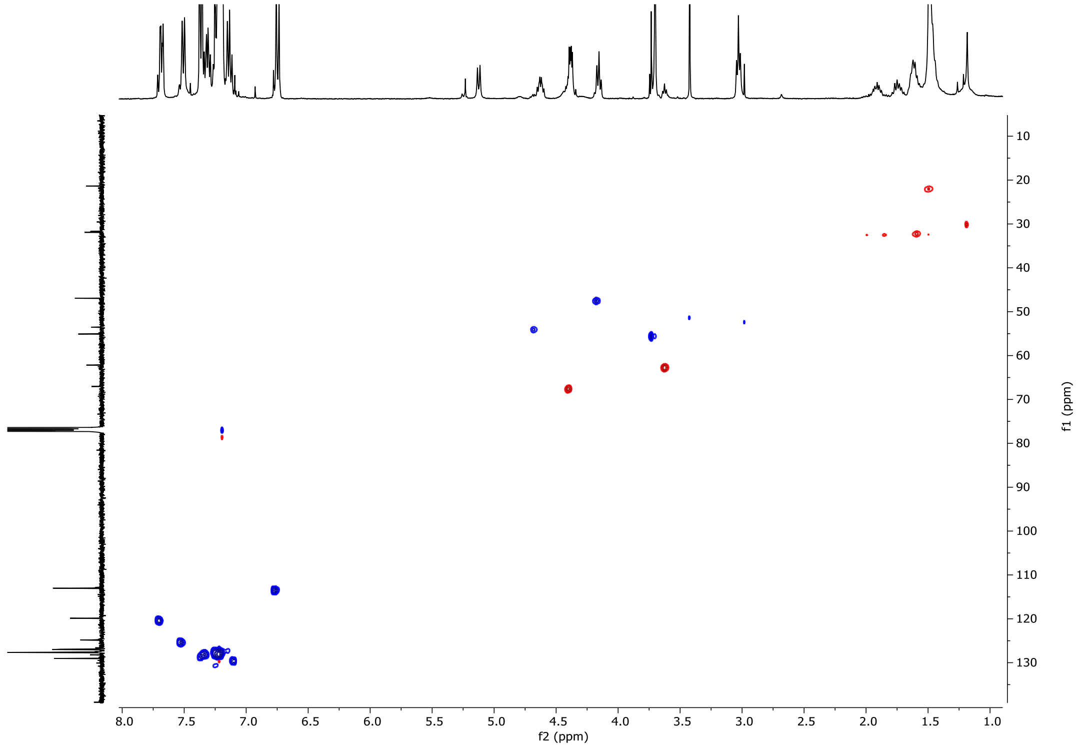

Figure 9. HSQC NMR (400 MHz, $CDCl_3$) spectrum of **1e.**

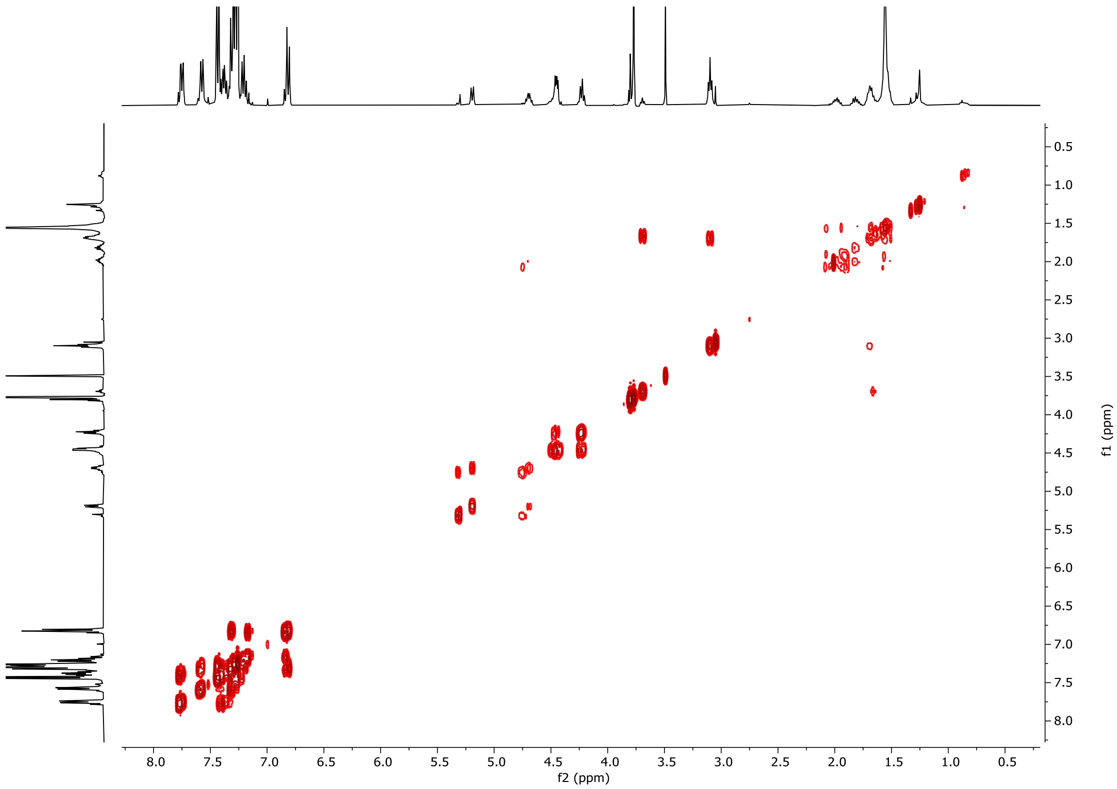

Figure 10. COSY NMR (400 MHz, $CDCl_3$) spectrum of **1e**.

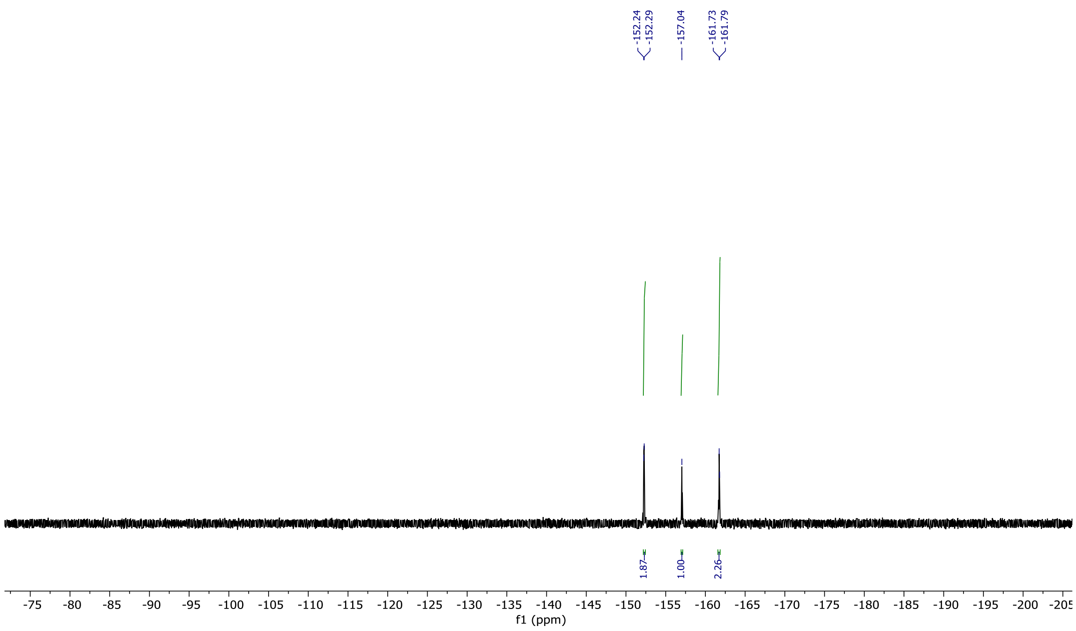

Figure 11. ^{19}F NMR (376 MHz, $CDCl_3$) spectrum of **1e.**

3.2. Synthesis of monosaccharide building blocks

Ethyl 2-O-Benzoyl-3,4,6-tri-O-benzyl-1-thio-α-D-mannopyranoside **(3)**

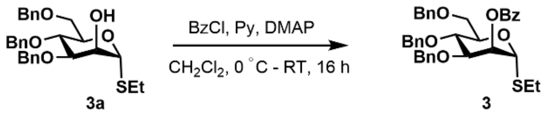

3a was prepared according to published procedures.[2]

To a solution of **3a** (4.25 g, 8.6 mmol) in anhydrous CH_2Cl_2 (20 mL), pyridine (4 mL, 38.2 mmol, 5.0 equiv) was added and stirred at 0 °C for 30 min. Then, benzoyl bromide (2.1 mL, 15.2 mmol, 2.0 equiv) was slowly added, followed by addition of DMAP (0.19 g, 1.52 mmol, 0.2 equiv). The reaction was warmed up to room temperature and stirred for 16 hours. After completion, the crude was diluted with CH_2Cl_2 (20 mL). The organic layer was washed with 10% of citric acid (10 mL, *w/v*) and brine (10 mL). Then, the crude solution was dried over Na_2SO_4, concentrated under reduced pressure and purified by column chromatography Hex/EtOAc (gradient from 20:1 to 9:1, *v/v*) to afford **3** (4.73 g, 7.9 mmol, 92%). R*f* (Hex/EtOAc 9:1, *v/v*) = 0.4. HR-MS (QTOF) m/z =621.2311 calcd for $C_{36}H_{38}O_6SNa$: 621.2287. ^1H NMR (400 MHz,

$CDCl_3$) δ 7.97 (dd, J = 8.3, 1.4 Hz, 2H), 7.45 (tt, J = 7.1, 1.3 Hz, 1H), 7.28 (d, J = 1.8 Hz, 2H), 7.25 – 7.12 (m, 13H), 7.08 (dd, J = 7.5, 2.1 Hz, 2H), 5.60 (dd, J = 3.1, 1.8 Hz, 1H), 5.33 (d, J = 2.0 Hz, 1H), 4.77 (d, J = 10.8 Hz, 1H), 4.64 (dd, J = 13.5, 11.6 Hz, 2H), 4.46 (s, 1H), 4.44 (s, 1H), 4.42 – 4.38 (m, 1H), 4.16 – 4.10 (m, 1H), 4.03 (t, J = 9.5 Hz, 1H), 3.91 (dd, J = 9.2, 3.1 Hz, 1H), 3.82 (dd, J = 10.8, 3.8 Hz, 1H), 3.65 (dd, J = 10.9, 2.0 Hz, 1H), 2.70 – 2.44 (m, 2H), 1.19 (t, J = 7.4 Hz, 3H). ^{13}C NMR (101 MHz, $CDCl_3$) δ 165.80, 138.50, 138.48, 137.84, 133.29, 130.14, 130.10, 130.01, 128.54, 128.48, 128.47, 128.45, 128.27, 128.09, 127.86, 127.78, 127.66, 127.63, 90.48, 82.71, 78.77, 75.42, 74.60, 73.52, 72.07, 71.71, 70.95, 69.10, 25.77, 15.12.

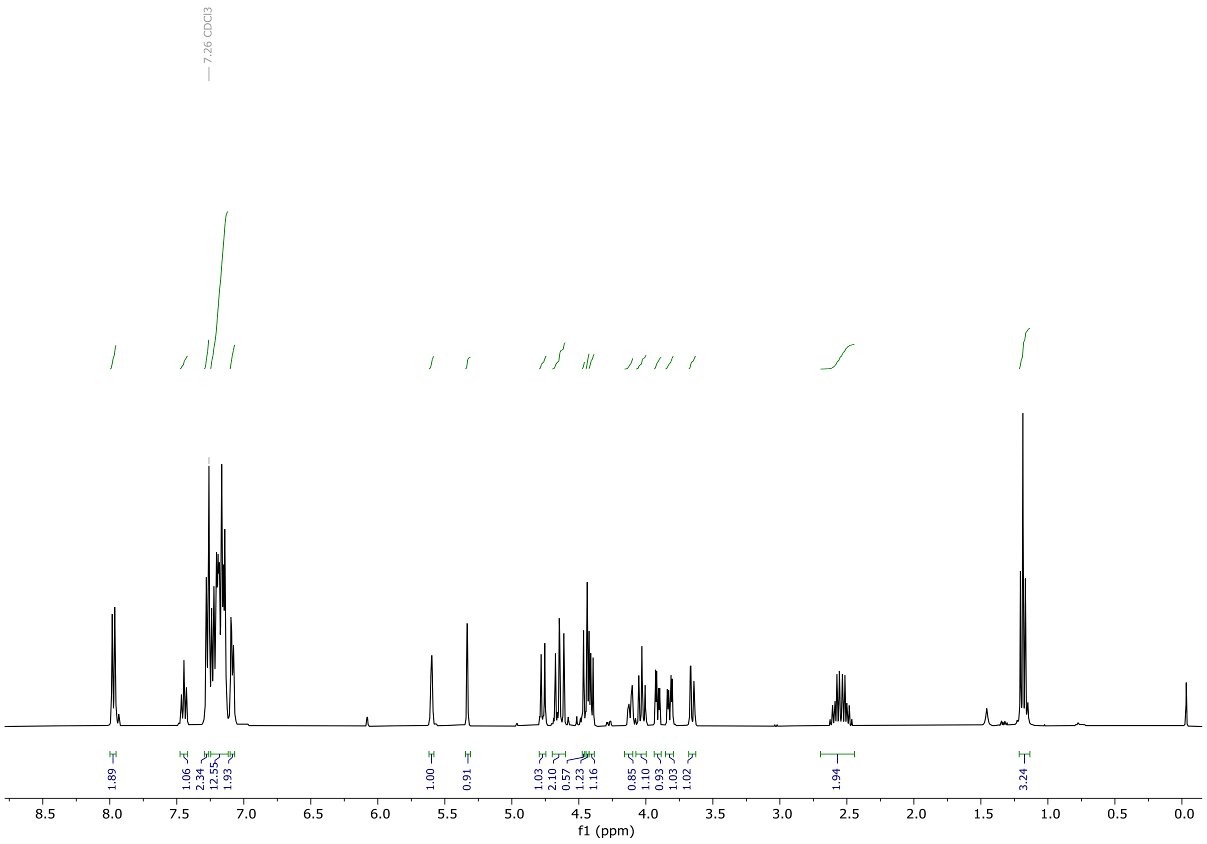

Figure 12. ^{1}H NMR (400 MHz, $CDCl_3$) spectrum of **3**.

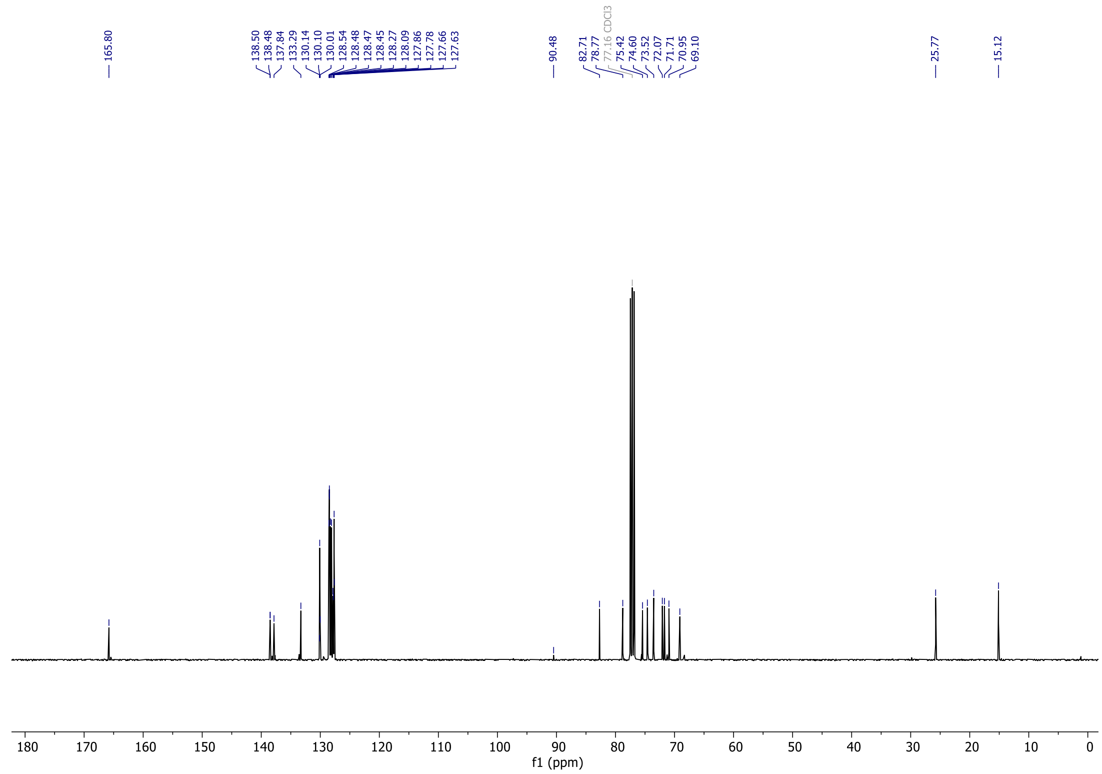

Figure 13. ^{13}C NMR (101 MHz, $CDCl_3$) spectrum of **3**.

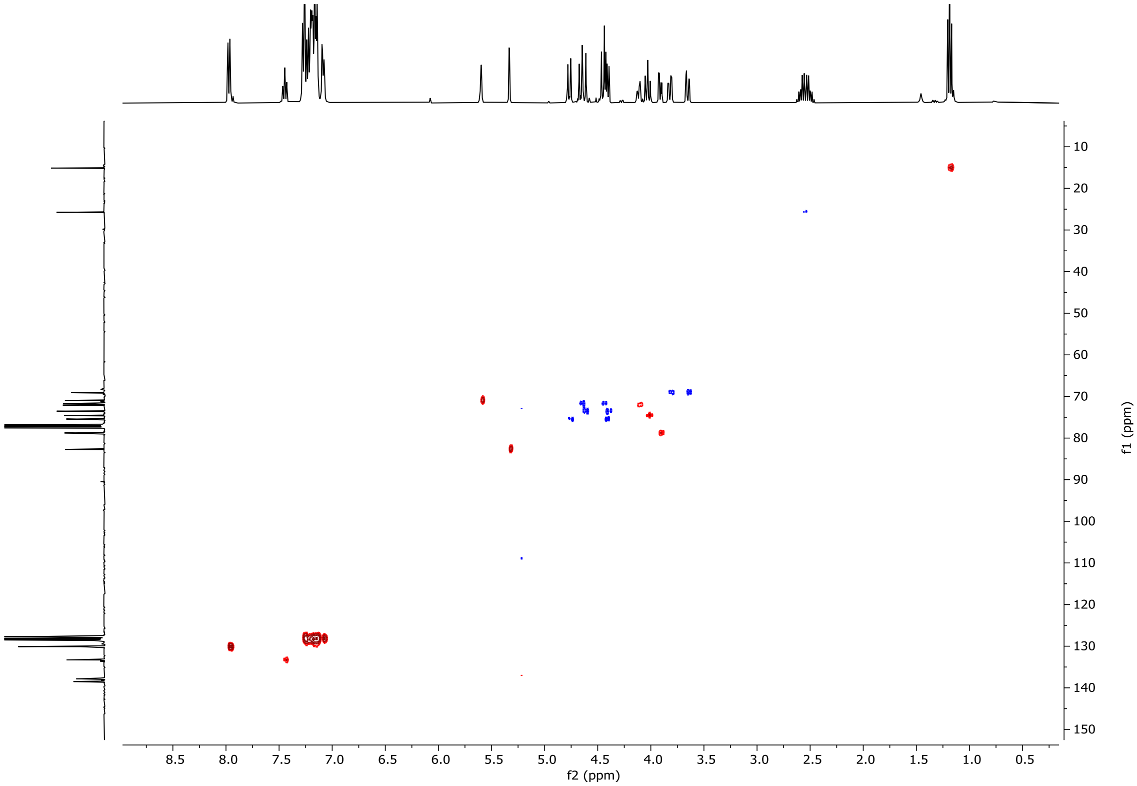

Figure 14. HSQC NMR (400 MHz, $CDCl_3$) spectrum of **3**.

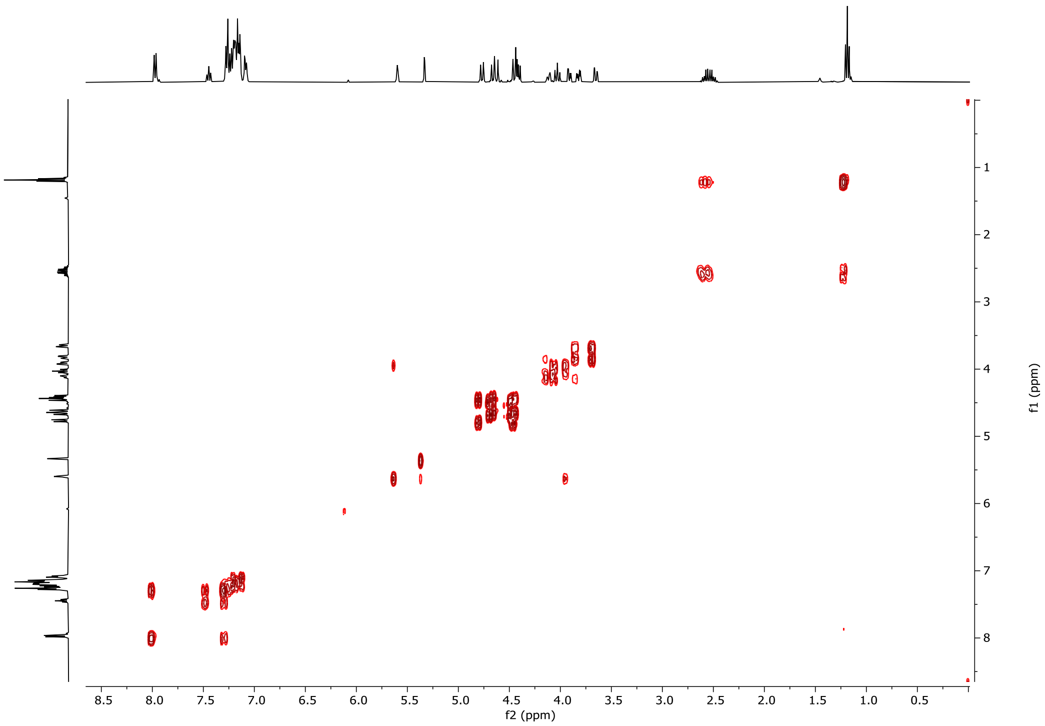

Figure 15. COSY NMR (400 MHz, $CDCl_3$) spectrum of **3**.

Ethyl 2-O-Benzoyl-4,6-bis-O-benzyl-3-O-(9-fluorenylmethoxycarbonyl)-1-thio- α-D-mannopyranoside **(4)**

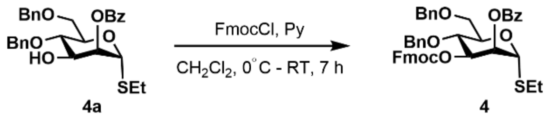

4a was prepared according to published procedures.[3]

To a solution of **4a** (0.50 g, 1.0 mmol) in anhydrous CH_2Cl_2 (10 mL), pyridine (0.4 mL, 5.0 mmol, 5.0 equiv) was added and stirred at 0 °C for 30 min. Then, fluorenylmethyloxycarbonyl chloride (0.39 g, 1.5 mmol, 1.5 equiv) was added slowly; the reaction was warmed up to room temperature and stirred overnight. After completion, the crude was diluted with CH_2Cl_2 (20 mL) and the organic phase was washed with 10% of citric acid (10 mL, *w/v*) and brine (10 mL). Then, the crude solution was dried over Na_2SO_4, concentrated under reduced pressure and purified by column chromatography Hex/Tol/EtOAc (10:10:1, *v/v*) to afford **4** (0.61 g, 0.83 mmol; 83%) R*f* (Hex/Tol/EA 4:4:1, *v/v*) = 0.5. HR-MS (QTOF) m/z =753.2549 calcd for $C_{44}H_{42}O_8SNa$: 753.2498.

^{1}H NMR (400 MHz, $CDCl_3$) δ 8.13 – 8.05 (m, 2H), 7.73 (ddt, *J* = 7.6, 2.0, 0.9 Hz, 2H), 7.61 (tt, *J* = 7.1, 1.3 Hz, 1H), 7.54 (ddd, *J* = 7.6, 2.0, 0.9 Hz, 2H), 7.42 – 7.33 (m, 8H), 7.29 – 7.23 (m, 6H), 7.15 (td, *J* = 7.5, 1.2 Hz, 1H), 5.77 (dd, *J* = 3.2, 1.7 Hz, 1H), 5.48 (d, *J* = 1.7 Hz, 1H), 5.32 – 5.21 (m, 1H), 4.79 (t, *J* = 11.9 Hz, 2H), 4.63 – 4.47 (m, 3H), 4.38 – 4.20 (m, 4H), 3.98 (dd, *J* = 10.9, 2.9 Hz, 1H), 3.79 (dd, *J* = 10.9, 1.3 Hz, 1H), 2.78 – 2.58 (m, 2H), 1.32 (t, *J* = 7.4 Hz, 3H). ^{13}C NMR (101 MHz, $CDCl_3$) δ 165.64, 154.30, 143.76, 143.24, 141.40, 141.31, 138.37, 138.01, 133.52, 130.20, 129.73, 128.64, 128.53, 128.51, 127.96, 127.91, 127.75, 127.72, 127.29, 127.23, 125.51, 125.24, 120.12, 120.07, 82.32, 75.30, 73.65, 73.15, 72.05, 72.02, 70.38, 68.83, 46.78, 25.67, 15.03.

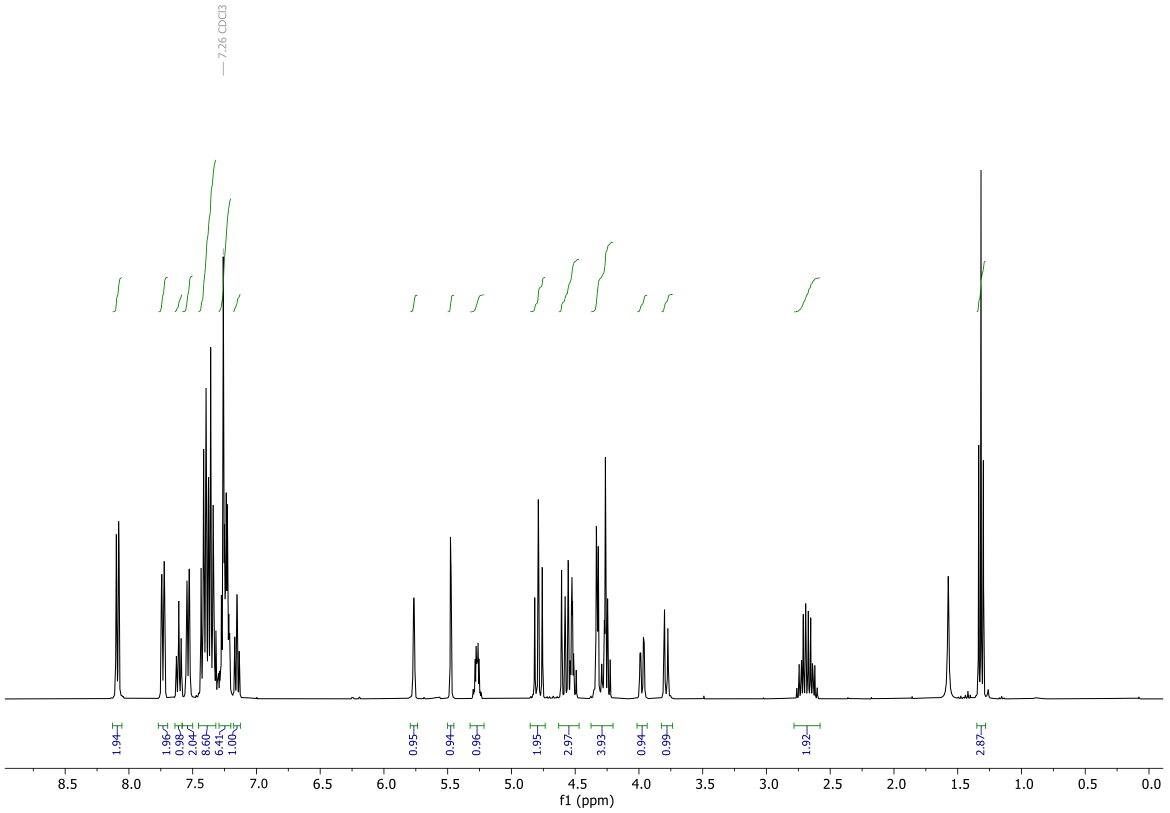

Figure 16. ^{1}H NMR (400 MHz, $CDCl_3$) spectrum of **4**.

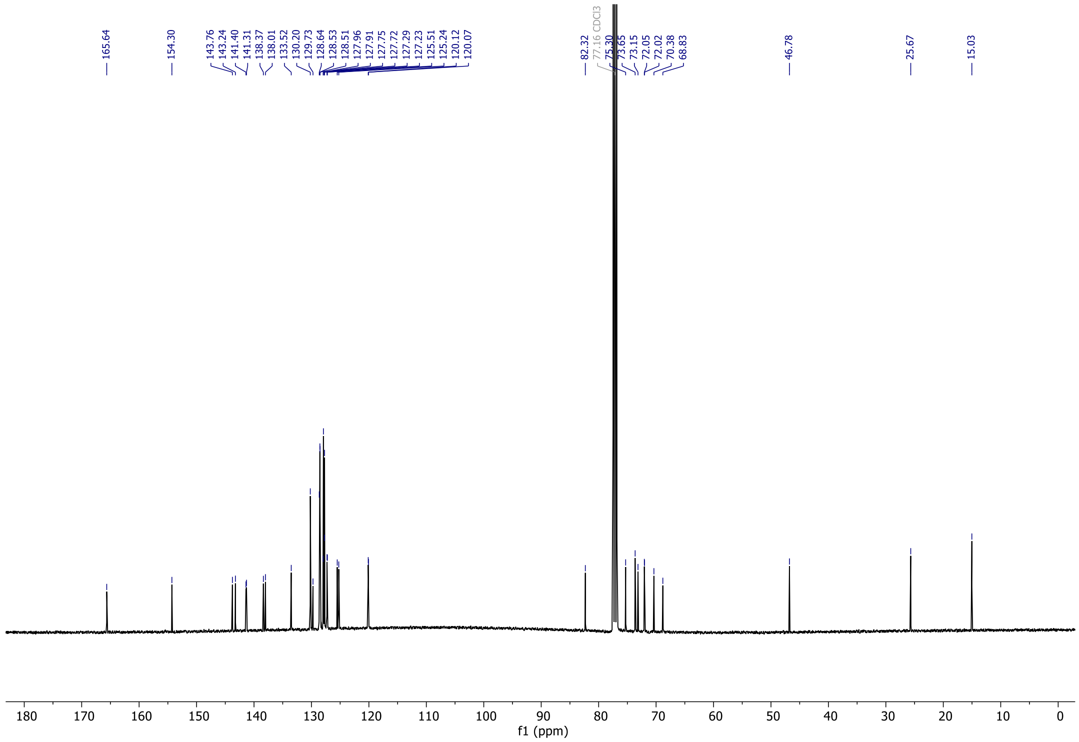

Figure 17. ^{13}C NMR (101 MHz, $CDCl_3$) spectrum of **4**.

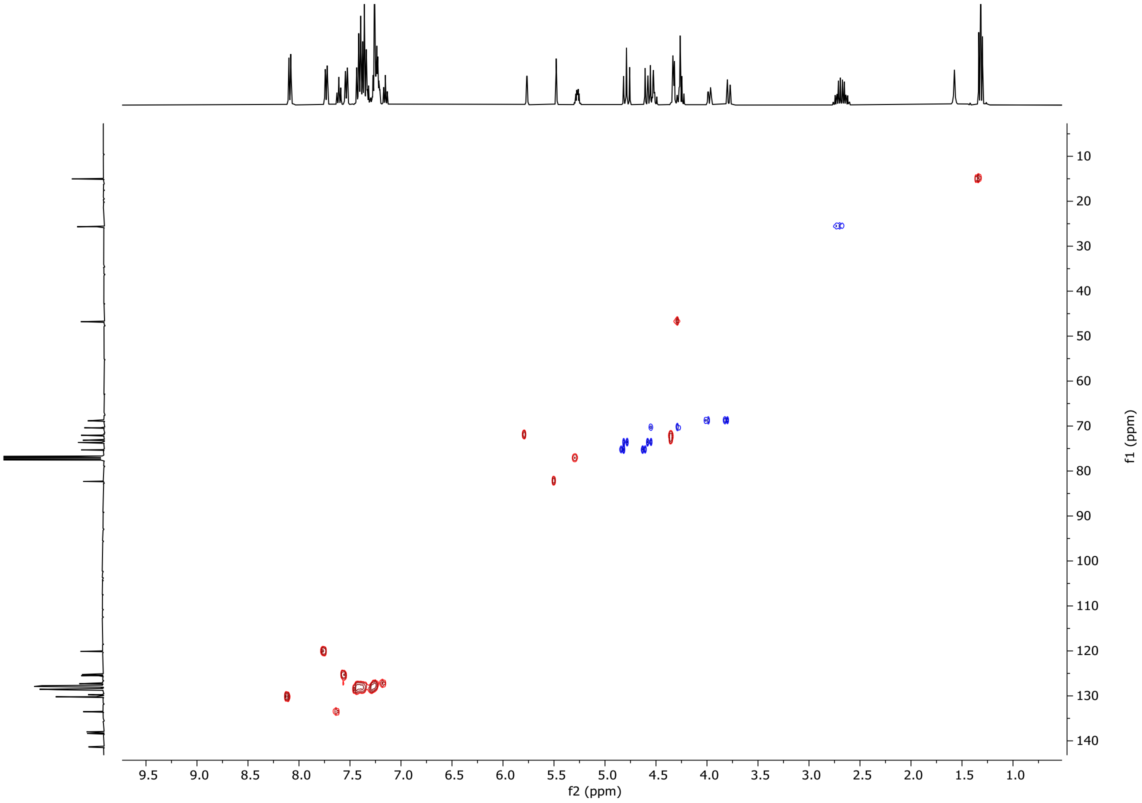

Figure 18. HSQC NMR (400 MHz, $CDCl_3$) spectrum of **4**.

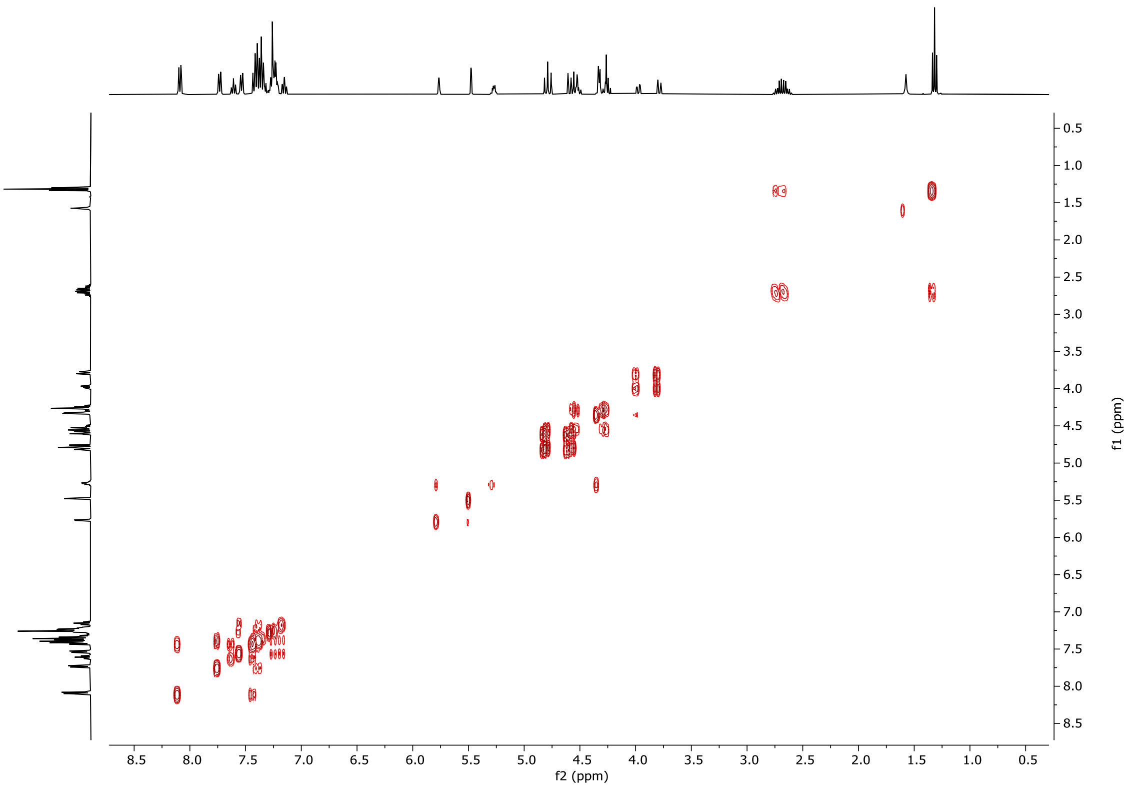

Figure 19. COSY NMR (400 MHz, $CDCl_3$) spectrum of **4**.

Galactose building blocks designed for α-glycosylation

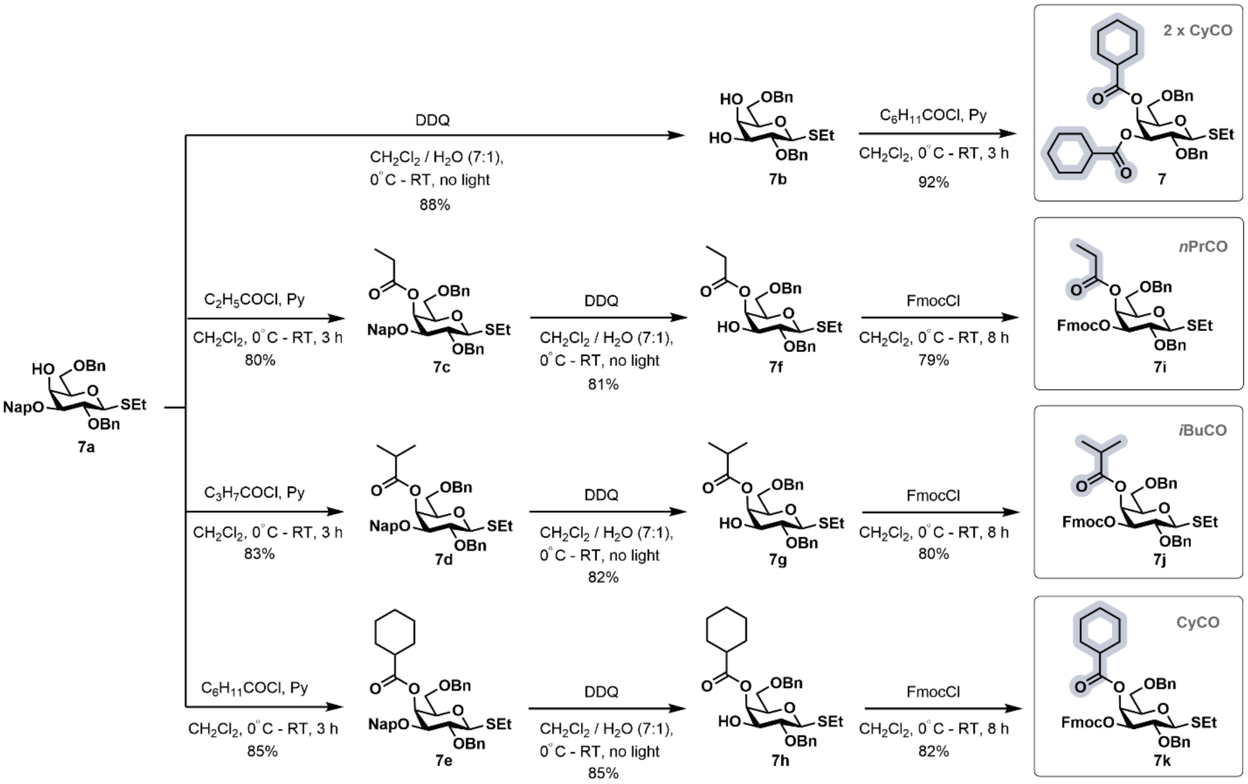

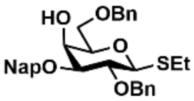

Ethyl 6-O-Benzyl-3-O-(2-napththalenylmethyl)-1-thio-β-D-galactopyranoside **(7a)**

This building block was prepared according to published procedures.[4] ^{1}H NMR (400 MHz, $CDCl_3$) δ 7.77 – 7.65 (m, 4H), 7.43 – 7.33 (m, 5H), 7.27 – 7.08 (m, 8H), 4.87 – 4.72 (m, 4H), 4.50 (s, 2H), 4.37 (d, J = 9.8 Hz, 1H), 4.05 (d, J = 3.5 Hz, 1H), 3.78 – 3.64 (m, 3H), 3.57 – 3.45 (m, 2H), 2.78 – 2.63 (m, 2H), 1.25 (t, J = 7.4 Hz, 3H). ^{13}C NMR (101 MHz, $CDCl_3$) δ 138.52, 138.23, 135.53, 133.54, 133.39, 129.40, 128.80, 128.74, 128.72, 128.59, 128.28, 128.17, 128.15, 128.07, 127.05, 126.58, 126.42, 126.14, 125.67, 85.42, 82.54, 78.25, 76.19, 74.05, 72.42, 69.70, 67.30, 25.14, 15.52.

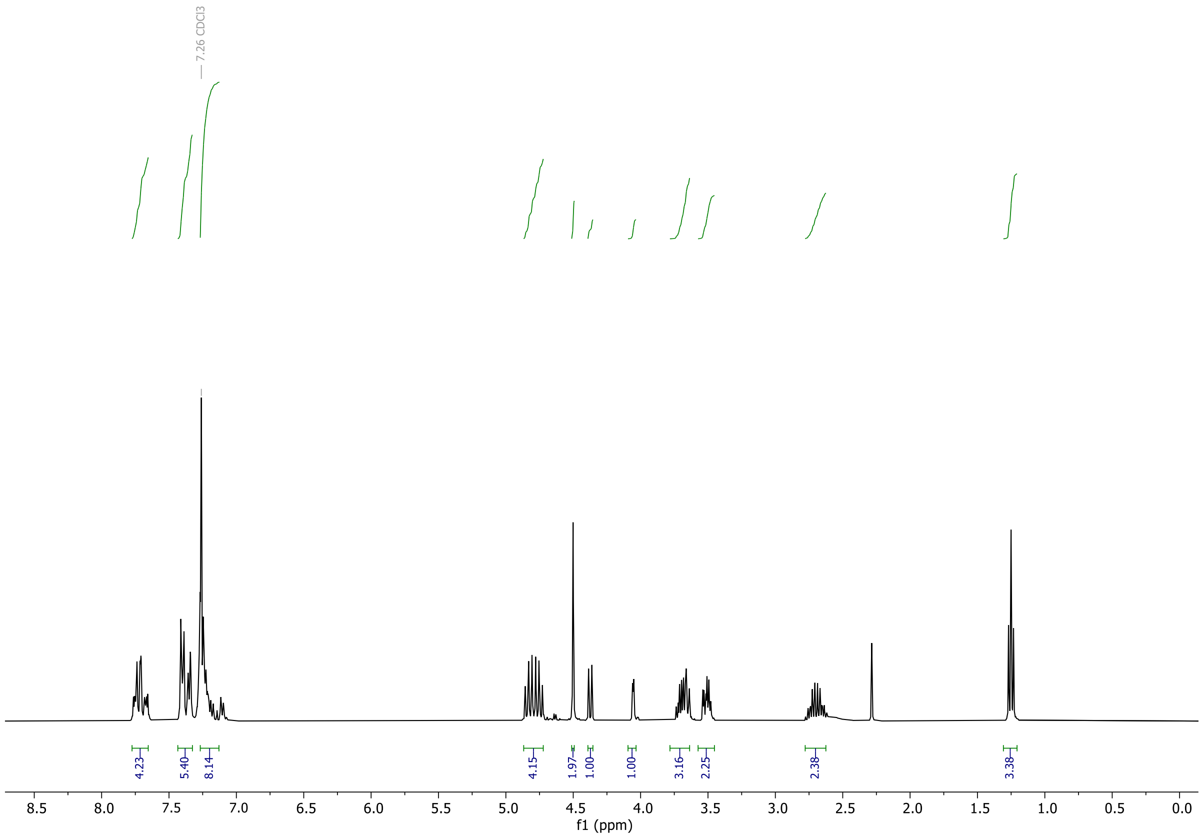

Figure 20. ^{1}H NMR (400 MHz, $CDCl_3$) spectrum of **7a**.

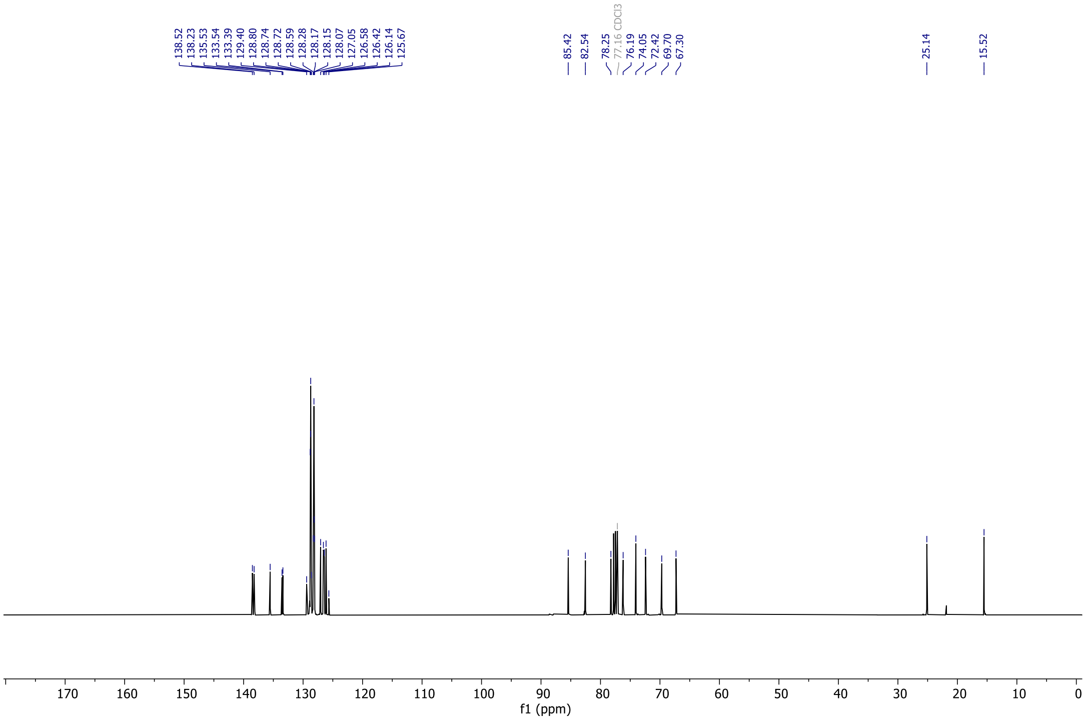

Figure 21. ^{13}C NMR (101 MHz, $CDCl_3$) spectrum of **7a.**

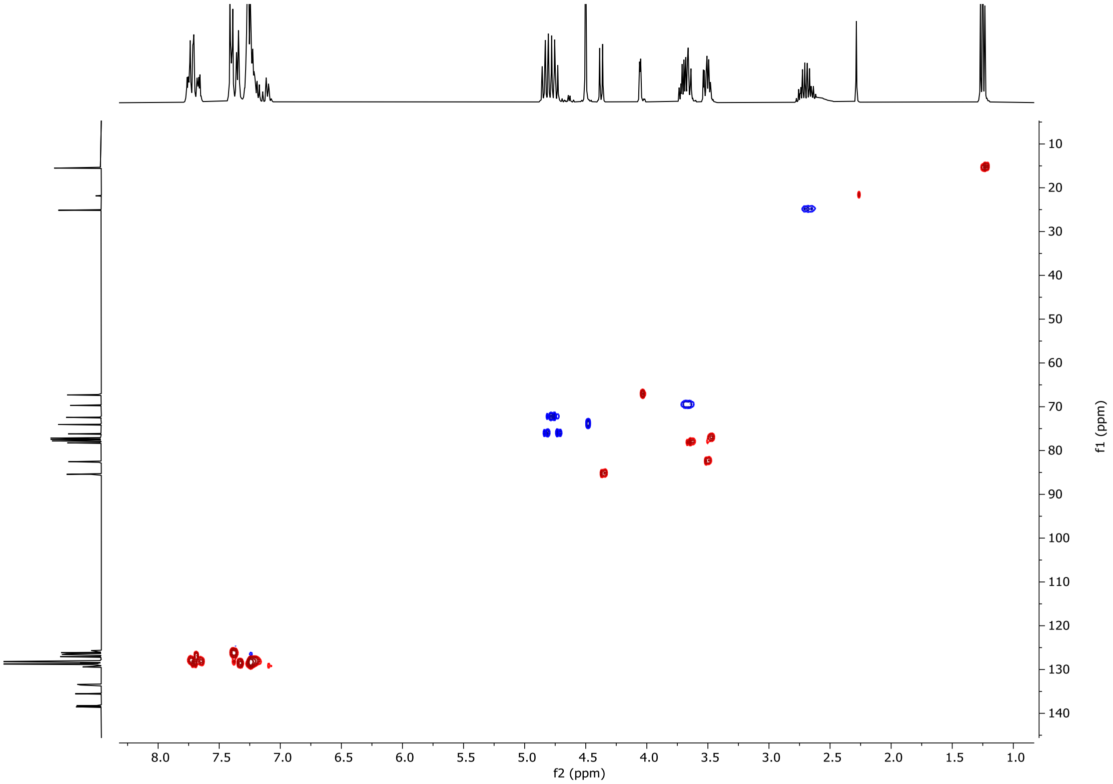

Figure 22. HSQC NMR (400 MHz, $CDCl_3$) spectrum of **7a.**

Ethyl 2,6-bis-O-benzyl-1-thio-β-D-galactopyranoside **(7b)**

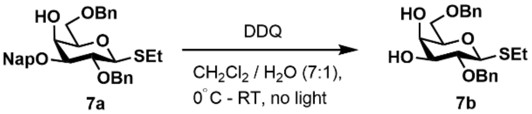

7a 7b

Building block **7a** (1.2g, 2.20 mmol) was dissolved in CH_2Cl_2/H_2O (7:1, 28 mL) and 2,3-dichloro-5,6-dicyano-1,4-benzoquinone (0.549 g, 2.40 mmol, 1.1 equiv) was added at 0 °C. Then, the suspension was stirred at room temperature for 2 h protected from light. After completion, the crude was diluted with CH_2Cl_2 (80 mL) and the organic phase was washed with sat aq sol $NaHCO_3$ (40 mL) and brine (40 mL). Then, the crude solution was dried over Na_2SO_4, concentrated under reduced pressure and purified by column chromatography Hex/EtOAc (1:1, *v/v)* to afford the corresponding compound **7b**. Yield: 88%. R*f* (Hex/EtOAc 1:1, *v/v*) = 0.35. HR-MS (QTOF) m/z =427.1548 calcd for $C_{22}H_{28}O_5SNa$: 427.1555. ^1H NMR (400 MHz, $CDCl_3$) δ 7.36 (dt, *J* = 6.0, 1.5 Hz, 2H), 7.29 (dt, *J* = 6.8, 1.5 Hz, 3H), 7.26 – 7.19 (m, 5H), 4.84 (d, *J* = 10.6 Hz, 1H), 4.66 (d, *J* = 10.6 Hz, 1H), 4.50 (s, 2H), 4.35 (dd, *J* = 9.6, 1.0 Hz, 1H), 3.90 (dd, *J* = 3.4, 1.1 Hz, 1H), 3.72 – 3.61 (m, 2H), 3.58 – 3.51 (m, 2H), 3.44 (t, *J* = 9.3 Hz, 1H), 2.74 – 2.61 (m, 2H), 1.25 (td, *J* = 7.4,

1.0 Hz, 3H), 1.18 (s, 2H). ^{13}C NMR (101 MHz, $CDCl_3$) δ 138.09, 138.02, 128.51, 128.44, 128.02, 127.90, 127.85, 85.03, 79.03, 75.51, 74.85, 73.71, 69.49, 25.03, 15.12.

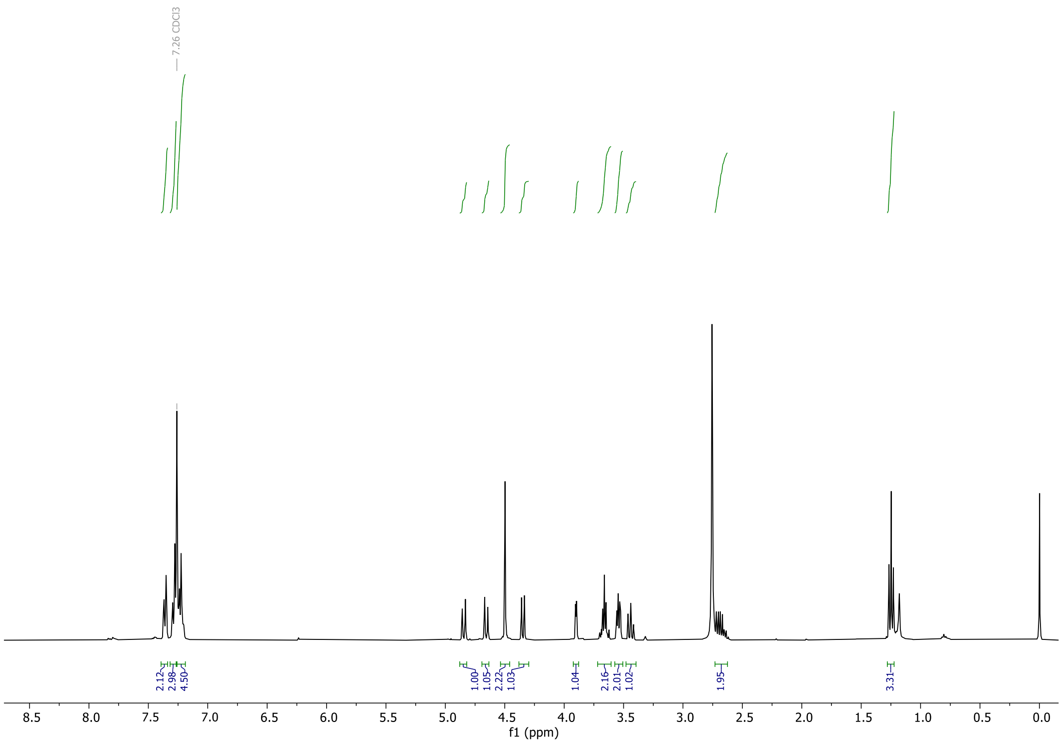

Figure 23. ^{1}H NMR (400 MHz, $CDCl_3$/MeOD) spectrum of **7b**.

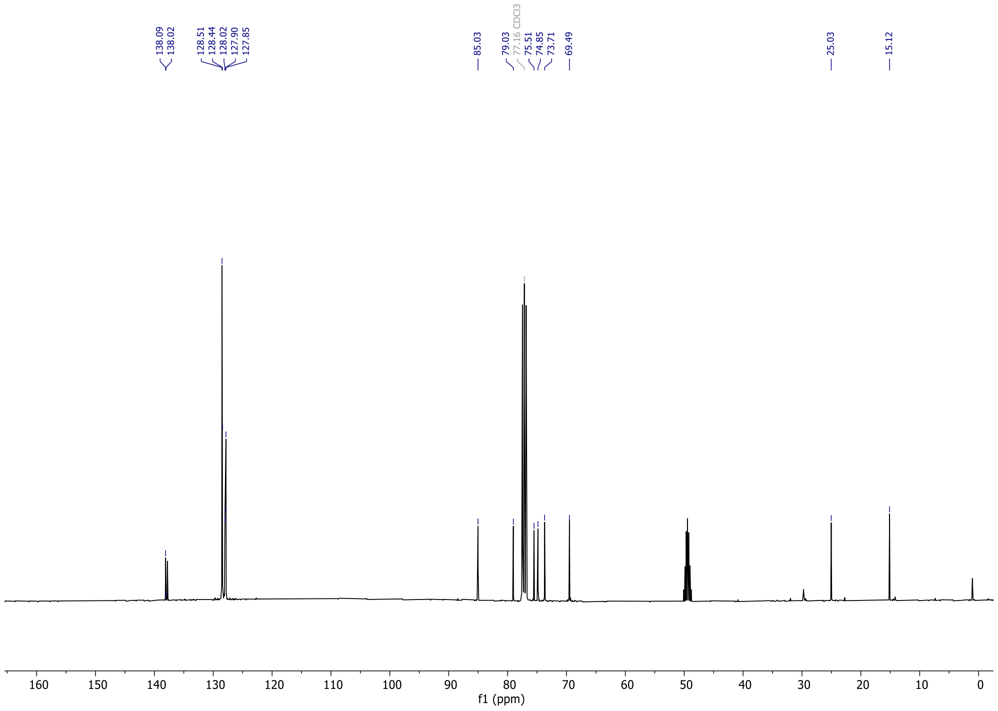

Figure 24. ^{13}C NMR (101 MHz, $CDCl_3$/MeOD) spectrum of **7b**.

General procedure for C3-/ C4-OH acylation

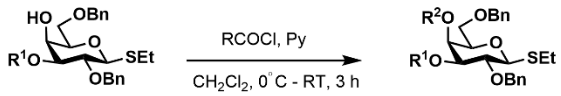

Compound	R^1
**7a**	Nap
**7a**	Nap
**7a**	Nap
**7b**	OH

Compound	R^1	R^2
**7c**	Nap	*n*PrCO
**7d**	Nap	*i*BuCO
**7e**	Nap	CyCO
**7**	CyCO	CyCO

To a solution of compound **7a** or **7b** (5.0 mmol) in anhydrous CH_2Cl_2 (25 mL), pyridine (25 mmol, 5.0 equiv) was added and stirred at 0 °C for 10 min. Then, the corresponding acyl chloride (2.0 equiv for compound **7a**; 4.0 equiv for compound **7b**) was added slowly; the reaction was warmed up to room temperature and stirred for 3 h. After completion, the crude was diluted with CH_2Cl_2 (20 mL). The organic layer was washed with 10% of citric acid (10 mL, *w/v*) and brine (10 mL). Then, the crude solution was dried over Na_2SO_4, concentrated under reduced pressure and purified by column chromatography Hex/EtOAc (10:1, *v/v*) to afford the acylated product as a colorless oil.

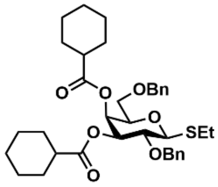

Ethyl 2,6-bis-O-Benzyl-3,4-bis-O-cyclohexanecarbonyl-1-thio-β-D-galactopyranoside **(7)**

Yield: 92%. R*f* (Hex/EtOAc 10:1, *v/v*) = 0.4. HR-MS (QTOF) m/z = 647.3038, calcd. for $C_{36}H_{48}O_7SNa$: 647.3018. ^{1}H NMR (400 MHz, $CDCl_3$) δ 7.30 – 7.21 (m, 10H), 5.45 (dd, *J* = 3.4, 1.0 Hz, 1H), 5.00 (dd, *J* = 9.7, 3.4 Hz, 1H), 4.78 (d, *J* = 10.8 Hz, 1H), 4.58 – 4.43 (m, 3H), 4.37 (d, *J* = 11.9 Hz, 1H), 3.80 (td, *J* = 6.3, 1.1 Hz, 1H), 3.58 (t, *J* = 9.7 Hz, 1H), δ 3.49 (dd, *J* = 9.7, 6.2 Hz, 1H), 3.38 (dd, *J* = 9.7, 6.4 Hz, 1H), 2.73 (qd, *J* = 7.5, 5.4 Hz, 2H), 2.29 (tt, *J* = 11.1, 3.6 Hz, 1H), 2.08 (tq, *J* = 11.4, 3.7 Hz, 1H), 1.84 – 1.76 (m, 4H), 1.74 – 1.65 (m, 4H), 1.64 – 1.54 (m, 2H), 1.42 – 1.22 (m, 10H), 1.23 – 1.07 (m, 3H). ^{13}C NMR (101 MHz, $CDCl_3$) δ 174.98, 174.83, 137.82, 137.73, 128.52, 128.42, 128.15, 127.99, 127.94, 127.88, 85.56, 76.30, 75.98, 75.44, 74.06, 73.70, 68.24, 67.86, 43.19, 43.09, 29.26, 29.11, 28.87, 28.78, 25.81, 25.56, 25.50, 25.41, 25.38, 15.15.

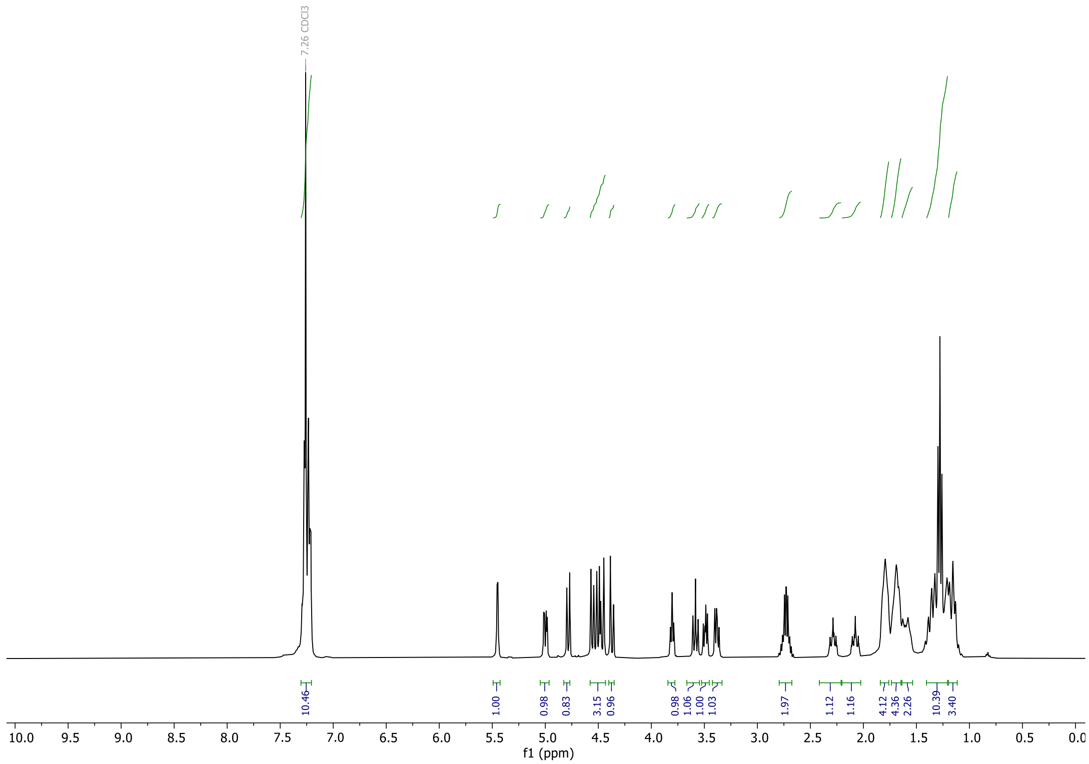

Figure 25. ^{1}H NMR (400 MHz, $CDCl_3$) spectrum of **7.**

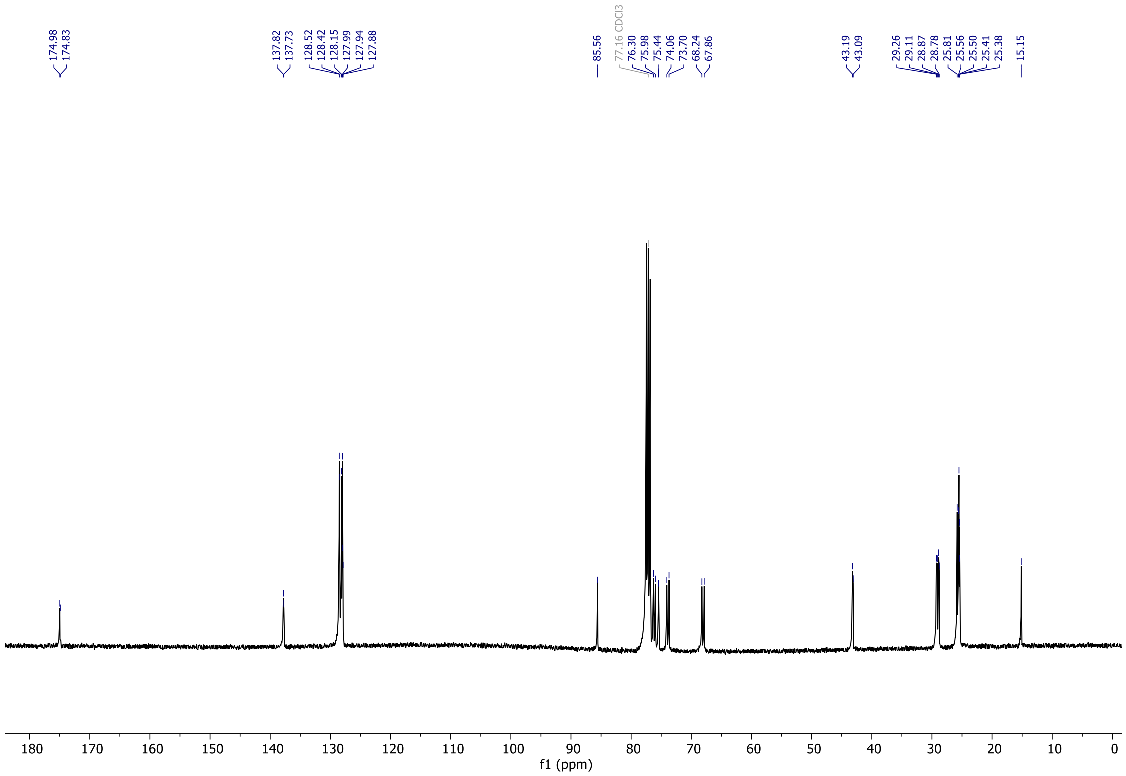

Figure 23. ^{13}C NMR (101 MHz, $CDCl_3$) spectrum of **7.**

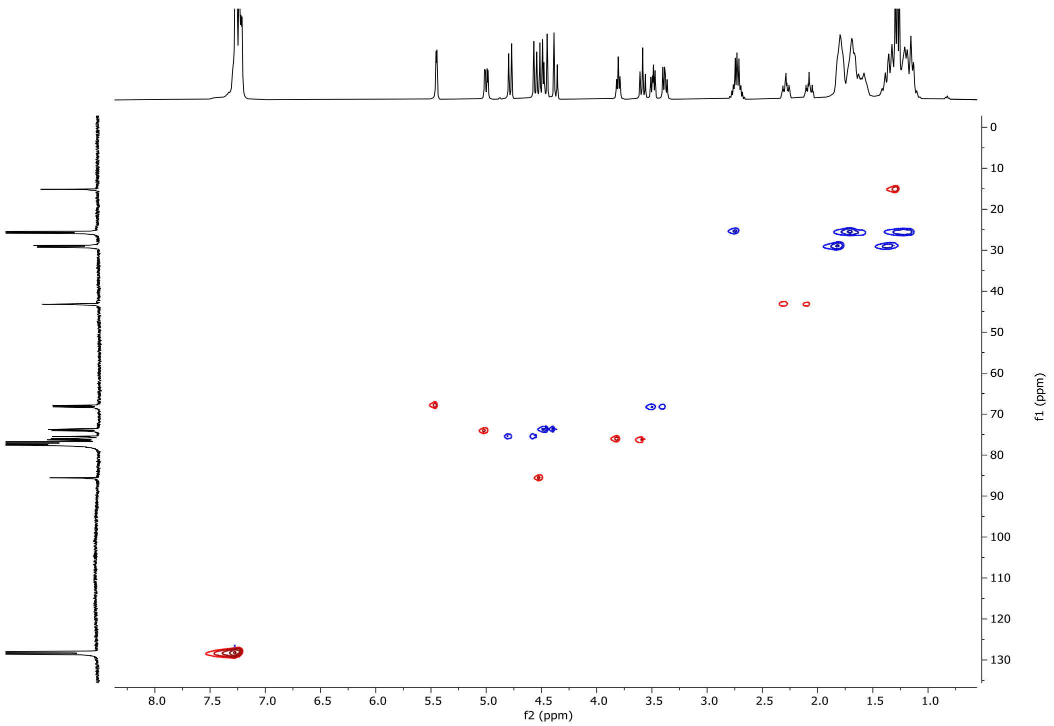

Figure 27. HSQC NMR (400 MHz, $CDCl_3$) spectrum of **7.**

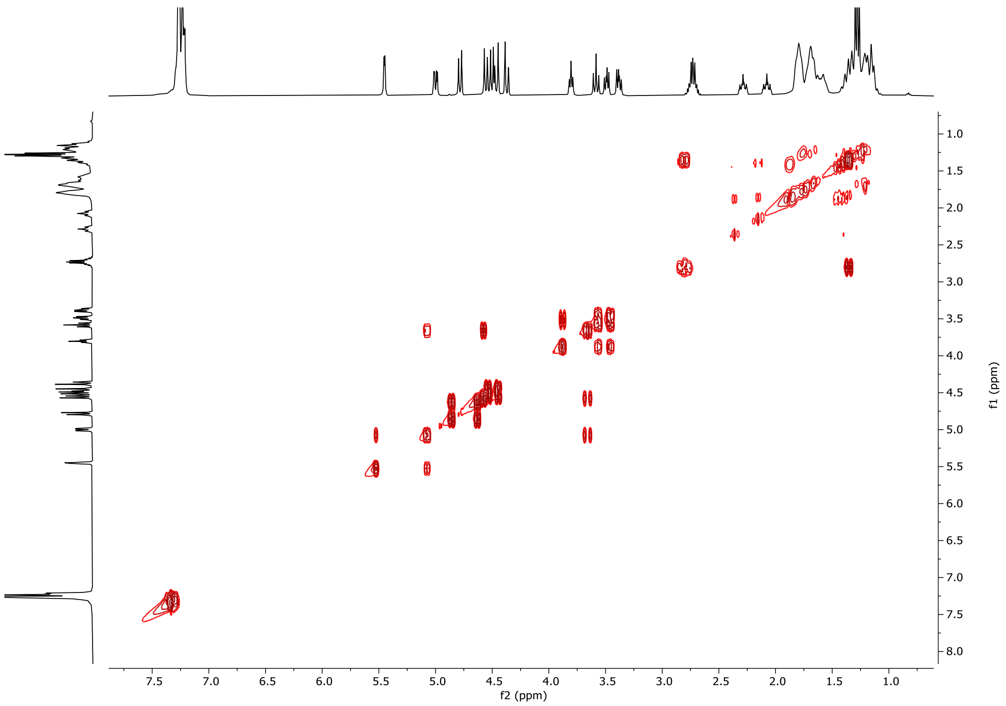

Figure 28. COSY NMR (400 MHz, $CDCl_3$) spectrum of **7.**

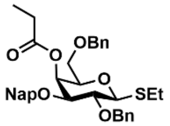

Ethyl 2,6-bis-O-Benzyl-3-O-(2-napththalenylmethyl)-4-O-propanoate-1-thio-β-D-galactopyranoside **(7c)**

Yield: 80%. R*f* (Hex/EtOAc 3:1, *v/v*) = 0.43. HR-MS (QTOF) m/z = 623.2450, calcd. for $C_{36}H_{40}O_6SNa$: 623.2443. ^{1}H NMR (400 MHz, $CDCl_3$) δ 7.86 – 7.74 (m, 3H), 7.74 – 7.67 (m, 1H), 7.53 – 7.42 (m, 3H), 7.41 – 7.36 (m, 3H), 7.36 – 7.28 (m, 7H), 5.72 (dd, *J* = 3.3, 1.1 Hz, 1H), 4.95 (d, *J* = 11.4 Hz, 1H), 4.86 – 4.77 (m, 2H), 4.68 (d, *J* = 11.4 Hz, 1H), 4.57 (d, *J* = 11.8 Hz, 1H), 4.52 – 4.45 (m, 2H), 3.76 (ddd, *J* = 6.9, 5.8, 1.1 Hz, 1H), 3.69 (dd, *J* = 9.1, 3.3 Hz, 1H), 3.65 – 3.56 (m, 2H), 3.52 (dd, *J* = 9.4, 6.9 Hz, 1H), 2.86 – 2.68 (m, 2H), 2.52 – 2.30 (m, 2H), 1.33 (t, *J* = 7.4 Hz, 3H), 1.17 (t, *J* = 7.6 Hz, 3H). ^{13}C NMR (101 MHz, $CDCl_3$) δ 174.05, 138.25, 137.71, 135.35, 133.35, 133.11, 128.56, 128.44, 128.19, 128.16, 128.06, 127.99, 127.89, 127.76, 127.08, 126.33, 126.10, 125.98, 85.46, 81.09, 77.89, 75.97, 73.83, 71.96, 68.33, 66.85, 27.62, 25.14, 15.19, 9.44.

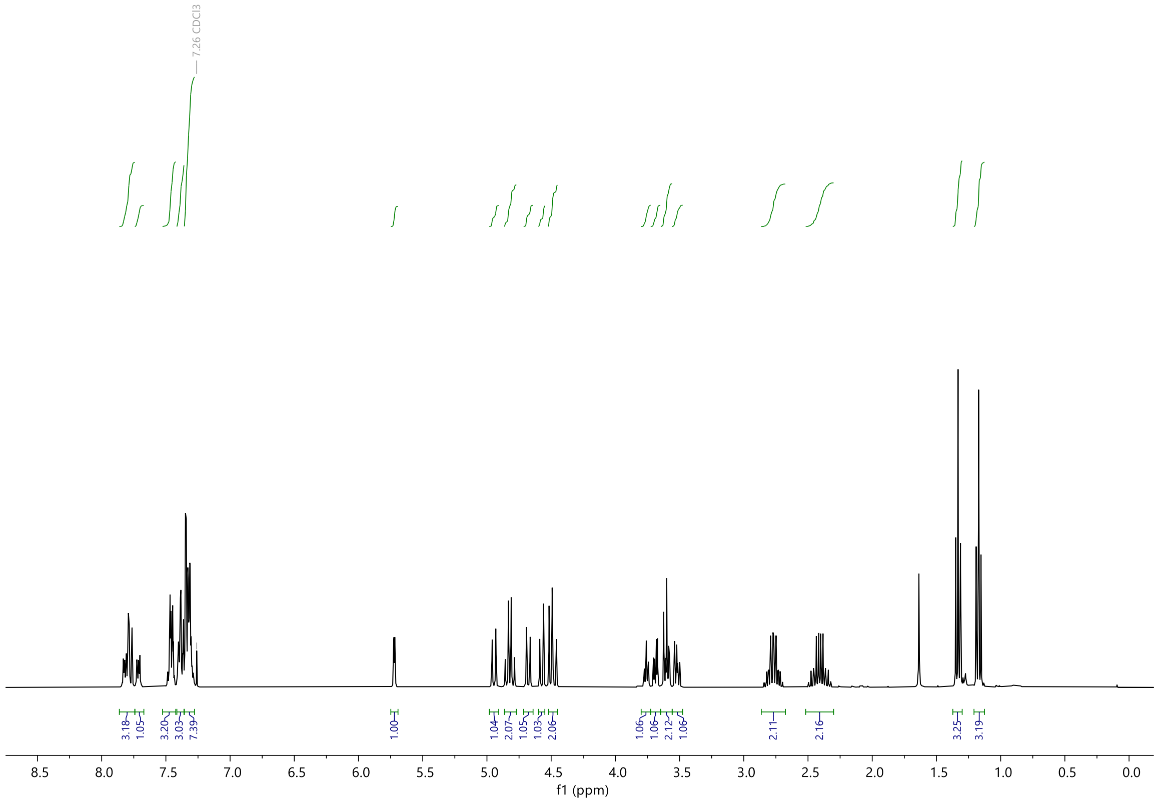

Figure 24. ^{1}H NMR (400 MHz, $CDCl_3$) spectrum of **7c.**

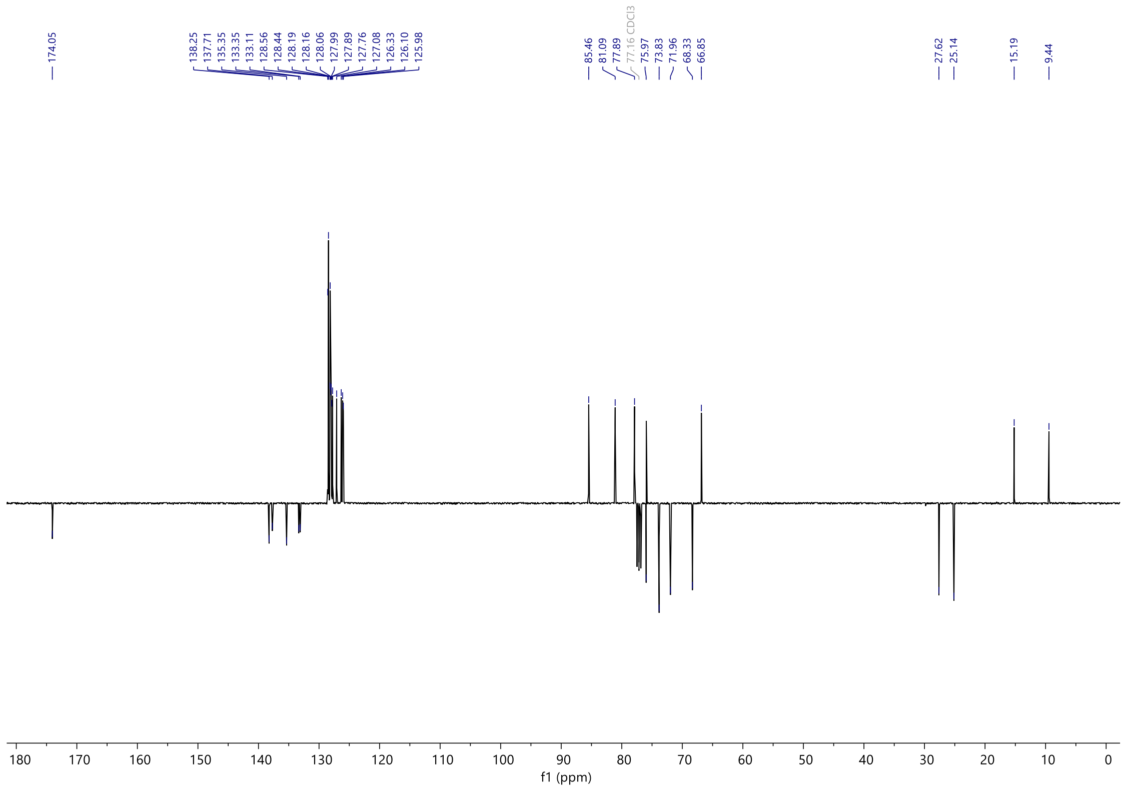

Figure 25. ^{13}C NMR (101 MHz, $CDCl_3$) spectrum of **7c.**

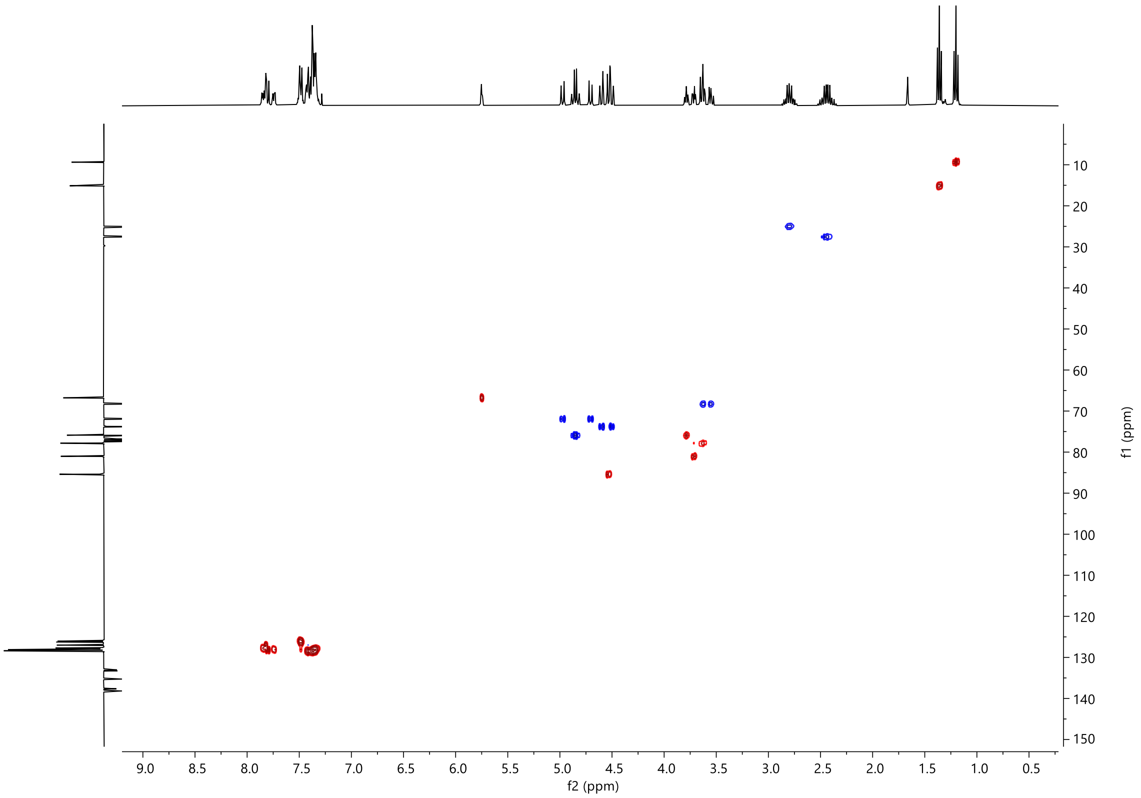

Figure 31. HSQC NMR (400 MHz, $CDCl_3$) spectrum of **7c.**

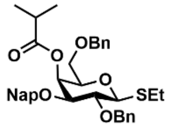

Ethyl 2,6-bis-O-Benzyl-3-O-(2-napththalenylmethyl)-4-O-(2-methylpropanoate)-1-thio-β-D-galactopyranoside **(7d)**

Yield: 83%. R*f* (Hex/EtOAc 3:1, *v/v*) = 0.45. HR-MS (QTOF) m/z = 637.2609, calcd. for $C_{37}H_{42}O_6SNa$: 637.2600. ^{1}H NMR (400 MHz, $CDCl_3$) δ 7.80 – 7.74 (m, 3H), 7.72 (s, 1H), 7.70 – 7.65 (m, 1H), 7.44 – 7.38 (m, 3H), 7.35 (dq, *J* = 5.9, 2.2 Hz, 2H), 7.33 – 7.25 (m, 7H), 5.67 (dd, *J* = 3.3, 1.1 Hz, 1H), 4.89 (d, *J* = 11.3 Hz, 1H), 4.79 (q, *J* = 10.3 Hz, 2H), 4.62 (d, *J* = 11.3 Hz, 1H), 4.51 (d, *J* = 11.7 Hz, 1H), 4.49 – 4.42 (m, 2H), 3.73 (ddd, *J* = 6.9, 5.8, 1.1 Hz, 1H), 3.64 (dd, *J* = 9.2, 3.3 Hz, 1H), 3.62 – 3.53 (m, 2H), 3.48 (dd, *J* = 9.5, 6.8 Hz, 1H), 2.81 – 2.71 (m, 2H), 2.71 – 2.56 (m, 2H), 1.29 (t, *J* = 7.4 Hz, 3H), 1.16 (s, 2H), 1.14 (d, *J* = 2.5 Hz, 3H). ^{13}C NMR (101 MHz, $CDCl_3$) δ 176.81, 138.47, 138.01, 135.70, 133.60, 133.34, 128.84, 128.76, 128.68, 128.36, 128.30, 128.24, 128.14, 128.02, 127.27, 126.60, 126.35, 126.21, 85.63, 81.45, 77.98, 76.26, 76.16, 74.11, 72.15, 68.68, 66.90, 34.39, 25.24, 19.67, 19.43, 15.46.

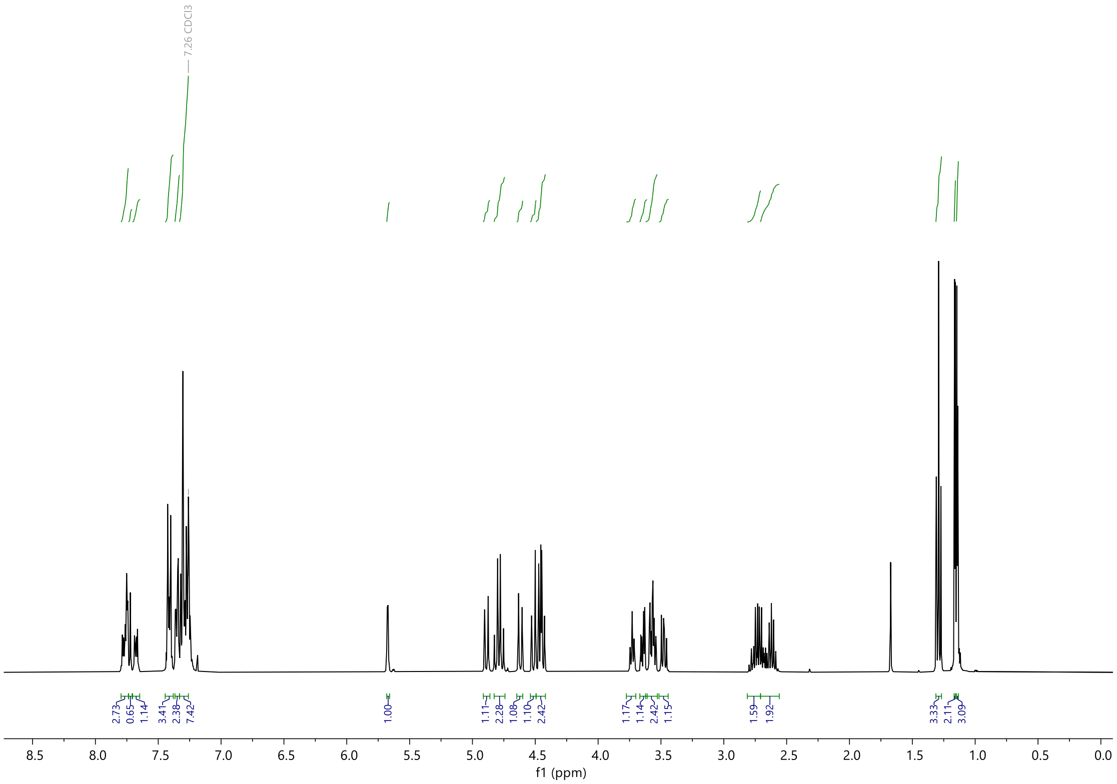

Figure 32. ^{1}H NMR (400 MHz, $CDCl_3$) spectrum of **7d.**

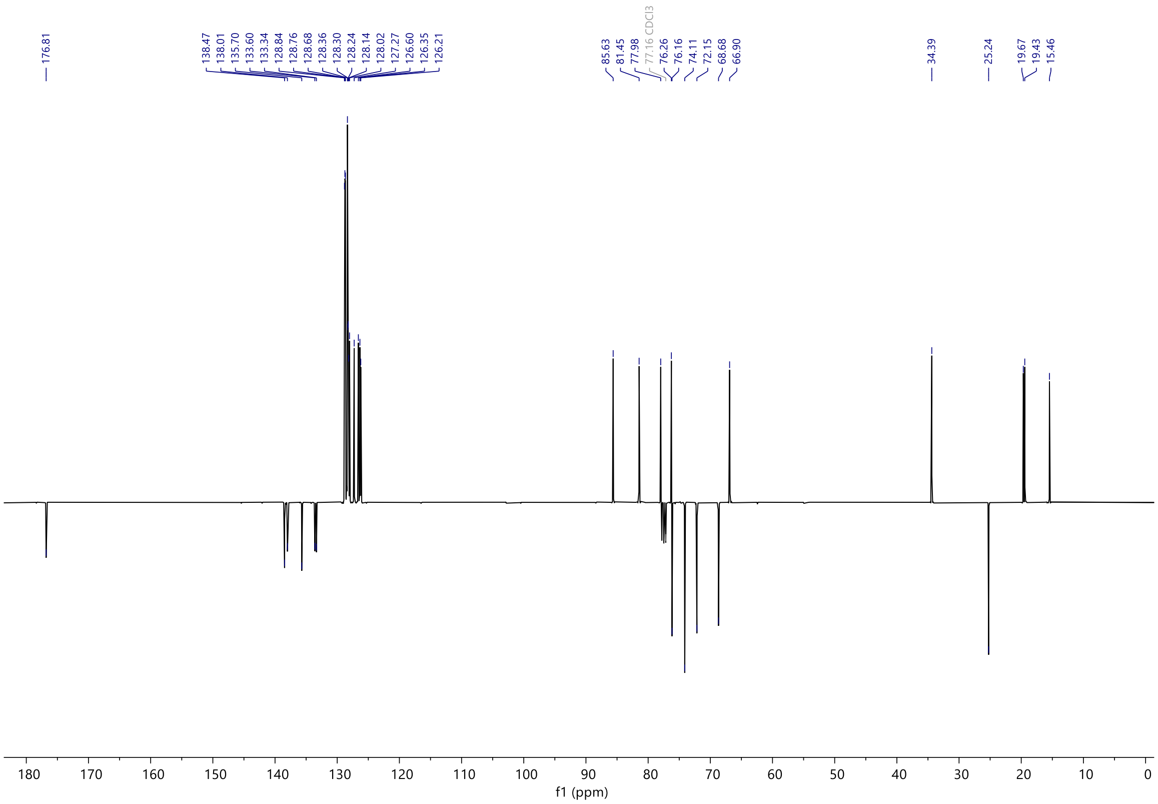

Figure 33. ^{13}C NMR (101 MHz, $CDCl_3$) spectrum of **7d.**

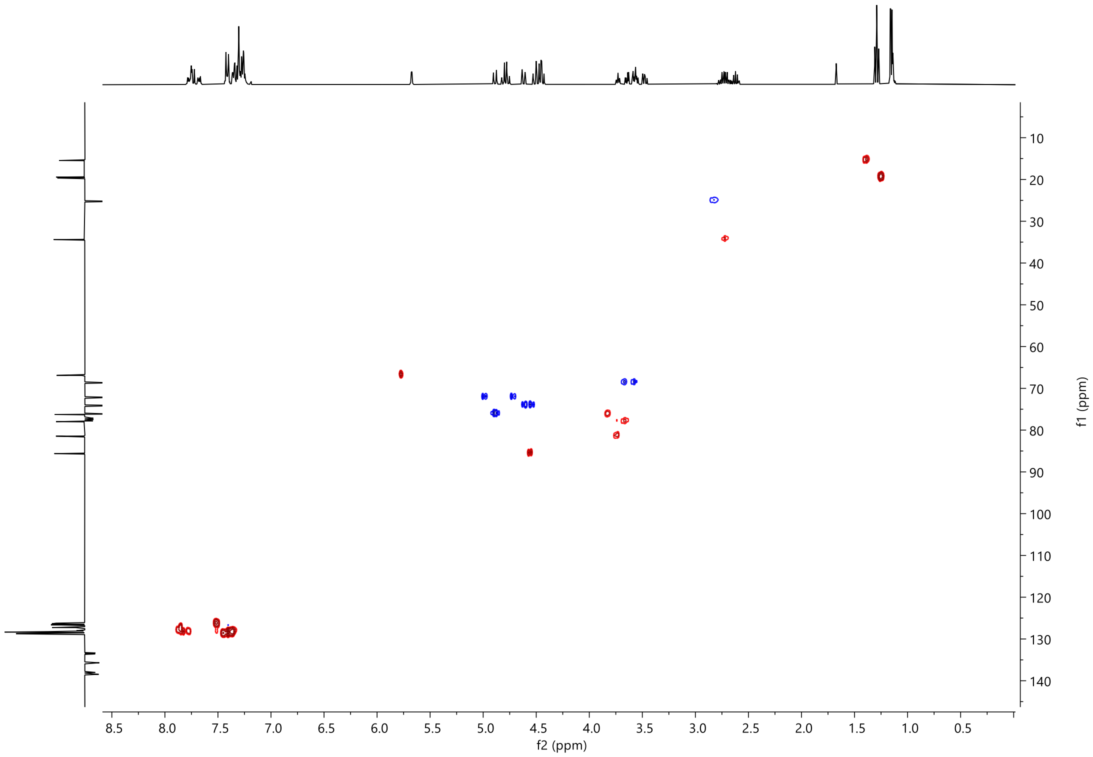

Figure 34. HSQC NMR (400 MHz, $CDCl_3$) spectrum of **7d.**

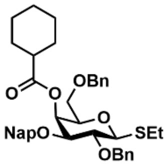

Ethyl 2,6-bis-O-Benzyl-3-O-(2-napththalenylmethyl)-4-O-cyclohexanecarbonyl-1-thio-β-D-galactopyranoside **(7e)**

Yield: 85%. R*f* (Hex/EtOAc 3:1, *v/v*) = 0.47. HR-MS (QTOF) m/z = 677.2932, calcd. for $C_{40}H_{46}O_6SNa$: 677.2913. ^1H NMR (400 MHz, $CDCl_3$) δ 7.70 (td, *J* = 10.3, 6.2 Hz, 3H), 7.64 – 7.59 (m, 1H), 7.39 – 7.33 (m, 3H), 7.30 – 7.20 (m, 10H), 5.61 (d, *J* = 3.3 Hz, 1H), 4.83 (d, *J* = 11.3 Hz, 1H), 4.73 (q, *J* = 10.3 Hz, 2H), 4.56 (d, *J* = 11.3 Hz, 1H), 4.45 (d, *J* = 11.7 Hz, 1H), 4.43 – 4.36 (m, 2H), 3.66 (t, *J* = 6.4 Hz, 1H), 3.58 (dd, *J* = 9.1, 3.2 Hz, 1H), 3.50 (td, *J* = 9.4, 5.9 Hz, 2H), 3.41 (dd, *J* = 9.5, 6.8 Hz, 1H), 2.67 (dtt, *J* = 20.0, 12.6, 7.5 Hz, 2H), 2.31 (tt, *J* = 11.2, 3.5 Hz, 1H), 1.81 (s, 2H), 1.64 (ddt, *J* = 13.9, 6.5, 3.2 Hz, 2H), 1.57 – 1.51 (m, 1H), 1.42 – 1.32 (m, 2H), 1.24 (d, *J* = 7.4 Hz, 3H), 1.19 – 1.09 (m, 3H). ^{13}C NMR (101 MHz, $CDCl_3$) δ 175.38, 138.27, 137.82, 135.50, 133.38, 133.12, 128.57, 128.52, 128.40, 128.12, 128.05, 127.95, 127.86, 127.77, 127.02, 126.36, 126.07, 125.93, 85.44, 81.24, 77.77, 76.12, 75.88, 73.90, 71.89, 68.53, 66.49, 43.18, 29.33, 29.20, 25.87, 25.49, 25.06, 15.20.

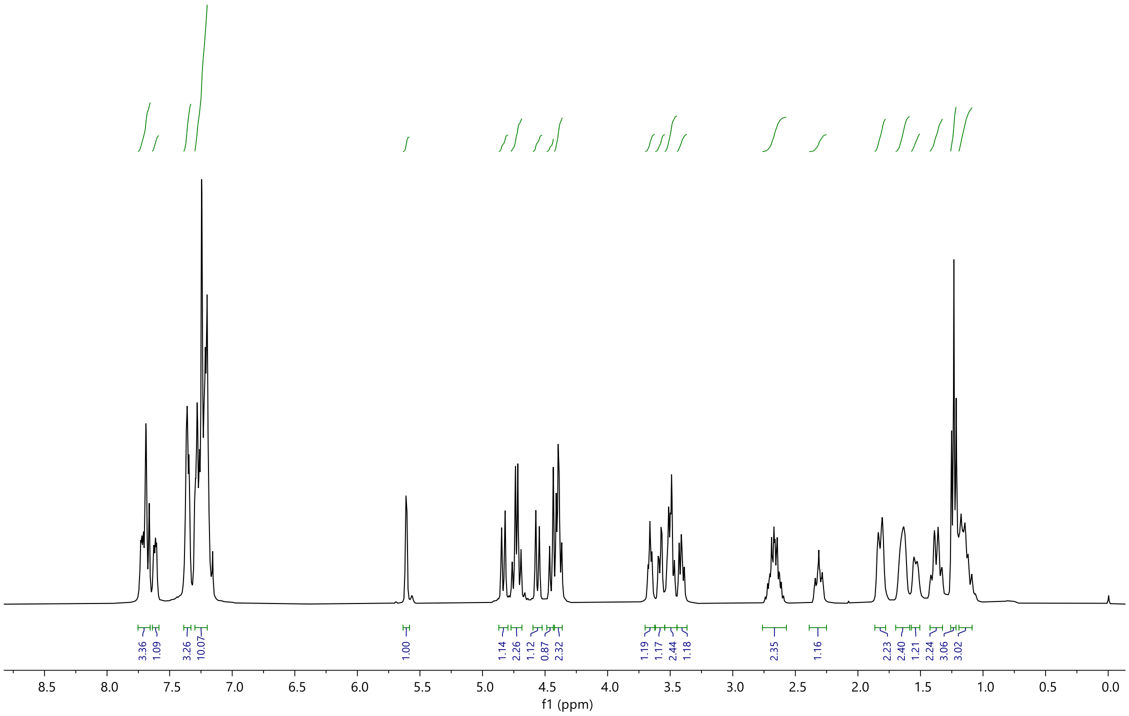

Figure 35. ^1H NMR (400 MHz, $CDCl_3$) spectrum of **7e.**

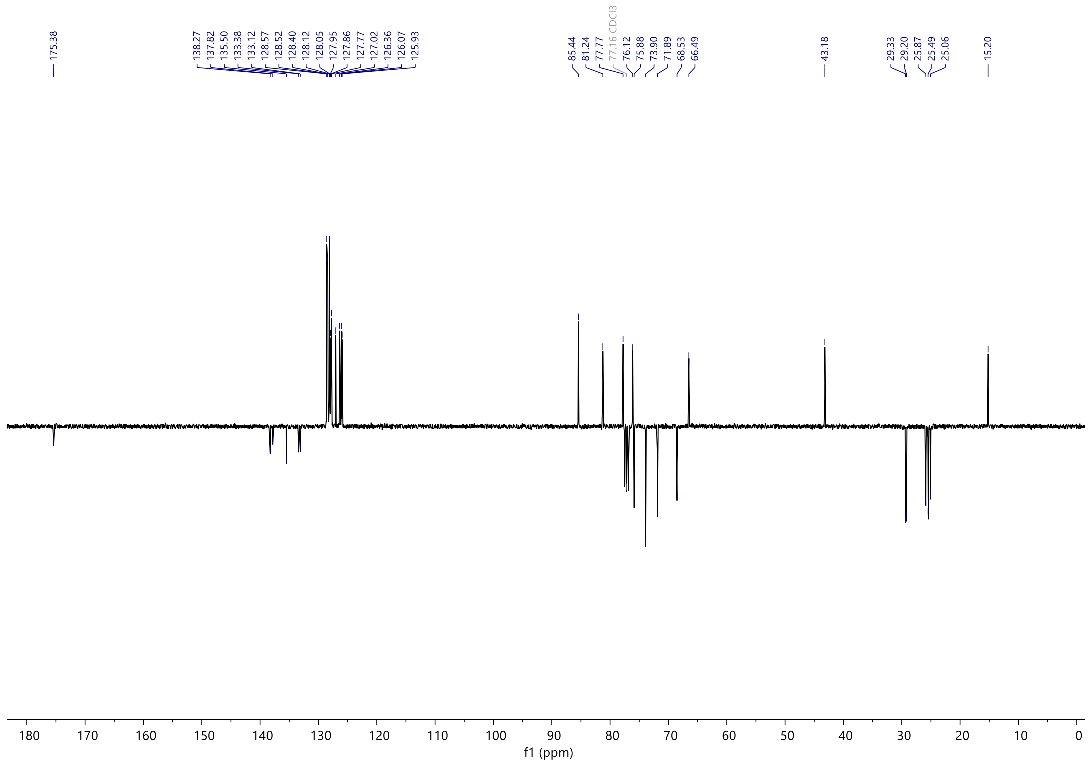

Figure 26. ^{13}C NMR (101 MHz, $CDCl_3$) spectrum of **7e.**

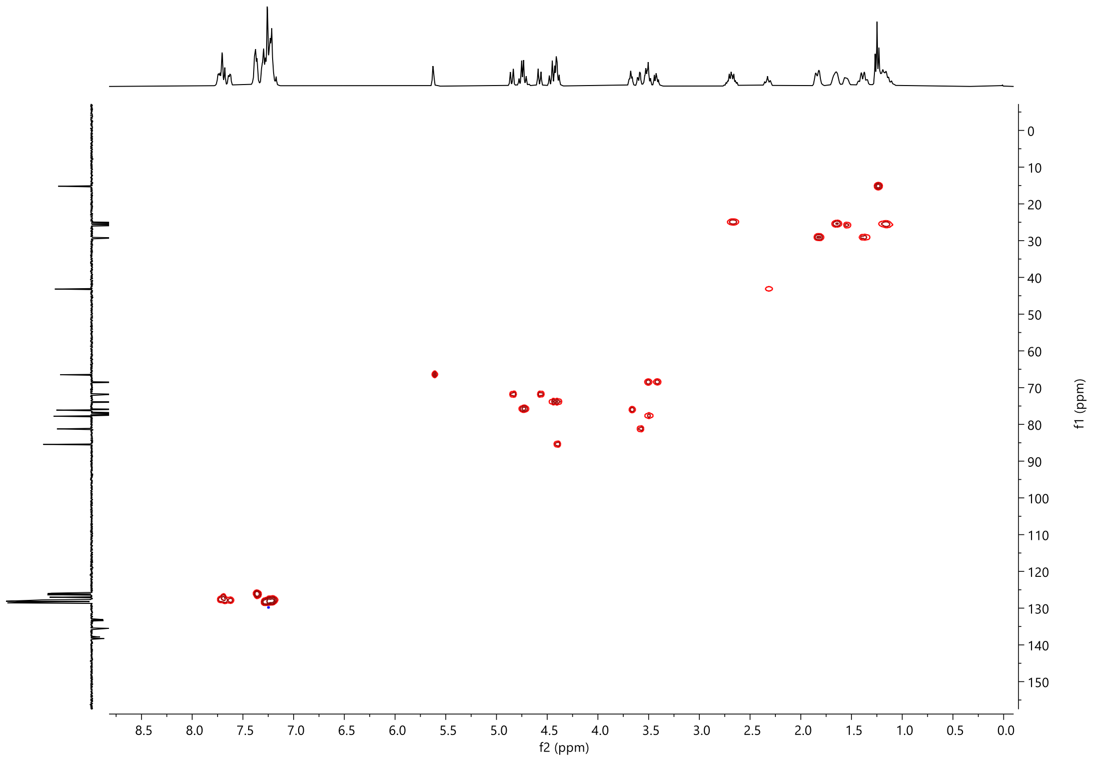

Figure 27. HSQC NMR (400 MHz, $CDCl_3$) spectrum of **7e.**

General procedure for C3-ONap deprotection

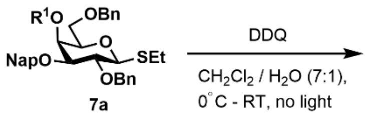

Compound	R^1
**7c**	*n*PrCO
**7d**	*i*BuCO
**7e**	CyCO

Compound	R^1
**7f**	*n*PrCO
**7g**	*i*BuCO
**7h**	CyCO

Each compound (**7c**, **7d**, **7e**; 2.20 mmol) was dissolved in CH_2Cl_2/H_2O (7:1, 28 mL) and 2,3-dichloro-5,6-dicyano-1,4-benzoquinone (2.40 mmol, 1.1 equiv) was added at 0 °C. Then, the suspension was stirred at room temperature for 2 h protected from light. After completion, the crude was diluted with CH_2Cl_2 (80 mL) and the organic phase was washed with sat aq sol $NaHCO_3$ (40 mL) and brine (40 mL). Then, the crude solution was dried over Na_2SO_4, concentrated under reduced pressure and purified by column chromatography Hex/EtOAc (1:1, *v/v)* to afford the corresponding compound (**7f, 7g, 7h**).

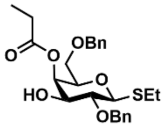

Ethyl 2,6-bis-O-Benzyl-4-O-propanoate-1-thio-β-D-galactopyranoside **(7f)**

Yield: 81%. R*f* (Hex/EtOAc 3:1, *v/v*) = 0.32. HR-MS (QTOF) m/z =483.1835 calcd for $C_{25}H_{32}O_6SNa$: 483.1817. ^{1}H NMR (400 MHz, $CDCl_3$) δ 7.38 – 7.34 (m, 2H), 7.33 – 7.29 (m, 2H), 7.26 (s, 4H), 7.24 (q, *J* = 3.2 Hz, 2H), 5.36 (dd, *J* = 3.4, 1.1 Hz, 1H), 4.89 (d, *J* = 10.7 Hz, 1H), 4.79 (d, *J* = 2.2 Hz, 2H), 4.64 (d, *J* = 10.7 Hz, 1H), 4.50 (d, *J* = 11.8 Hz, 1H), 4.46 – 4.38 (m, 2H), 3.75 (ddd, *J* = 9.4, 4.7, 2.2 Hz, 1H), 3.52 (dd, *J* = 9.6, 6.1 Hz, 1H), 3.46 – 3.41 (m, 2H), 2.74 (p, *J* = 7.4 Hz, 2H), 2.42 – 2.20 (m, 2H), 1.29 (t, *J* = 7.5 Hz, 3H), 1.09 (t, *J* = 7.6 Hz, 3H). ^{13}C NMR (101 MHz, $CDCl_3$) δ 174.81, 137.99, 137.77, 128.65, 128.53, 128.48, 128.18, 128.00, 127.93, 85.27, 78.85, 76.12, 75.60, 73.93, 73.72, 70.11, 68.40, 27.60, 25.34, 15.16, 9.34.

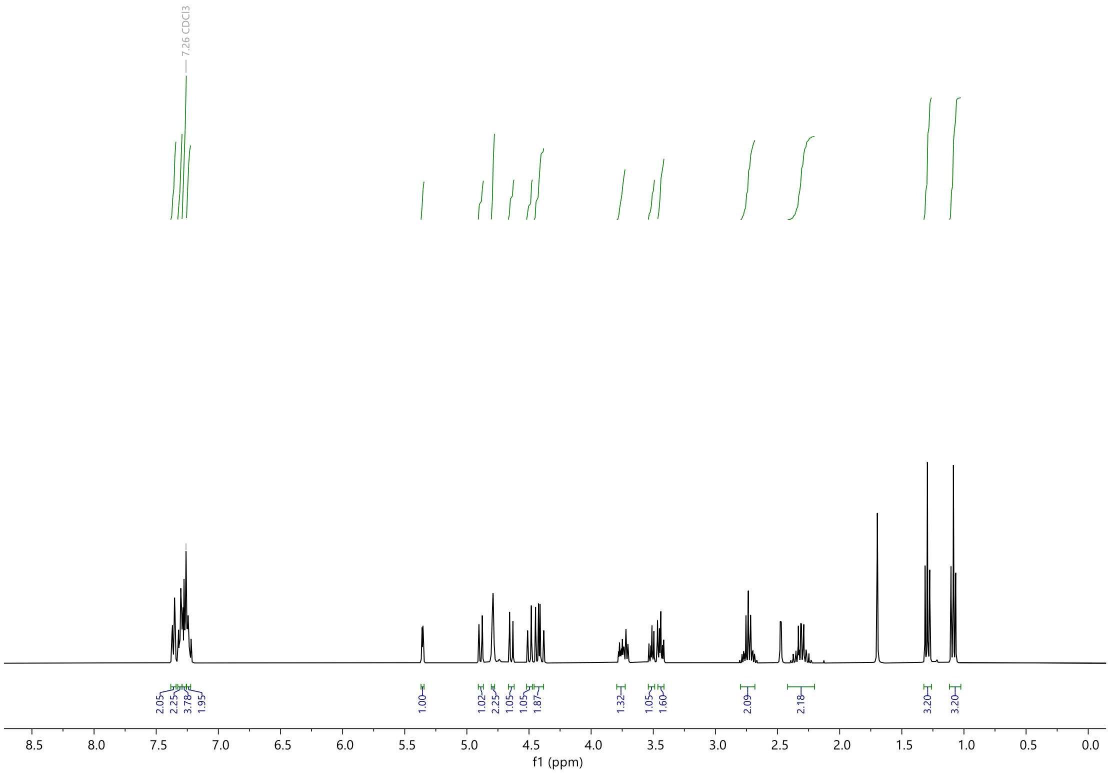

Figure 28. ^{1}H NMR (400 MHz, $CDCl_3$/MeOD) spectrum of **7f**.

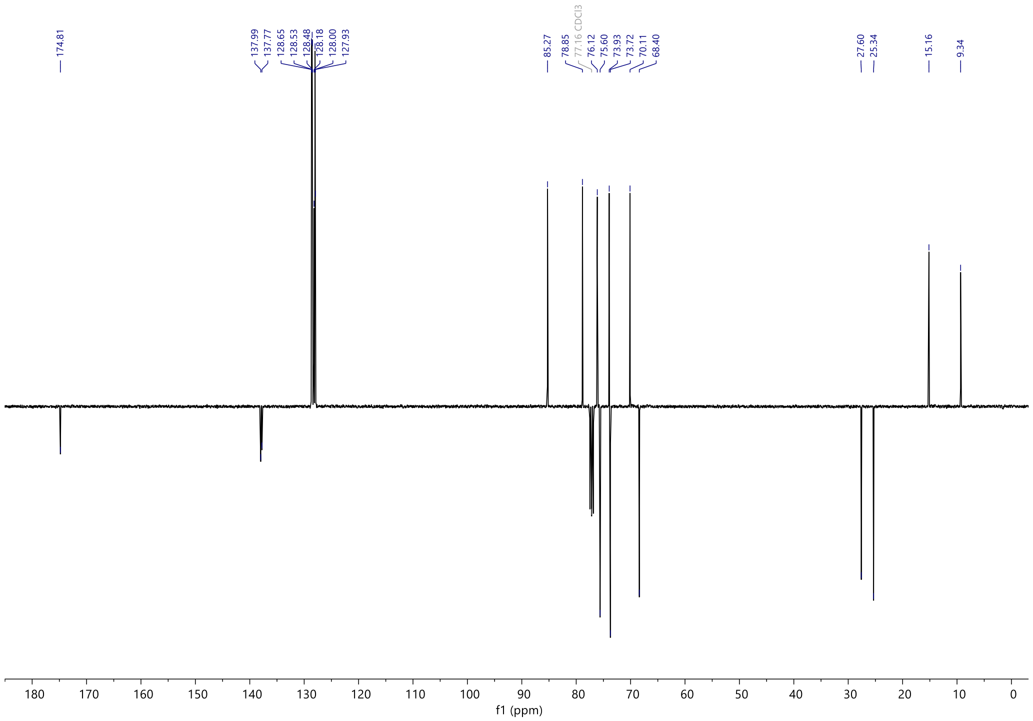

Figure 29. ^{13}C NMR (101 MHz, $CDCl_3$/MeOD) spectrum of **7f**.

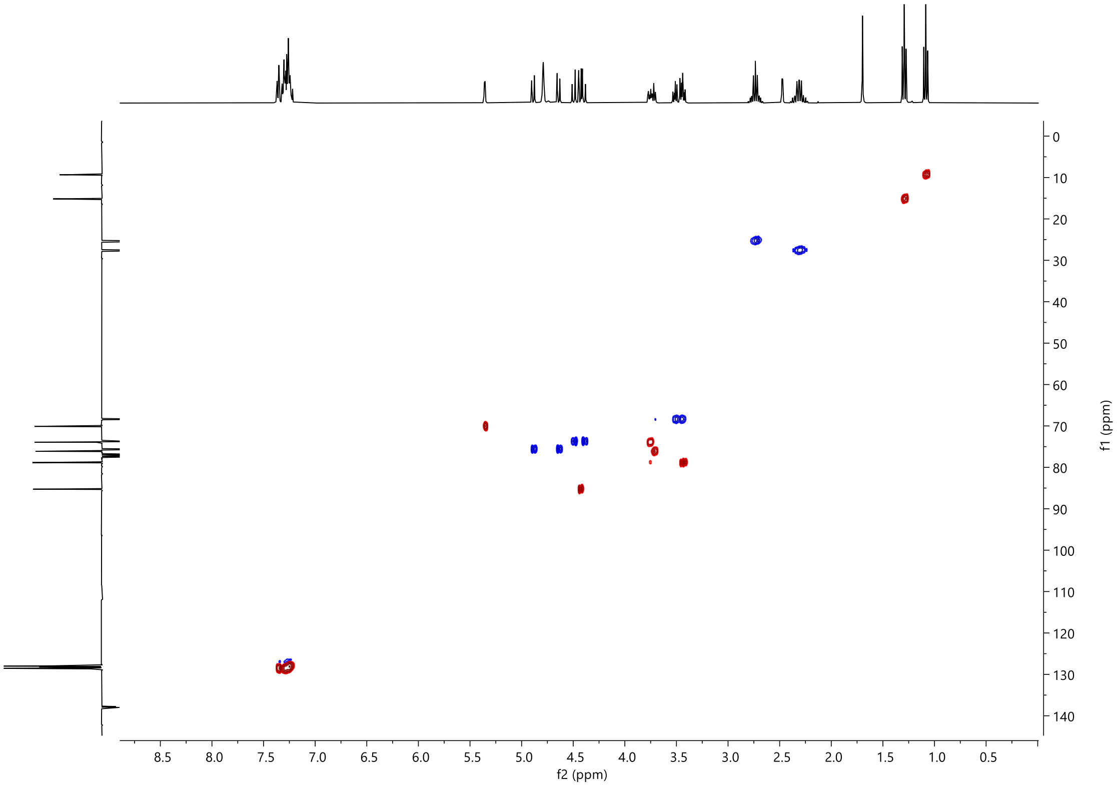

Figure 30. HSQC NMR (400 MHz, $CDCl_3$) spectrum of **7f.**

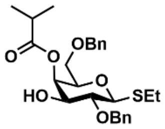

Ethyl 2,6-bis-O-Benzyl-4-O-(2-methylpropanoate)-1-thio-β-D-galactopyranoside **(7g)**

Yield: 82%. R*f* (Hex/EtOAc 3:1, *v/v*) = 0.33. HR-MS (QTOF) m/z =497.1985 calcd for $C_{26}H_{34}O_6SNa$: 497.1974. ^{1}H NMR (400 MHz, $CDCl_3$) δ 7.37 – 7.34 (m, 2H), 7.33 – 7.25 (m, 6H), 7.26 – 7.22 (m, 2H), 5.35 (dd, *J* = 3.5, 1.1 Hz, 1H), 4.89 (d, *J* = 10.8 Hz, 1H), 4.65 (d, *J* = 10.9 Hz, 1H), 4.48 (d, *J* = 11.7 Hz, 1H), 4.45 – 4.38 (m, 2H), 3.77 – 3.70 (m, 2H), 3.52 (dd, *J* = 9.7, 6.2 Hz, 1H), 3.47 – 3.39 (m, 2H), 2.80 – 2.67 (m, 2H), 2.55 (h, *J* = 7.0 Hz, 1H), 1.30 (t, *J* = 7.4 Hz, 3H), 1.12 (d, *J* = 0.7 Hz, 3H), 1.10 (d, *J* = 0.6 Hz, 3H). ^{13}C NMR (101 MHz, $CDCl_3$) δ 177.61, 138.26, 129.02, 128.90, 128.86, 128.55, 128.27, 128.24, 85.53, 78.84, 76.51, 75.79, 74.29, 74.06, 70.19, 68.81, 34.50, 25.56, 19.66, 19.33, 15.47.

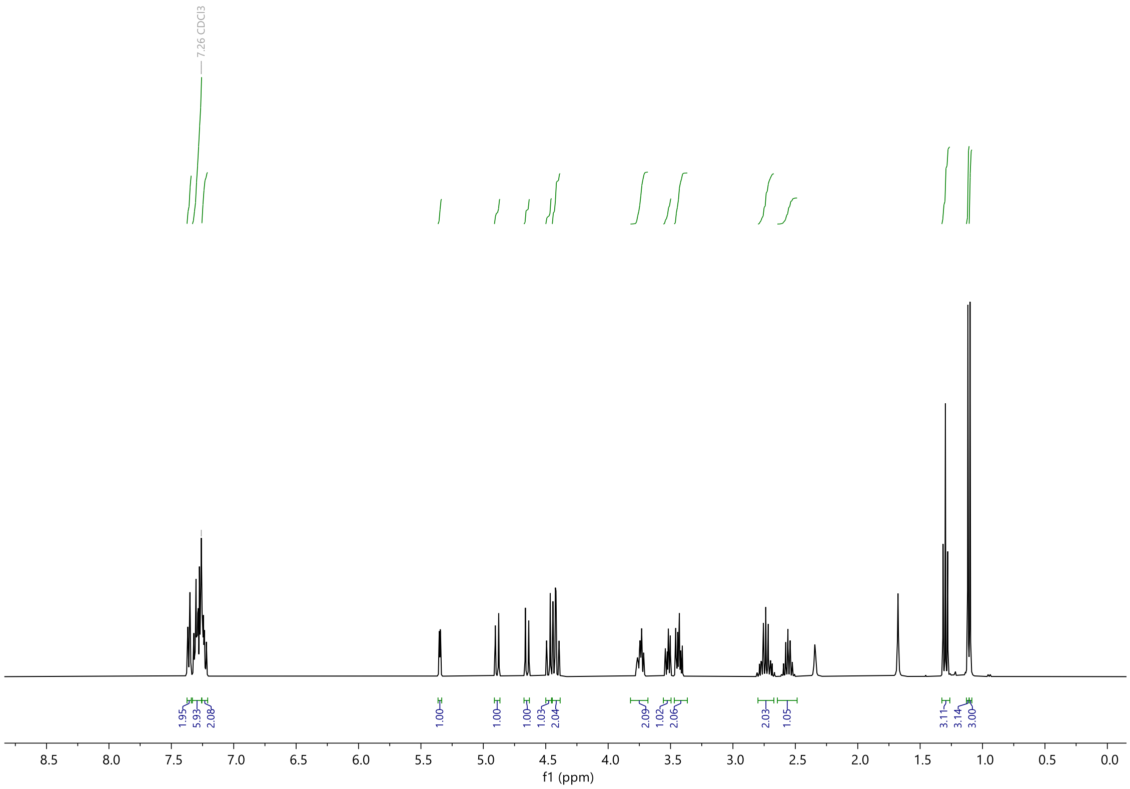

Figure 31. ^{1}H NMR (400 MHz, $CDCl_3$/MeOD) spectrum of **7g**.

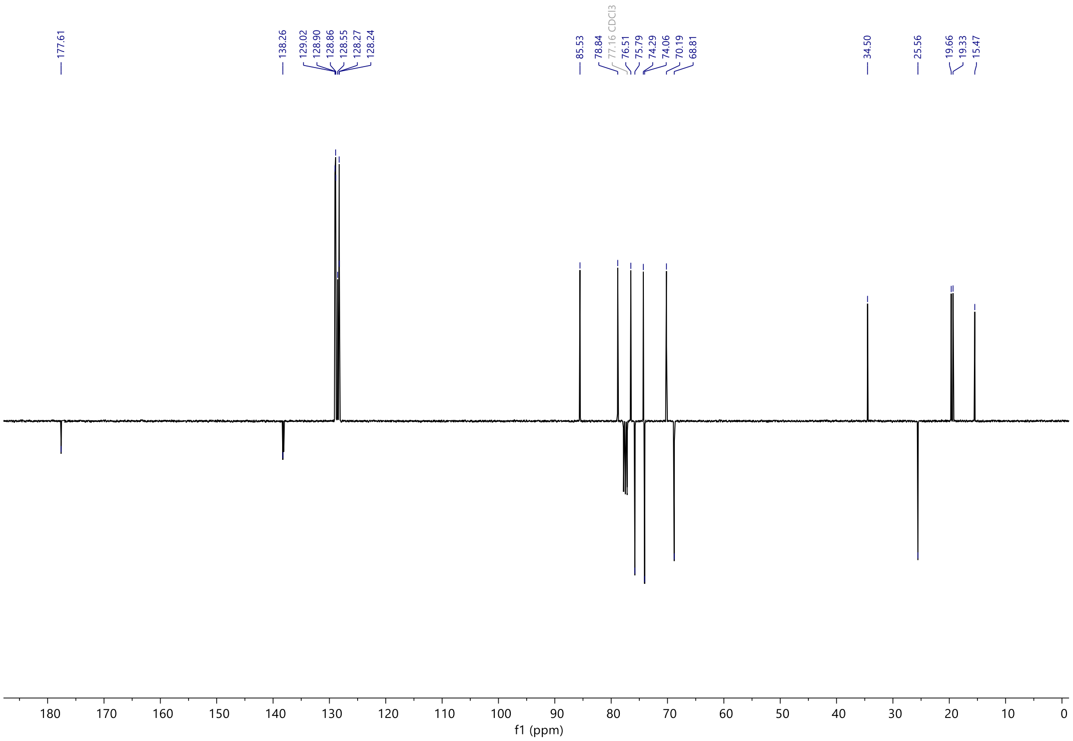

Figure 32. ^{13}C NMR (101 MHz, $CDCl_3$/MeOD) spectrum of **7g**.

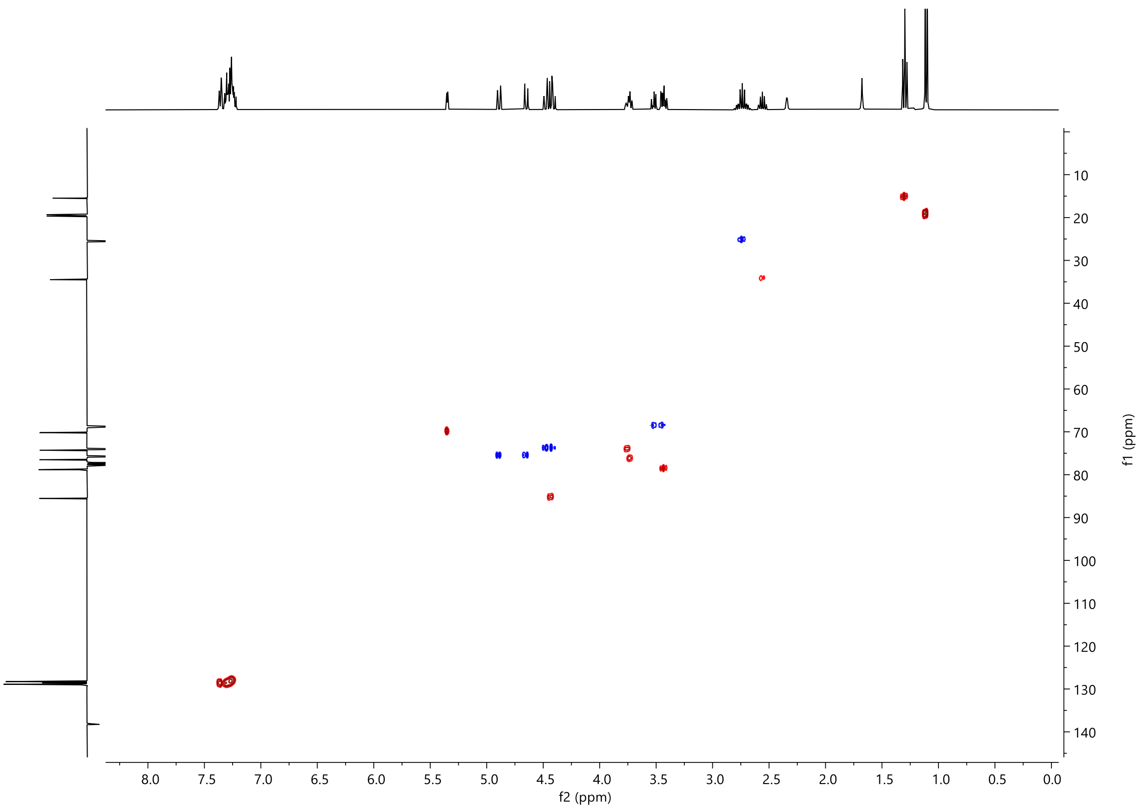

Figure 33. HSQC NMR (400 MHz, $CDCl_3$) spectrum of **7g.**

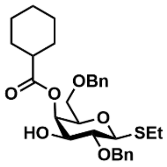

Ethyl 2,6-bis-O-Benzyl-4-O-cyclohexanecarbonyl-1-thio-β-D-galactopyranoside **(7h)**

Yield: 85%. R*f* (Hex/EtOAc 3:1, *v/v*) = 0.35. HR-MS (QTOF) m/z =537.2292 calcd for $C_{29}H_{38}O_6SNa$: 537.2287. ^{1}H NMR (400 MHz, $CDCl_3$) δ 7.38 – 7.34 (m, 2H), 7.33 – 7.30 (m, 2H), 7.30 – 7.26 (m, 4H), 7.24 (d, *J* = 2.6 Hz, 2H), 5.34 (d, *J* = 3.4 Hz, 1H), 4.89 (d, *J* = 10.9 Hz, 1H), 4.66 (d, *J* = 10.9 Hz, 1H), 4.50 – 4.43 (m, 2H), 4.42 – 4.38 (m, 1H), 3.78 – 3.70 (m, 2H), 3.52 (dd, *J* = 9.7, 6.2 Hz, 1H), 3.47 – 3.38 (m, 2H), 2.74 (p, *J* = 7.3 Hz, 2H), 2.30 (tt, *J* = 11.2, 3.6 Hz, 1H), 1.81 (dd, *J* = 11.5, 6.4 Hz, 2H), 1.69 (s, 2H), 1.65 – 1.55 (m, 1H), 1.40 – 1.33 (m, 2H), 1.30 (t, *J* = 7.4 Hz, 4H), 1.27 – 1.14 (m, 3H). ^{13}C NMR (101 MHz, $CDCl_3$) δ 176.54, 138.24, 138.10, 128.98, 128.93, 128.84, 128.52, 128.30, 128.22, 85.56, 78.77, 76.50, 75.73, 74.29, 74.06, 70.00, 68.81, 43.53, 29.61, 29.30, 26.10, 25.84, 25.72, 25.65, 15.47.

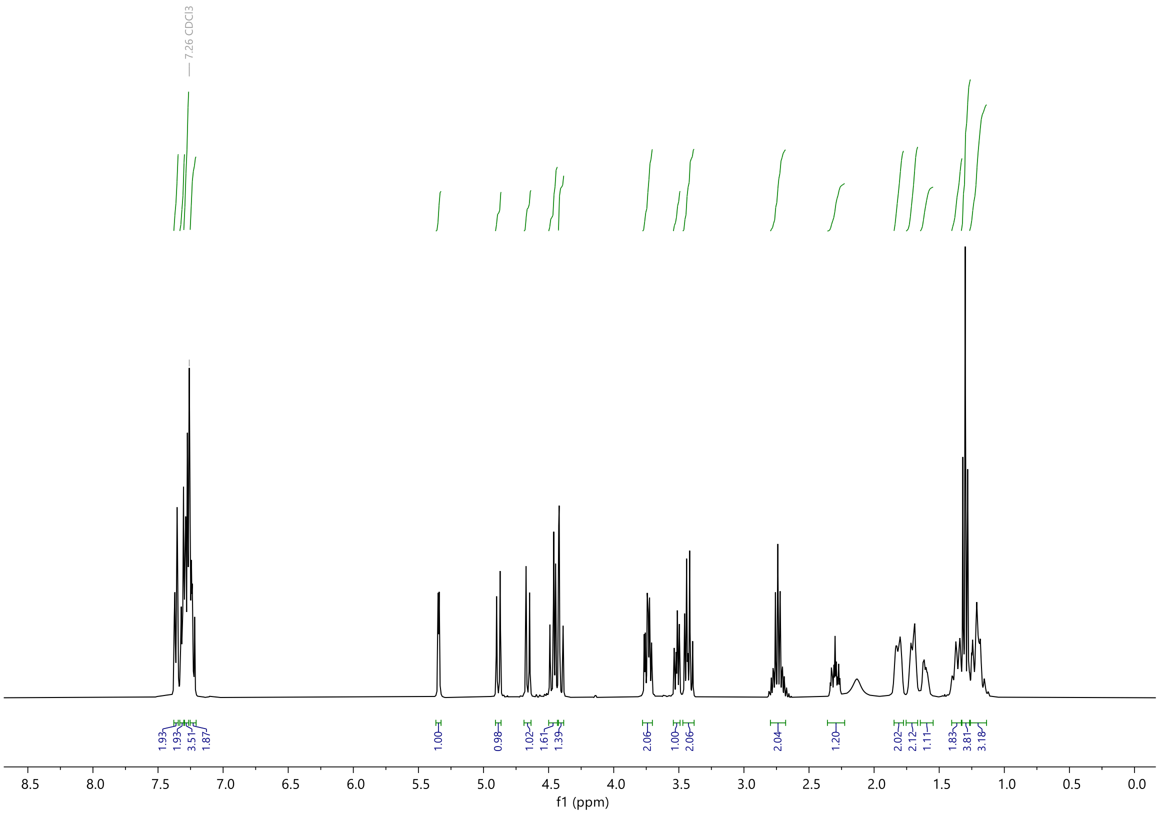

Figure 34. ^{1}H NMR (400 MHz, $CDCl_3$/MeOD) spectrum of **7h**.

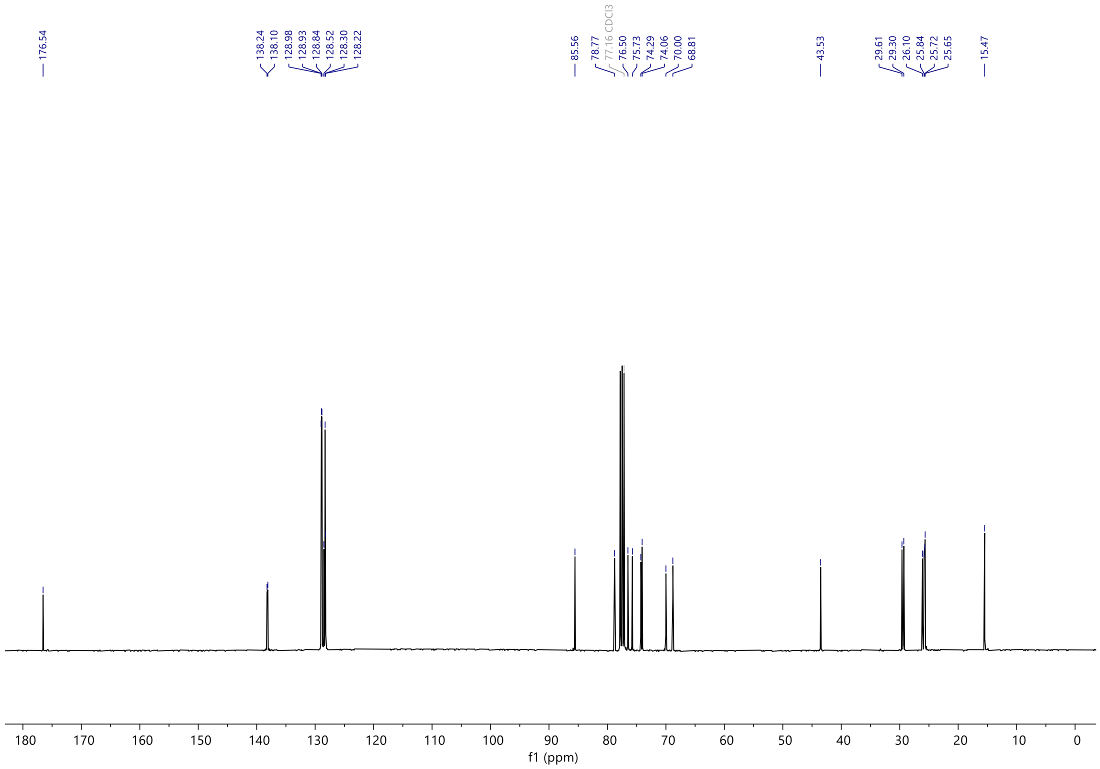

Figure 35. ^{13}C NMR (101 MHz, $CDCl_3$/MeOD) spectrum of **7h**.

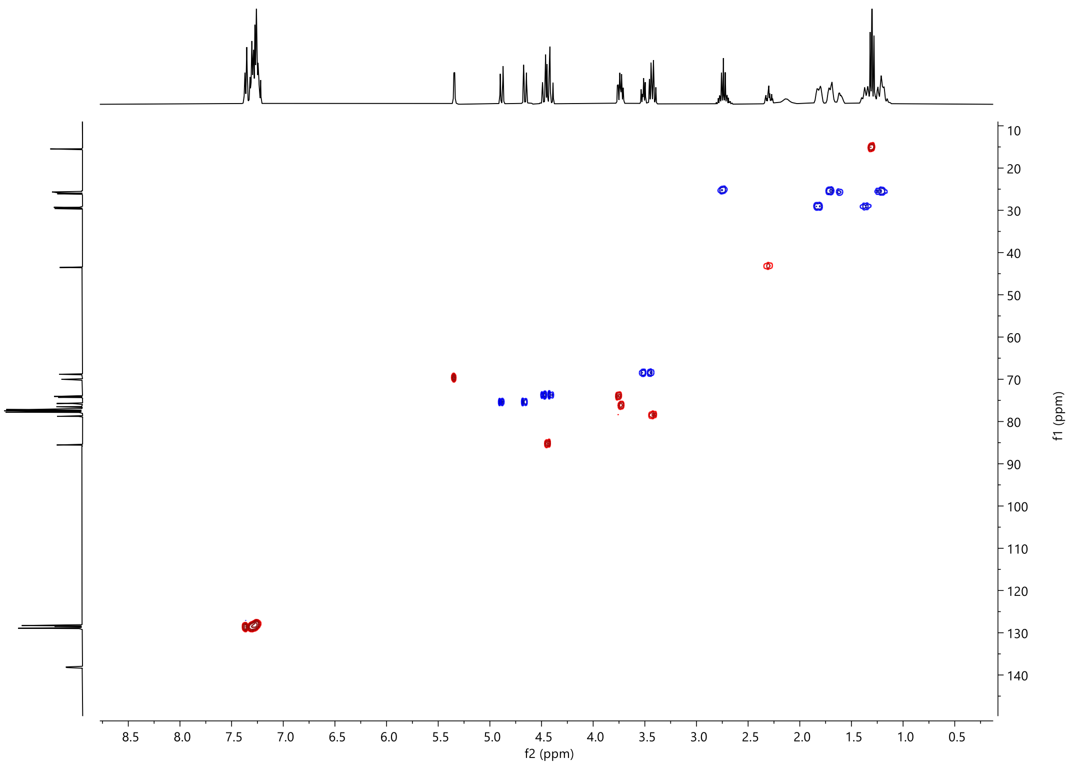

Figure 36. HSQC NMR (400 MHz, $CDCl_3$) spectrum of **7h.**

General procedure for C3-OH Fmoc incorporation

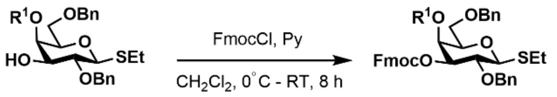

Compound	R^1
**7f**	*n*PrCO
**7g**	*i*BuCO
**7h**	CyCO

Compound	R^1
**7i**	*n*PrCO
**7j**	*i*BuCO
**7k**	CyCO

To a solution of compound (**7f**, **7g** and **7h**; 1.0 mmol) in anhydrous CH_2Cl_2 (25 mL), pyridine (25 mmol, 5.0 equiv) was added and stirred at 0 °C for 10 min. Then, Fmoc chloride (1.5 mmol, 1.5 equiv) was added slowly; the reaction was warmed up to room temperature and stirred for 8 h. After completion, the crude was diluted with CH_2Cl_2 (20 mL). The organic layer was washed with 10% of citric acid (10 mL, *w/v*) and brine (10 mL). Then, the crude solution was dried over Na_2SO_4, concentrated under reduced pressure and purified by column chromatography Hex/EtOAc (10:1, *v/v*) to afford the corresponding product as a white solid**.**

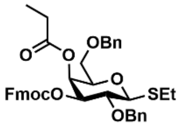

Ethyl 2,6-bis-O-Benzyl-3-O-fluorenylmethoxycarbonyl-4-O-propanoate-1-thio-β-D-galactopyranoside **(7i)**

Yield: 79%. R*f* (Hex/EtOAc 10:1, *v/v*) = 0.41. HR-MS (QTOF) m/z =705.2477 calcd for $C_{40}H_{42}O_8SNa$: 7052498. ^{1}H NMR (400 MHz, $CDCl_3$) δ 7.77 (dq, *J* = 7.6, 1.0 Hz, 2H), 7.61 (tq, *J* = 8.5, 0.9 Hz, 2H), 7.40 (tdd, *J* = 7.6, 2.4, 1.2 Hz, 2H), 7.37 – 7.32 (m, 4H), 7.32 – 7.26 (m, 8fH), 5.64 (dd, *J* = 3.4, 1.1 Hz, 1H), 4.91 – 4.82 (m, 2H), 4.70 (d, *J* = 10.6 Hz, 1H), 4.57 (d, *J* = 2.4 Hz, 1H), 4.53 (dd, *J* = 7.1, 4.2 Hz, 2H), 4.43 (d, *J* = 11.9 Hz, 1H), 4.31 – 4.25 (m, 2H), 3.85 (td, *J* = 6.4, 1.1 Hz, 1H), 3.70 (t, *J* = 9.7 Hz, 1H), 3.57 (dd, *J* = 9.6, 6.1 Hz, 1H), 3.48 (dd, *J* = 9.6, 6.5 Hz, 1H), 2.92 – 2.70 (m, 2H), 2.49 – 2.27 (m, 2H), 1.34 (t, *J* = 7.4 Hz, 3H), 1.14 (t, *J* = 7.6 Hz, 3H). ^{13}C NMR (101 MHz, $CDCl_3$) δ 174.17, 154.59, 144.16, 143.58, 141.68, 138.03, 128.88, 128.78, 128.63, 128.32, 127.58, 125.74, 125.59, 120.45, 85.78, 79.00, 76.49, 74.02, 70.69, 68.36, 68.16, 47.11, 27.85, 25.72, 15.50, 9.72.

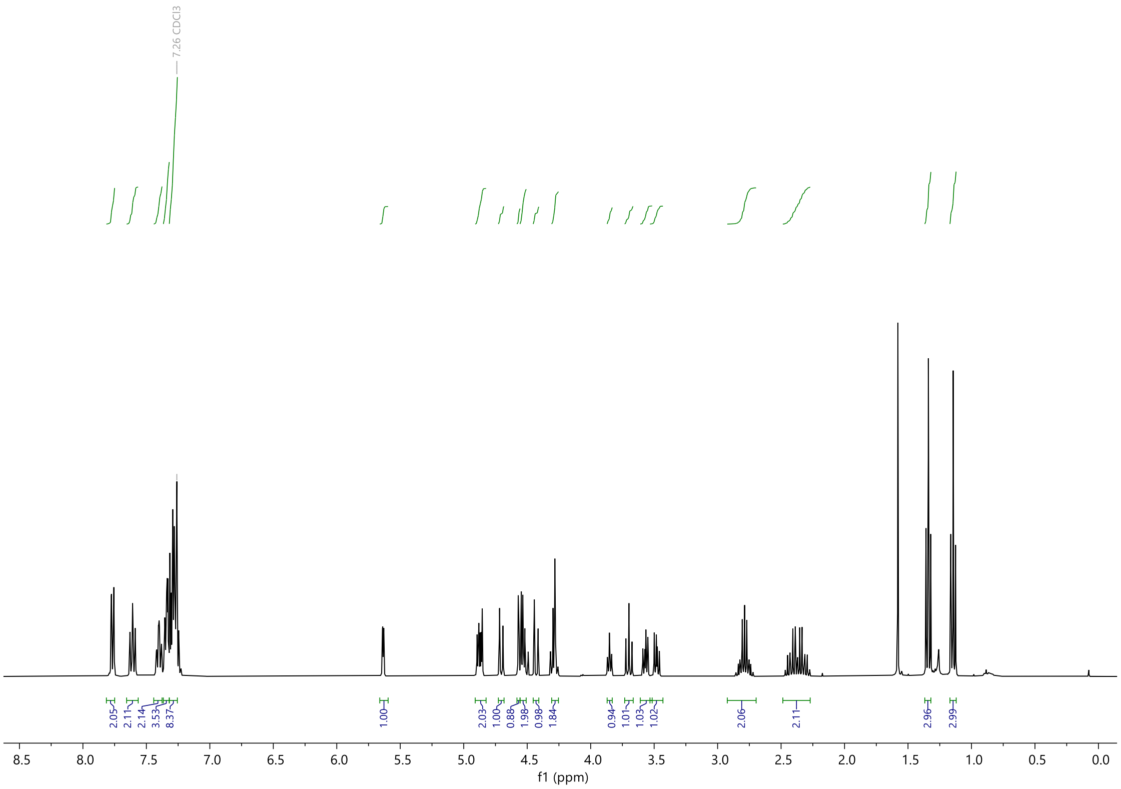

Figure 37. ^{1}H NMR (400 MHz, $CDCl_3$/MeOD) spectrum of **7i**.

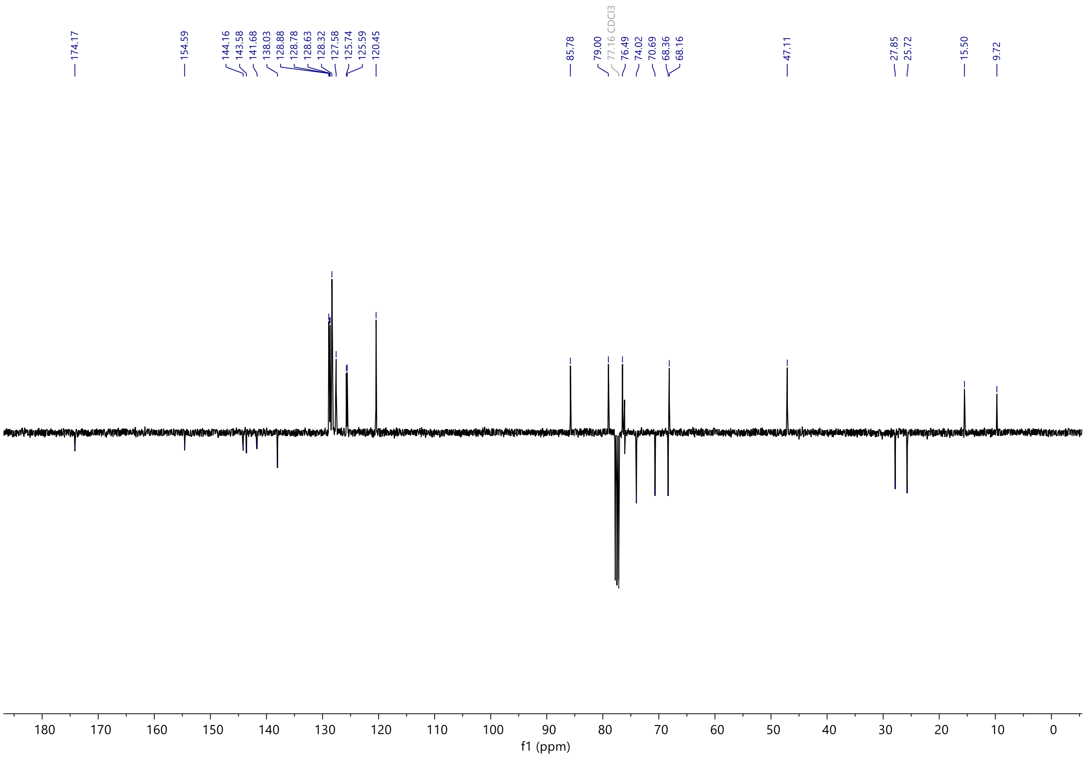

Figure 38. ^{13}C NMR (101 MHz, $CDCl_3$/MeOD) spectrum of **7i**.

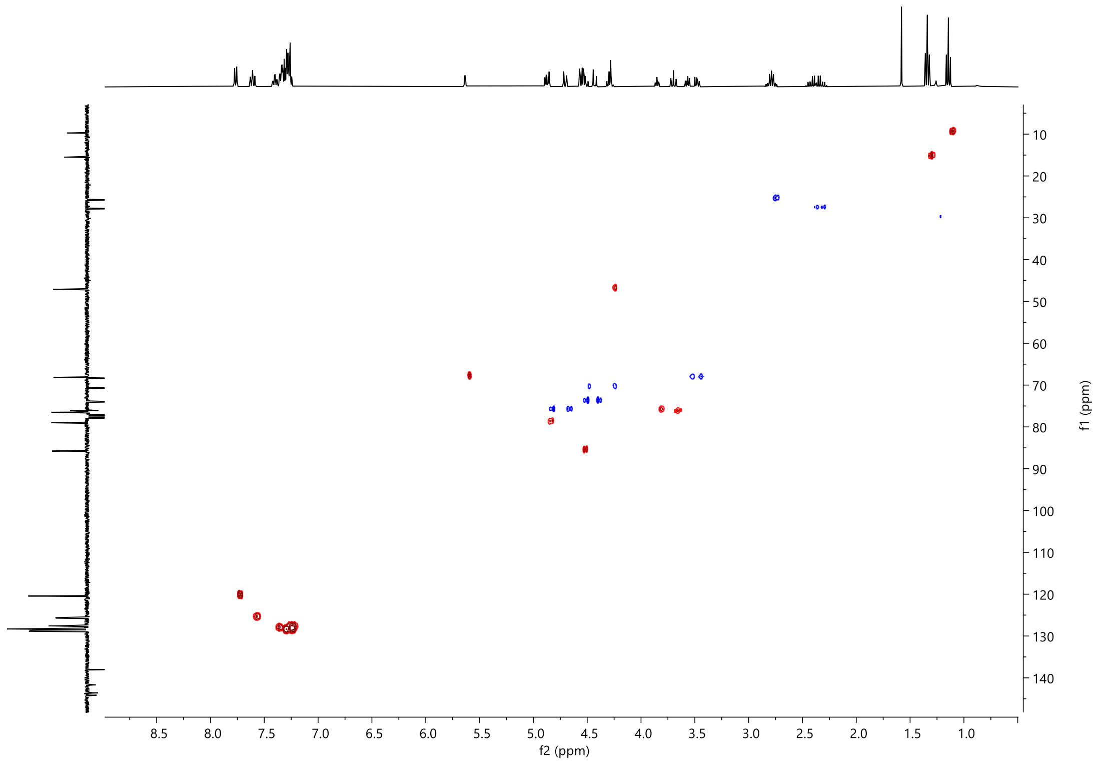

Figure 39. HSQC NMR (400 MHz, $CDCl_3$) spectrum of **7i.**

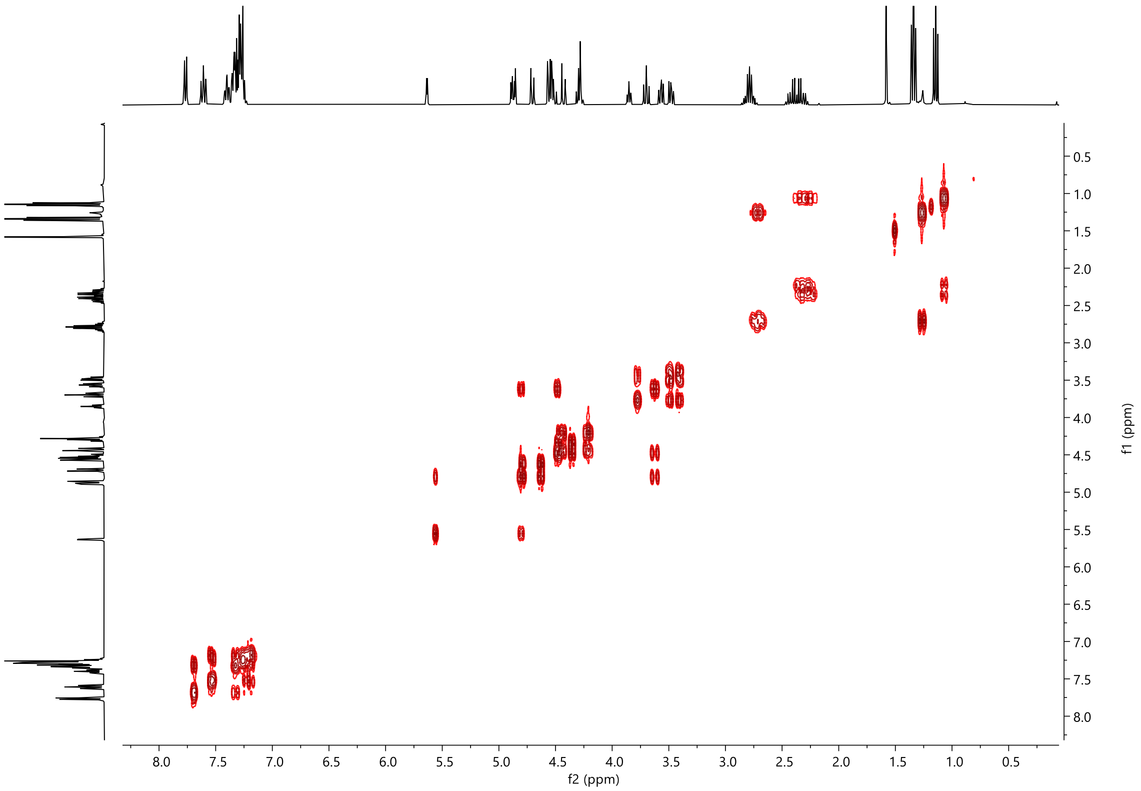

Figure 40. COSY NMR (400 MHz, $CDCl_3$) spectrum of **7i.**

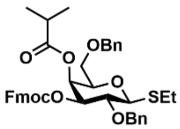

Ethyl 2,6-bis-O-Benzyl-3-O-fluorenylmethoxycarbonyl-4-O-(2-methylpropanoate)-1-thio-β-D-galactopyranoside **(7j)**

Yield: 80%. R*f* (Hex/EtOAc 10:1, *v/v*) = 0.42. HR-MS (QTOF) m/z = 719.2632 calcd for $C_{41}H_{44}O_8SNa$: 719.2655. ^{1}H NMR (400 MHz, $CDCl_3$) δ 7.77 (d, *J* = 7.6 Hz, 2H), 7.61 (t, *J* = 7.7 Hz, 2H), 7.39 (d, *J* = 7.5 Hz, 2H), 7.29 (m, 12H), 5.62 (d, *J* = 3.4 Hz, 1H), 4.91 – 4.83 (m, 2H), 4.70 (d, *J* = 10.6 Hz, 1H), 4.55 (d, *J* = 9.6 Hz, 1H), 4.53 – 4.48 (m, 2H), 4.44 (d, *J* = 11.9 Hz, 1H), 4.32 – 4.25 (m, 2H), 3.86 (t, *J* = 6.3 Hz, 1H), 3.68 (t, *J* = 9.7 Hz, 1H), 3.58 (dd, *J* = 9.7, 6.2 Hz, 1H), 3.47 (dd, *J* = 9.7, 6.4 Hz, 1H), 2.79 (p, *J* = 7.6 Hz, 2H), 2.63 (dq, *J* = 13.9, 7.0 Hz, 1H), 1.34 (t, *J* = 7.4 Hz, 3H), 1.19 (d, *J* = 7.0 Hz, 3H), 1.16 (d, *J* = 7.0 Hz, 3H). ^{13}C NMR (101 MHz, $CDCl_3$) δ 176.70, 154.59, 144.09, 143.62, 141.74, 138.05, 137.91, 128.88, 128.76, 128.28, 127.61, 125.73, 125.61, 120.44, 85.71, 78.95, 76.20, 75.96, 74.05, 70.61, 68.50, 68.01, 47.11, 34.39, 25.62, 19.70, 19.33, 15.49.

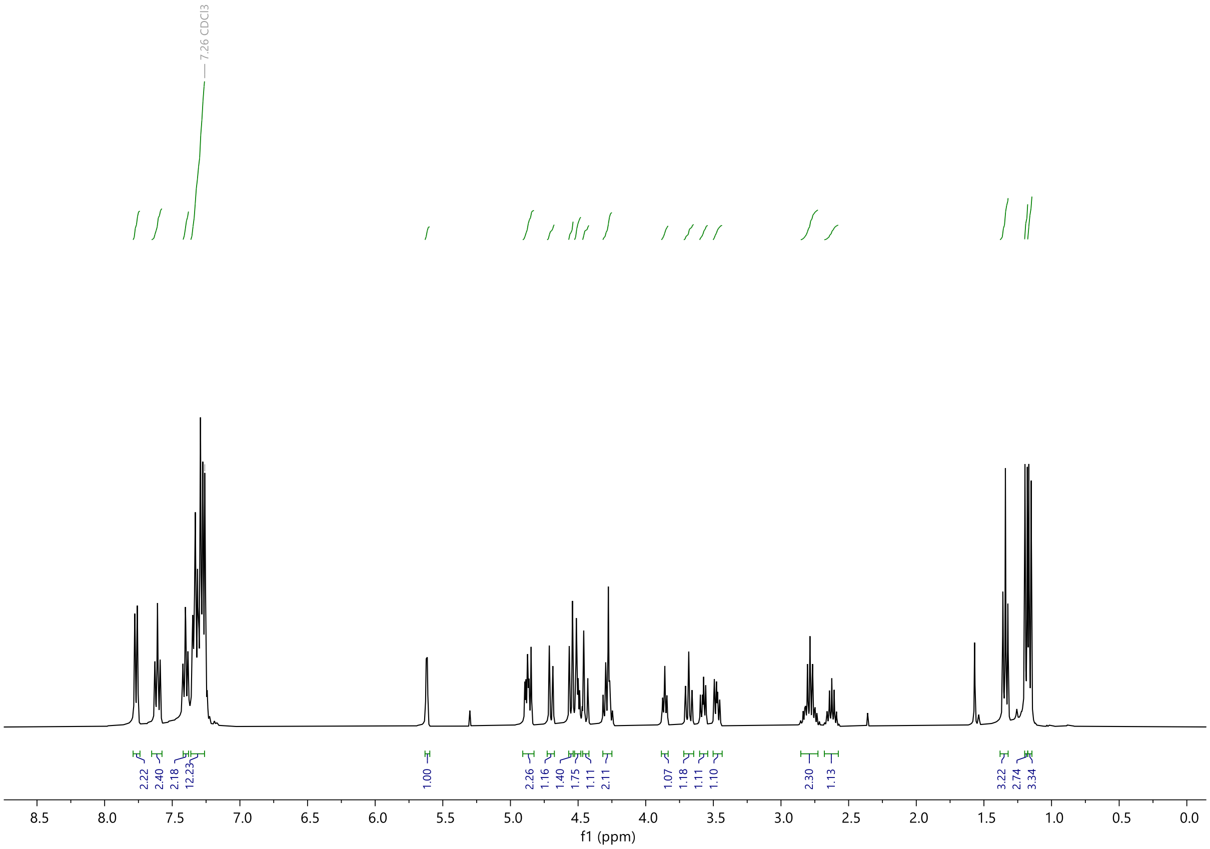

Figure 41. ^{1}H NMR (400 MHz, $CDCl_3$/MeOD) spectrum of **7j**.

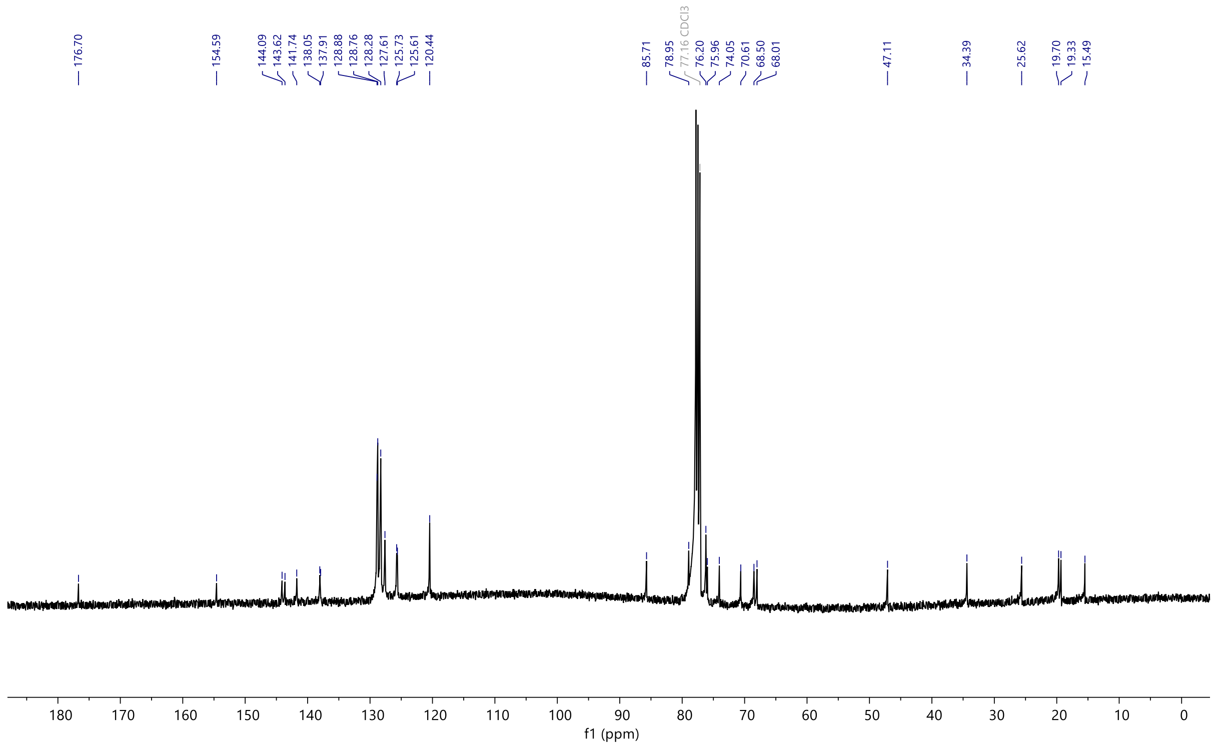

Figure 42. ^{13}C NMR (101 MHz, $CDCl_3$/MeOD) spectrum of **7j**.

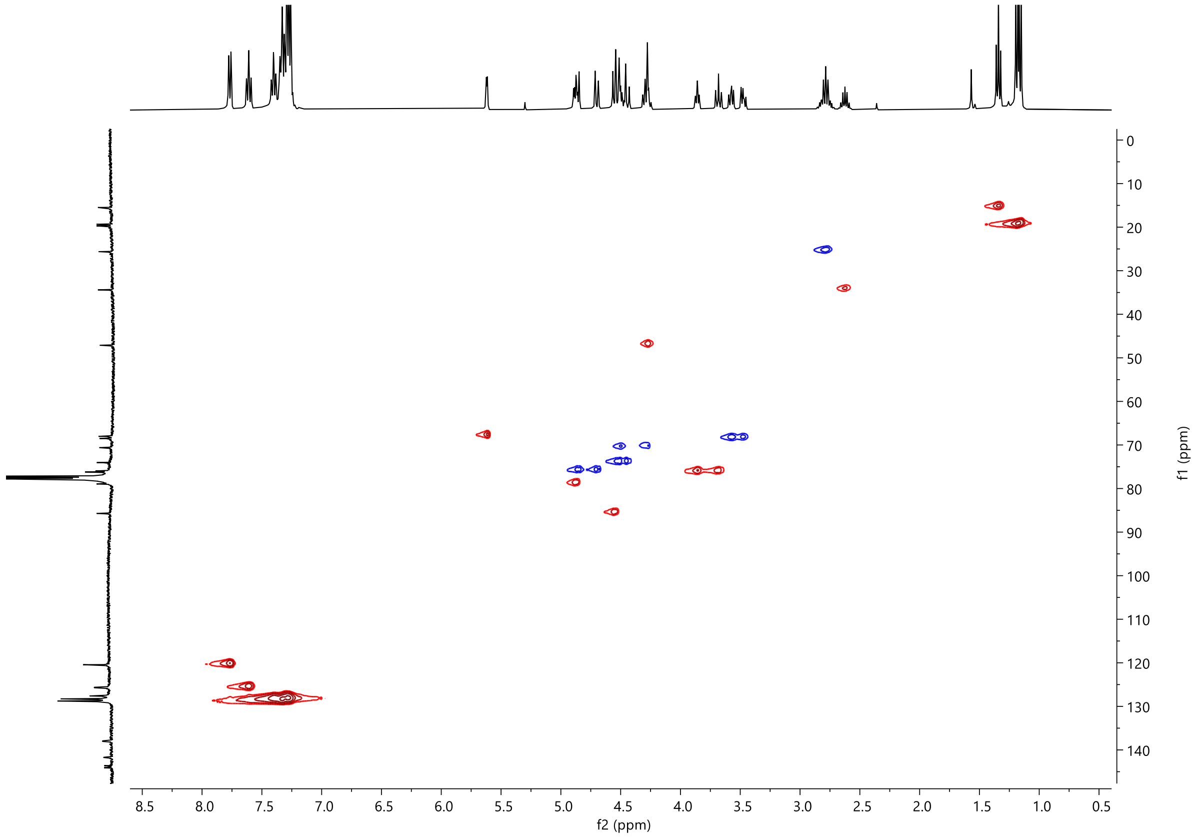

Figure 43. HSQC NMR (400 MHz, $CDCl_3$) spectrum of **7j.**

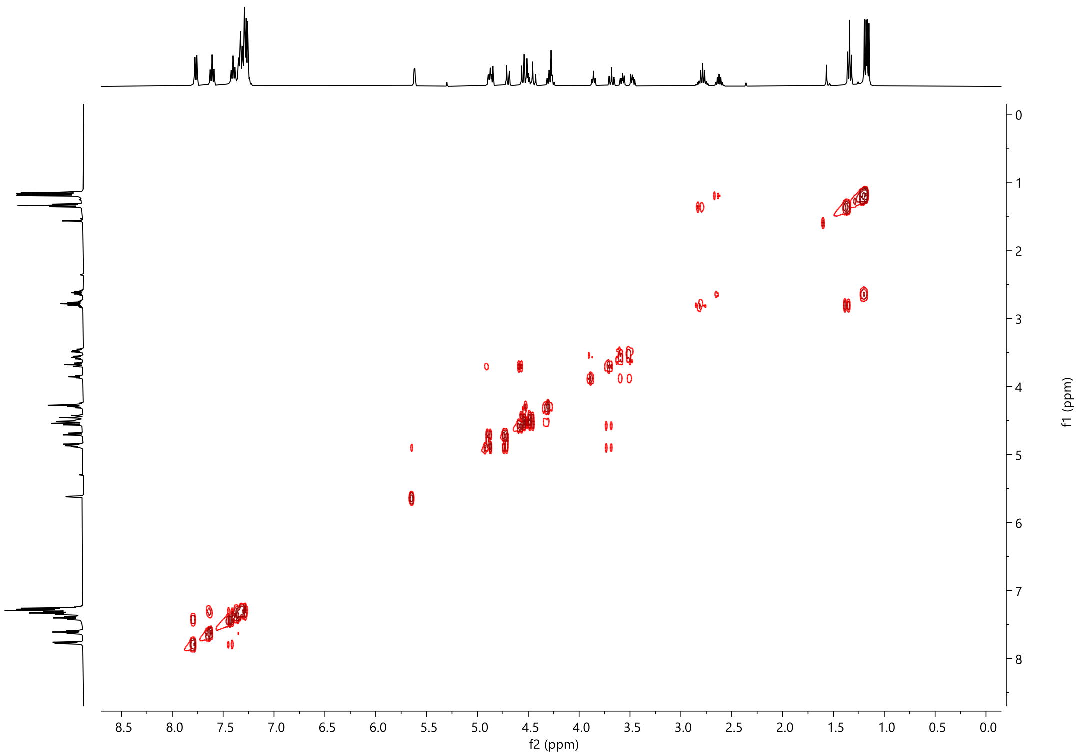

Figure 44. COSY NMR (400 MHz, $CDCl_3$) spectrum of **7j**.

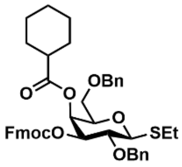

Ethyl 2,6-bis-O-Benzyl-3-O-fluorenylmethoxycarbonyl-4-O-cyclohexanecarbonyl-1-thio-β-D-galactopyranoside **(7k)**

Yield: 82%. R*f* (Hex/EtOAc 10:1, *v/v*) = 0.44. HR-MS (QTOF) m/z = 759.2979 calcd for $C_{44}H_{48}O_8SNa$: 759.2968. ^{1}H NMR (400 MHz, $CDCl_3$) δ 7.73 (d, *J* = 7.6 Hz, 2H), 7.62 – 7.54 (m, 2H), 7.37 (t, *J* = 7.5 Hz, 2H), 7.26 (m, 12H), 5.59 (d, *J* = 3.4 Hz, 1H), 4.84 (dd, *J* = 11.6, 7.4 Hz, 2H), 4.68 (d, *J* = 10.8 Hz, 1H), 4.54 (s, 1H), 4.48 (d, *J* = 4.8 Hz, 1H), 4.39 (s, 1H), 4.30 – 4.21 (m, 2H), 3.82 (t, *J* = 6.3 Hz, 1H), 3.64 (t, *J* = 9.6 Hz, 1H), 3.54 (dd, *J* = 9.7, 6.2 Hz, 1H), 3.44 (dd, *J* = 9.7, 6.3 Hz, 1H), 2.75 (p, *J* = 6.9 Hz, 2H), 2.33 (d, *J* = 3.8 Hz, 1H), 1.82 (d, *J* = 12.3 Hz, 2H), 1.74 (dd, *J* = 12.8, 5.8 Hz, 2H), 1.63 (d, *J* = 9.6 Hz, 1H), 1.39 (t, *J* = 10.1 Hz, 2H), 1.31 (t, *J* = 7.5 Hz, 4H), 1.22 (dd, *J* = 16.5, 7.5 Hz, 3H). ^{13}C NMR (101 MHz, $CDCl_3$) δ 175.51, 154.52, 144.05, 143.58, 141.69, 141.63, 138.00, 137.83, 129.43, 128.82, 128.77, 128.69, 128.63, 128.26, 128.19, 127.54, 127.49, 125.69, 125.56, 120.39, 85.68, 78.87, 76.11, 76.02, 75.81, 73.99, 70.49, 68.45, 67.73, 47.07, 43.34, 29.62, 29.20, 26.10, 25.80, 25.64, 15.44.

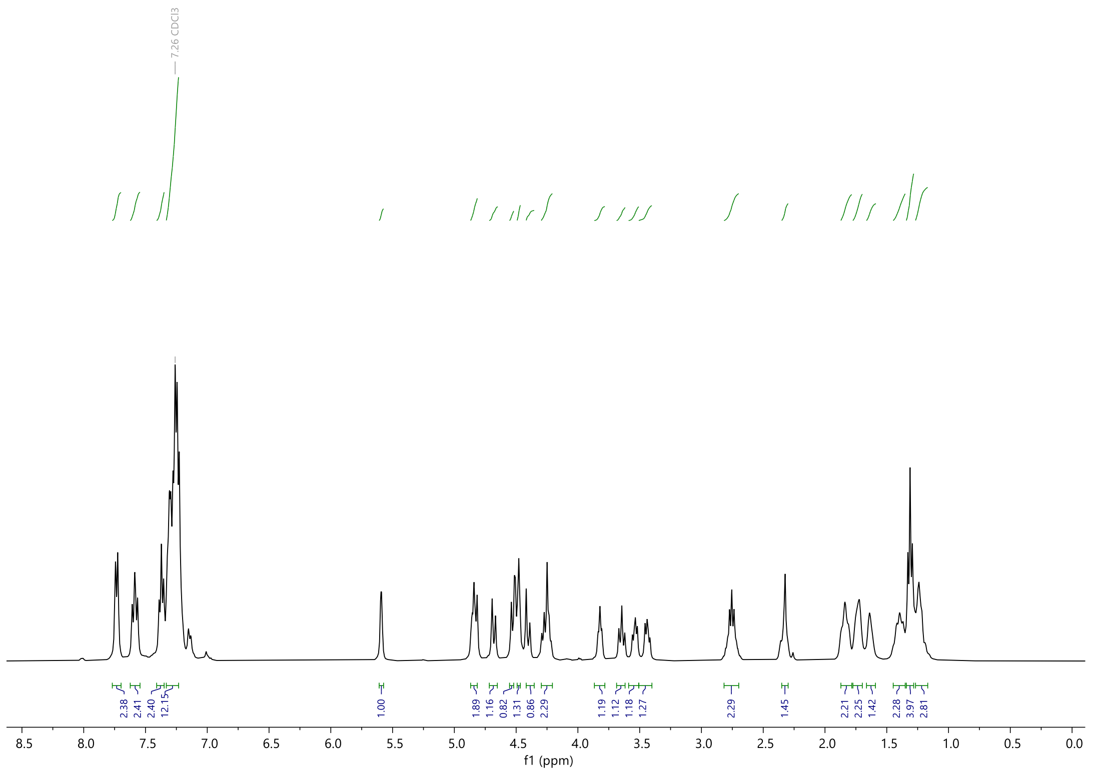

Figure 45. ^{1}H NMR (400 MHz, $CDCl_3$/MeOD) spectrum of **7k**.

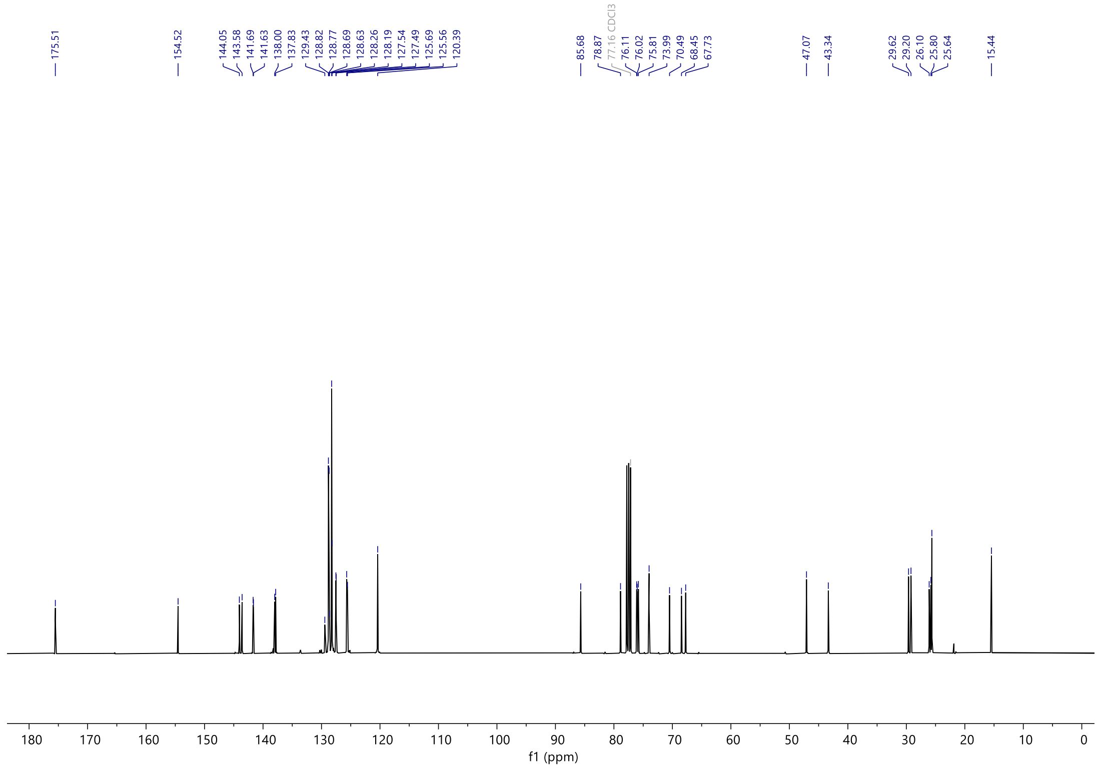

Figure 46. ^{13}C NMR (101 MHz, $CDCl_3$/MeOD) spectrum of **7k**.

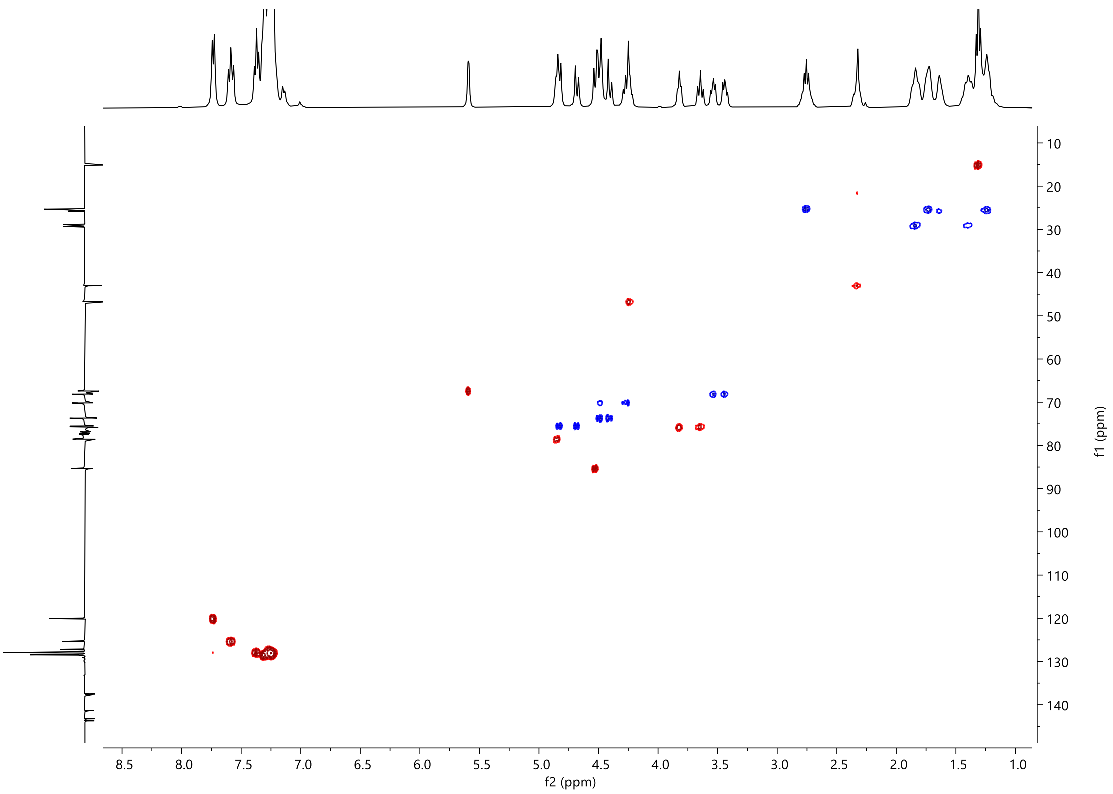

Figure 47. HSQC NMR (400 MHz, $CDCl_3$) spectrum of **7k.**

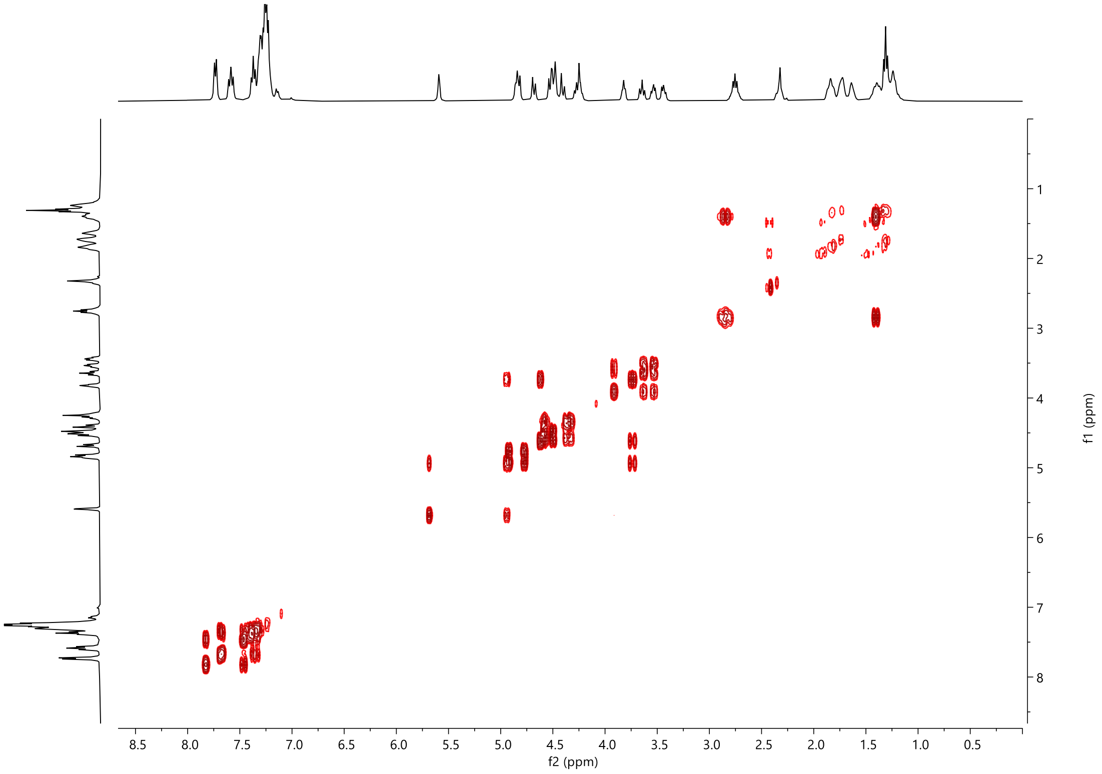

Figure 48. COSY NMR (400 MHz, $CDCl_3$) spectrum of **7k.**

3.2.1. Stereoselectivity study of designed galactose building blocks

Synthesis of model acceptor 2-O-Benzoyl-3,6-bis-O-benzyl-1-O-isopropyl-β-D-galactopyranoside **(6a)**

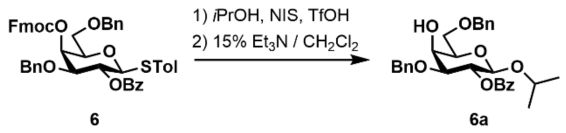

A solution of **6** (0.5 g, 0.6 mmol) and *i*PrOH (80 µL, 0.9 mmol, 1.5 equiv) in anhydrous CH_2Cl_2 (13 mL) was stirred over activated 4 Å molecular sieves for 30 minutes at room temperature. Then, the solution was cooled down to -10 °C and NIS (213 mg, 0.9 mmol, 1.5 equiv) and TfOH (11 µL, 0.1 mmol, 0.2 equiv) were added. The reaction was warmed up to 0 °C and stirred for 1 h. After completion, the reaction mixture was quenched with pyridine, diluted with CH_2Cl_2 (20 mL), filtered and was washed with 10% of $Na_2S_2O_3$ (10 mL, *w/v*). The organic layer was washed with brine (20 mL), dried over Na_2SO_4, filtered, concentrated and purified by column chromatography Hex/EtOAc (10:1 to 4:1, *v/v*) to give the intermedium compound than was further treated with 15% Et_3N in CH_2Cl_2 (15 mL) for 30 min. After completion, the reaction was washed with 10% of citric acid (20 mL, *w/v*) and brine (20 mL), dried over Na_2SO_4 and concentrated under reduced pressure. The crude was purified by column chromatography Hex/EtOAc (gradient from 4:1 to 2:1, *v/v*) to afford **6a** (0.22 g, 0.4 mmol; 72% over two steps). R*f* (Hex/EtOAc 2:1, *v/v*) = 0.35. HR-MS (QTOF) m/z = 529.2205 $[M+Na]^+$, calcd for $C_{30}H_{34}O_7Na$: 529.2202. ^1H NMR (400 MHz, $CDCl_3$) δ 7.93 – 7.90 (m, 2H), 7.53 – 7.46 (m, 1H), 7.38 – 7.33 (m, 2H), 7.27 (d, *J* = 0.9 Hz, 2H), 7.24 – 7.16 (m, 3H), 7.10 – 7.03 (m, 5H), 5.31 (dd, *J* = 9.8, 7.9 Hz, 1H), 4.59 (d, *J* = 12.5 Hz, 1H), 4.52 (s, 2H), 4.43 (d, *J* = 1.2 Hz, 1H), 4.41 (d, *J* = 5.5 Hz, 1H), 4.06 – 4.01 (m, 1H), 3.85 – 3.74 (m, 2H), 3.67 (dd, *J* = 9.8, 5.9 Hz, 1H), 3.57 – 3.52 (m, 2H), 3.51 (d, *J* = 3.4 Hz, 1H), 1.08 (d, *J* = 6.2 Hz, 3H), 0.90 (d, *J* = 6.1 Hz, 3H). ^{13}C NMR (101 MHz, $CDCl_3$) δ 165.47, 138.14, 137.33, 133.08, 130.33, 129.86, 128.60, 128.53, 128.45, 128.03, 127.97, 127.93, 100.14, 78.42, 73.89, 73.59, 72.45, 71.23, 69.19, 66.21, 23.47, 22.08.

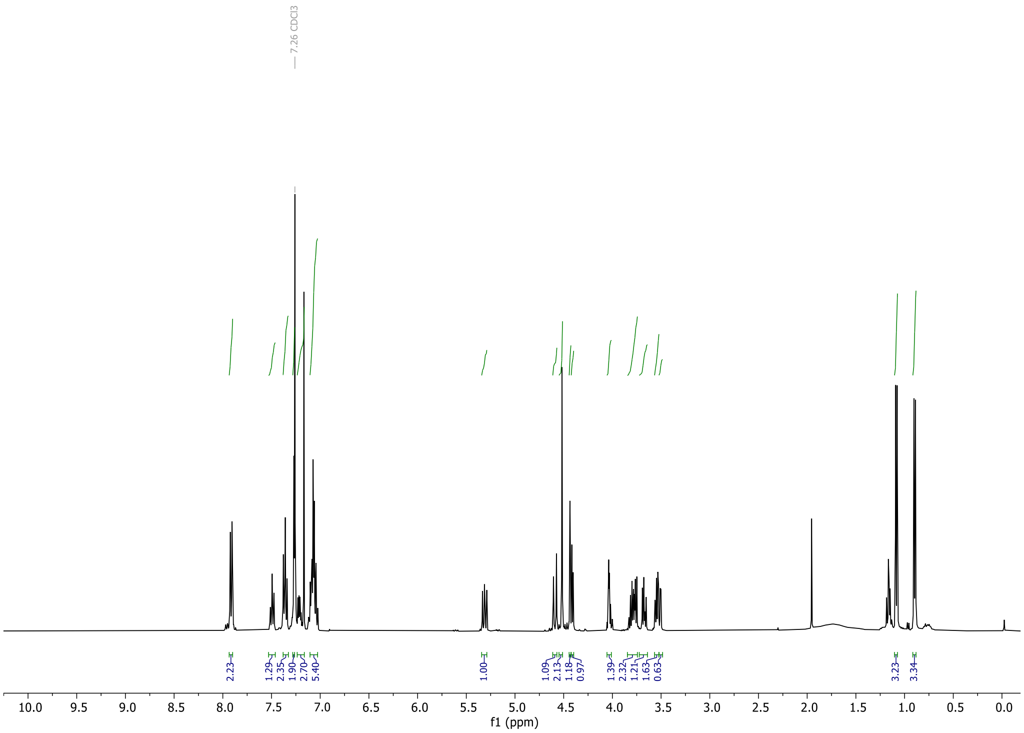

Figure 49. ^{1}H NMR (400 MHz, $CDCl_3$) spectrum of **6a.**

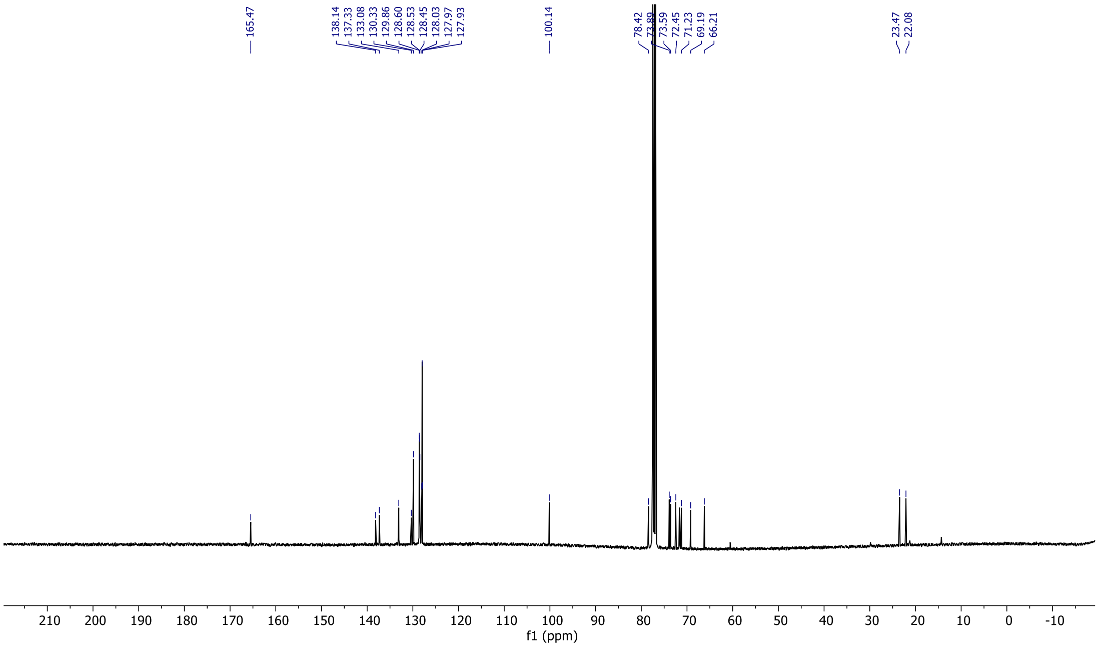

Figure 50. ^{13}C NMR (101 MHz, $CDCl_3$) spectrum of **6a.**

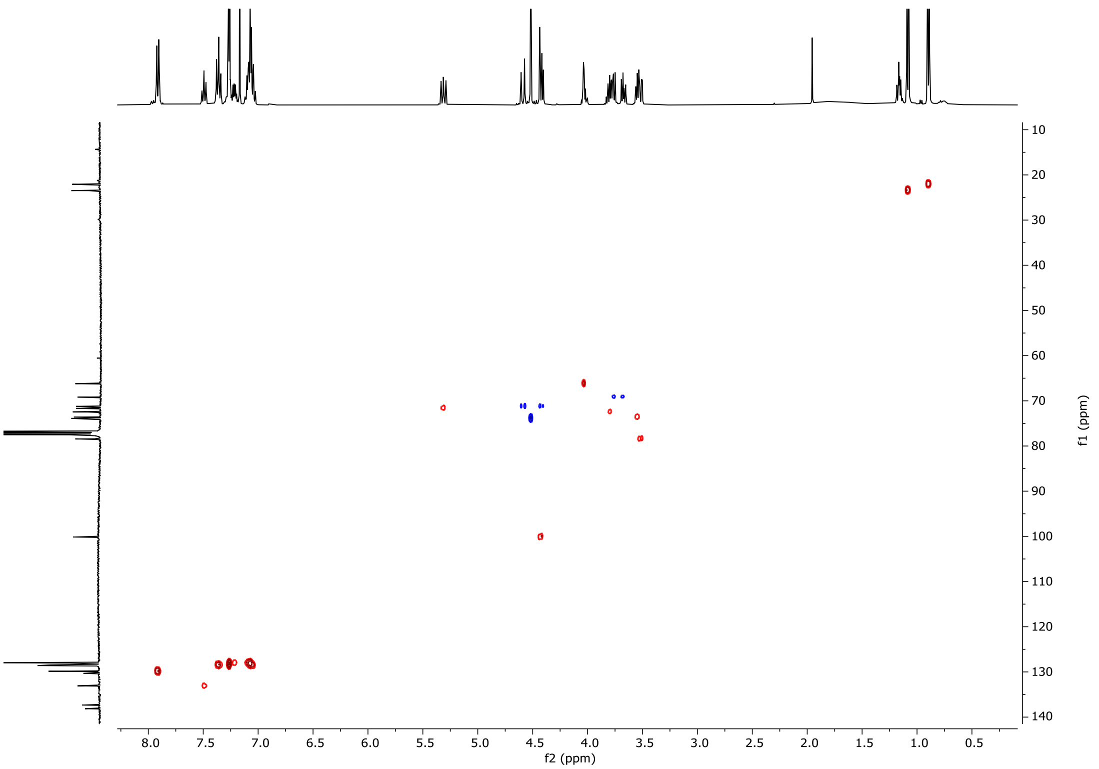

Figure 51. HSQC NMR (400 MHz, $CDCl_3$) spectrum of **6a.**

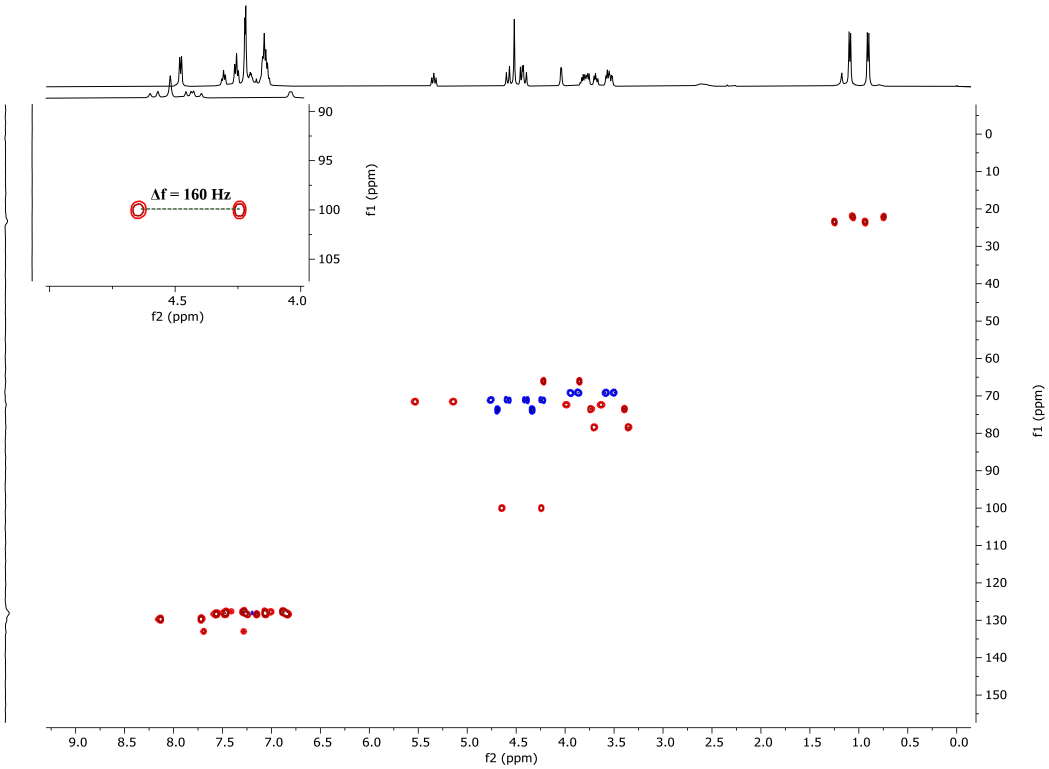

Figure 52. Coupled HSQC NMR (400 MHz, $CDCl_3$) spectrum of **6a.**

Solution-phase galactosylation screening

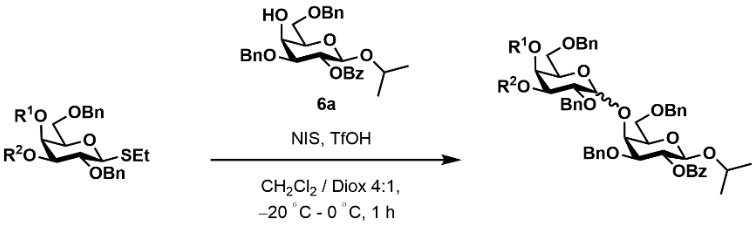

Compound	R^1	R^2
**7e**	PrCO	Fmoc
**7h**	*i*BCO	Fmoc
**7k**	CyCO	Fmoc
**7**	CyCO	CyCO

Compound	R^1	R^2	Yield (%)	α / β
**7.1**	PrCO	Fmoc	79	94:6
**7.2**	*i*BCO	Fmoc	81	98:2
**7.3**	CyCO	Fmoc	85	99:1
**7.4**	CyCO	CyCO	86	only α

Donor (35 µmol, 1.0 equiv) and acceptor **6a** (35 µmol, 1.0 equiv) were co-evaporated with toluene (3×2 mL) and kept under vacuum for 1 h. Anhydrous CH_2Cl_2/Diox 4:1 was added and the mixture was stirred over activated 4Å molecular sieves for 30 minutes at room temperature. Then, the solution was cooled down to -20 °C and NIS (53 µmol, 1.5 equiv) followed by TfOH (60 µL of a 1% solution in CH_2Cl_2, 7 µmol, 0.2 equiv) were added. The reaction was slowly warmed up to 0 °C and stirring for 1 h. After completion, the reaction was quenched with pyridine, diluted with CH_2Cl_2 (20 mL) and filtered. The organic layer was washed with 10% $Na_2S_2O_3$ (20 mL, *w/v*), brine (20 mL), dried over Na_2SO_4, filtered and concentrated. The residue was analyzed (Method A-1) and purified by HPLC (Method A-2).

Analytical NP-HPLC (Method A-1): NP-HPLC was conducted on an Agilent 1200 Series system. A YMC-Diol-300-NP column (150 mm x 4.600 fmm I.D.) was used with a flow rate of 1.00 mL/min and Hex/EtOAc as eluent (2 to 25% EtOAc in 25 minutes).

Preparative NP-HPLC (Method A-2): Preparative NP-HPLC was conducted on an Agilent 1200 Series system. A YMC-Diol-300-NP column (150 mm x 4.600 mm I.D.) was used with a flow rate of 15.00 mL/min and Hex/EtOAc as eluent (2 to 25% EtOAc in 25 minutes).

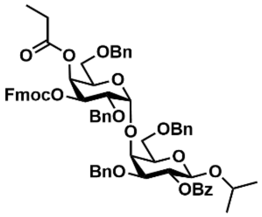

2-O-Benzoyl-3,6-bis-O-benzyl-4-O-[2,6-bis-O-benzyl-3-O-fluorenylmethoxycarbonyl-4-O-propanoate-1-O-isopropyl-α-D-galactopyranosyl]-1-O-β-D- galactopyranoside **(7.1)**

Yield: 79%. Ratio α/β = 94:6. R_t (α) = 16.1 min; R_t (β) = 16.8 min (Method A-1). MALDI-MS *m/z:* 1149.795 $[M+Na]^+$, calcd. for $C_{68}H_{70}O_{15}Na$: 1150.283. Data of major isomer (α): ^{1}H NMR (400 MHz, $CDCl_3$) δ 8.01 – 7.99 (m, 2H), 7.78 – 7.73 (m, 2H), 7.63 (t, *J* = 7.3 Hz, 2H), 7.60 – 7.54 (m, 2H), 7.47 – 7.44 (m, 2H), 7.43 – 7.39 (m, 1H), 7.36 (dd, *J* = 5.7, 1.6 Hz, 4H), 7.31 – 7.27 (m, 4H), 7.24 – 7.18 (m, 4H), 7.16 (d, *J* = 3.5 Hz, 6H), 7.11 – 7.09 (m, 4H), 5.78 (dd, *J* = 3.4, 1.3 Hz, 1H), 5.41 (dt, *J* = 5.5, 2.4 Hz, 1H), 5.40 – 5.35 (m, 1H), 5.14 (d, *J* = 3.6 Hz, 1H), 4.87 (d, *J* = 11.9 Hz, 1H), 4.75 – 4.70 (m, 1H), 4.69 – 4.66 (m, 1H), 4.64 (d, *J* = 4.1 Hz, 1H), 4.61 (s, 1H), 4.54 – 4.52 (m, 1H), 4.38 (d, *J* = 3.7 Hz, 1H), 4.35 (d, *J* = 3.3 Hz, 1H), 4.32 (d, *J* = 3.1 Hz, 2H), 4.29 (s, 1H), 4.17 (d, *J* = 2.9 Hz, 1H), 4.15 (s, 1H), 4.12 (d, *J* = 5.7 Hz, 1H), 4.03 (dd, *J* = 5.6, 2.6 Hz, 1H), 3.87 (ddd, *J* = 6.2, 4.4, 1.3 Hz, 1H), 3.58 (d, *J* = 5.0 Hz, 2H), 3.35 – 3.26 (m, 2H), 2.36 – 2.23 (m, 2H), 1.25 (s, 2H), 1.17 (dd, *J* = 6.2, 2.6 Hz, 3H), 1.10 (t, *J* = 7.5 Hz, 3H), 1.01 – 0.98 (m, 3H). ^{13}C NMR (101 MHz, $CDCl_3$) δ 173.98, 165.36, 154.38, 144.41, 143.82, 141.74, 141.68, 138.63, 138.56, 138.47, 138.23, 133.21, 130.90, 130.19, 128.88, 128.77, 128.72, 128.67, 128.56, 128.37, 128.31, 128.20, 128.14, 128.05, 127.97, 127.77, 127.62, 127.52, 125.87, 125.75, 120.36, 100.96, 100.74, 78.98, 75.37, 75.07, 74.57, 74.04, 73.65, 73.59, 73.47, 72.62, 72.16, 71.75, 70.50, 69.20, 68.29, 67.90, 67.60, 47.19, 30.17, 27.91, 23.79, 22.48, 9.70.

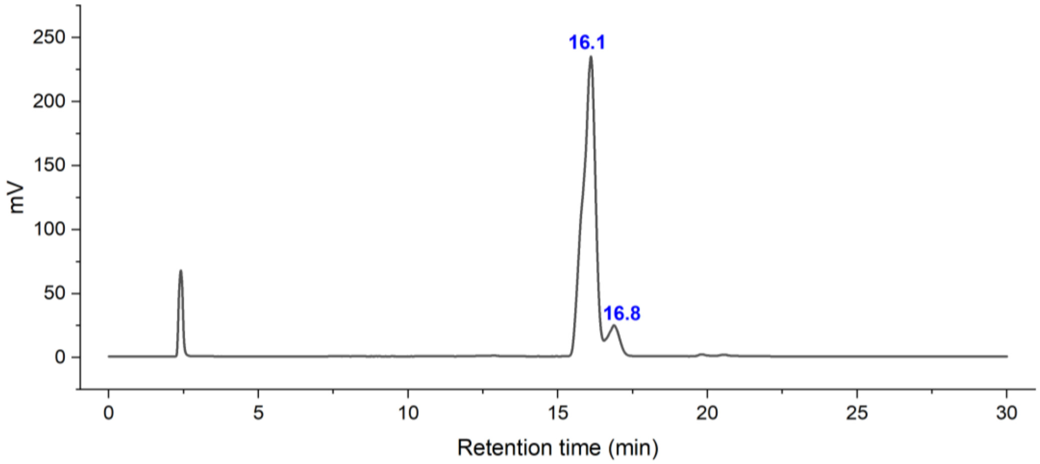

Figure 53. Crude NP-HPLC of compound **7.1.**

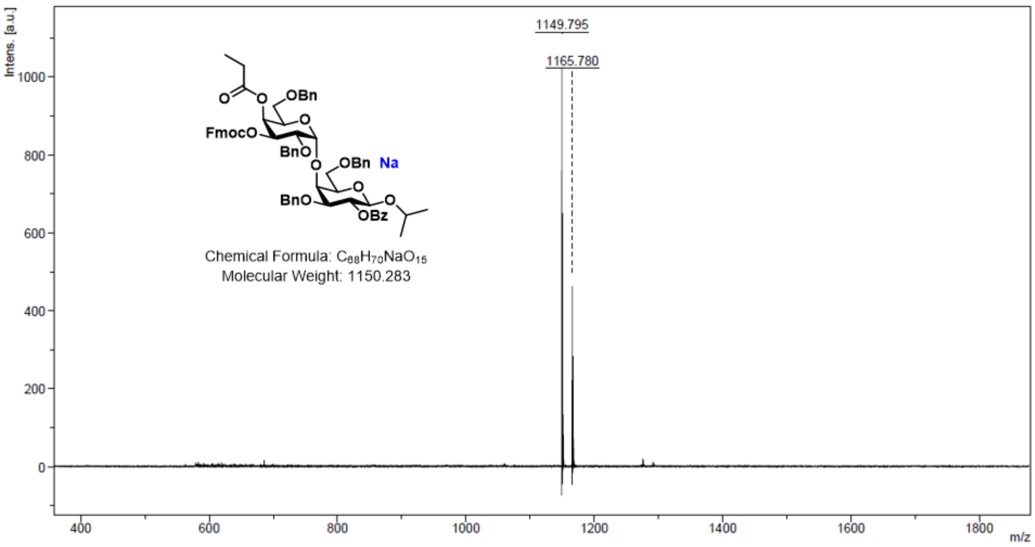

Figure 54. MALDI-MS of dimer **7.1**.

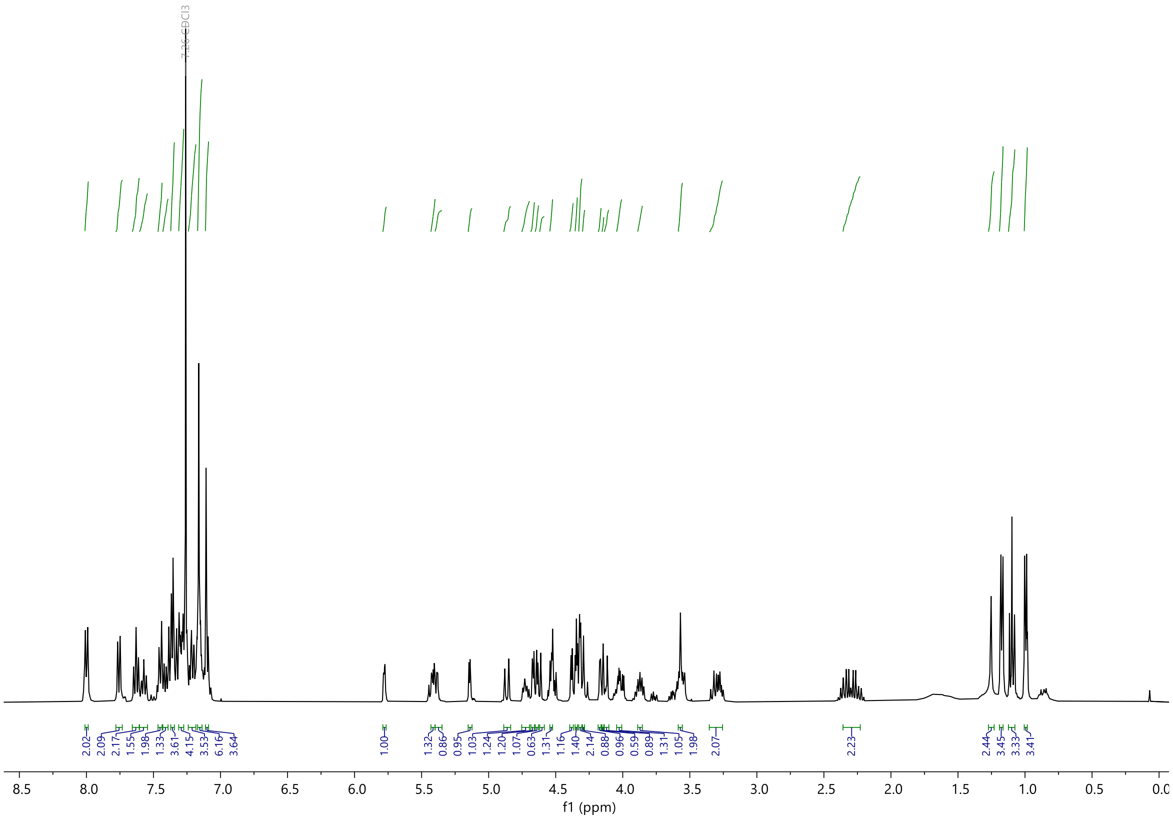

Figure 55. ^{1}H NMR (400 MHz, $CDCl_3$/MeOD) spectrum of isomer (α) of **7.1**.

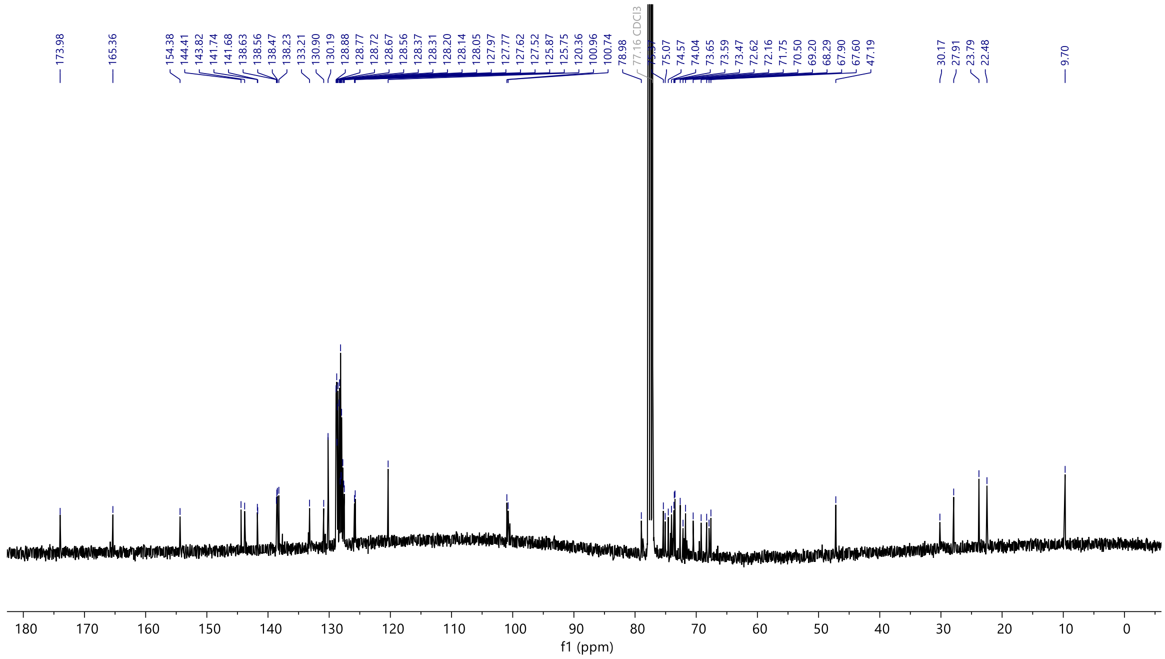

Figure 56. ^{13}C NMR (101 MHz, $CDCl_3$) spectrum of isomer (α) of **7.1**.

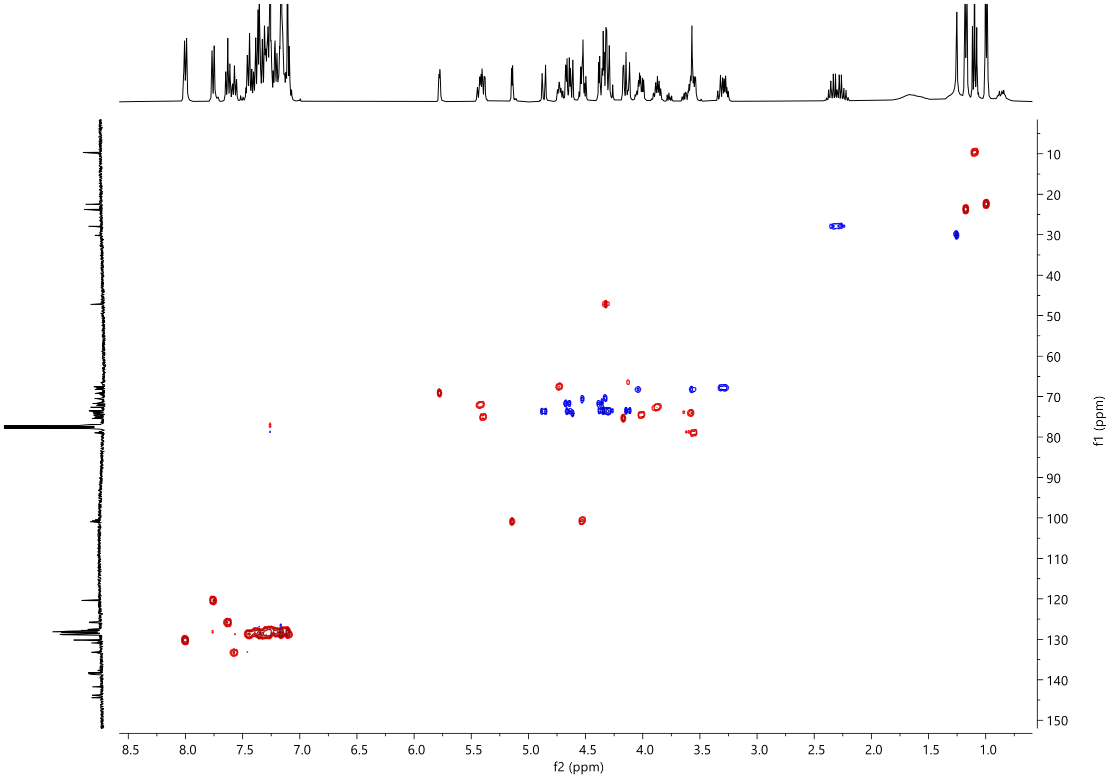

Figure 57. HSQC NMR (400 MHz, $CDCl_3$) spectrum of isomer (α) of **7.1**.

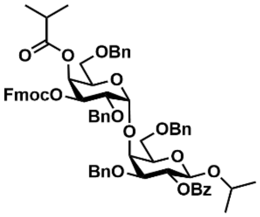

2-O-Benzoyl-3,6-bis-O-benzyl-4-O-[2,6-bis-O-benzyl-3-O-fluorenylmethoxycarbonyl-4-O-(2-methylpropanoate)-1-O-isopropyl-α-D-galactopyranosyl]-1-O-β-D- galactopyranoside **(7.2)**

Yield: 81%. Ratio α/β = 98:2. R_t (α) = 15.5 min; R_t (β) = 16.6 min (Method A-1). MALDI-MS *m/z:* 1164.209 $[M+Na]^+$, calcd. for $C_{69}H_{72}O_{15}Na$: 1164.310. Data of major isomer (α): ^{1}H NMR (400 MHz, $CDCl_3$) δ 8.02 – 7.98 (m, 2H), 7.78 – 7.75 (m, 2H), 7.64 (t, *J* = 7.4 Hz, 2H), 7.61 – 7.54 (m, 1H), 7.45 (d, *J* = 7.8 Hz, 2H), 7.43 – 7.40 (m, 1H), 7.38 – 7.31 (m, 6H), 7.31 – 7.27 (m, 4H), 7.26 – 7.21 (m, 3H), 7.18 – 7.09 (m, 10H), 5.77 (dd, *J* = 3.4, 1.4 Hz, 1H), 5.44 – 5.41 (m, 1H), 5.39 (d, *J* = 3.3 Hz, 1H), 5.15 (d, *J* = 3.6 Hz, 1H), 4.83 (d, *J* = 12.0 Hz, 1H), 4.76 (dd, *J* = 7.5, 5.6 Hz, 1H), 4.68 (s, 1H), 4.66 (d, *J* = 3.7 Hz, 1H), 4.62 (d, *J* = 6.3 Hz, 1H), 4.54 – 4.51 (m, 1H), 4.39 (s, 1H), 4.36 (d, *J* = 2.6 Hz, 2H), 4.34 (d, *J* = 1.2 Hz, 1H), 4.32 (s, 1H), 4.32 – 4.29 (m, 1H), 4.19 – 4.17 (m, 2H), 4.10 – 4.04 (m, 1H), 4.00 – 3.97 (m, 1H), 3.88 (p, *J* = 6.1 Hz, 1H), 3.59 – 3.54 (m, 2H), 3.36 – 3.27 (m, 2H), 2.56 (h, *J* = 7.0 Hz, 1H), 1.26 (s, 1H), 1.19 – 1.15 (m, 4H), 1.13 (d, *J* = 2.1 Hz, 3H), 1.11 (s, 2H), 1.00 (d, *J* = 6.1 Hz, 3H). ^{13}C NMR (101 MHz, $CDCl_3$) δ 176.43, 165.33, 154.37, 144.39, 143.87, 141.74, 141.69, 138.57, 138.52, 138.23, 133.19, 130.90, 130.19, 128.88, 128.77, 128.65, 128.56, 128.45, 128.16, 128.08, 127.97, 127.92, 127.71, 127.60, 127.52, 125.87, 125.77, 120.36, 100.90, 100.72, 78.86, 75.31, 75.04, 74.31, 74.03, 73.61, 73.51, 73.43, 72.57, 72.15, 71.65, 70.41, 68.93, 68.30, 67.94, 67.65, 47.20, 34.48, 23.78, 22.46, 19.73, 19.25.

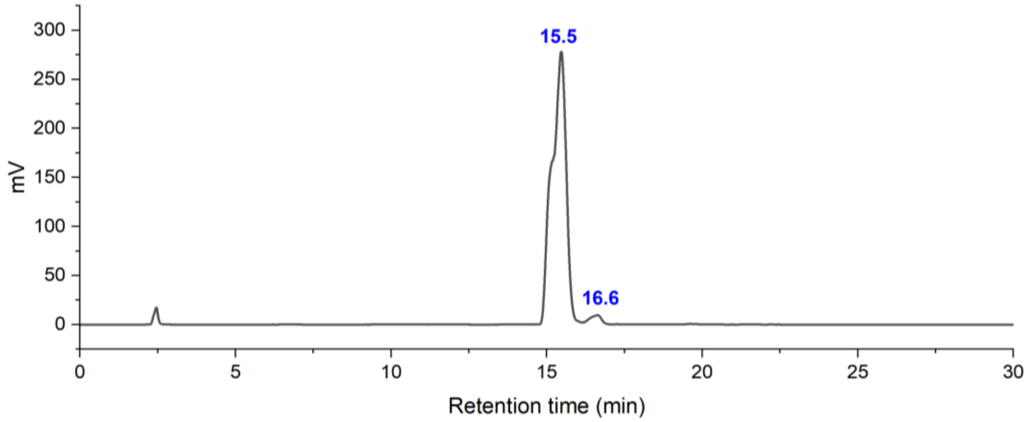

Figure 58. Crude NP-HPLC of compound **7.2**.

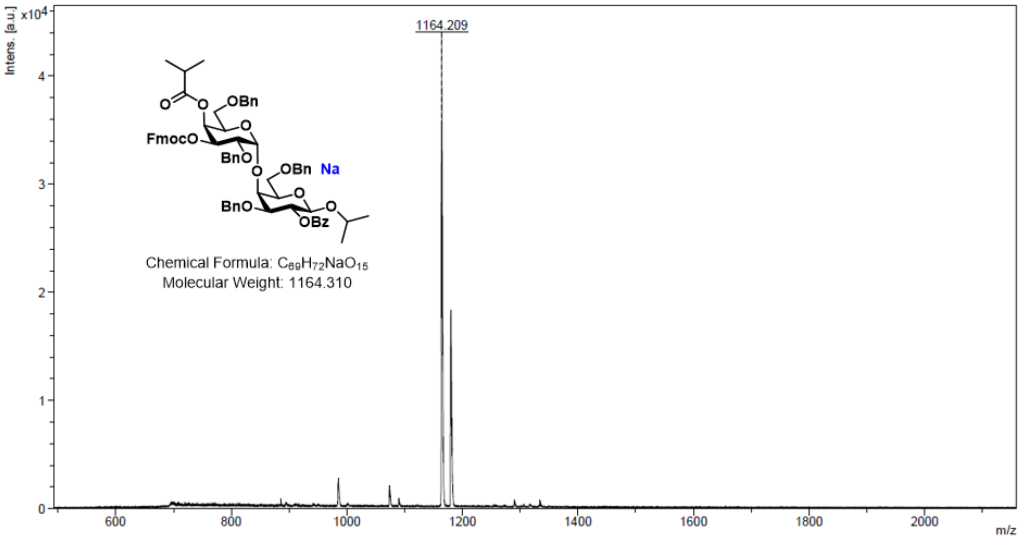

Figure 59. Figure 60. MALDI-MS of dimer **7.2**.

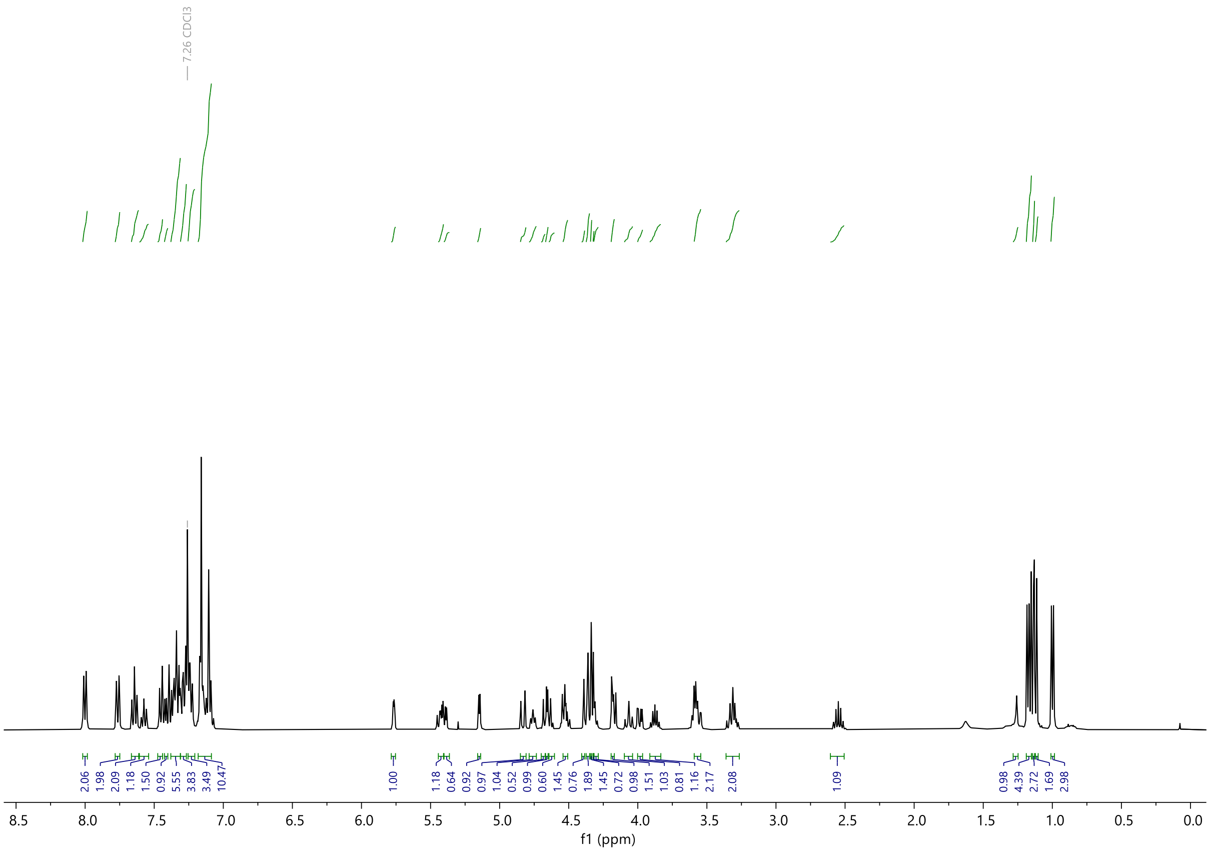

Figure 61. ^{1}H NMR (400 MHz, $CDCl_3$/MeOD) spectrum of isomer (α) of **7.2**.

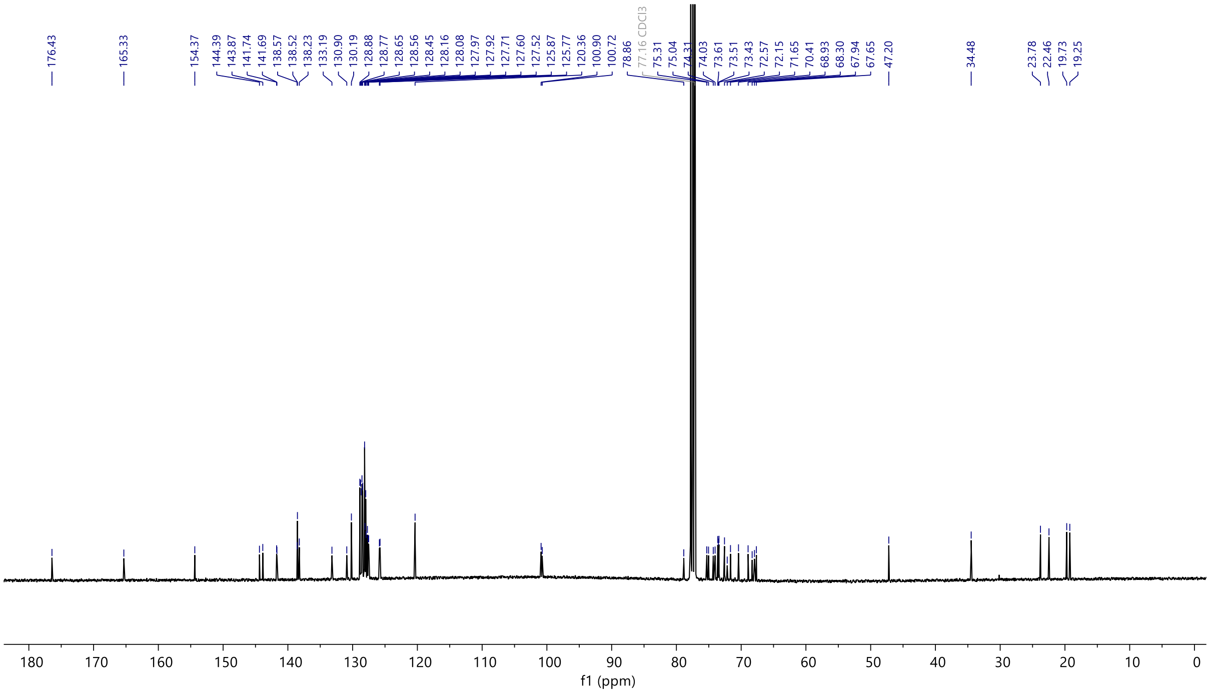

Figure 62. ^{13}C NMR (101 MHz, $CDCl_3$) spectrum of isomer (α) of **7.2**.

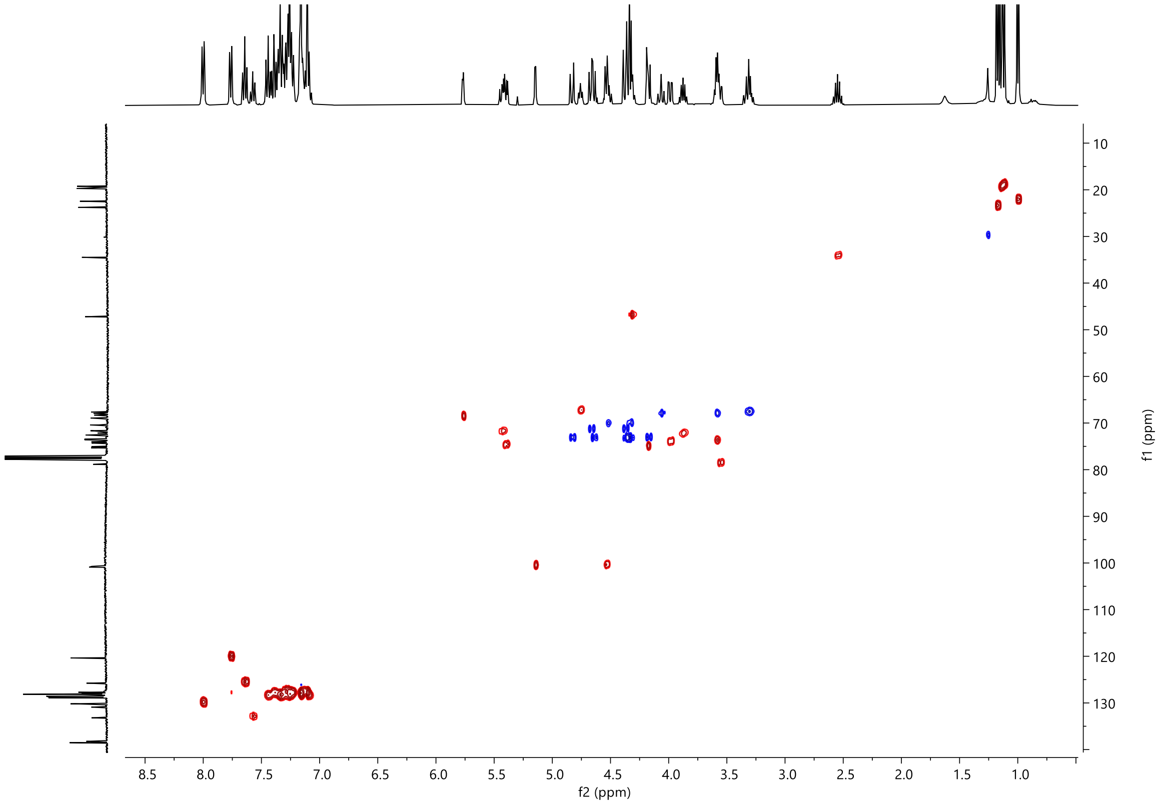

Figure 63. HSQC NMR (400 MHz, $CDCl_3$) spectrum of isomer (α) of **7.2**.

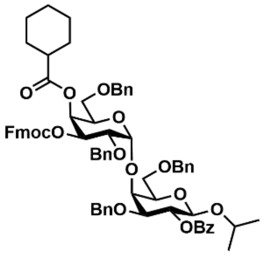

2-O-Benzoyl-3,6-bis-O-benzyl-4-O-[2,6-bis-O-benzyl-3-O-fluorenylmethoxycarbonyl-4-O-cyclohexanecarbonyl-1-O-isopropyl-α-D-galactopyranosyl]-1-O-β-D- galactopyranoside **(7.3)**

Yield: 85%. Ratio α/β = 99:1. R_t (α) = 15.6 min; R_t (β) = 16.7 min (Method A-1). MALDI-MS *m/z:* 1203.860 $[M+Na]^+$, calcd. for $C_{72}H_{76}O_{15}Na$: 1204.375 Data of major isomer (α): ^1H NMR (400 MHz, $CDCl_3$) δ 8.01 (dq, *J* = 7.2, 1.8 Hz, 2H), 7.77 – 7.74 (m, 2H), 7.63 (t, *J* = 7.5 Hz, 2H), 7.57 (ddd, *J* = 7.4, 5.6, 1.4 Hz, 1H), 7.48 – 7.43 (m, 2H), 7.42 – 7.34 (m, 6H), 7.30 (ddd, *J* = 8.4, 2.7, 0.9 Hz, 4H), 7.24 – 7.14 (m, 10H), 7.13 – 7.09 (m, 4H), 5.78 (dd, *J* = 3.4, 1.3 Hz, 1H), 5.43 – 5.40 (m, 1H), 5.41 – 5.38 (m, 1H), 5.15 (d, *J* = 3.6 Hz, 1H), 4.87 (d, *J* = 11.8 Hz, 1H), 4.75 – 4.70 (m, 1H), 4.70 – 4.66 (m, 1H), 4.64 (d, *J* = 4.0 Hz, 1H), 4.61 (s, 1H), 4.55 – 4.50 (m, 2H), 4.39 (d, *J* = 3.6 Hz, 1H), 4.35 (d, *J* = 3.2 Hz, 1H), 4.32 (d, *J* = 3.1 Hz, 2H), 4.30 (s, 1H), 4.17 (d, *J* = 2.9 Hz, 1H), 4.14 (d, *J* = 5.8 Hz, 1H), 4.12 (d, *J* = 3.6 Hz, 1H), 4.06 – 3.99 (m, 2H), 3.89 – 3.85 (m, 1H), 3.57 (q, *J* = 4.2 Hz, 2H), 3.36 – 3.22 (m, 2H), 2.37 – 2.22 (m, 2H), 1.26 (s, 3H), 1.18 (dd, *J* = 6.2, 2.5 Hz, 4H), 1.10 (t, *J* = 7.5 Hz, 4H), 1.00 (dd, *J* = 6.1, 2.5 Hz, 4H). ^{13}C NMR (101 MHz, $CDCl_3$) δ 173.97, 165.35, 154.37, 144.40, 143.81, 141.73, 141.67, 138.62, 138.55, 138.46, 138.22, 133.21, 130.89, 130.18, 128.91, 128.87, 128.76, 128.71, 128.66, 128.55, 128.35, 128.30, 128.19, 128.13, 128.04, 127.96, 127.77, 127.61, 127.52, 125.86, 125.74, 120.36, 100.95, 100.45, 78.97, 78.70, 75.37, 75.07, 74.56, 74.21, 74.03, 73.64, 73.57, 73.46, 72.61, 72.15, 71.74, 70.49, 69.19, 68.28, 67.89, 67.59, 47.18, 30.16, 27.91, 23.77, 22.47.

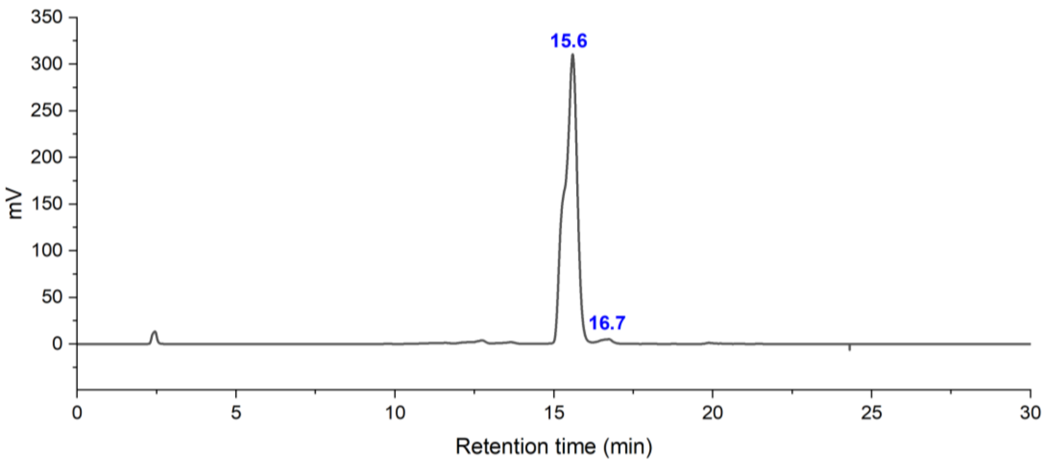

Figure 64. Crude NP-HPLC of compound **7.3.**

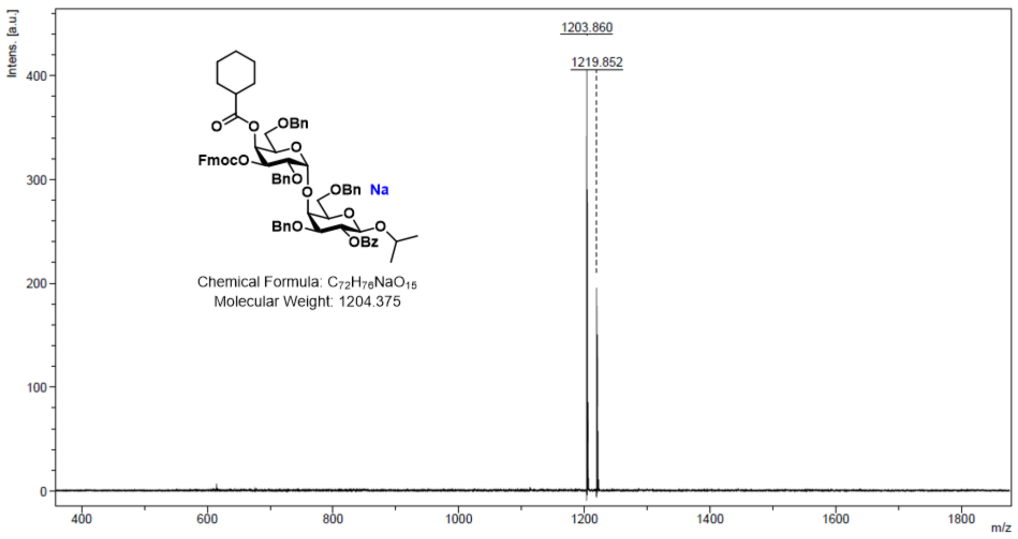

Figure 65. Figure 66. MALDI-MS of dimer **7.3**.

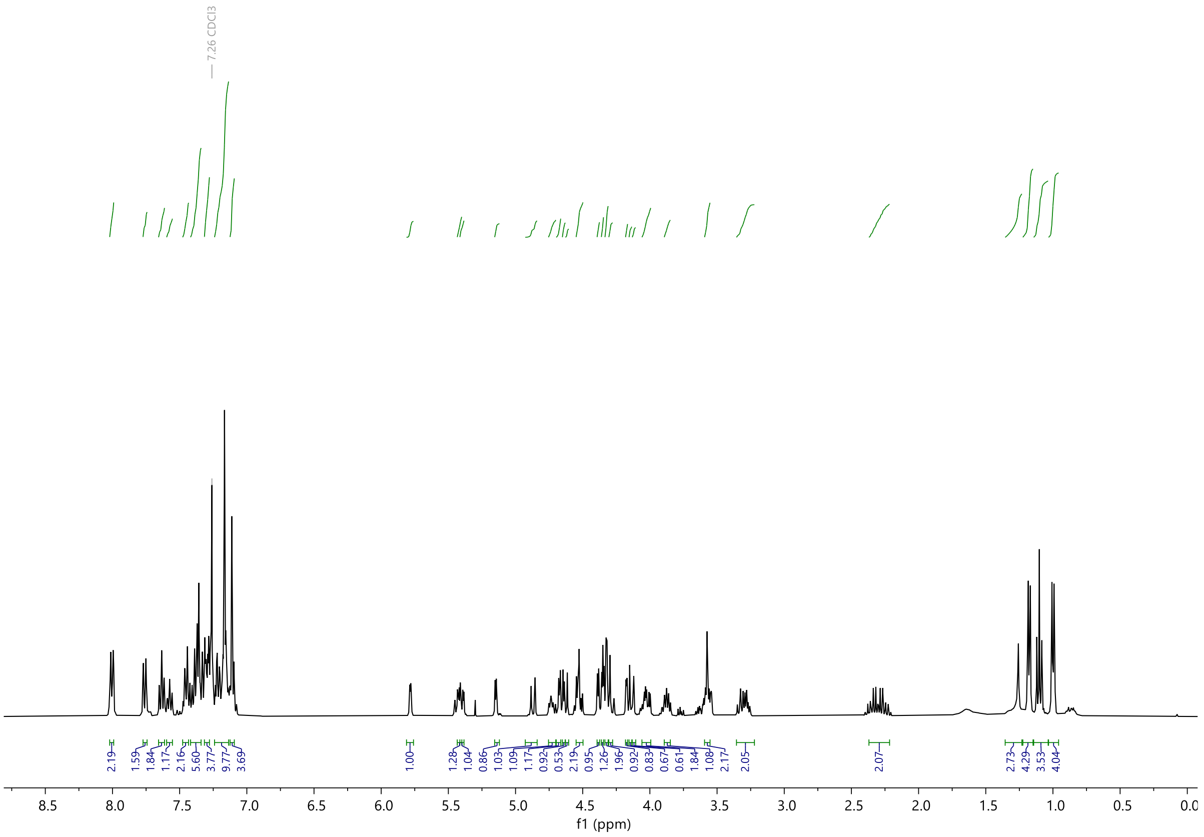

Figure 67. ^{1}H NMR (400 MHz, $CDCl_3$/MeOD) spectrum of isomer (α) of **7.3**.

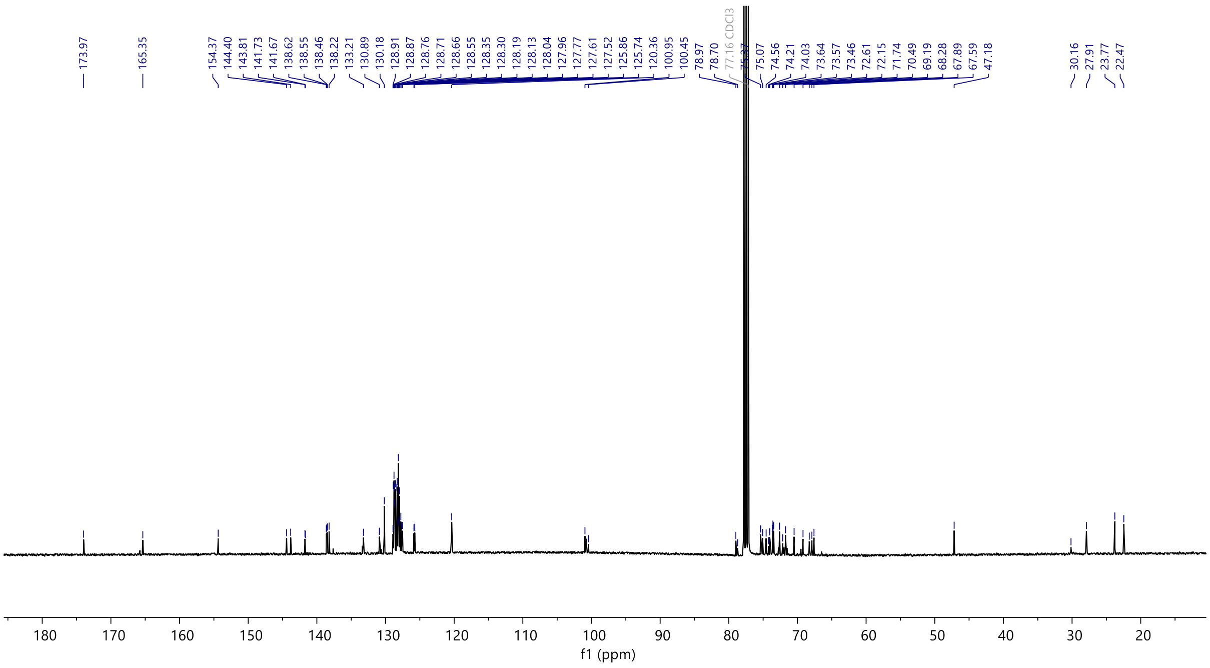

Figure 68. ^{13}C NMR (101 MHz, $CDCl_3$) spectrum of isomer (α) of **7.3**.

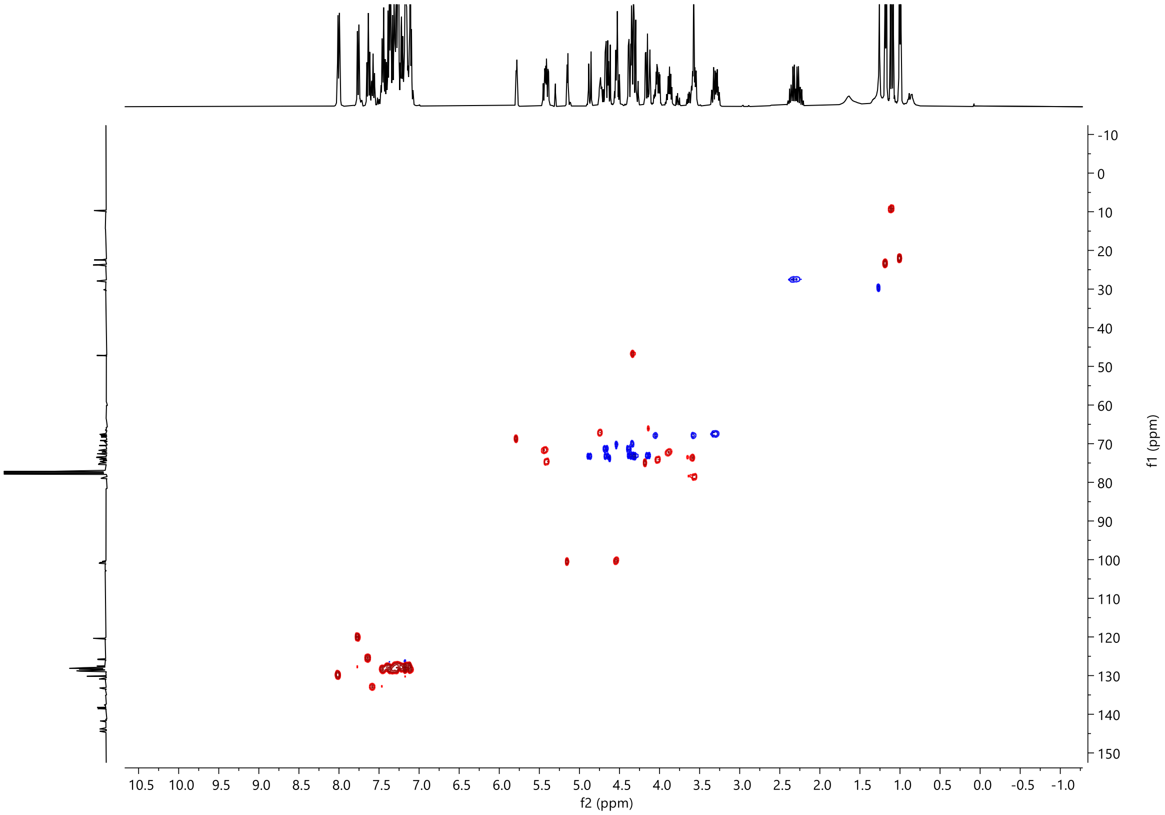

Figure 69. HSQC NMR (400 MHz, $CDCl_3$) spectrum of isomer (α) of **7.3**.

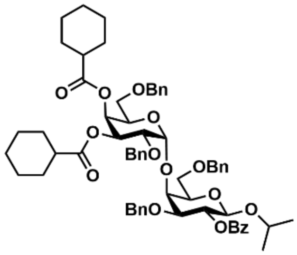

2-O-Benzoyl-3,6-bis-O-benzyl-4-O-[2,6-bis-O-benzyl-3,4-bis-O-(cyclohexanecarbonyl)-1-O-isopropyl-α-D-galactopyranosyl]-1-O-β-D-galactopyranoside **(7.4)**

Yield: 86%. Ratio α/β = only α observed. R_t (α) = 18.6 min (Method A-1). MALDI-MS *m/z:* 1091.330 $[M+Na]^+$, calcd for $C_{64}H_{76}O_{14}Na$: 1092.288. Data of detected isomer (α): ^1H NMR (400 MHz, $CDCl_3$) δ 8.04 – 7.96 (m, 2H), 7.59 – 7.52 (m, 1H), 7.48 – 7.39 (m, 2H), 7.36 – 7.27 (m, 7H), 7.24 – 7.20 (m, 2H), 7.18 – 7.13 (m, 6H), 7.12 – 7.08 (m, 4H), 5.65 (d, *J* = 1.3 Hz, 1H), 5.53 (dd, *J* = 10.7, 3.2 Hz, 1H), 5.41 (dd, *J* = 10.3, 7.8 Hz, 1H), 5.14 (d, *J* = 3.5 Hz, 1H), 4.81 – 4.64 (m, 3H), 4.64 – 4.48 (m, 2H), 4.41 – 4.24 (m, 4H), 4.21 – 4.10 (m, 2H), 4.06 – 3.83 (m, 3H), 3.62 – 3.52 (m, 3H), 3.32 – 3.26 (m, 2H), 2.21 (dtt, *J* = 32.8, 11.4, 3.6 Hz, 2H), 1.92 – 1.78 (m, 4H), 1.78 – 1.57 (m, 9H), 1.46 – 1.30 (m, 5H), 1.31 – 1.15 (m, 12H), 1.00 (d, *J* = 6.1 Hz, 3H), 0.95 – 0.85 (m, 2H). ^{13}C NMR (101 MHz, $CDCl_3$) δ 175.01, 174.77, 165.31, 138.71, 138.61, 138.52, 138.28, 133.17, 130.93, 130.19, 128.86, 128.71,

128.69, 128.64, 128.55, 128.37, 128.26, 128.12, 128.05, 127.95, 127.89, 127.70, 100.96, 100.71, 78.89, 75.15, 74.60, 74.09, 73.57, 73.48, 73.23, 72.55, 72.21, 71.64, 70.44, 69.27, 68.34, 68.18, 67.87, 43.66, 43.56, 32.05, 29.66, 29.34, 29.24, 29.15, 26.22, 26.00, 25.94, 25.76, 23.77, 23.12, 22.47, 14.59.

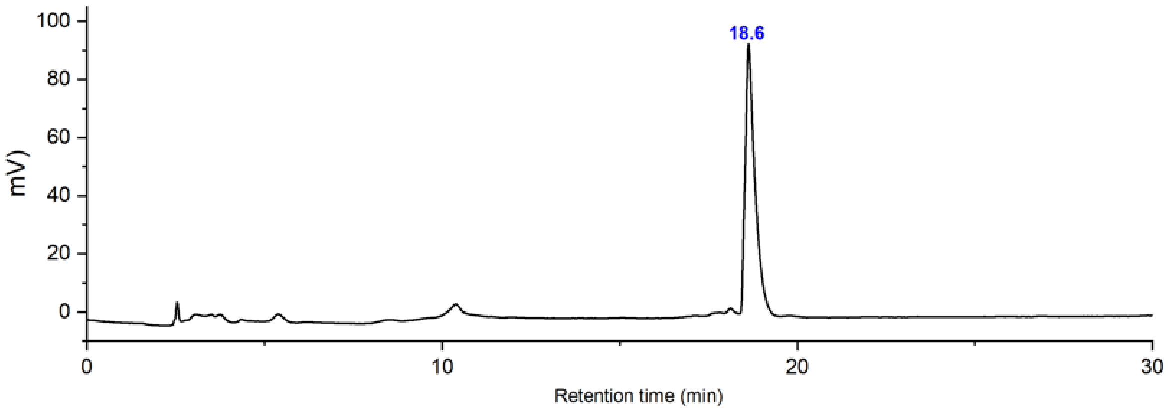

Figure 70. Crude NP-HPLC of compound **7.4**. No β-product detected.

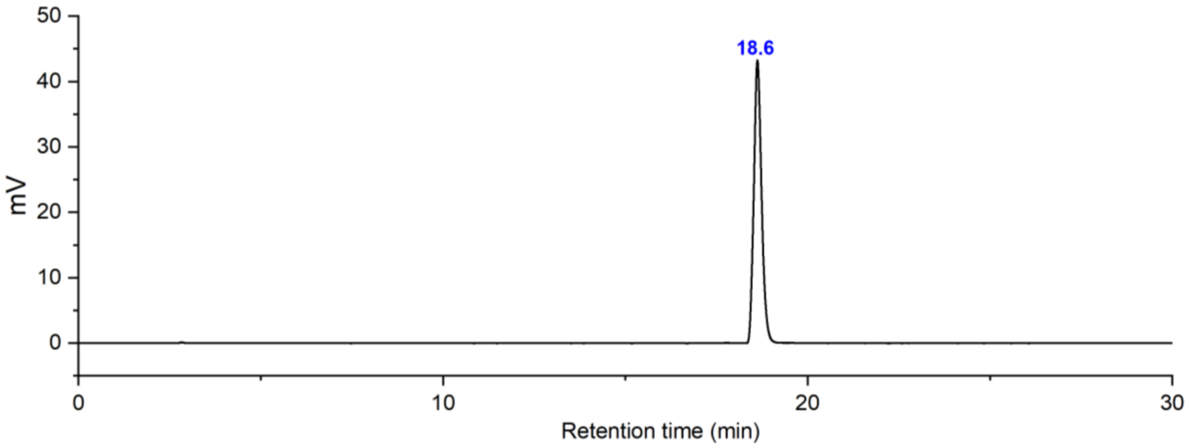

Figure 71. NP-HPLC of purified detected isomer (α) of compound **7.4**.

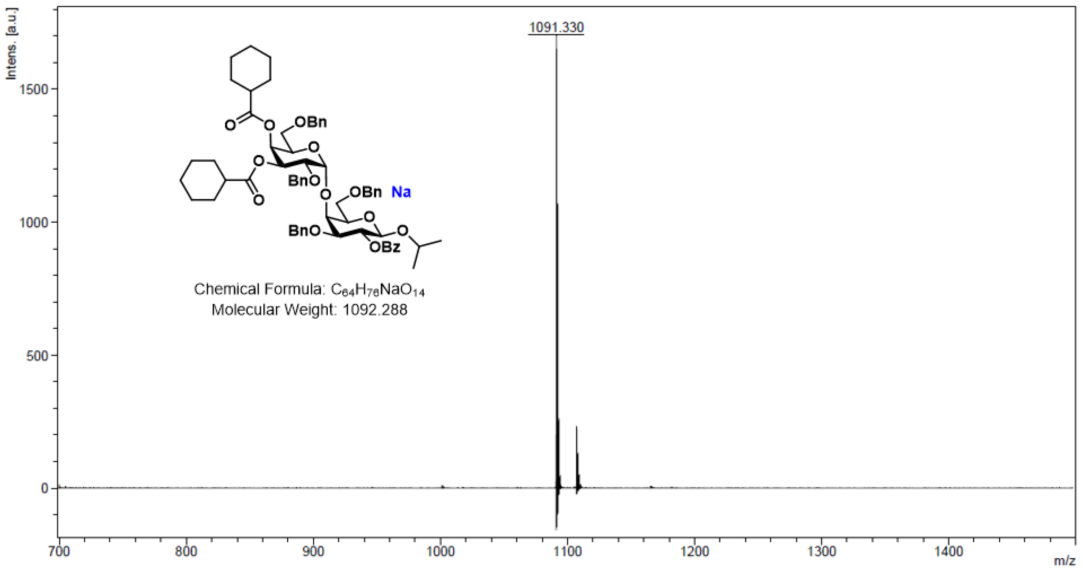

Figure 72. MALDI-MS of dimer **7.4**.

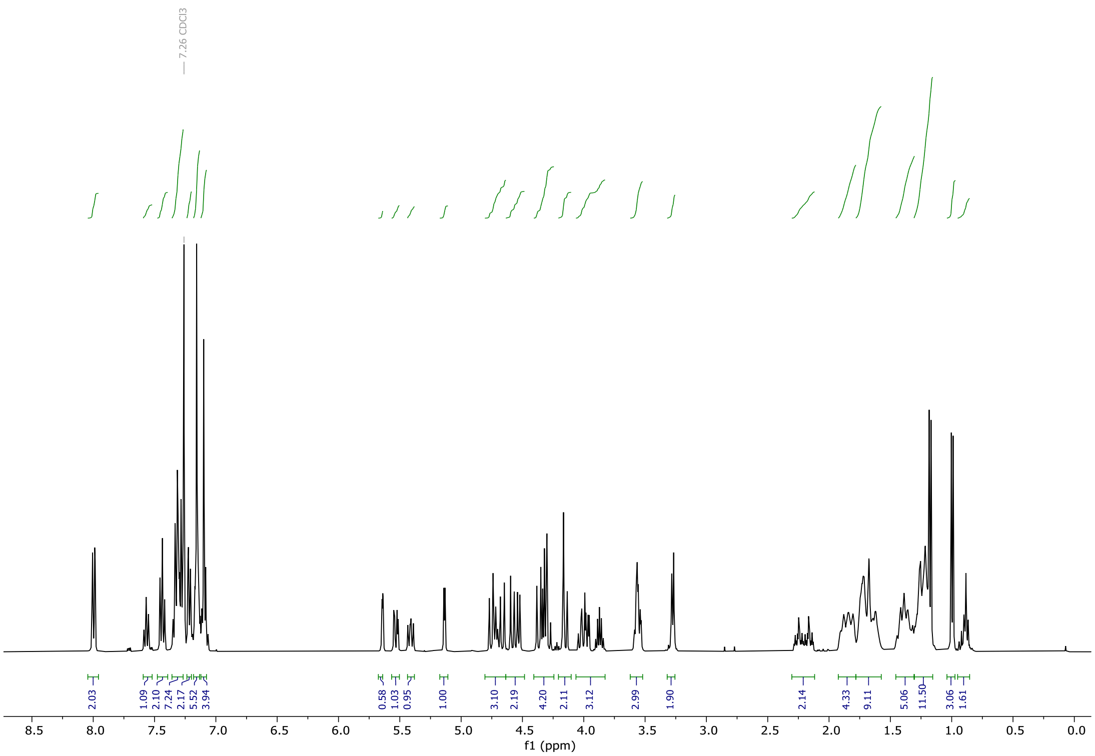

Figure 73. ^{1}H NMR (400 MHz, $CDCl_3$) spectrum of isomer (α) of **7.4**.

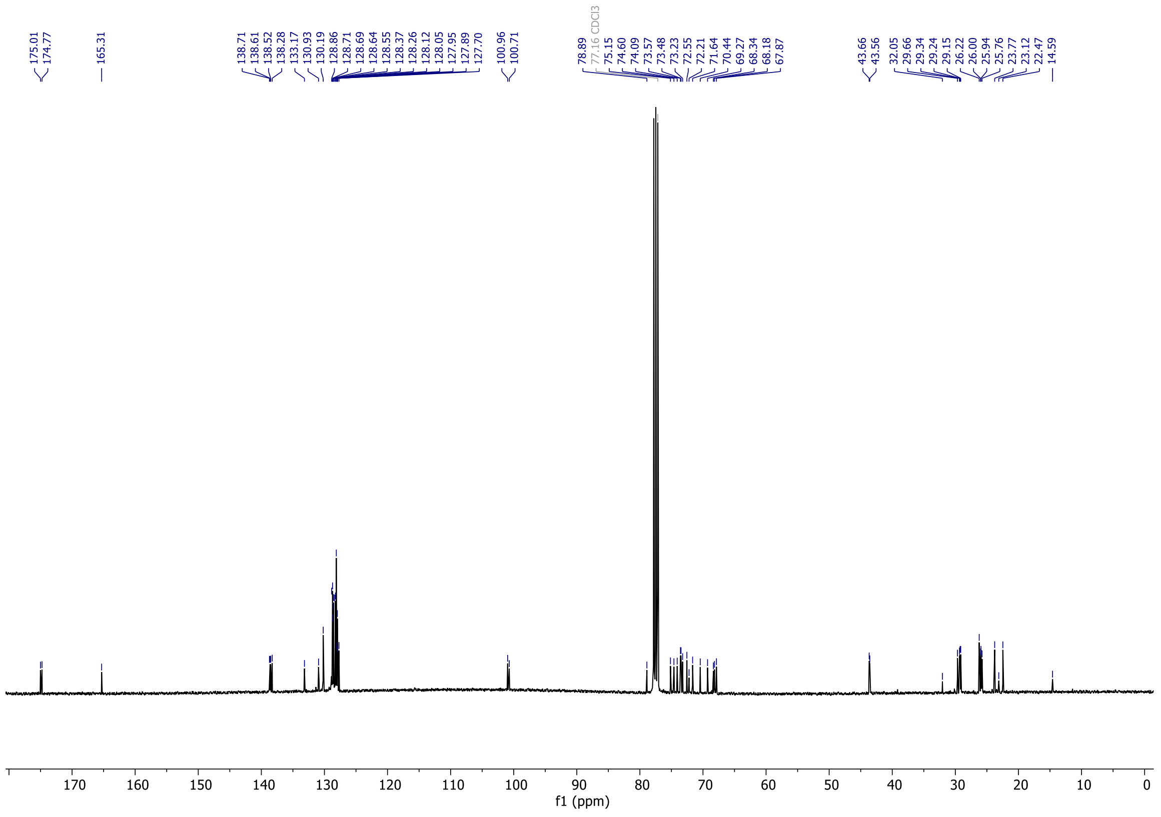

Figure 74. ^{13}C NMR (101 MHz, $CDCl_3$) spectrum of isomer (α) of **7.4**.

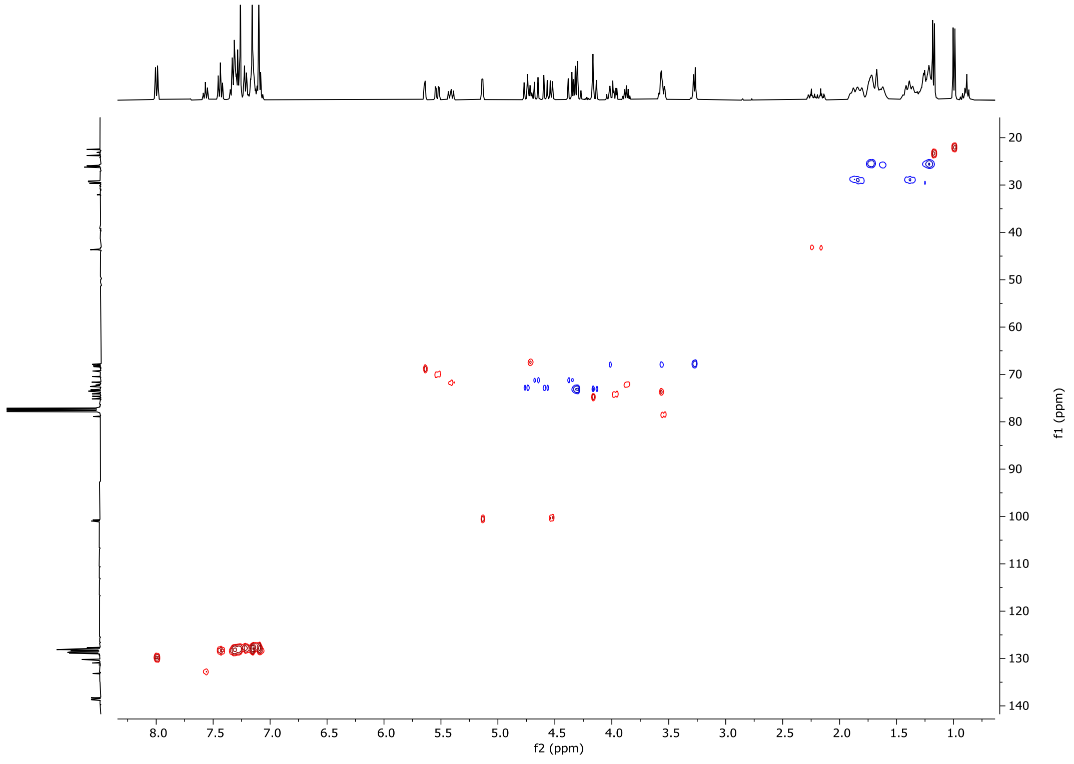

Figure 75. HSQC NMR (400 MHz, $CDCl_3$) spectrum of isomer (α) of **7.4**.

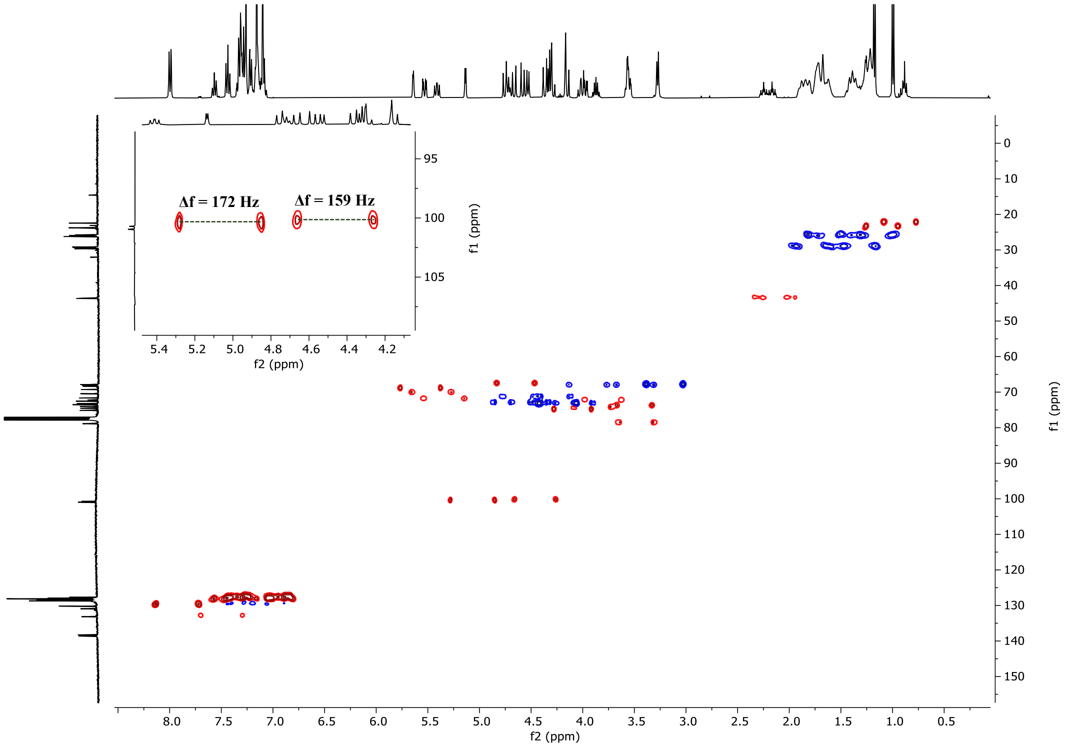

Figure 76. Coupled- HSQC NMR (400 MHz, $CDCl_3$) spectrum of isomer (α) of **7.4**.

3.3. Synthesis of Cholesterol derivative

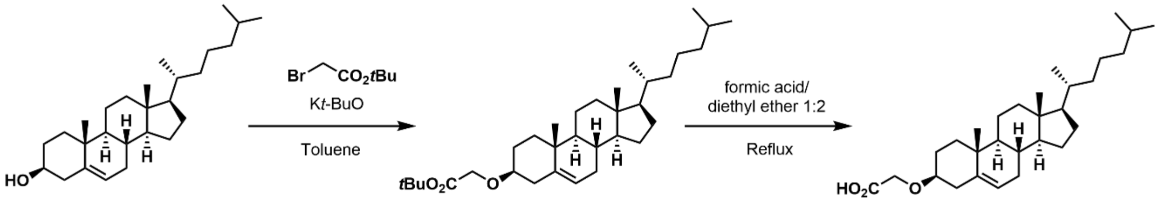

According to a published method,[5] cholesterol **11a** (3.0 g, 7.8 mmol) and potassium *t*-butoxide (0.4 g, 15.5 mmol) were mixed in toluene (100 mL) and the suspension was stirred for 3 h. Then, *t*-butyl bromoacetate (2.3 mL, 15.5 mmol) was added and the reaction mixture was stirred overnight. Afterwards, the reaction was diluted with EtOAc (200 mL) and the mixture was washed with water (50 mL), brine (50 mL) and the organic layer separated and dried over Na_2SO_4, filtered, concentrated and purified by column chromatography Hex/EtOAc (gradient from 10:1, *v/v*) to afford **11b** (2.3 g, 60%) as a white solid, R*f* (Hex/EtOAc 10:1, *v/v*) = 0.6. Next, a solution of **11b** (2.3 g, 4.6 mmol) in HCO_2H/diethyl ether (30 mL 1:2 *v*/*v*) was refluxed for 4 h. Solvents were removed at reduced pressure to afford cholesterol derivative **11** (2.0 g, 99% yield) as a white solid.

HR-MS (QTOF) m/z = 443.3521 [M-H]$^-$, calcd for $C_{29}H_{47}O_3^-$: 443.3531. ^{1}H NMR (400 MHz, $CDCl_3$) δ 5.36 (dt, *J* = 5.7, 1.9 Hz, 1H), 4.16 (s, 2H), 3.29 (tt, *J* = 11.3, 4.5 Hz, 1H), 2.38 (ddd, *J* = 13.2, 4.9, 2.2 Hz, 1H), 2.28 (ddt, *J* = 13.7, 10.5, 2.7 Hz, 1H), 2.02 (t, *J* = 3.6 Hz, 1H), 1.99 (t, *J* = 3.1 Hz, 1H), 1.95 (td, *J* = 4.6, 2.4 Hz, 1H), 1.92 – 1.79 (m, 3H), 1.62 – 1.42 (m, 7H), 1.40 – 1.21 (m, 4H), 1.19 – 1.03 (m, 7H), 1.00 (s, 4H), 0.91 (d, *J* = 6.5 Hz, 3H), 0.87 (d, *J* = 1.9 Hz, 3H), 0.85 (d, *J* = 1.8 Hz, 3H), 0.67 (s, 3H). ^{13}C NMR (100 MHz, $CDCl_3$) δ 175.05, 140.18, 122.45, 80.49, 65.27, 56.87, 56.29, 50.25, 42.44, 39.88, 39.65, 38.77, 37.14, 36.90, 36.32, 35.91, 32.05, 31.98, 28.35, 28.18, 28.14, 24.41, 23.96, 22.95, 22.70, 21.20, 19.46, 18.85, 11.99.

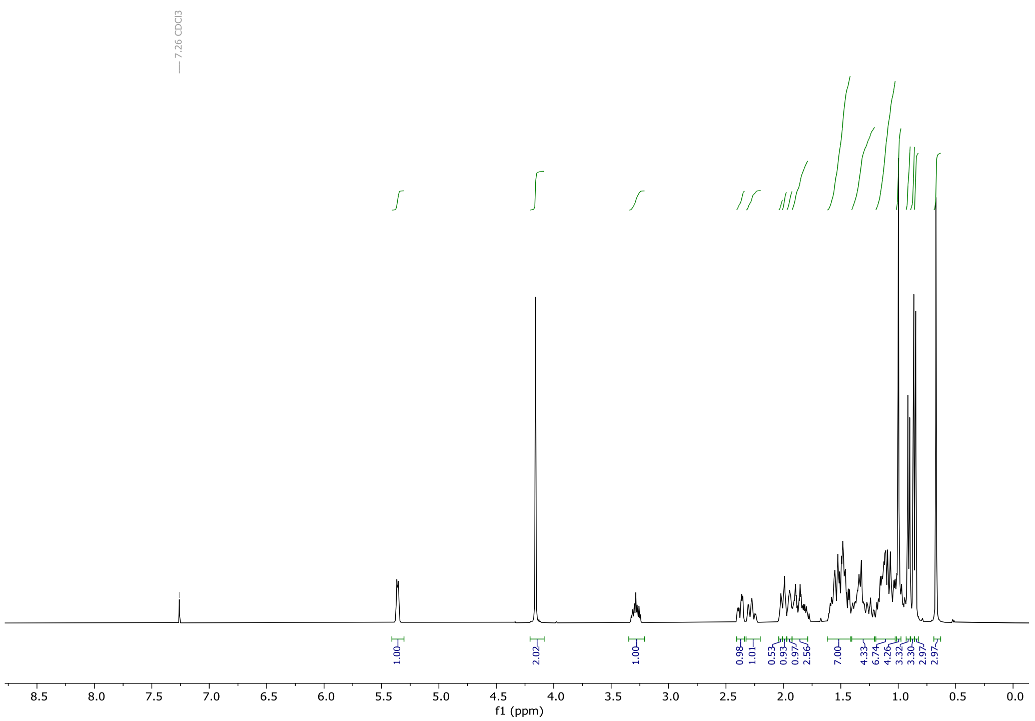

Figure 77. ^{1}H NMR (400 MHz, $CDCl_3$) spectrum of **11**.

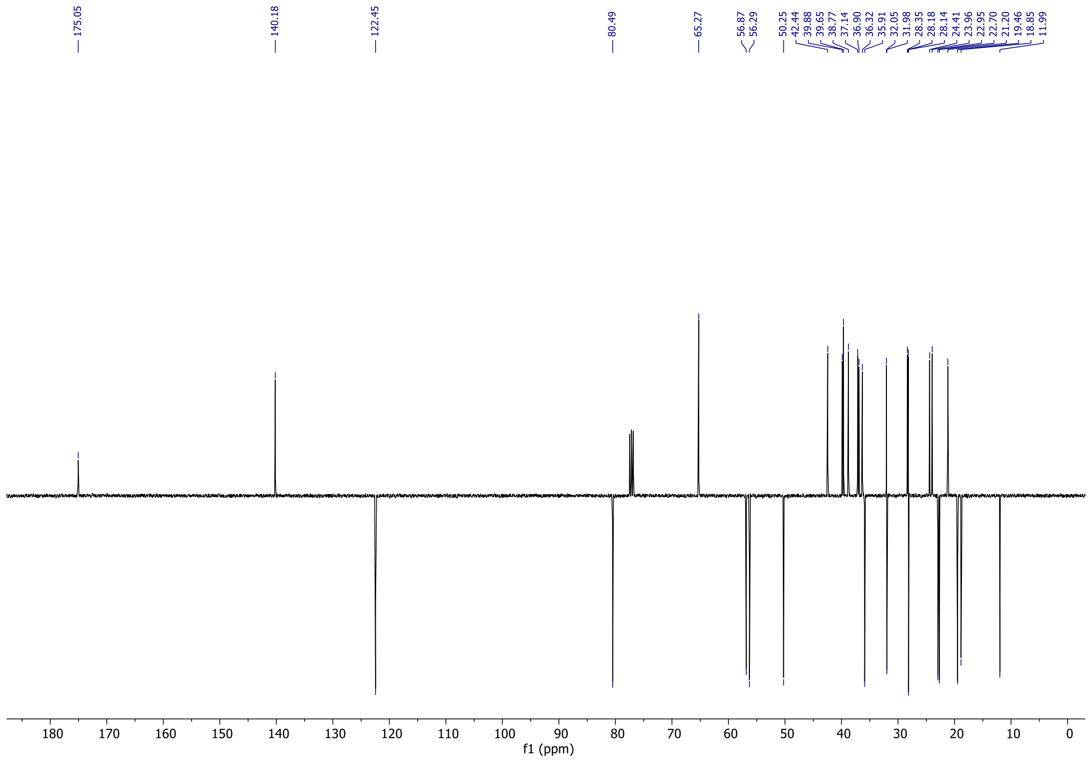

Figure 78. ^{13}C NMR (101 MHz, $CDCl_3$) spectrum of **11.**

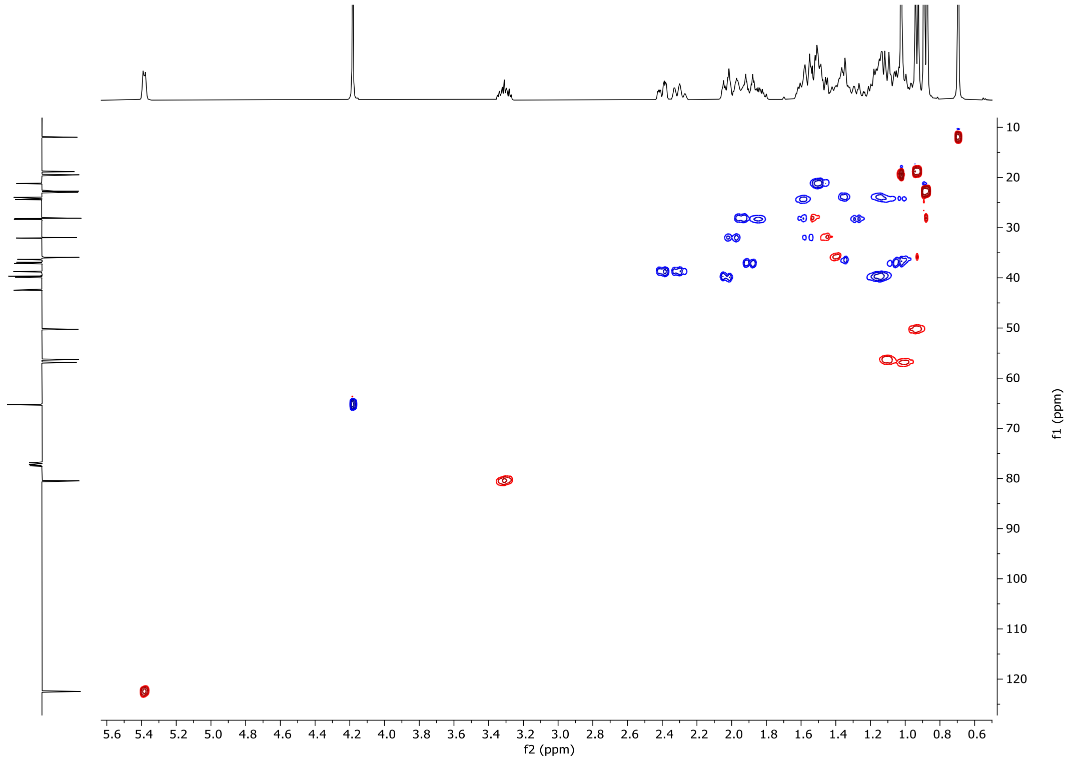

Figure 79. HSQC NMR (400 MHz, $CDCl_3$) spectrum of **11.**

4. Resin Loading of Hle Linker

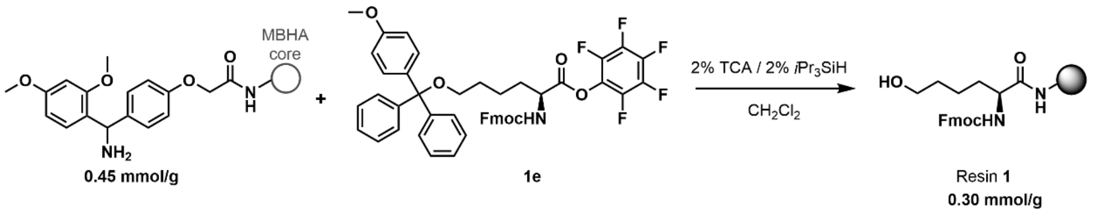

After pre-swelling of the MBHA Rink Amide resin (0.45 mmol/g, 0.2 mmol) and Fmoc group removal, **1e** (0.130 mg, 0.16 mmol, 0.8 equiv) was dissolved in DMF, added to the resin and stirred at room temperature twice for two hours. Kaiser test proved the completion of the reaction. Adding a solution of 2% TCA (*w/v*) and 2% *i*-Pr_3SiH (*v/v*) in CH_2Cl_2 to the resin (3×10 min) allows for removal of the Mmt group to afford resin **1**. The resin was intensively washed in between all the steps with CH_2Cl_2 (3×1 min) and DMF (3×1 min) and an additional rinse with ether at the end of the synthesis. The resin was kept overnight under vacuum followed by loading determination by measuring the absorbance of the resulting fulvene–piperidine adduct at λ = 301 nm.[6] The final loading was 0.3 mmol/g.

4.1. Stability of resins towards acid conditions

The stability of the resin towards acidic conditions was evaluated for Fmoc-RAM-MBHA (Table 1, entry A– E) and Fmoc-Ala-Wang (Table 1, entry F– I) core resins coupled with Fmoc-Hle by determining the loading by a colorimetric method[6] before and after treatment with different acid conditions. Stability of resins towards standard concentration used in acid wash in AGA (Table 1, entry A and F), half of standard acid concentration (Table 1, entry B and G), glycosylation condition (entry C) and reported conditions for removal of Mmt group (Table 1, entry D, E, H and I) was studied.

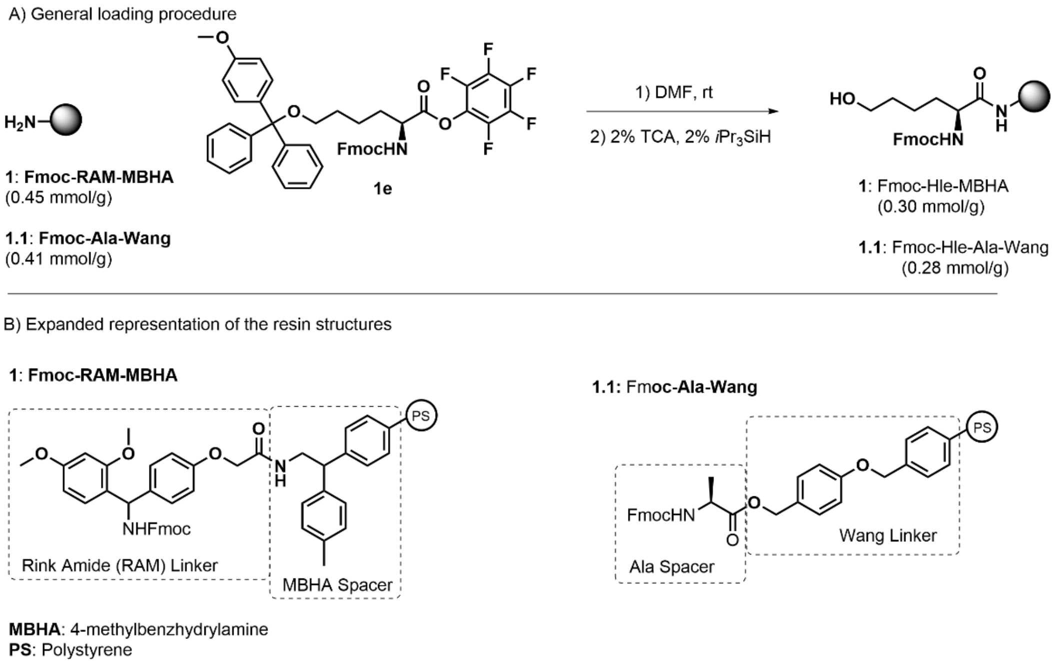

Table 1. Stability of MBHA and Wang resin cores towards acid conditions

Entry	Core resin	Acid conditions*	Initial loading (mmol/g)	Final loading (mmol/g)**	%
**A**	MBHA	1% TMSOTf	0.30	0.26	86
**B**	MBHA	0.5% TMSOTf	0.30	0.27	91
**C**	MBHA	0.15% TfOH	0.30	0.29	97
**D**	MBHA	2% TFA	0.30	0.28	95
**E**	MBHA	2% TCA	0.30	0.30	99
**F**	Wang	1% TMSOTf	0.28	0.09	32
**G**	Wang	0.5% TMSOTf	0.28	012	43
**H**	Wang	2% TFA	0.28	0.21	75
**I**	Wang	2% TCA	0.28	0.23	82

* All solutions were in CH_2Cl_2.

** Results after three cycles of acid wash (Module I) or glycosylation (Module II) in AGA.

5. Solid-phase synthesis of glycolipids

Assembly of glycosyl amphiphiles is carried out by automated glycan assembly followed by manual solid phase peptide synthesis. Synthesis of resin-bound glycan is performed using a home-built synthesizer developed at the Max Planck Institute of Colloids and Interfaces.[1] After completion, the solid support is placed into a manual setup to incorporate the corresponding linker and lipophilic moiety using peptide chemistry.

5.1. Automated Glycan Assembly

5.1.1. Materials and conditions for Automated Glycan Assembly

Solvents used for dissolving building blocks, activator and acid wash solutions were taken from an anhydrous solvent system (JC Meyer). Other solvents used were HPLC grade. The building blocks were co-evaporated three times with toluene and dried for 2 h under high vacuum before use. All solutions were freshly prepared and kept under argon during the run. All syntheses were performed on 0.015 mmol scale using Fmoc-Hle-MBHA resin **1** (50 mg, 0.30 mmol/g). Before starting the run in the machine, all reagent lines were washed and primed.

5.1.1.1.Stock solutions

Building Block solution: 0.2 mmol of thioglycoside building block was dissolved in 1 mL (per glycosylation cycle) of anhydrous CH_2Cl_2.

Acidic wash solution: TCA (0.8 g, 0.4 mmol) was added to 40 mL of anhydrous CH_2Cl_2.

Activator recrystallized NIS (1.55 g, 6.0 mmol) dissolved in CH_2Cl_2/Diox (45 mL, 2:1, *v/v*) in the presence of triflic acid (64 μL, 0.6 mmol). The solution is kept under ice-bath cooling for the duration of the automated run.

Fmoc removal solution: Et_3N (20 mL) was added to 80 mL anhydrous DMF.

5.1.1.2. Modules of Automated Glycan Assembly

The automated synthesis consisted of an initiation step, then, the iterative execution of acidic wash (**Module I**), thioglycoside glycosylation **(Module II)** and temporary protecting groups removal processes (**Module II)** and termination as the final step. The experimental conditions for each of these processes are described as follows.

Initiation: The resin is placed in the reaction vessel and washed with DMF, THF, and CH_2Cl_2 (3×3mL for 15 s, respectively). Next, the resin is swollen in CH_2Cl_2 (2 mL) for 20 minutes while

the temperature of the reaction vessel is cooled to the lowest temperature required throughout the synthesis.

Module I - *Acidic wash*: The resin is placed in the reaction vessel and washed with DMF, THF, and CH_2Cl_2 (3×3mL for 15 s, respectively). Next, the resin is swollen in CH_2Cl_2 (2 mL) for 20 minutes while the temperature of the reaction vessel is cooled to the lowest temperature required throughout the synthesis.

Module II - *Glycosylation with thioglycoside*: The **building block solution** is delivered to the reaction vessel. After the set temperature (T_1) is reached, the reaction is started by dropwise addition of the **activator solution** (1 mL, 0.15 mmol). The glycosylation mixture is incubated for t_1 min at T_1, then the reaction temperature is ramped to T_2 and the mixture is incubated for an additional t_2 min. After completion, the solution is drained and the resin is washed with CH_2Cl_2, CH_2Cl_2 / Diox (1:2, *v/v*, 3 mL for 20 s), and CH_2Cl_2 (2×2 mL each for 25 s). The temperature of the reaction vessel is increased to 25°C for the next module.

Table 2. Conditions for glycosylation with thioglycosides in AGA (Module II)

Equivalents	Repeat	Module II	Notes
**6.5**	1x	II-A	-20 °C (T_1) 20 min (t_1)
			0 °C (T_2) 10 min (t_2)
**6.5**	2x	II-B	-30 °C (T_1) 20 min (t_1)
			-10 °C (T_2) 10 min (t_2)

Module III - *Fmoc deprotection:* The resin is washed with DMF (3×2 mL for 25 s) and the temperature of the reaction vessel is adjusted to 25 °C. **Fmoc removal solution** is delivered to the reaction vessel and kept under argon bubbling. After 5 min, the reaction solution is drained and the resin is washed with DMF (3×2 mL for 25 s) and CH_2Cl_2 (5×2 mL for 25 s). The temperature of the reaction vessel decreased to -20 °C for the next module.

Termination: The temperature of the reactor is adjusted to 25 °C and the resin is washed with DMF, THF, and CH_2Cl_2 (3×3 mL for 15 s respectively). At the end, the resin is kept suspended in CH_2Cl_2 (4 mL).

5.1.2. Post-Automated Glycan Assembly monitoring

Micro-cleavage (Method B): Few resin beads are taken and treated with the cleavage cocktail 30% TFA/2% *i*-Pr_3SiH/CH_2Cl_2 (0.5 mL, *v/v/v*) and the mixture is shaken for 1 h. The solvents are co-evaporated with toluene under a N_2 stream and the resulting crude is dissolved in MeCN (20 µL) for MALDI analysis. Later, the solvent is evaporated under a N_2 stream and the crude is re-dissolved in Hex/EtOAc (20 µL, 1:1, *v/v*) for NP-HPLC analysis.

Analytical NP-HPLC (Method A-3*):* NP-HPLC was conducted on an Agilent 1200 Series system. A YMC-Diol-300-NP column (150 mm x 4.600 mm I.D.) was used with a flow rate of 1.00 mL/min and Hex/EtOAc as eluent [isocratic 10% EtOAc (5 min), linear gradient to 60% EtOAc (45 minutes)].

5.1.3. Glycan assembly of synthetic glycolipids

*α-(1→2)-Mannose trimer (**12a**)*

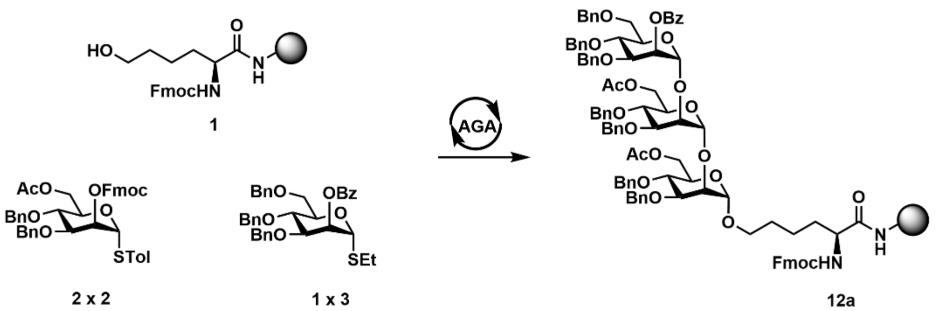

Resin-bound oligosaccharide **12a** was obtained by AGA (Method 1). BB **2** was incorporated two times (Module II-A) followed by incorporation of BB **3** (Module II-B). After synthesis completion, the resulting crude from micro-cleavage (Method B) was analyzed by NP-HPLC (Method A-3), R_t = 41.1 min. and MALDI-MS. MALDI-MS *m/z:* 1696.7 $[M+Na]^+$, calcd. for $C_{99}H_{104}N_2NaO_{22}$: 1696.7. After assembly of the glycan, the resin is ready for subsequent SPPS (Method 2).

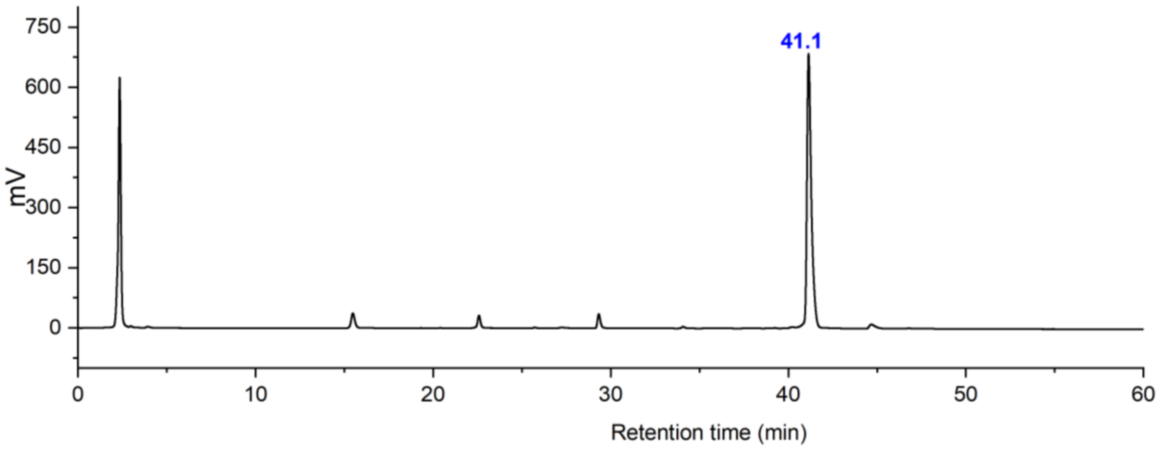

Figure 80. NP-HPLC trace of crude **12a** after micro-cleavage.

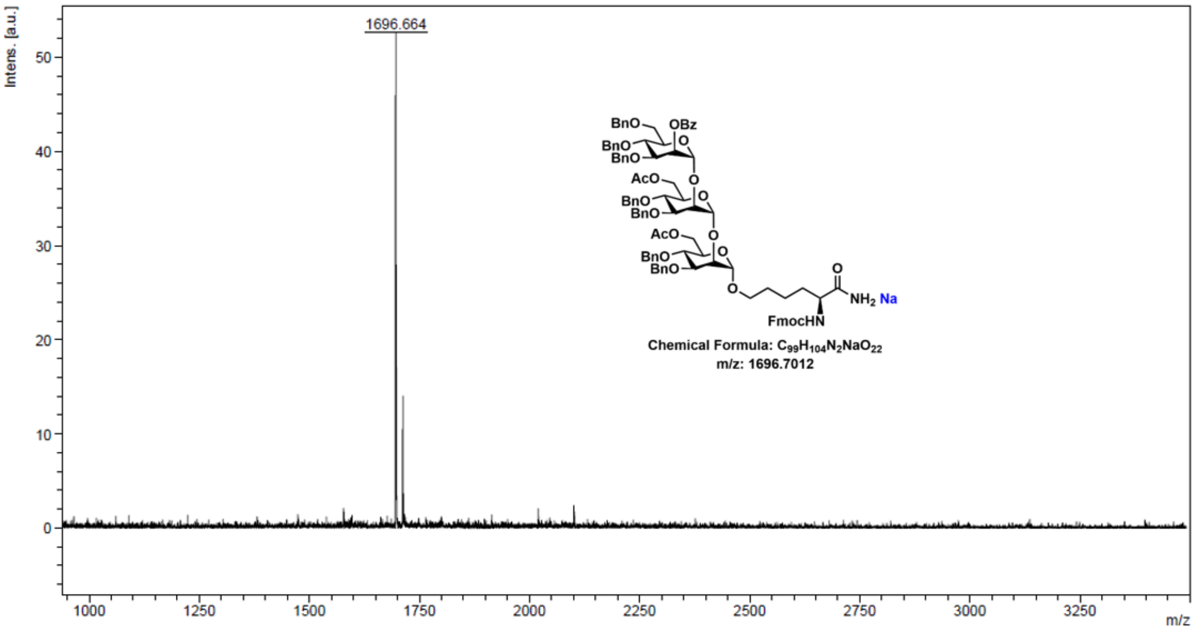

Figure 81. MALDI-MS of oligosaccharide **12a** after micro-cleavage.

α-(1→2)-α-(1→3)-Mannose trimer ***(14a)***

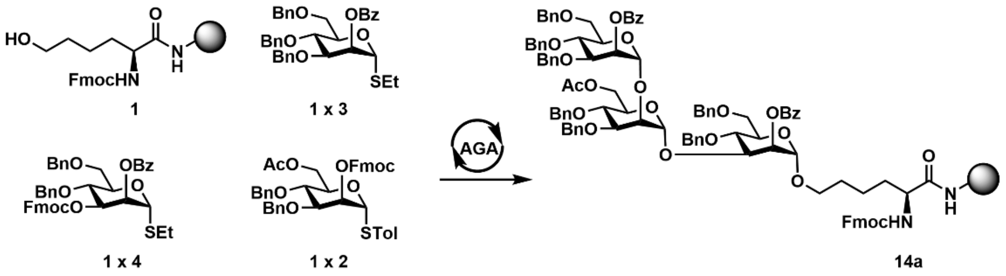

Resin-bound oligosaccharide **14a** was obtained by AGA (Method 1). Each BB **4**, **2** (Module II-A) and **3** (Module II-B) respectively was incorporated one time After synthesis completion, the resulting crude from micro-cleavage (Method B) was analyzed by NP-HPLC (Method A-3), R_t = 40.4 min. and MALDI-MS. MALDI-MS *m/z:* 1758.7 $[M+Na]^+$, calcd. for $C_{104}H_{106}N_2NaO_{22}$: 1758.7. After glycan assembly, the resin is ready for subsequent SPPS (Method 2).

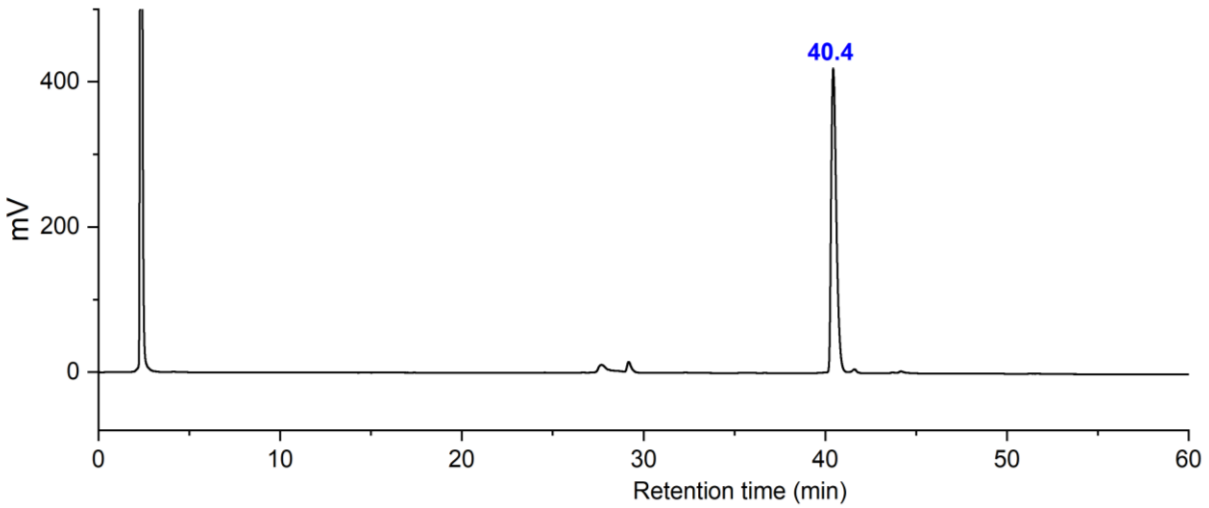

Figure 82. NP-HPLC trace of crude **14a** after micro-cleavage.

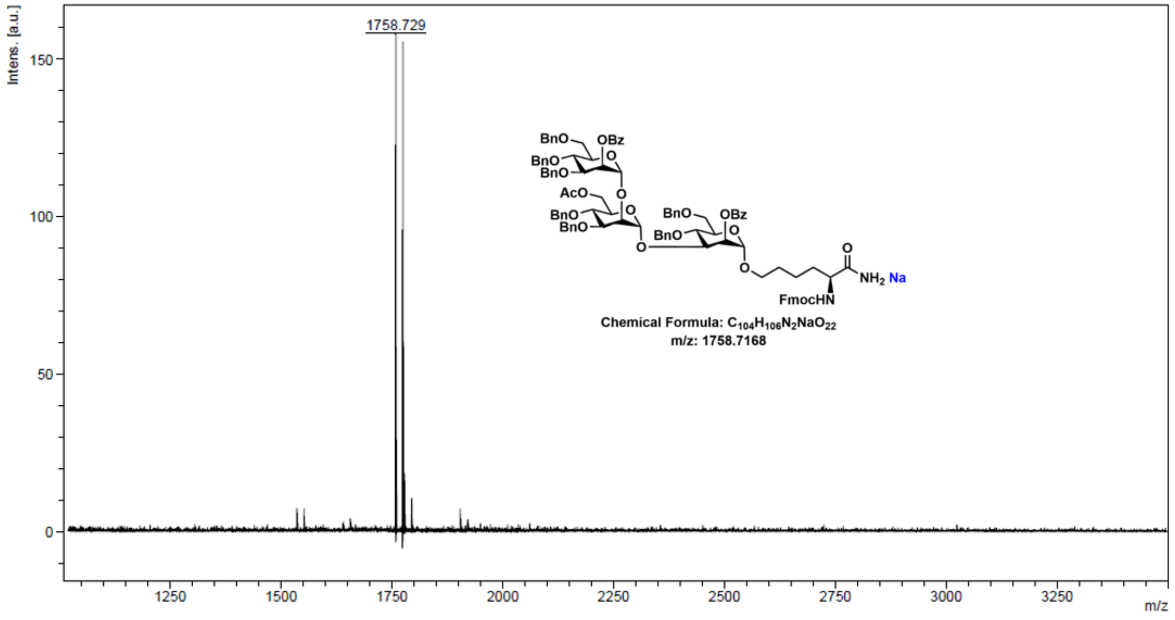

Figure 83. MALDI-MS of oligosaccharide **14a** after micro-cleavage.

*Globotriose (Gb3) (**16a**)*

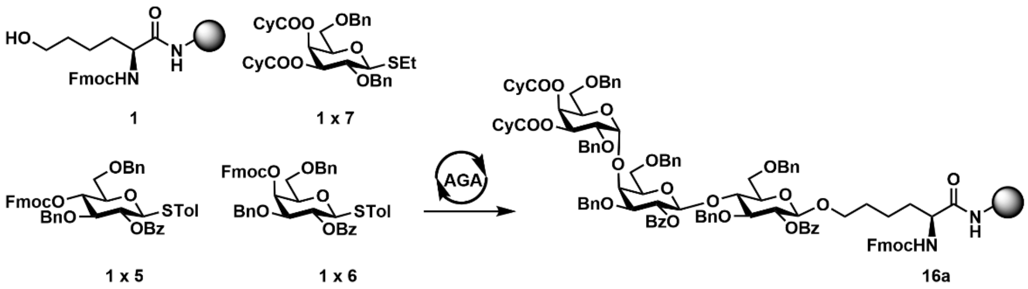

Resin-bound oligosaccharide **16a** was obtained by AGA (Method 1). Each BB (**5**, **6** and **7** respectively) was incorporated one time (Module II-A). After synthesis completion, the resulting crude from micro-cleavage (Method B) was analyzed by NP-HPLC (Method A-3), R_t = 46.0 min. and MALDI-MS. MALDI-MS *m/z:* 1846.9 $[M+Na]^+$, calcd. for $C_{109}H_{118}N_2NaO_{23}$: 1846.8. After glycan assembly, the resin is ready for subsequent SPPS (Method 2).

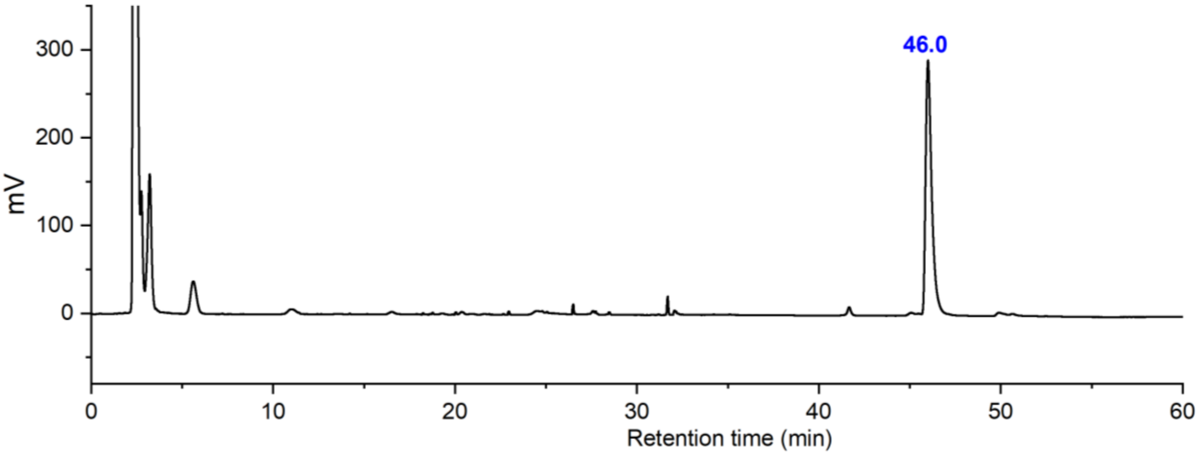

Figure 84. NP-HPLC trace of crude **16a** after micro-cleavage.

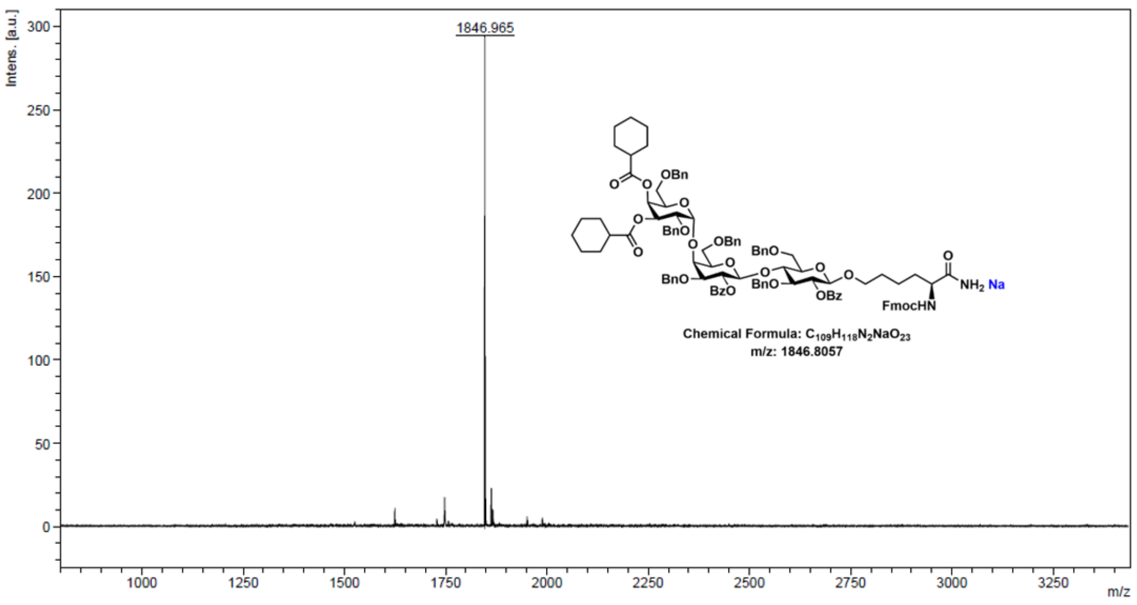

Figure 85. MALDI-MS of oligosaccharide **16a** after micro-cleavage.

5.2. Solid Phase Peptide Synthesis

5.2.1. Materials and conditions for Solid Phase Peptide Synthesis

Assembly of the corresponding spacer and hydrophobic moieties was carried out manually following the Fmoc/tBu strategy. Synthesis is performed on glycan-bound resin (0.3 mmol/g) at 0.015 mmol scale. First, the resin is swollen for 20 min in CH_2Cl_2 followed by iterative processes of *Fmoc removal* and *PyBOP-based coupling* **(Method 2)** as described below.

Fmoc removal: The resin is treated with a solution of 20% piperidine in DMF (2×10 min), then washed with DMF (3×1 min) and CH_2Cl_2 (3×1 min).

PyBOP-based coupling: Fmoc protected amino acid (5 equiv) and PyBOP (5 equiv) are dissolved in DMF, then NMM (10 equiv) is added. The mixture is pre-activated for 4 min, added to the resin, and stirred at room temperature for 90 min. The resin is washed with DMF (3×1 min) and CH_2Cl_2 (3×1 min).

5.2.2. Post-solid phase manipulations

After linear on-resin assembly, the resin-bound glycolipopeptides were subjected to the sequential procedure of 1) *on-resin methanolysis*, 2) *acidic cleavage* and 3) *hydrogenolysis* to remove the protecting groups and to achieve cleavage from the solid support, and 4) *purification* as described below.

On-resin methanolysis: The glycolipid-bound resin was mixed with anhydrous THF (4.0 mL). A solution of NaOMe in MeOH (0.5 M, 0.4 mL) was added and the mixture was shaken at room temperature for 16 h. The reaction mixture was filtered and the resin was washed with THF (3×1 min) and CH_2Cl_2 (2×1 min).

Acidic cleavage: The resin is treated with the cocktail TFA/*i*-Pr_3SiH/ CH_2Cl_2 (30% TFA, 2% *i*-Pr_3SiH, *v/v*) and the mixture is shaken for 2×3 mL for 1 h. The solutions were combined, the solvents are co-evaporated with toluene and the resulting crude is dissolved in the corresponding solvent mixture for the next step.

Hydrogenolysis: The crude compound is dissolved in a solvent mixture (see specific conditions for each compound) until complete dissolution up to 3 mL final volume. $Pd(OH)_2/C$ (0.1 g) was added and the reaction was stirred under H_2 atmosphere overnight. The mixture was filtered through Rotilabo syringe filters (Roth), PTFE filters (pore size: 0.45 µm) and washed with the corresponding solvents. The filtrates were concentrated under reduced pressure and lyophilized.

5.2.2.1. Analytical RP-HPLC and purification

Method A-4: Crude product was dissolved in MeOH/H_2O (4:1, *v/v*) and analyzed/purified using analytical/semi-preparative RP-HPLC Agilent 1200 Series. A Phenomenex, luna C5 column (250 x 4.6 mm, 5 µm) flow rate of 1.0 mL /min with MeCN/H_2O (0.1% formic acid) as eluents [isocratic 30% MeCN (5 min), linear gradient to 100% MeCN (30 min)] was used for analytical and a Phenomenex, Luna C5 column (250 x 10 mm, 5 µm), flow rate of 4.0 mL /min with MeCN /H_2O (0.1% formic acid) as eluents [isocratic 30% MeCN (5 min), linear gradient to 100% MeCN (30 min)] as semi-preparative conditions.

Method A-5: Crude product was dissolved in *i*PrOH/H_2O (2:1, *v/v*) and analyzed/purified using analytical/semi-preparative RP-HPLC Agilent 1200 Series. A Phenomenex, Luna C5 column (250 x 4.6 mm, 5 µm) flow rate of 1.0 mL /min with MeCN/H_2O (0.1% formic acid) as eluents [isocratic 50% MeCN (5 min), linear gradient to 100% MeCN (30 min)] at 40 –50 °C was used for analytical and a Phenomenex, Luna C5 column (250 x 10 mm, 5 µm), flow rate of 4.0 mL /min with MeCN/H_2O (0.1% formic acid) as eluents [isocratic 50% MeCN (5 min), linear gradient to 100% MeCN (30 min)] at 40 –50 °C as semi-preparative conditions.

5.2.3. Constructing spacer and glycolipid anchor

*Pam2-PEG- α-(1→2)-trimannose (**12**)*

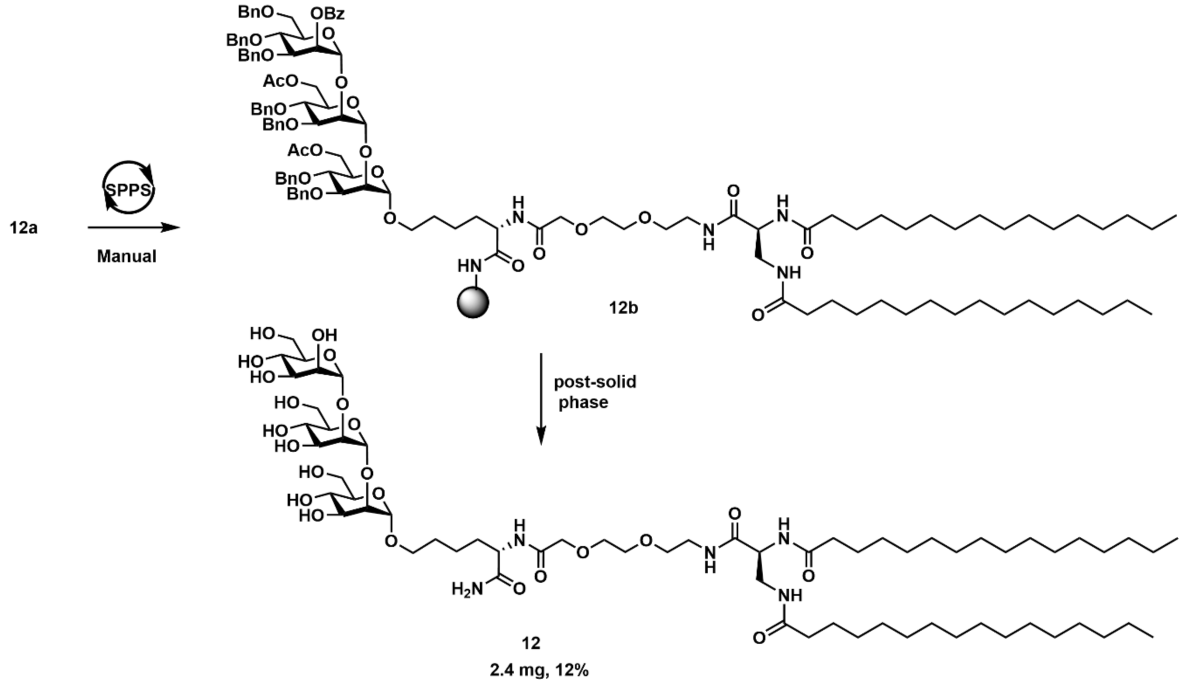

Glycolipid **12** (2.4 mg, 12%) was synthesized as a white solid from resin bound oligosaccharide **12a** (0.015 mmol) using stepwise SPPS (Method 2), followed by post-solid phase manipulation [hydrogenolysis (THF/tBuOH/H_2O, 2:1:1, *v/v*)] and purification by semi-preparative RP-HPLC (Method A-5). R_t = 20.9 min. HR-MS (QTOF) m/z = 1340.8468 $[M+H]^+$, calcd. for $C_{44}H_{122}N_5O_{21}$: 1340.8525. ^{1}H NMR (700 MHz, $CDCl_3$/ MeOD) δ 4.97 (d, *J* = 8.5 Hz, 1H), 4.68 (t, *J* = 5.8 Hz, 1H), 3.66 (t, *J* = 4.6 Hz, 1H), 3.52 (t, *J* = 4.7 Hz, 3H), 3.47 – 3.41 (m, 2H), 3.42 – 3.33 (m, 4H), 3.31 (q, *J* = 5.4 Hz, 1H), 3.28 – 3.16 (m, 3H), 3.16 (d, *J* = 5.5 Hz, 1H), 3.34 – 3.12 (m, 3H), 3.15 (s, 2H), 3.11 (s, 1H), 3.09 (s, 1H), 3.06 (s, 2H), 2.95 (s, 1H), 1.99 (d, *J* = 7.5 Hz, 1H), 1.94 – 1.89 (m, 1H), 1.31 (p, *J* = 7.7 Hz, 4H), 1.07 – 0.92 (m, 48H), 0.59 (q, *J* = 6.7 Hz, 6H). ^{13}C NMR (176 MHz, $CDCl_3$/MeOD) δ 177.31, 174.91, 174.15, 170.57, 170.38, 102.32, 100.74, 98.35, 73.36, 73.19, 72.74, 71.24, 71.14, 71.04, 70.89, 70.53, 70.32, 69.85, 69.75, 67.90, 67.71, 66.55, 62.12, 61.73, 49.08, 48.96, 48.91, 48.84, 48.79, 48.67, 48.55, 48.43, 48.31, 48.18, 41.04, 39.14, 36.26, 34.42, 33.56, 31.83, 31.04, 29.61, 29.56, 29.54, 29.50, 29.47, 29.39, 29.33, 29.26, 29.22, 29.14, 29.12, 28.48, 25.73, 25.54, 25.01, 22.56, 13.80.

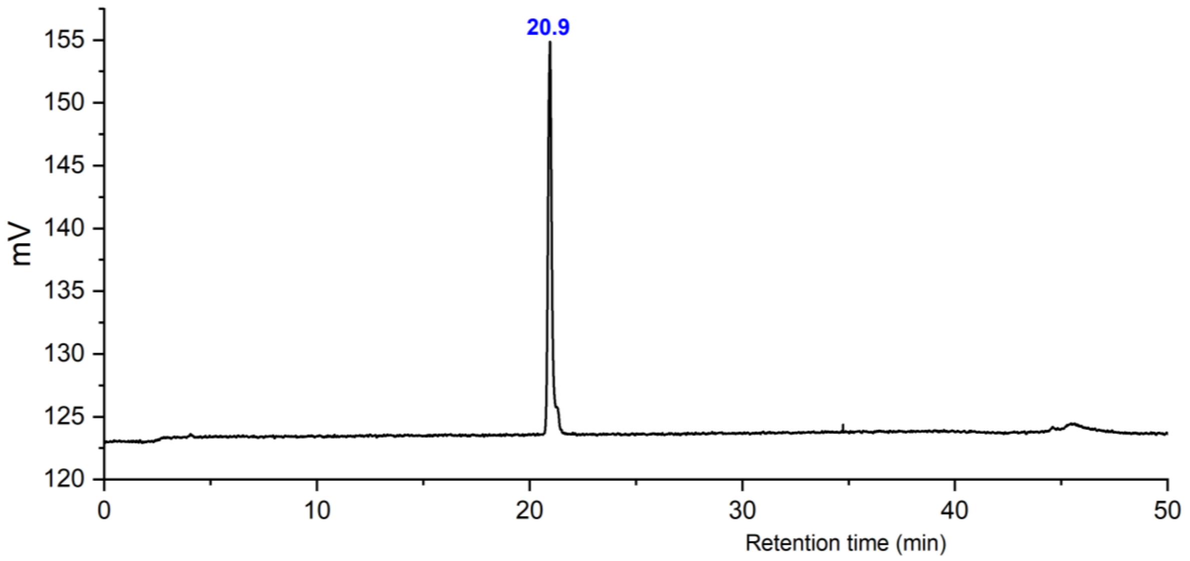

Figure 86. RP-HPLC trace of compound **12.**

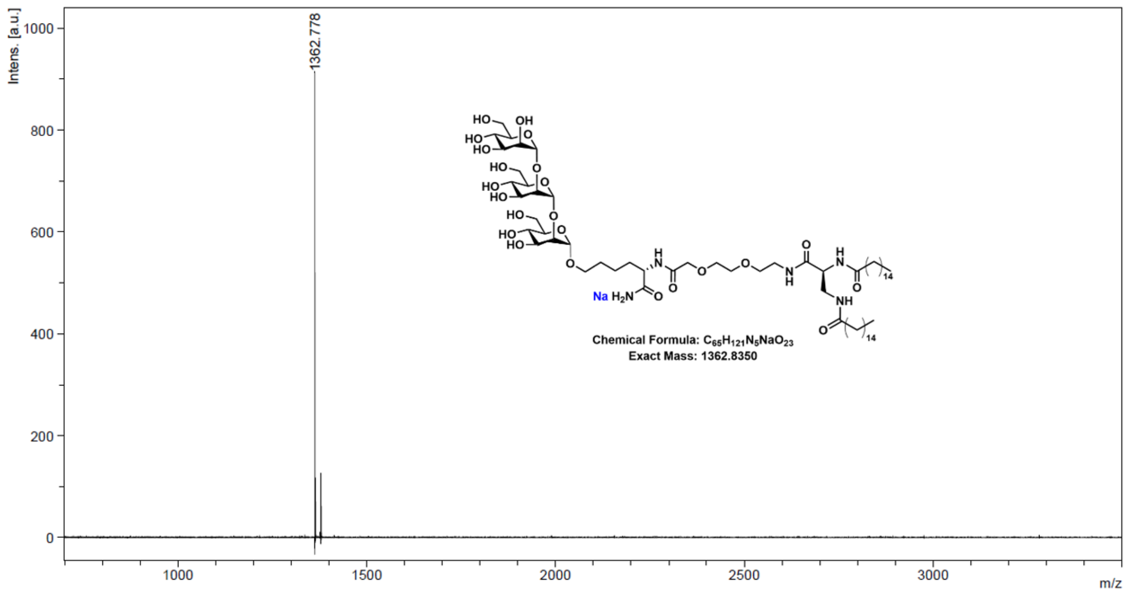

Figure 87. MALDI-MS of compound **12**.

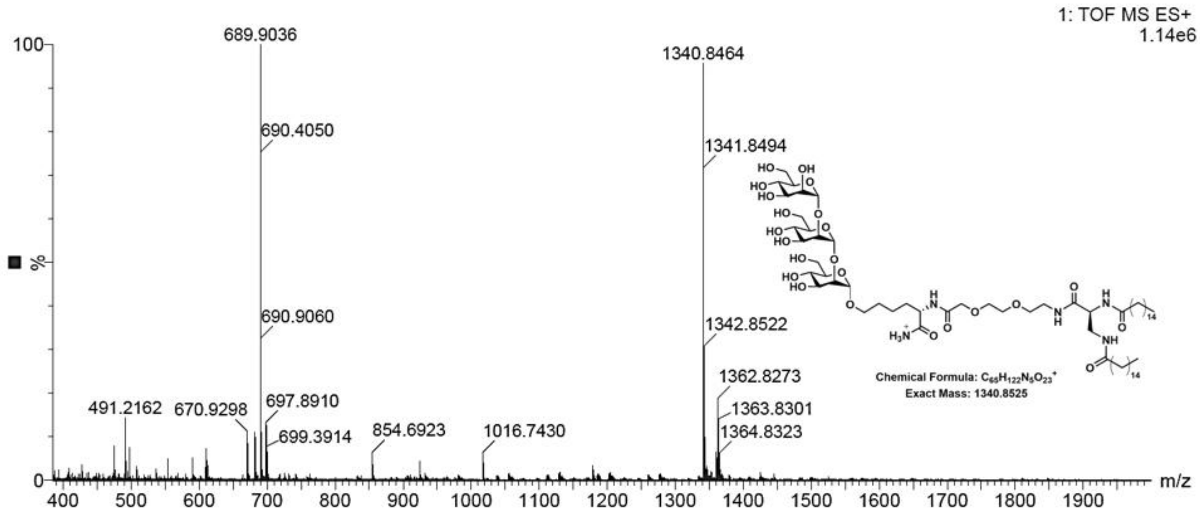

Figure 88. HR-MS of compound **12.**

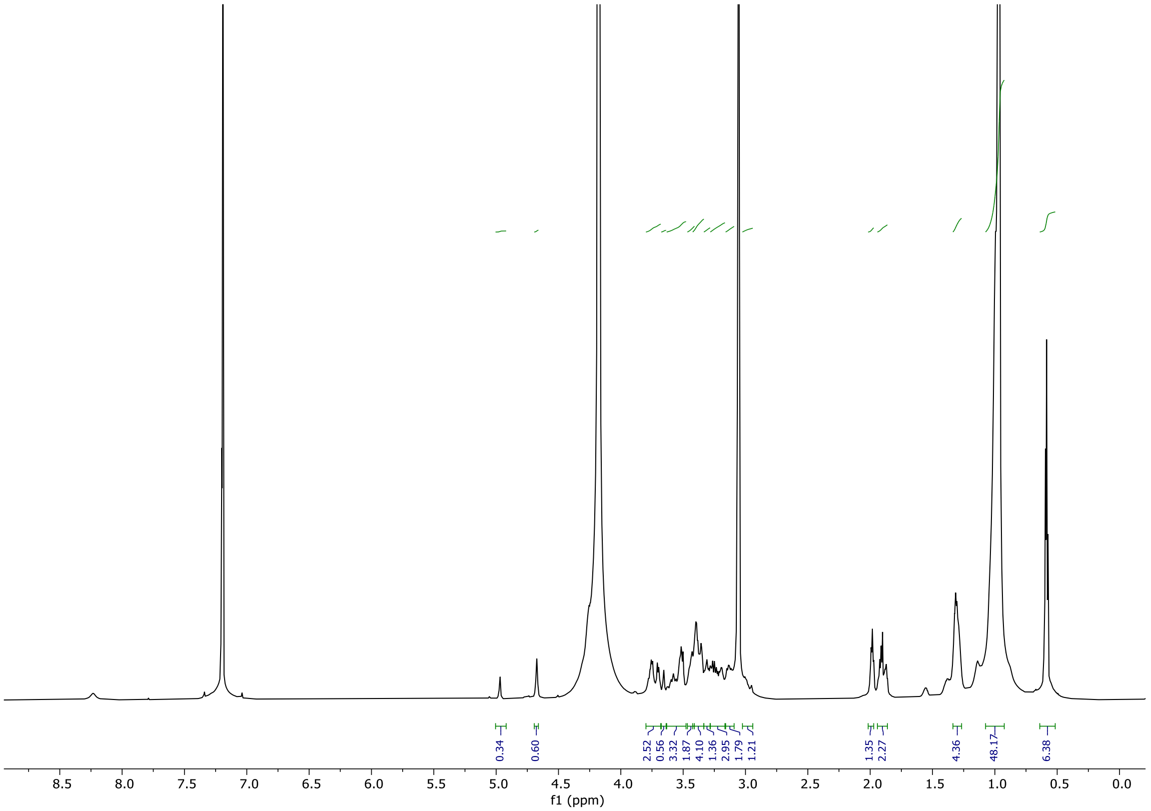

Figure 89. ^{1}H NMR (700 MHz, $CDCl_3$/MeOD, 2:1, *v/v*) spectrum of **12.**

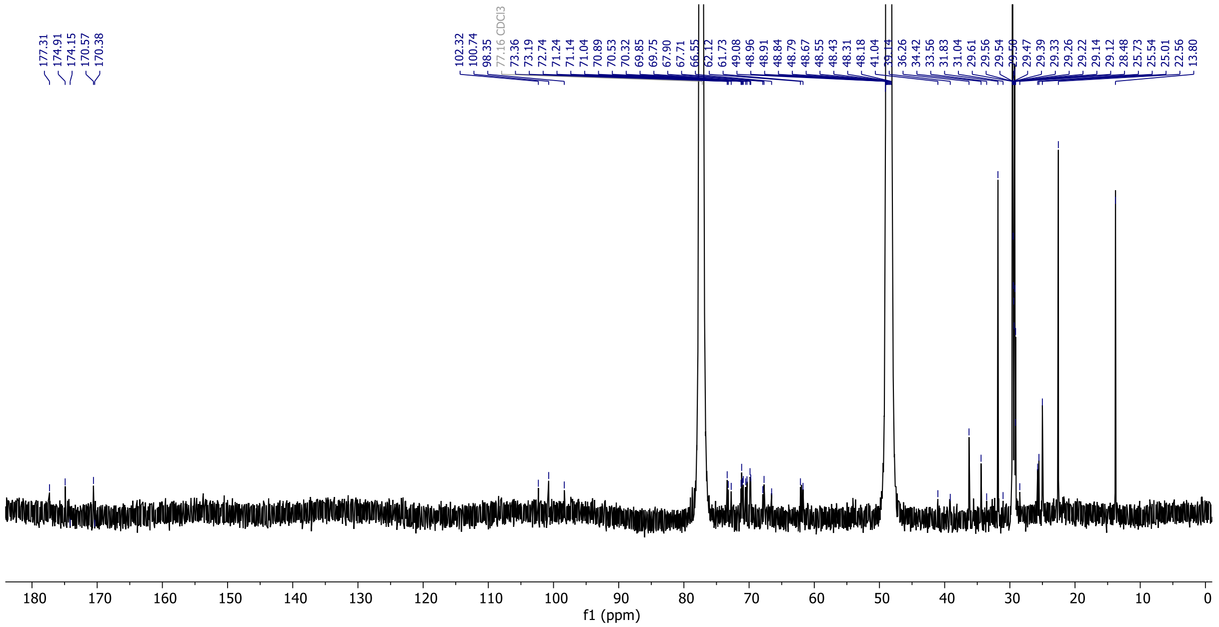

Figure 90. ^{13}C NMR (176 MHz, $CDCl_3$/MeOD, 2:1, *v/v*) spectrum of **12.**

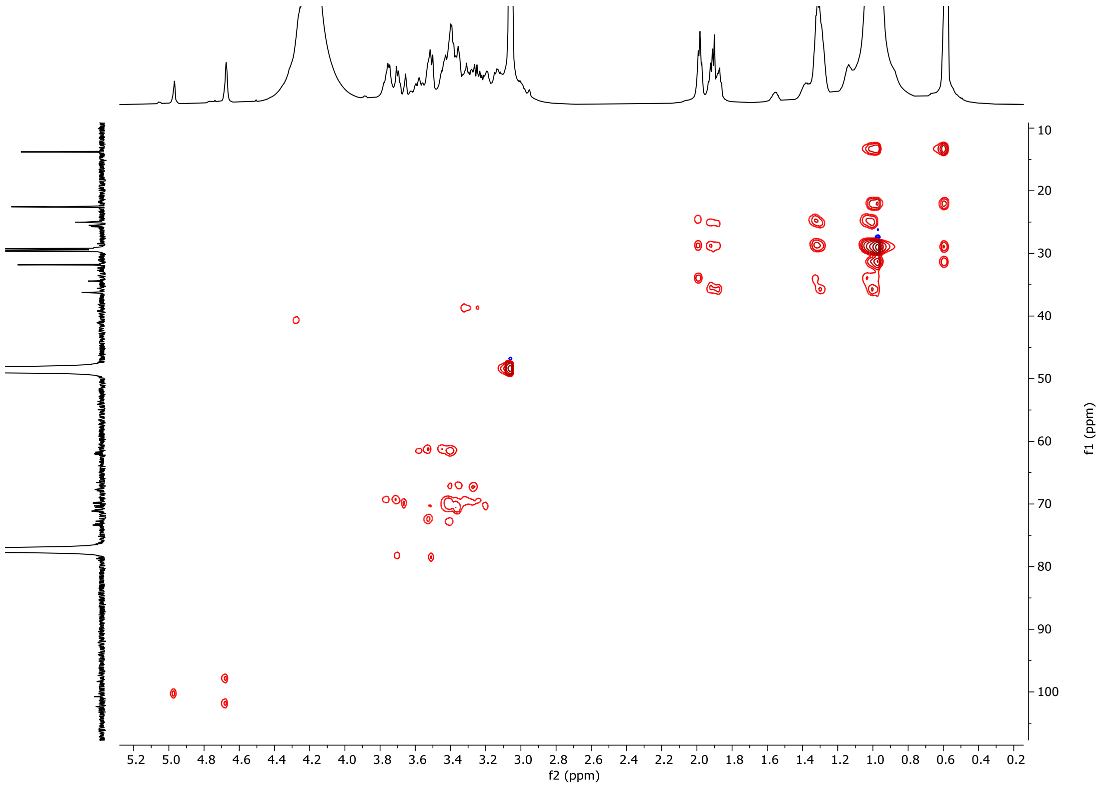

Figure 91. HSQC NMR (700 MHz, $CDCl_3$/MeOD, 2:1, *v/v*) spectrum of **12.**

*Cho-PEG$_2$- α-(1→2)-trimannose (**13**)*

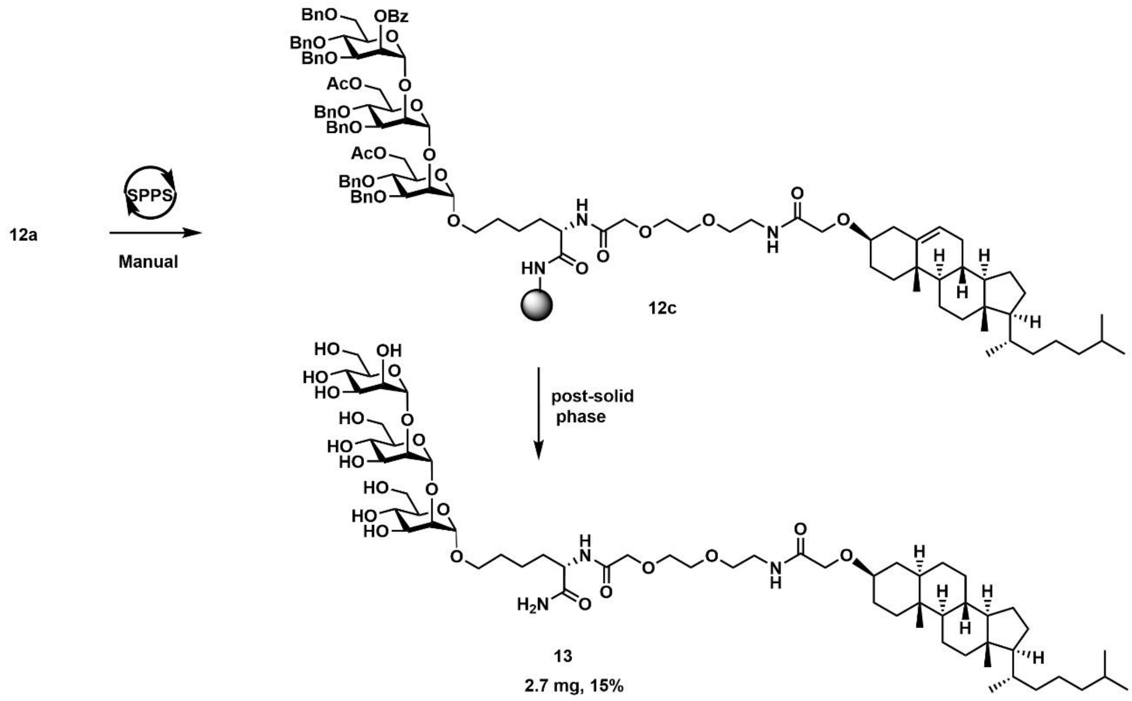

Glycolipid **13** (2.7 mg, 15%) was synthesized as a white solid from resin bound oligosaccharide **12a** (0.015 mmol) using stepwise SPPS (Method 2), followed by post-solid phase manipulation [hydrogenolysis (THF/tBuOH, 2:1, *v/v*)] and purification by semi-preparative RP-HPLC (Method A-4). R_t = 24.0 min. HR-MS (QTOF) m/z = 1206.7283 $[M+H]^+$, calcd. for $C_{59}H_{104}N_3O_{22}^+$: 1206.7106. ^{1}H NMR (700 MHz, MeOD/D_2O) δ 6.72 (s, 1H), 5.85 (dd, J = 8.3, 5.6 Hz, 1H), 5.52 (s, 1H), 5.45 (d, J = 5.2 Hz, 2H), 5.33 – 5.31 (m, 1H), 5.30 – 5.27 (m, 2H), 5.19 (d, J = 3.2 Hz, 1H), 5.17 – 5.14 (m, 3H), 5.08 (dt, J = 7.2, 3.6 Hz, 2H), 5.04 (dd, J = 9.7, 1.9 Hz, 2H), 4.97 (dt, J = 6.3, 2.5 Hz, 1H), 4.91 (q, J = 4.8 Hz, 1H), 4.85 (p, J = 1.7 Hz, 1H), 3.41 (d, J = 10.2 Hz, 1H), 3.32 – 3.27 (m, 1H), 3.19 – 3.16 (m, 1H), 3.08 (d, J = 16.1 Hz, 3H), 2.94 (dt, J = 13.0, 6.5 Hz, 2H), 2.77 (d, J = 8.7 Hz, 2H), 2.73 – 2.69 (m, 2H), 2.52 (t, J = 7.8 Hz, 3H), 2.44 – 2.38 (m, 2H), 2.34 (d, J = 6.1 Hz, 2H), 2.30 (dd, J = 6.6, 3.1 Hz, 3H), 2.25 (s, 2H), 2.08 (d, J = 26.4 Hz, 2H).^{13}C NMR (176 MHz, MeOD/D_2O) δ 176.7, 172.7, 171.1, 101.2, 100.0, 99.4, 80.9, 80.3, 79.8, 79.4, 77.2, 74.5, 71.7, 71.3, 70.8, 68.3, 62.5, 62.3, 61.3, 61.1, 59.3, 58.9, 57.8, 57.7, 57.5, 57.4, 57.3, 34.4, 30.8, 29.6, 29.1, 23.8, 17.2, 17.1, 16.9, 16.8, 16.7, 13.3, 12.2.

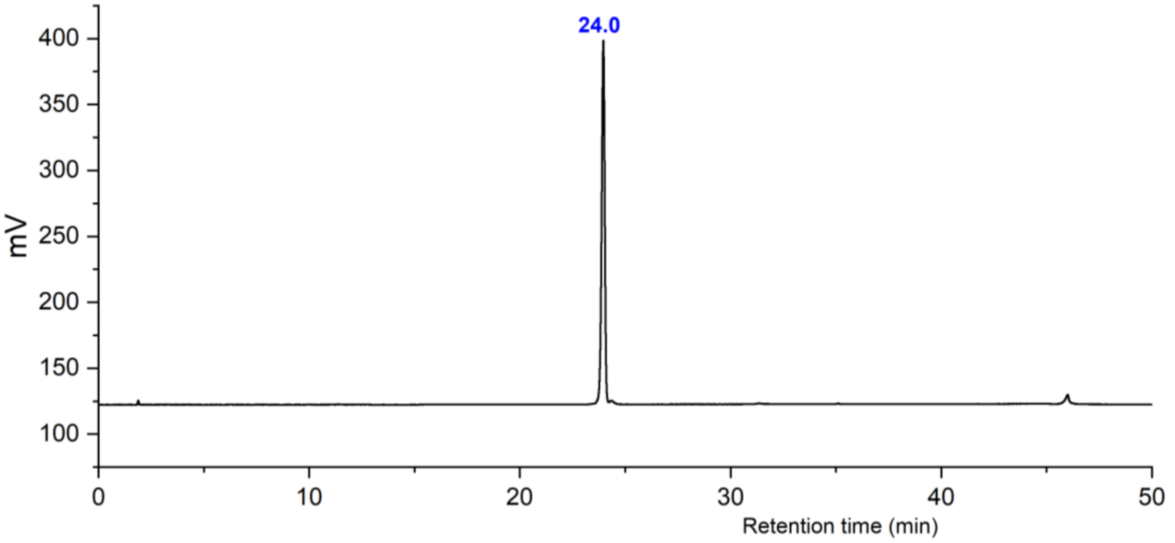

Figure 92. RP-HPLC trace of compound **13.**

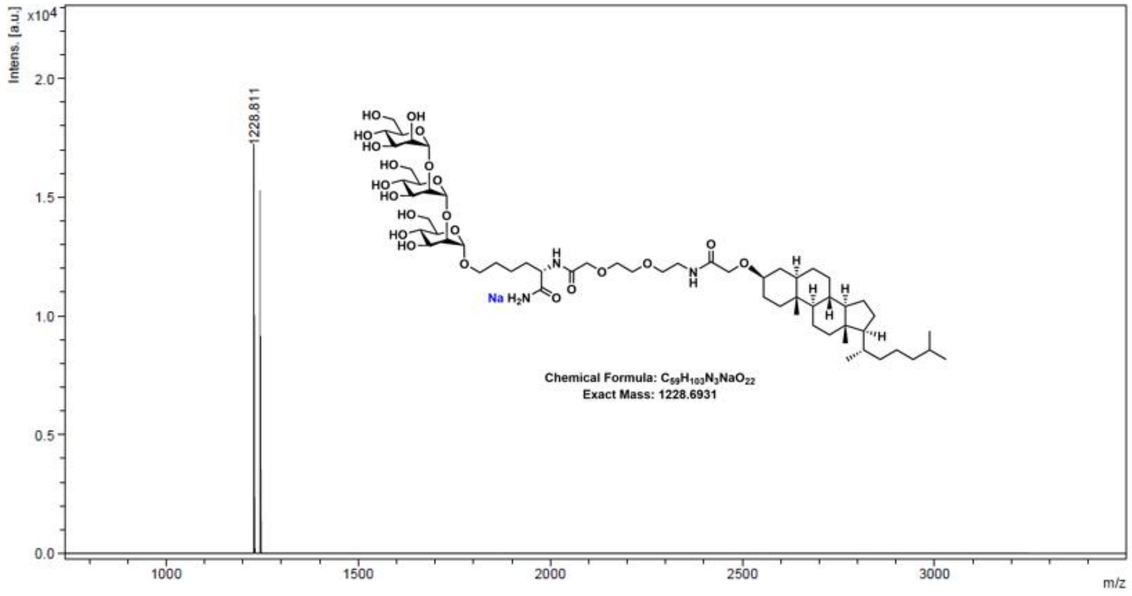

Figure 93. MALDI-MS of compound **13**.

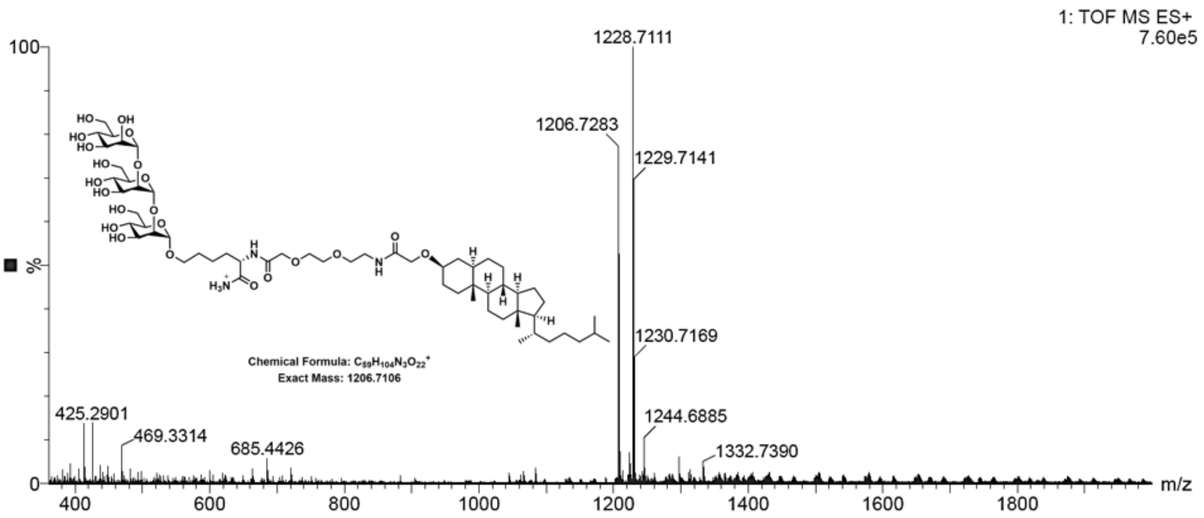

Figure 94. HR-MS of compound **13.**

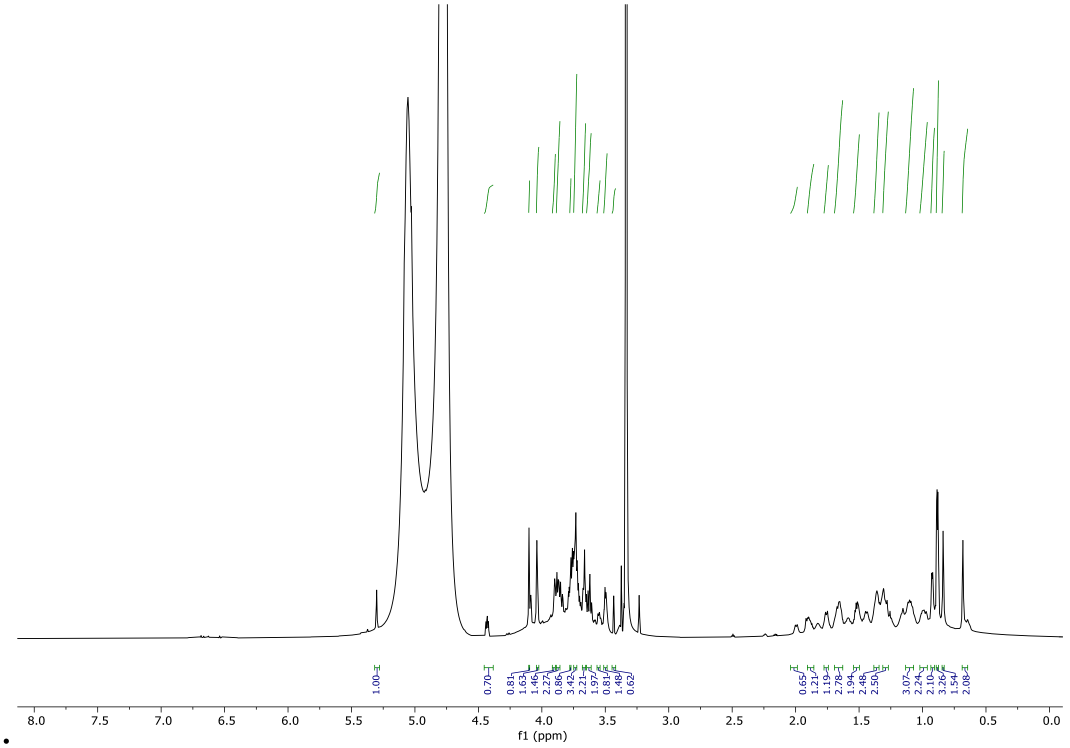

Figure 95. ^{1}H NMR (700 MHz, MeOD/D_2O, 2:1, *v/v*) spectrum of **13.**

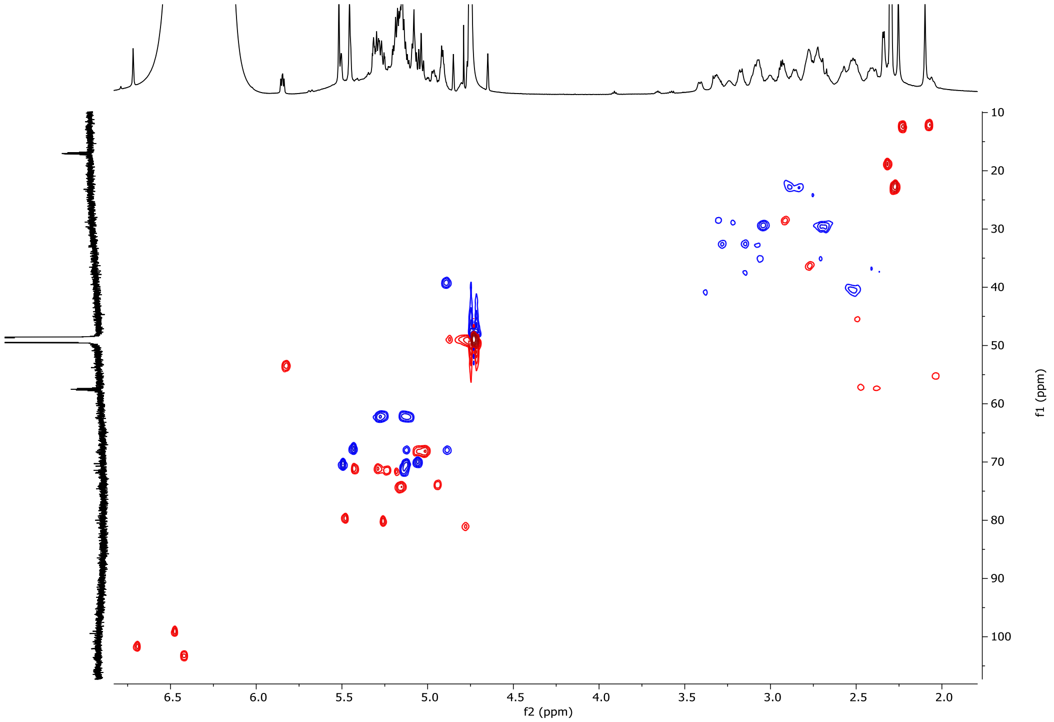

Figure 96. HSQC NMR (176 MHz, MeOD/D_2O, 2:1, *v/v*) spectrum of **13.**

Pam₂-PEG₂- α-(1→2)- α-(1→3)-trimannose ***(14)***

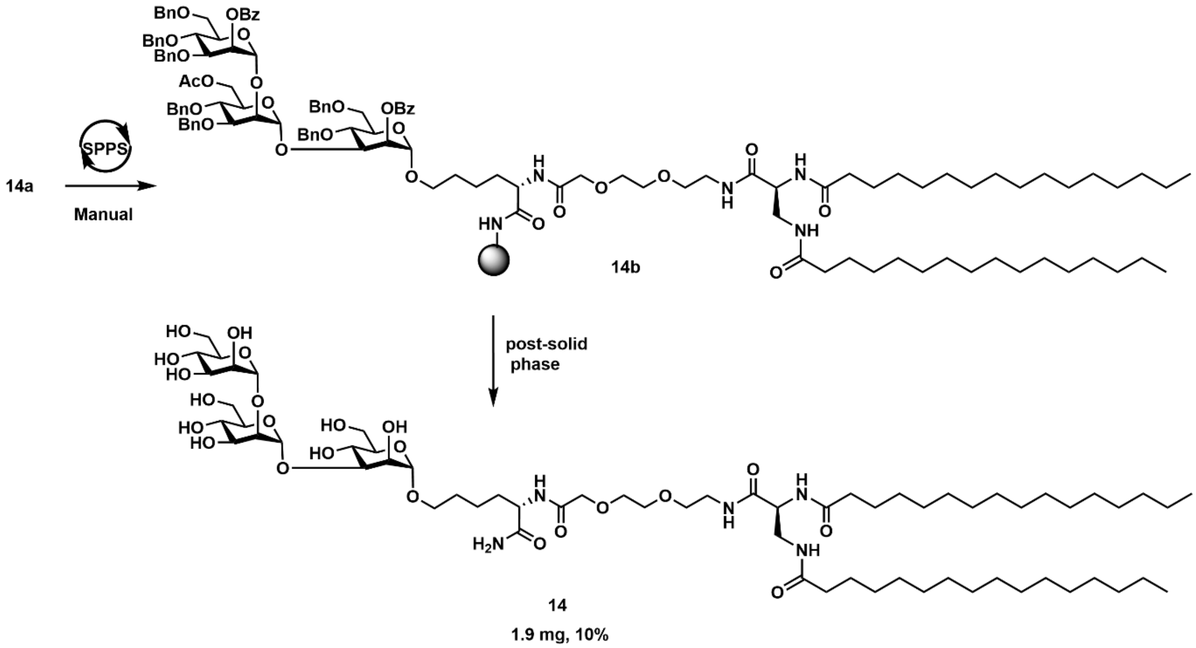

Glycolipid **14** (1.9 mg, 10%) was synthesized as a white solid from resin bound oligosaccharide **14a** (0.015 mmol) using stepwise SPPS (Method 2), followed by post-solid phase manipulation [hydrogenolysis (THF/tBuOH/H_2O, 2:1:1, *v/v*)] and purification by semi-preparative

RP-HPLC (Method A-5). R_t = 18.4 min. HR-MS (QTOF) m/z = 1362.8461 $[M+Na]^+$, calcd. for $C_{65}H_{121}N_5O_{23}Na$: 1362.8350. ^{1}H NMR (700 MHz, $CDCl_3$/ MeOD) δ 5.30 – 5.23 (m, 1H), 4.91 – 4.89 (m, 1H), 4.64 – 4.62 (m, 1H), 4.00 – 3.93 (m, 5H), 3.92 (dq, *J* = 5.4, 2.9 Hz, 2H), 3.87 (tq, *J* = 5.4, 3.0 Hz, 1H), 3.83 – 3.75 (m, 2H), 3.75 – 3.67 (m, 3H), 3.64 – 3.58 (m, 5H), 3.56 (td, *J* = 4.9, 2.3 Hz, 1H), 3.53 – 3.46 (m, 3H), 3.45 – 3.40 (m, 1H), 3.36 – 3.31 (m, 1H), 2.16 – 2.03 (m, 4H), 1.97 – 1.86 (m, 1H), 1.83 – 1.67 (m, 1H), 1.62 – 1.44 (m, 7H), 1.36 (q, *J* = 8.0 Hz, 2H), 1.34 – 1.09 (m, 48H), 0.81 – 0.74 (m, 6H).^{13}C NMR (176 MHz, $CDCl_3$/ MeOD) δ 175.78, 175.39, 172.12, 171.42, 170.08, 130.69, 103.09, 101.20, 100.85, 74.27, 73.85, 71.94, 71.46, 71.14, 70.97, 70.68, 68.45, 67.79, 67.13, 62.83, 62.57, 62.31, 57.52, 57.39, 57.27, 37.09, 36.38, 32.65, 30.43, 30.39, 30.33, 30.31, 30.23, 30.16, 30.09, 29.51, 27.88, 26.56, 26.43, 26.36, 23.39, 23.01, 17.43, 14.63.

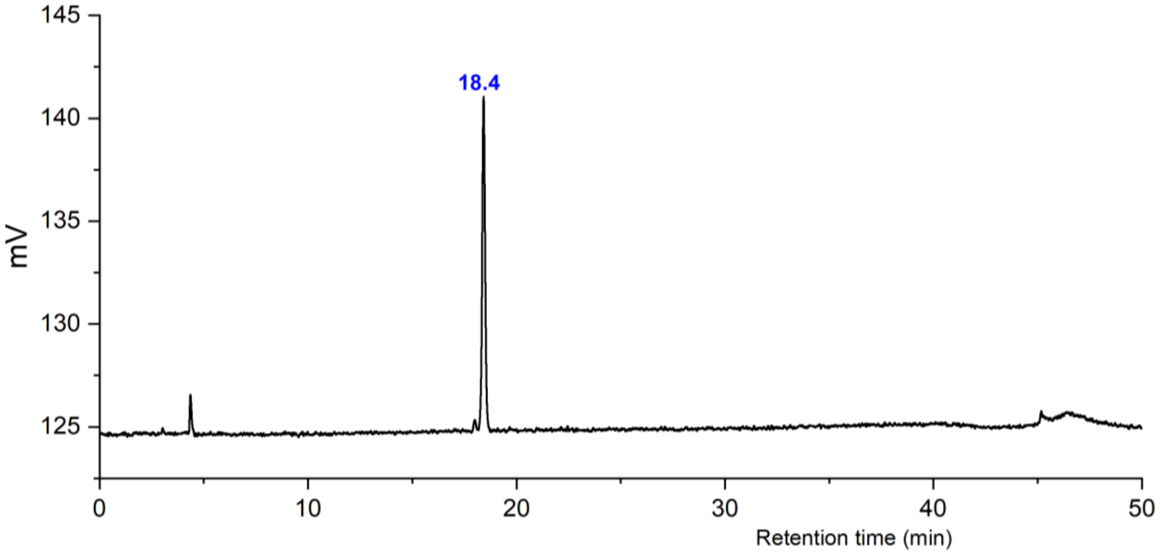

Figure 97. RP-HPLC trace of compound **14.**

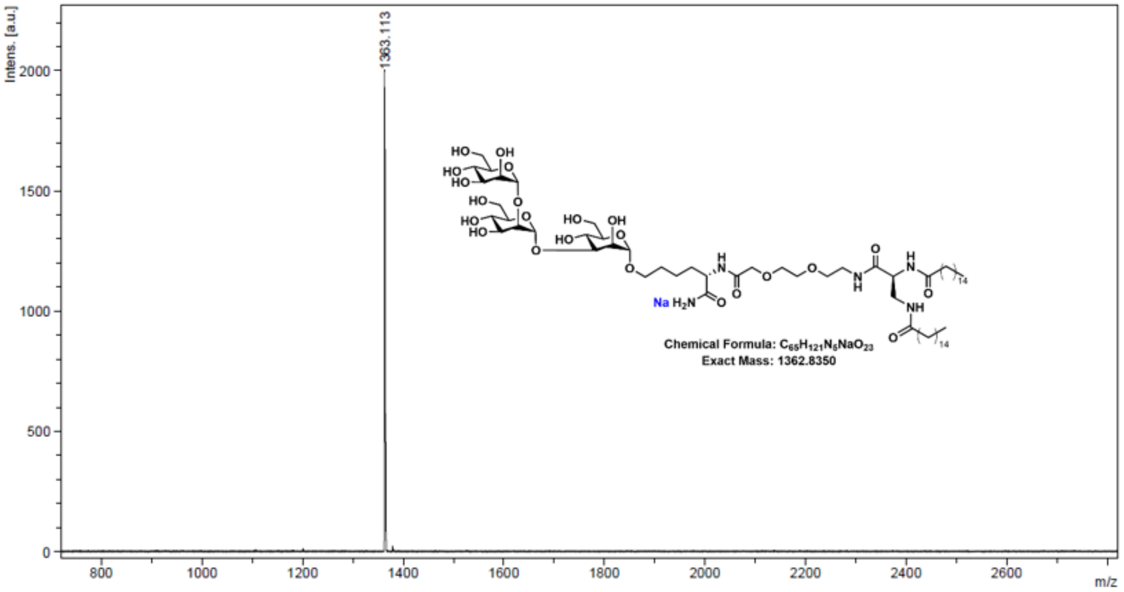

Figure 98. MALDI-MS of compound **14**.

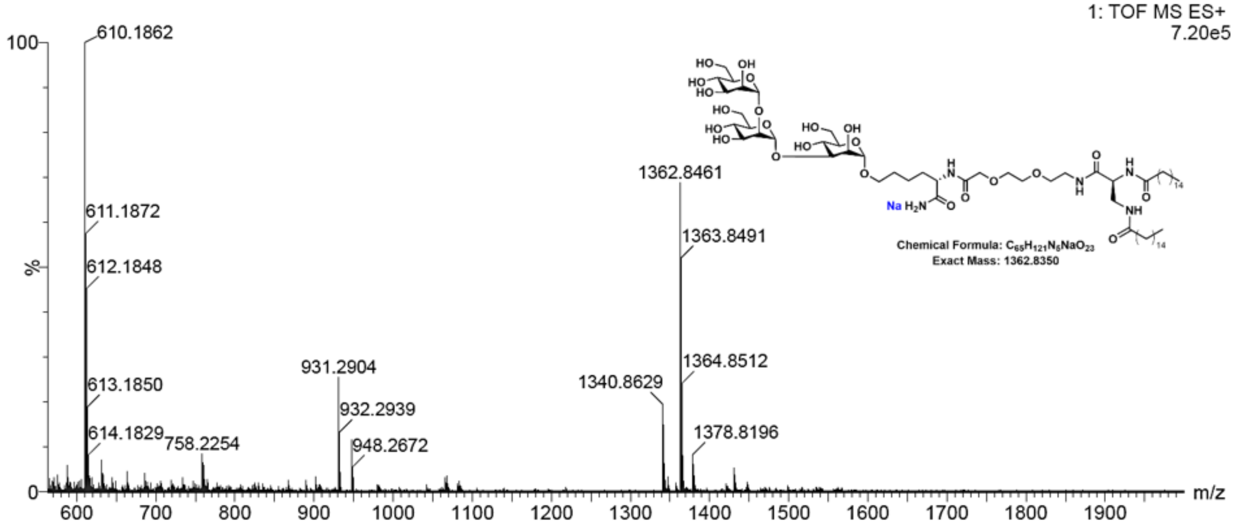

Figure 99. HR-MS of compound **14.**

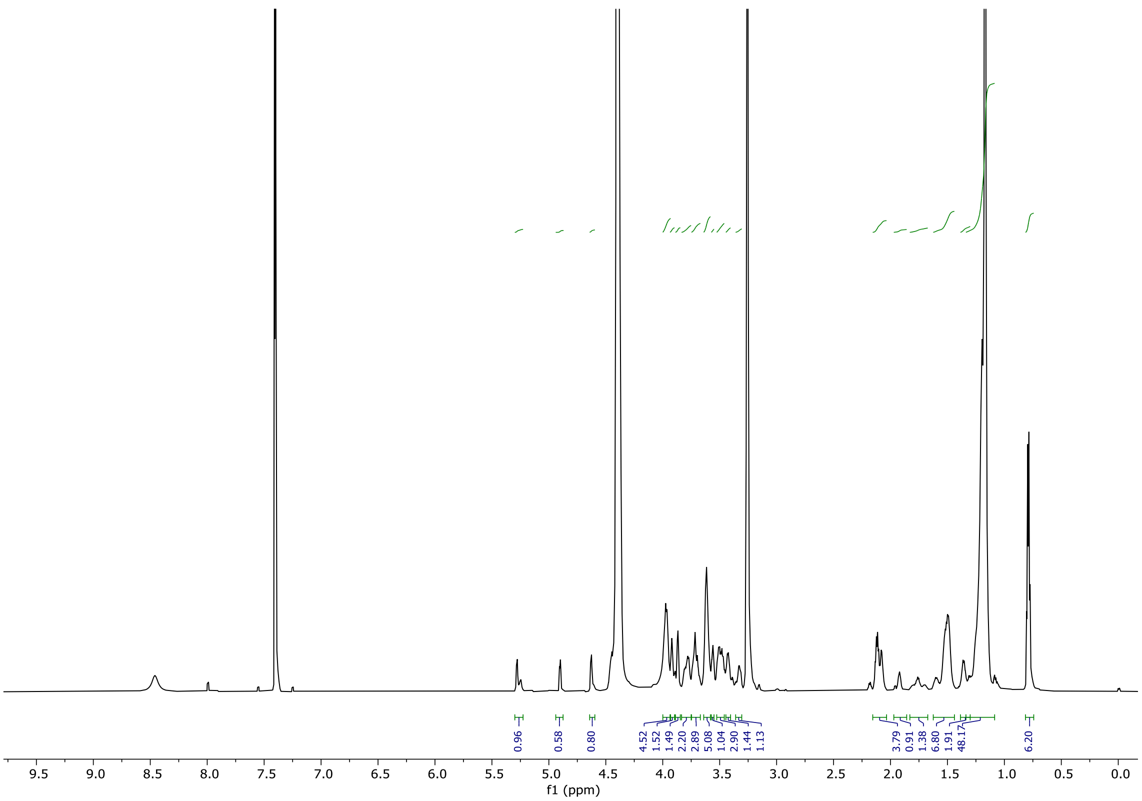

Figure 100. ^{1}H NMR (700 MHz, $CDCl_3$/MeOD, 2:1, *v/v*) spectrum of **14.**

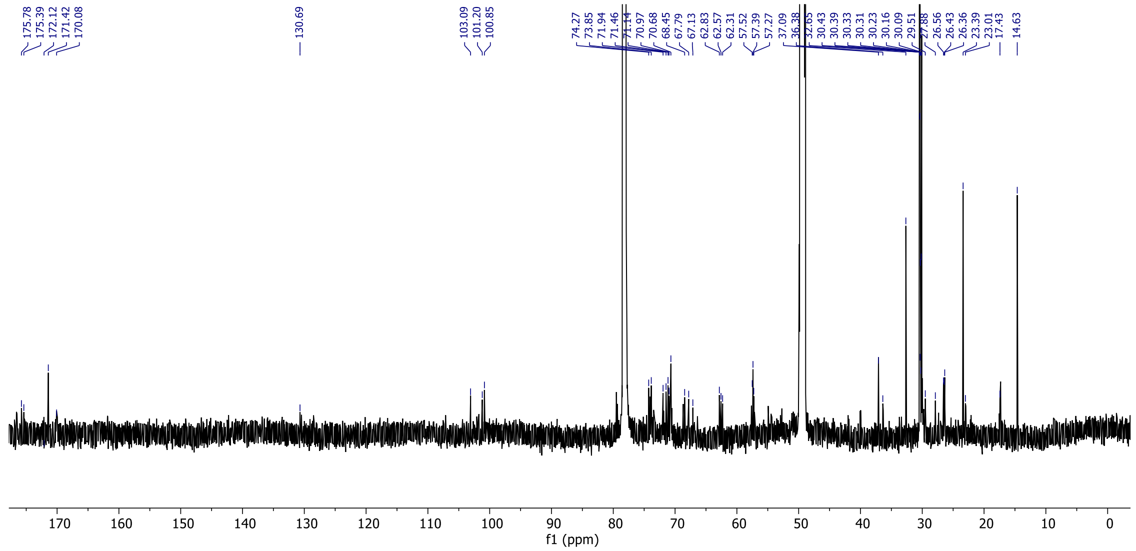

Figure 101. ^{13}C NMR (176 MHz, $CDCl_3$/MeOD, 2:1, *v/v*) spectrum of **14.**

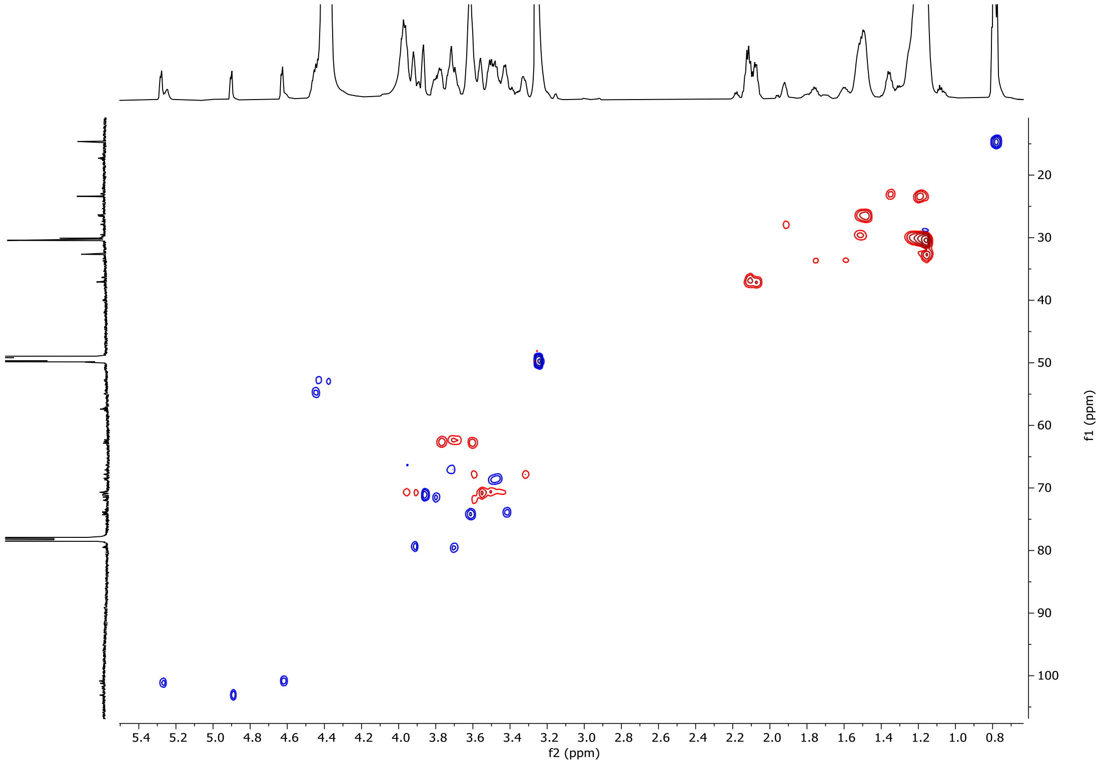

Figure 102. HSQC NMR (700 MHz, $CDCl_3$/MeOD, 2:1, *v/v*) spectrum of **14.**

Cho-PEG- α-(1→2)- α-(1→3)-trimannose ***(15)***

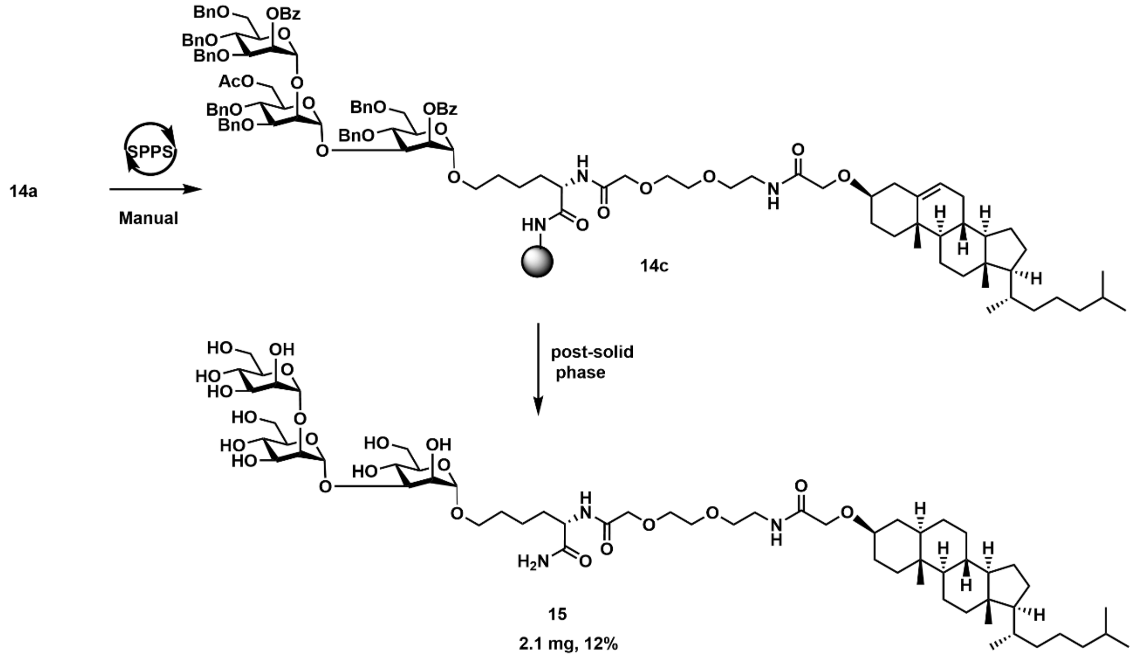

Glycolipid **15** (2.1 mg, 12%) was synthesized as a white solid from resin bound oligosaccharide **14a** (0.015 mmol) using stepwise SPPS (Method 2), followed by post-solid phase manipulation [hydrogenolysis (THF/tBuOH, 2:1, *v/v*)] and purification by semi-preparative RP-HPLC (Method A-4). R_t = 23.6 min. HR-MS (QTOF) m/z = 1206.6573 $[M+H]^+$, calcd. for $C_{59}H_{104}N_3O_{22}^+$: 1206.7106**.** ^{1}H NMR (700 MHz, MeOD/D_2O) δ 5.37 (d, *J* = 8.1 Hz, 1H), 4.42 (t, *J* = 6.9 Hz, 1H), 4.13 – 4.08 (m, 2H), 4.05 – 3.99 (m, 3H), 3.85 (dd, *J* = 9.0, 3.8 Hz, 2H), 3.77 – 3.68 (m, 8H), 3.66 (dt, *J* = 8.5, 3.9 Hz, 3H), 3.49 (p, *J* = 6.6 Hz, 2H), 2.27 – 2.22 (m, 1H), 2.04 – 1.95 (m, 2H), 1.92 – 1.85 (m, 2H), 1.76 (d, *J* = 10.1 Hz, 2H), 1.66 (dq, *J* = 12.2, 6.8 Hz, 2H), 1.61 – 1.56 (m, 2H), 1.52 (d, *J* = 7.9 Hz, 2H), 1.47 – 1.35 (m, 5H), 1.32 – 1.28 (m, 5H), 1.05 (dd, *J* = 7.5, 2.0 Hz, 2H), 0.96 – 0.84 (m, 9H), 0.69 (d, *J* = 9.5 Hz, 2H). ^{13}C NMR (176 MHz, MeOD/D_2O) δ 173.82, 172.73, 170.86, 103.67, 101.97, 101.17, 81.36, 79.96, 74.66, 70.77, 68.05, 62.52, 57.75, 57.63, 57.50, 57.38, 55.51, 53.85, 45.78, 43.54, 41.18, 40.39, 40.11, 39.55, 37.79, 37.04, 36.79, 36.61, 35.42, 33.00, 30.34, 29.70, 29.14, 28.85, 25.06, 24.55, 23.32, 23.13, 22.85, 22.15, 19.09, 17.29, 17.18, 17.07, 16.96, 16.85, 14.41, 12.68, 12.40.

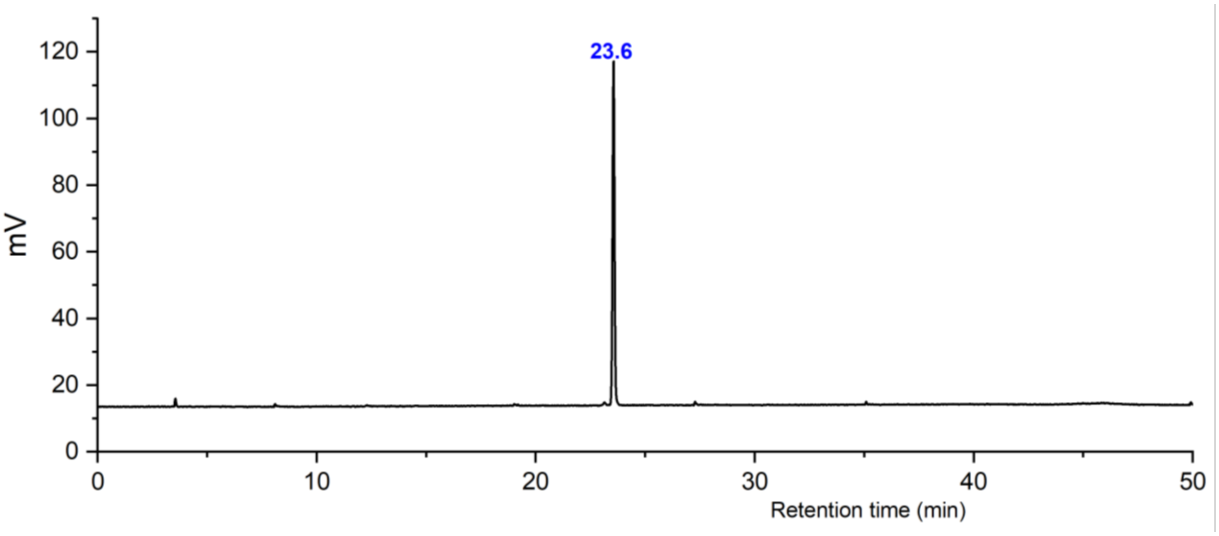

Figure 103. RP-HPLC trace of compound **15**.

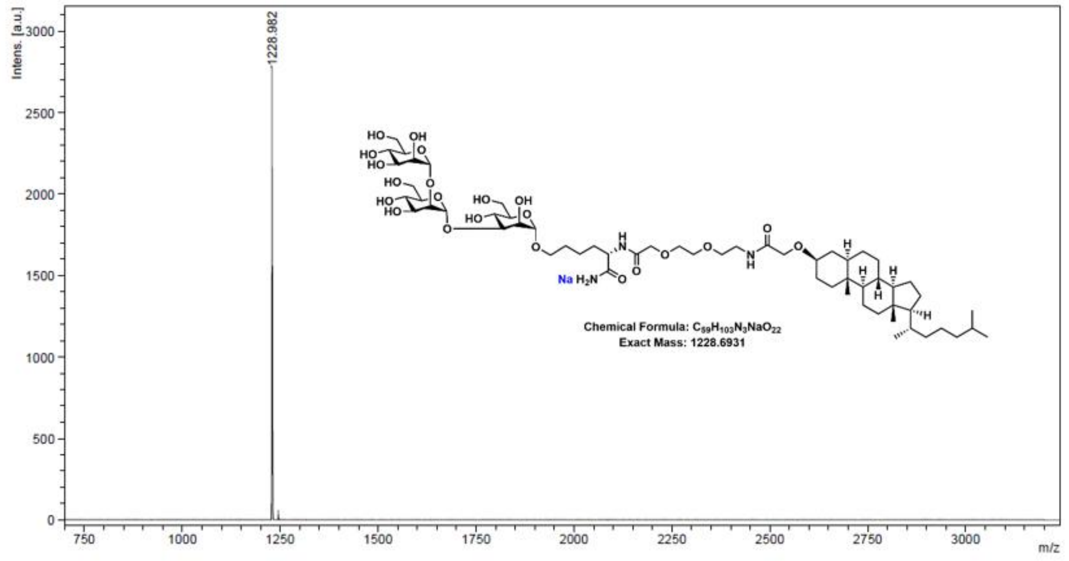

Figure 104. MALDI-MS of compound **15**.

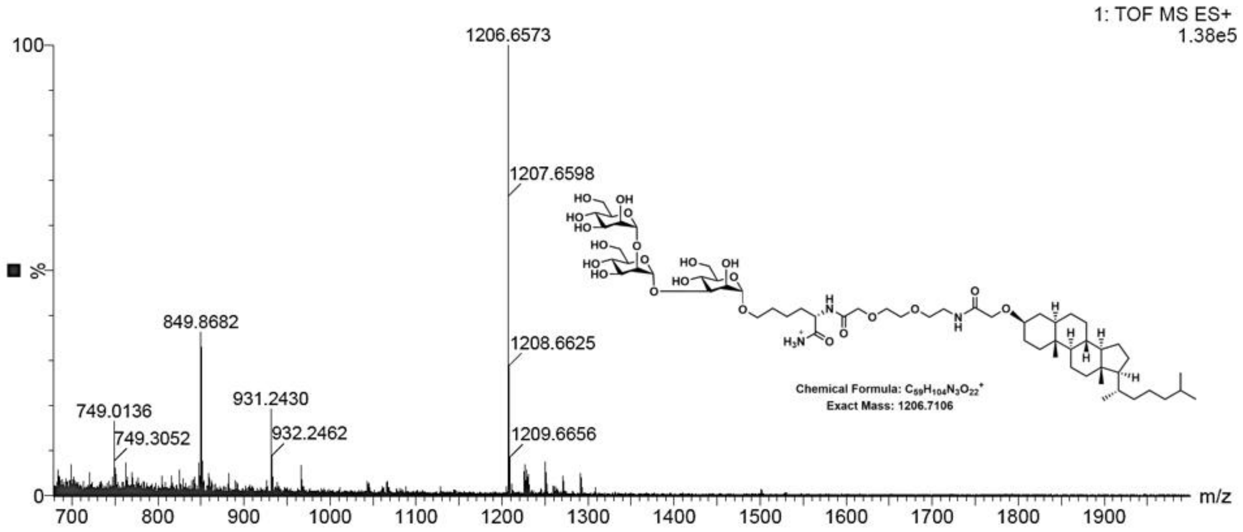

Figure 105. HR-MS of compound **15**.

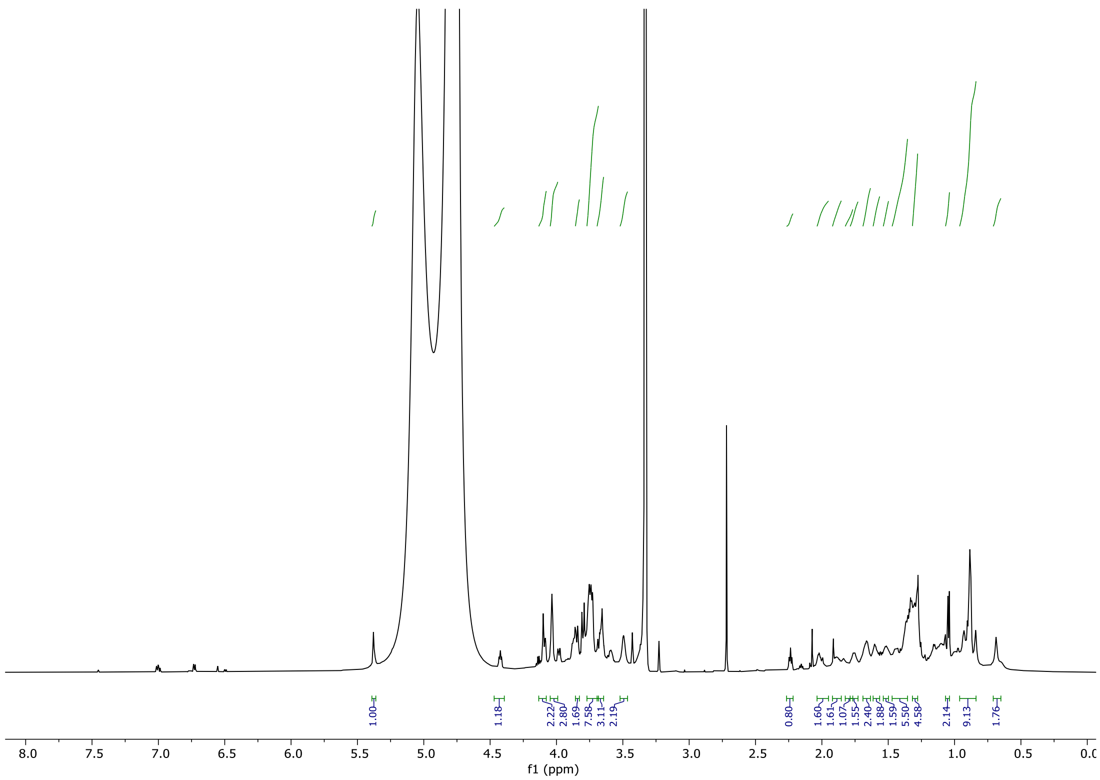

Figure 106. ^{1}H NMR (700 MHz, MeOD/D_2O, 2:1, *v/v*) spectrum of **15.**

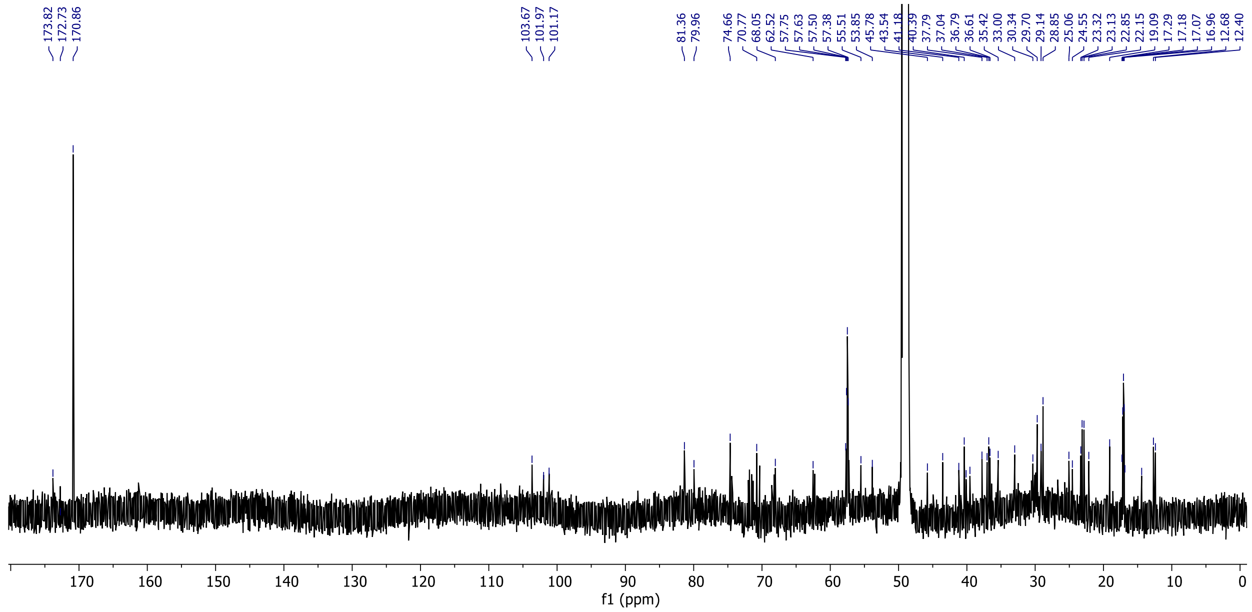

Figure 107. ^{13}C NMR (176 MHz, MeOD/D_2O, 2:1, *v/v*) spectrum of **15.**

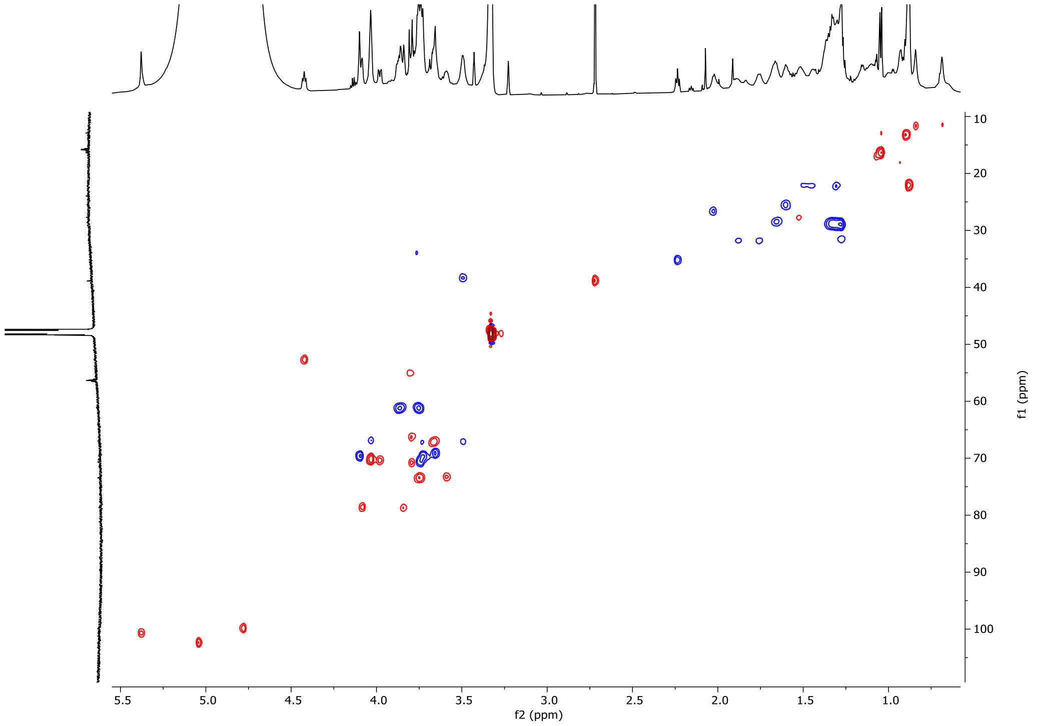

Figure 108. HSQC NMR (176 MHz, MeOD/D_2O, 2:1, *v/v*) spectrum of **15.**

*Pam$_2$-PEG$_2$-Gb3 (**16**)*

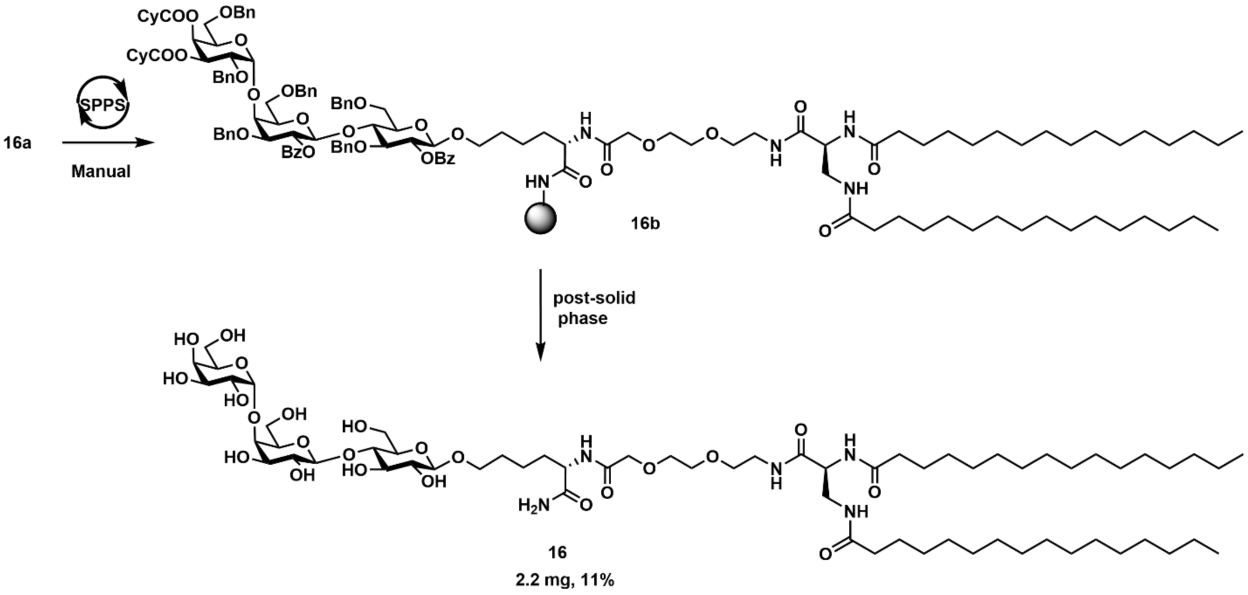

Glycolipid **16** (2.2 mg, 11%) was synthesized as a white solid from resin bound oligosaccharide **16a** (0.015 mmol) using stepwise SPPS (Method 2), followed by post-solid phase manipulation [hydrogenolysis (THF/tBuOH/H_2O, 2:1:1, *v/v*)] and purification by semi-preparative RP-HPLC (Method A-5). R_t = 21.4 min. HR-MS (QTOF) m/z = 1340.8472 $[M+H]^+$, calcd. for

$C_{44}H_{122}N_5O_{21}$: 1340.8525. ^{1}H NMR (700 MHz, $CDCl_3$/MeOD) δ 4.89 (s, 1H), 4.34 (s, 1H), 4.21 (s, 1H), 3.92 (s, 1H), 3.83 (s, 3H), 3.71 (t, *J* = 10.2 Hz, 2H), 3.63 (d, *J* = 7.6 Hz, 2H), 3.58 (s, 1H), 3.51 (s, 2H), 3.46 (s, 3H), 3.39 (s, 1H), 3.22 (d, *J* = 8.6 Hz, 1H), 2.16 (s, 1H), 2.14 (s, 1H), 2.09 (s, 1H), 1.97 (s, 2H), 1.78 (s, 3H), 1.57 (d, *J* = 54.2 Hz, 3H), 1.43 – 1.00 (m, 48H), 0.88 – 0.79 (m, 6H).^{13}C NMR (176 MHz, $CDCl_3$/MeOD) δ 177.06, 174.74, 174.73, 171.79, 170.89, 139.73, 103.73, 102.61, 101.42, 74.57, 73.12, 69.59, 69.07, 68.40, 61.55, 36.05, 34.35, 31.62, 30.19, 29.39, 29.34, 29.26, 29.12, 29.05, 28.81, 26.36, 25.34, 22.35, 21.50, 18.04, 13.57.

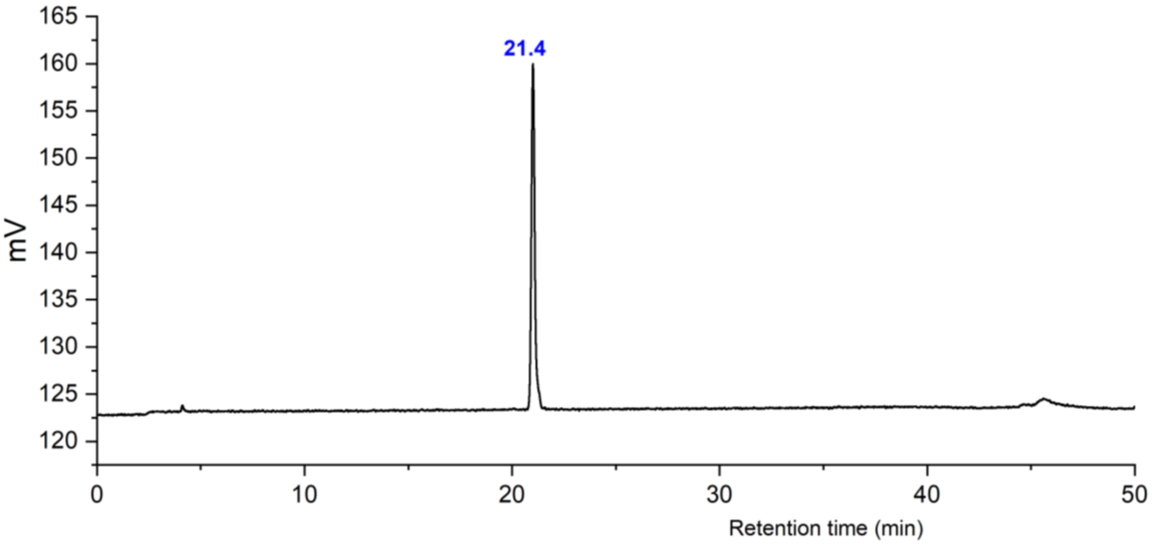

Figure 109. RP-HPLC trace of compound **16**.

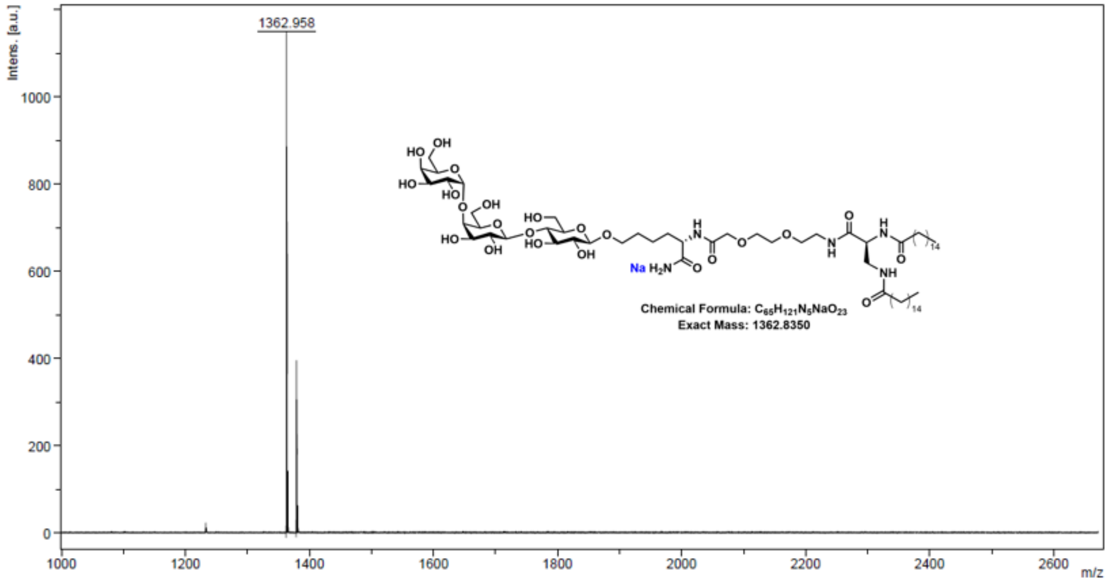

Figure 110. MALDI-MS of compound **16**.

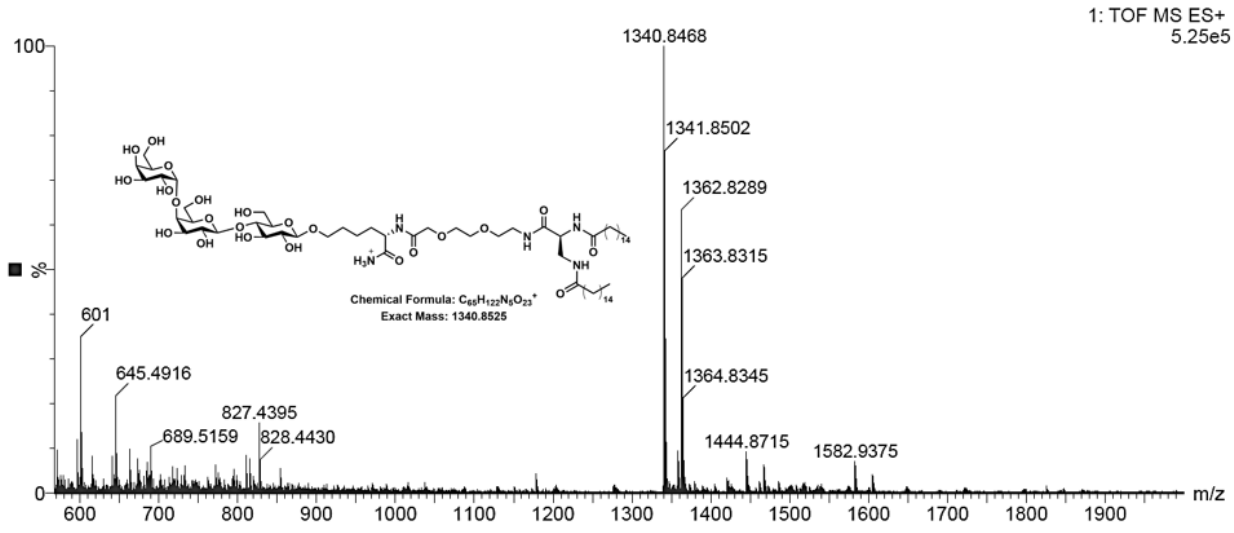

Figure 111. HR-MS of compound **16**.

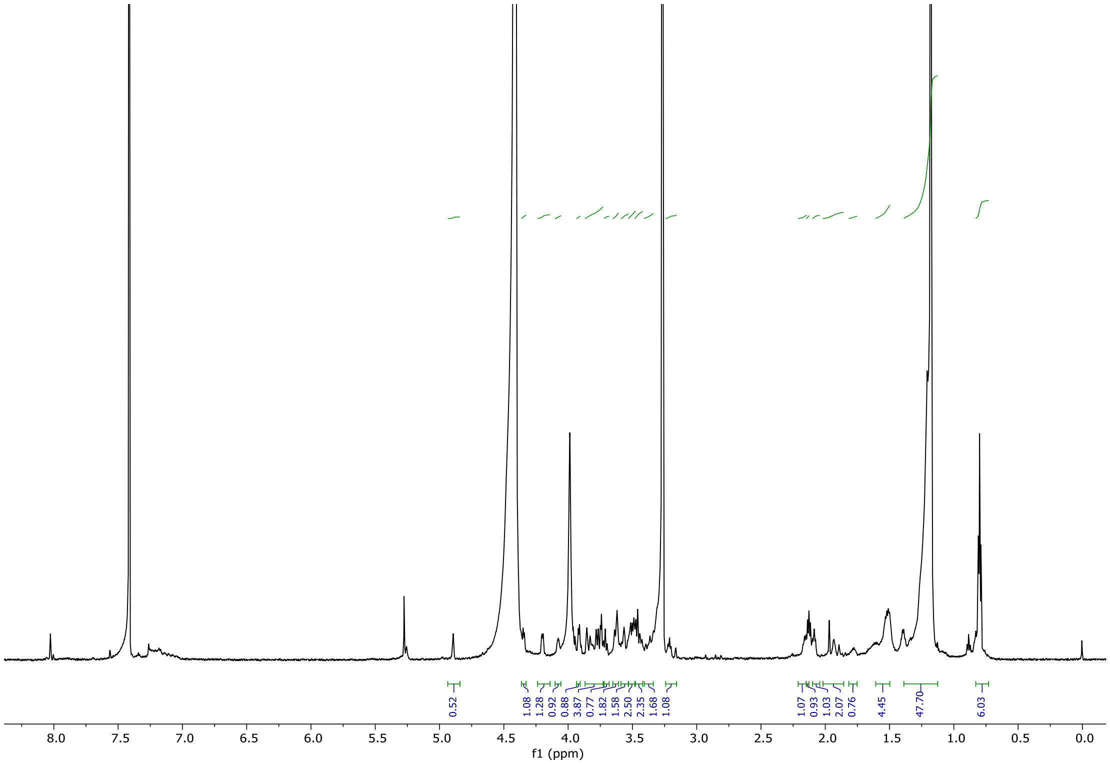

Figure 112. ^{1}H NMR (700 MHz, $CDCl_3$/MeOD, 2:1, *v/v*) spectrum of **16.**

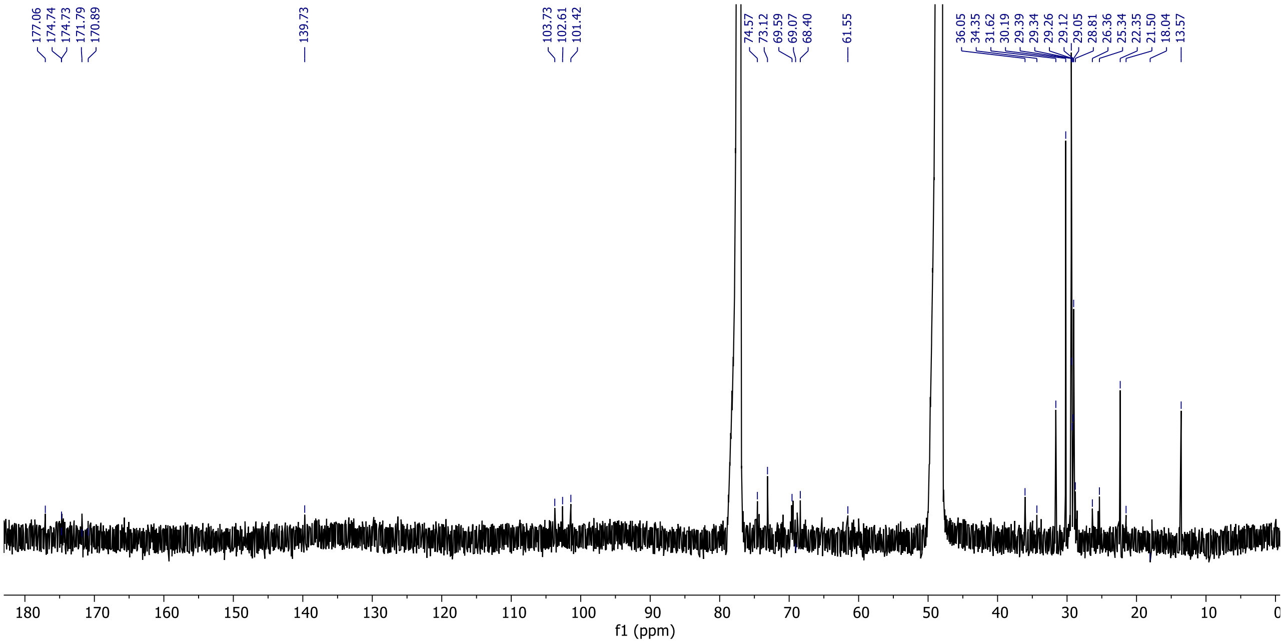

Figure 113. ^{13}C NMR (176 MHz, $CDCl_3$/MeOD, 2:1, *v/v*) spectrum of **16**.

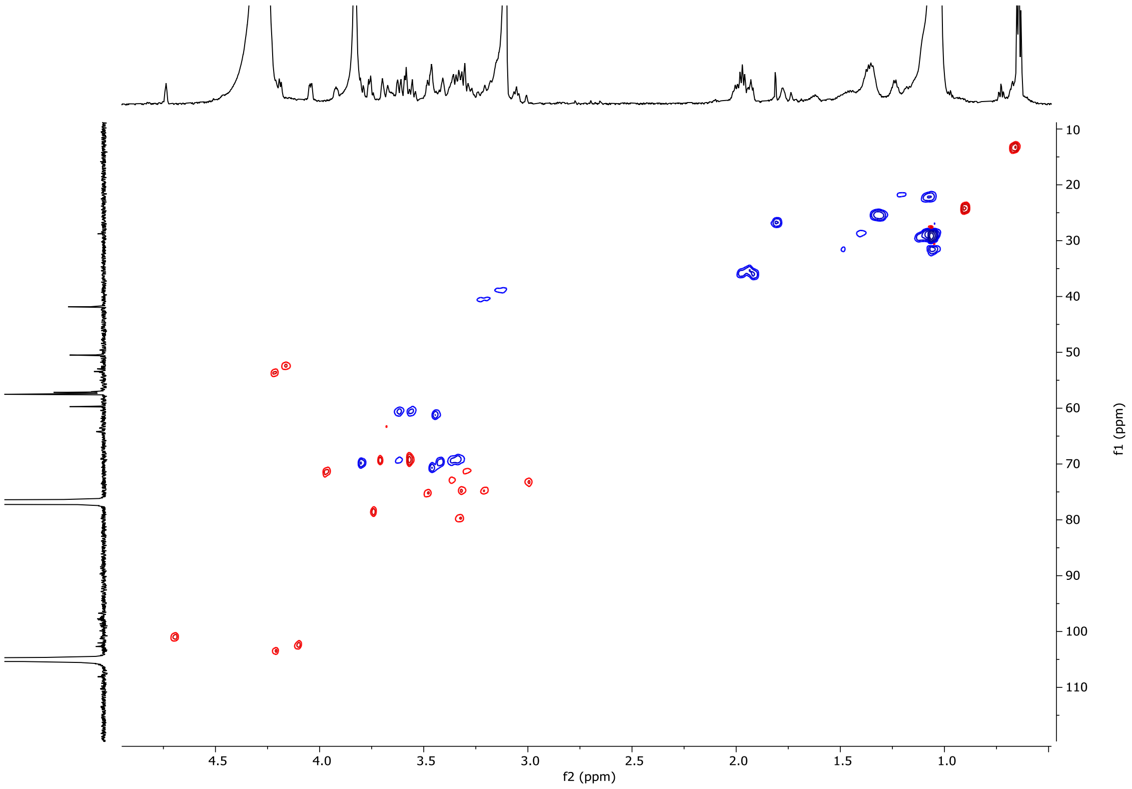

Figure 114. HSQC NMR (700 MHz, $CDCl_3$/MeOD, 2:1, *v/v*) spectrum of **2.16.**

*Cho-PEG$_2$-Gb3 (**17**)*

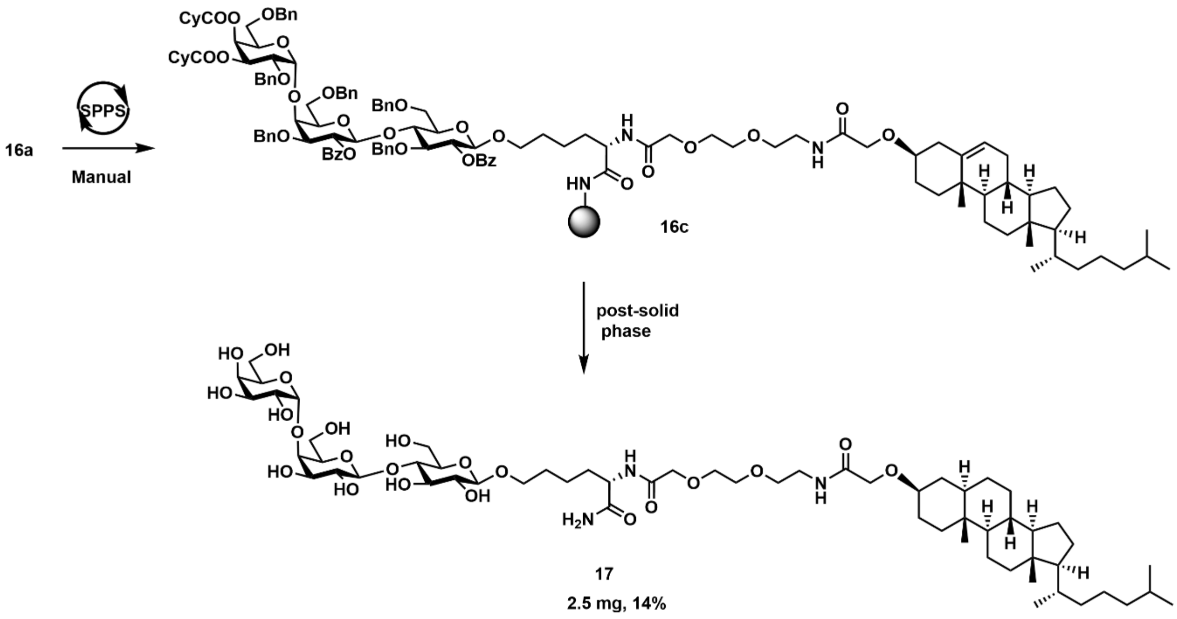

Glycolipid **17** (2.5 mg, 14%) was synthesized as a white solid from resin bound oligosaccharide **16a** (0.015 mmol) using stepwise SPPS (Method 2), followed by post-solid phase manipulation [hydrogenolysis (THF/tBuOH, 2:1, *v/v*)] and purification by semi-preparative RP-HPLC (Method A-4). R_t = 27.1 min. HR-MS (QTOF) m/z = 1206.7211 $[M+H]^+$, calcd. for $C_{59}H_{104}N_3O_{22}$: 1206.7106. ^{1}H NMR (700 MHz, MeOD/D_2O) δ 6.44 (s, 1H), 5.43 – 5.37 (m, 1H), 5.28 (dt, *J* = 24.4, 7.2 Hz, 1H), 5.03 (s, 1H), 4.97 (d, *J* = 3.8 Hz, 2H), 4.91 – 4.80 (m, 3H), 4.79 (t, *J* = 3.6 Hz, 2H), 4.68 (tt, *J* = 10.0, 4.5 Hz, 5H), 4.60 (d, *J* = 4.9 Hz, 2H), 4.59 – 4.50 (m, 3H), 4.43 (dq, *J* = 9.1, 5.5 Hz, 2H), 2.88 – 2.74 (m, 1H), 2.70 (dq, *J* = 12.9, 4.1 Hz, 1H), 2.67 – 2.59 (m, 2H), 2.50 – 2.39 (m, 3H), 2.30 (dt, *J* = 8.4, 4.6 Hz, 3H), 2.25 (td, *J* = 9.9, 4.4 Hz, 4H), 2.06 – 1.98 (m, 4H), 1.92 (ddd, *J* = 26.1, 12.9, 6.2 Hz, 2H), 1.86 (d, *J* = 6.6 Hz, 3H), 1.82 (dd, *J* = 6.7, 3.2 Hz, 5H), 1.78 (s, 2H), 1.62 (s, 2H).^{13}C NMR (176 MHz, MeOD/D_2O) δ 176.52, 173.53, 172.52, 104.68, 103.57, 101.91, 81.09, 80.26, 78.97, 76.33, 75.94, 74.22, 73.81, 72.20, 72.16, 71.61, 70.61, 70.44, 70.32, 70.14, 69.93, 67.84, 61.89, 61.22, 57.46, 57.22, 55.30, 54.60, 53.55, 45.57, 43.33, 40.97, 40.19, 39.33, 37.58, 36.85, 36.60, 36.41, 36.35, 35.22, 32.86, 32.69, 30.74, 29.60, 29.49, 28.93, 28.65, 24.85, 24.37, 22.90, 22.85, 22.62, 21.94, 18.87, 17.32, 13.96, 12.46, 12.18.

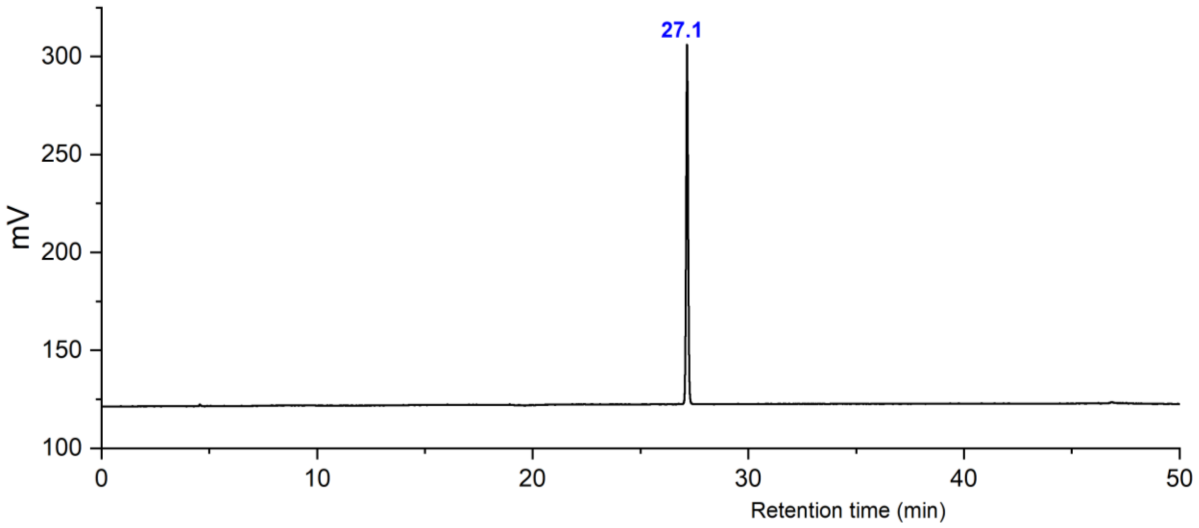

Figure 115. RP-HPLC trace of compound **17**.

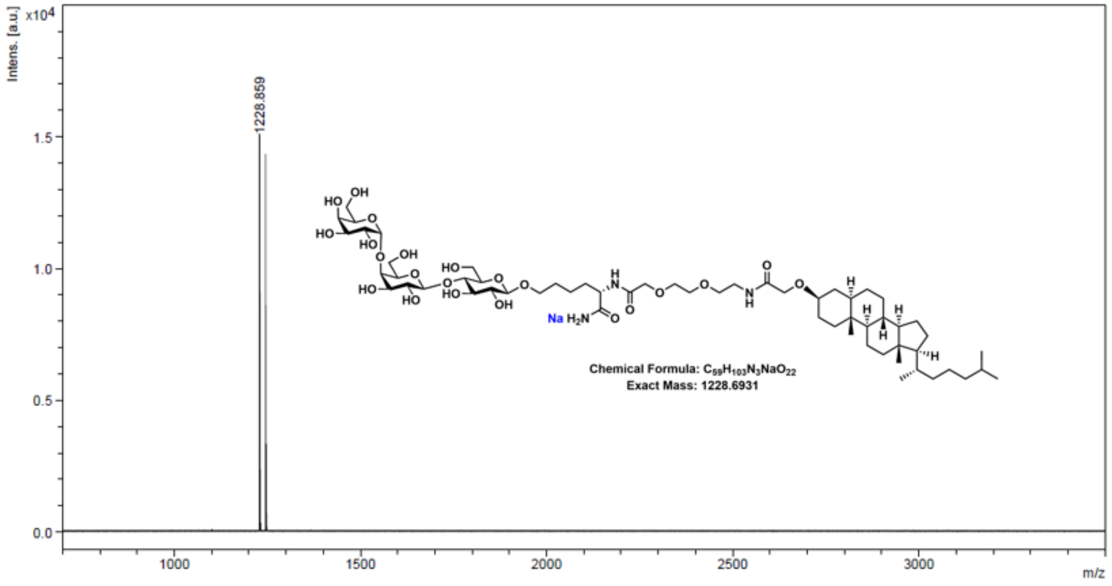

Figure 116. MALDI-MS of compound **17**.

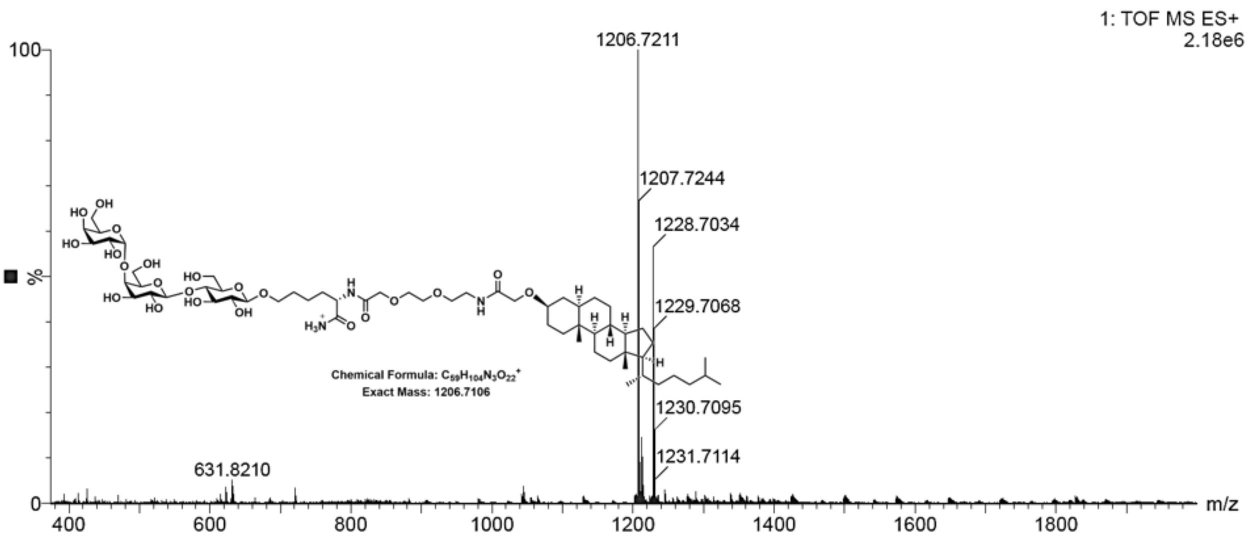

Figure 117. HR-MS of compound **17**.

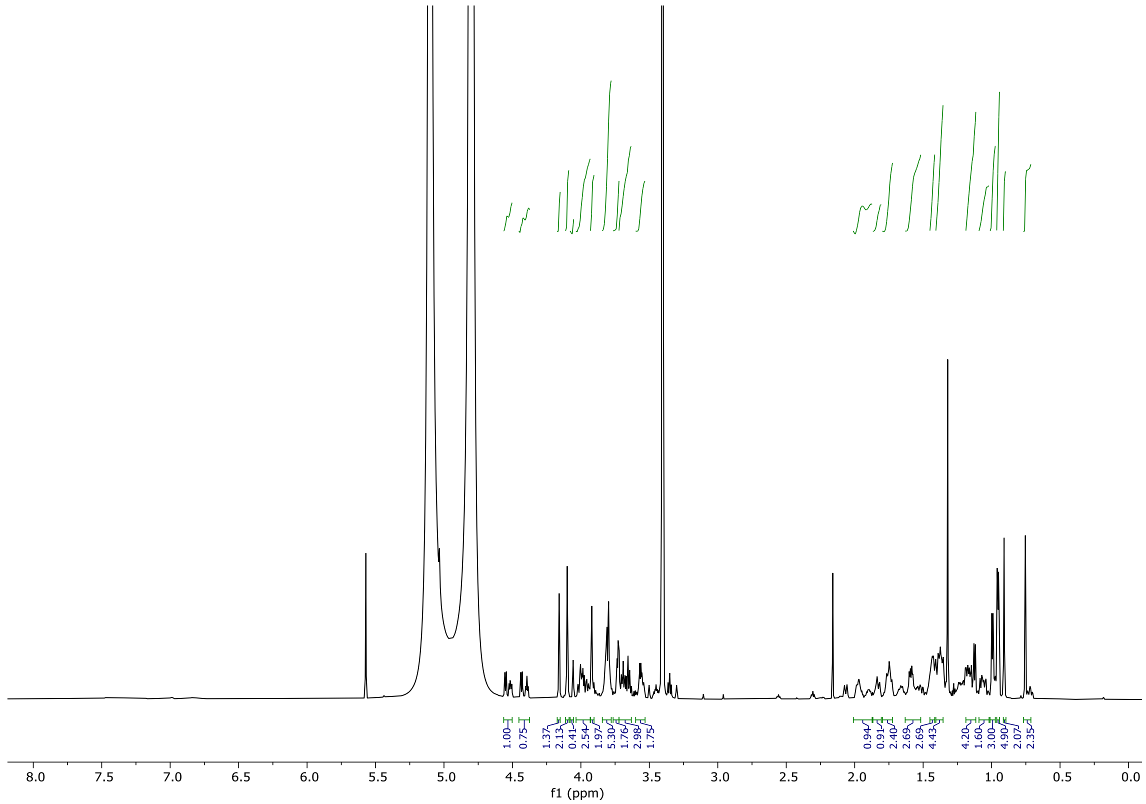

Figure 118. ^{1}H NMR (700 MHz, MeOD/D_2O, 2:1, *v/v*) spectrum of **17.**

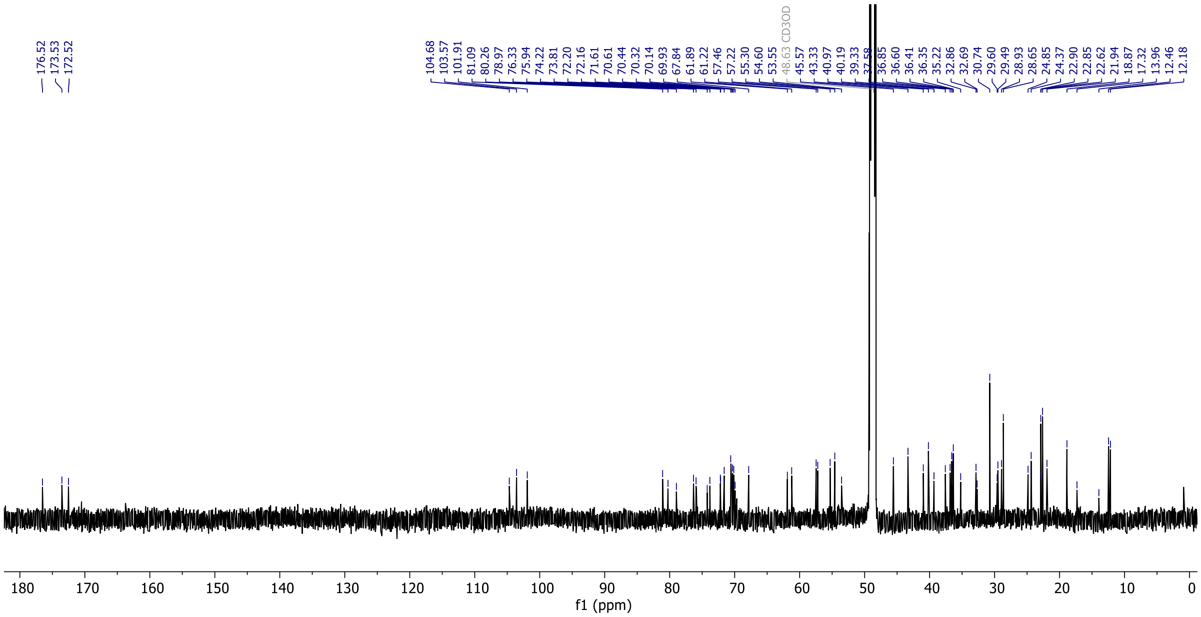

Figure 119. ^{13}C NMR (176 MHz, MeOD/D_2O, 2:1, *v/v*) spectrum of **17.**

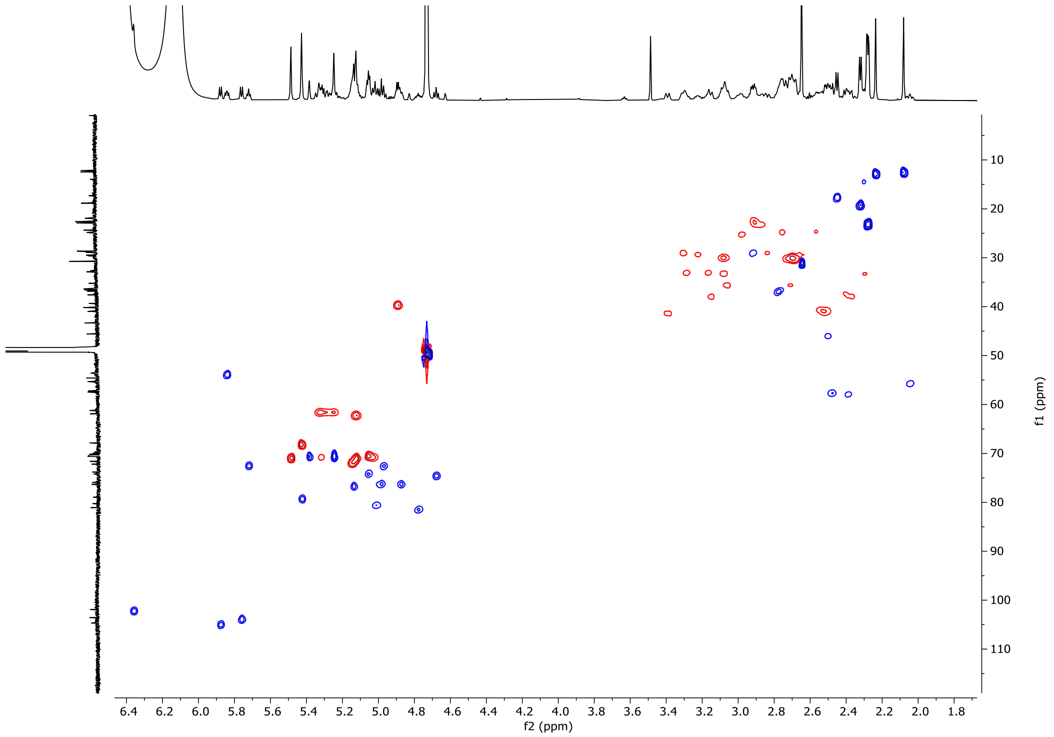

Figure 120. HSQC NMR (176 MHz, MeOD/D_2O, 2:1, *v/v*) spectrum of **17.**

6. Synthesis of fluorescent tag Cholesterol-K(FAM)

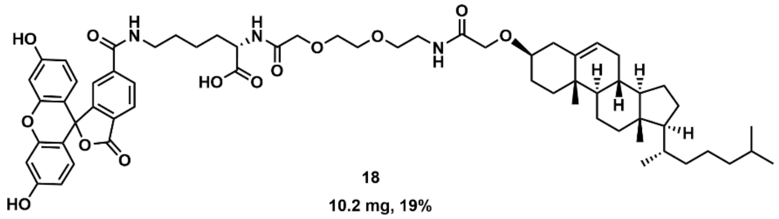

18

10.2 mg, 19%

Fluorescent tag **18** for labeling glycoliposomes was synthetized as a dark orange solid by manual SPPS (Method 2) on 2-CTC resin on a 0.05 mmol scale. The compound was cleaved from the resin by treatment with HFIP/*i*-Pr_3SiH/ CH_2Cl_2 (30:2:68, *v/v/v*) for 2×20 min. Solvents were evaporated under N_2 stream and crude was purified by column chromatography CH_2Cl_2/MeOH (gradient from 30:1 to 10:1, *v/v*) to afford the product (10.2 mg, 19%). R*f* (CH_2Cl_2/MeOH 30:1, *v/v*) = 0.35. HR-MS (QTOF) m/z =1076.5940 calcd for $C_{62}H_{82}N_3O_{13}^+$: 1076.5769. ^{1}H NMR (400 MHz, MeOD) δ 8.17 – 8.04 (m, 2H), 7.67 – 7.62 (m, 1H), 6.69 (d, *J* = 2.4 Hz, 2H), 6.65 – 6.50 (m, 4H), 5.35 – 5.29 (m, 1H), 4.42 (dd, *J* = 8.5, 4.9 Hz, 1H), 3.93 (d, *J* = 4.9 Hz, 4H), 3.70 – 3.49 (m, 6H), 3.45 – 3.37 (m, 2H), 3.22 – 3.11 (m, 1H), 2.33 (ddd, *J* = 13.1, 4.9, 2.3 Hz, 1H), 2.25 – 2.13 (m, 1H), 2.06 – 1.66 (m, 7H), 1.66 – 1.33 (m, 14H), 1.33 – 1.17 (m, 3H), 1.17 – 1.02 (m, 7H), 1.02 – 0.86 (m, 15H), 0.69 (s, 3H). ^{13}C NMR (100 MHz, MeOD) δ 175.89, 171.87, 168.04, 167.42, 167.00, 157.87, 153.40, 141.83, 140.93, 129.70, 129.61, 123.44, 122.40, 113.13, 110.29, 103.04, 81.64, 80.95, 71.45, 70.42, 69.93, 67.65, 57.46, 56.90, 50.96, 42.84, 40.46, 40.24, 40.06, 39.24, 39.01, 37.61, 37.24, 36.73, 36.48, 32.56, 32.37, 32.06, 29.20, 28.68, 28.52, 24.67, 24.31, 23.50, 22.55, 22.31, 21.54, 19.22, 18.62, 11.69.

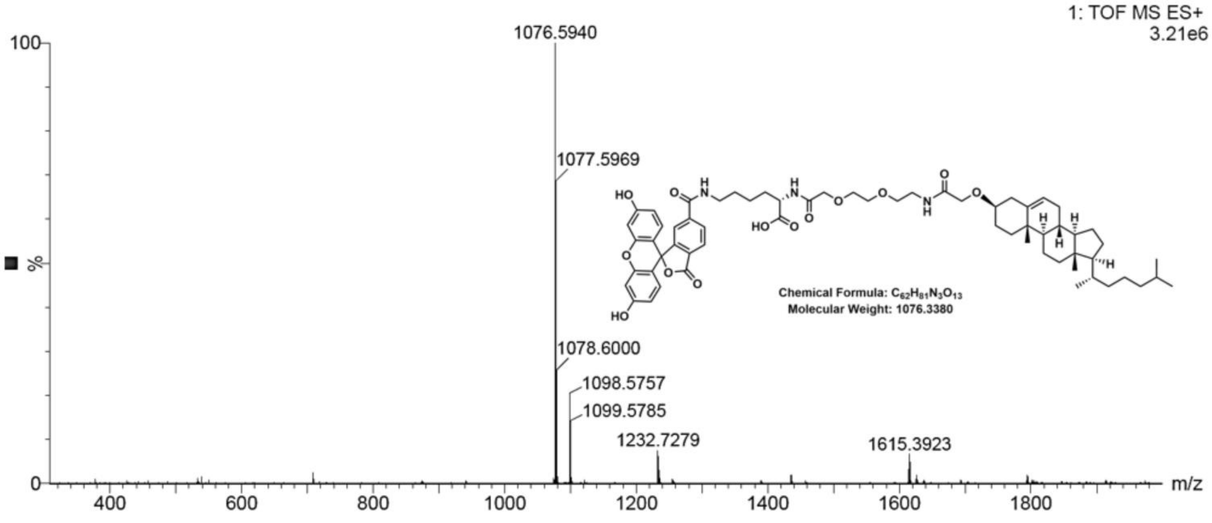

Figure 121. HR-MS of compound **18**.

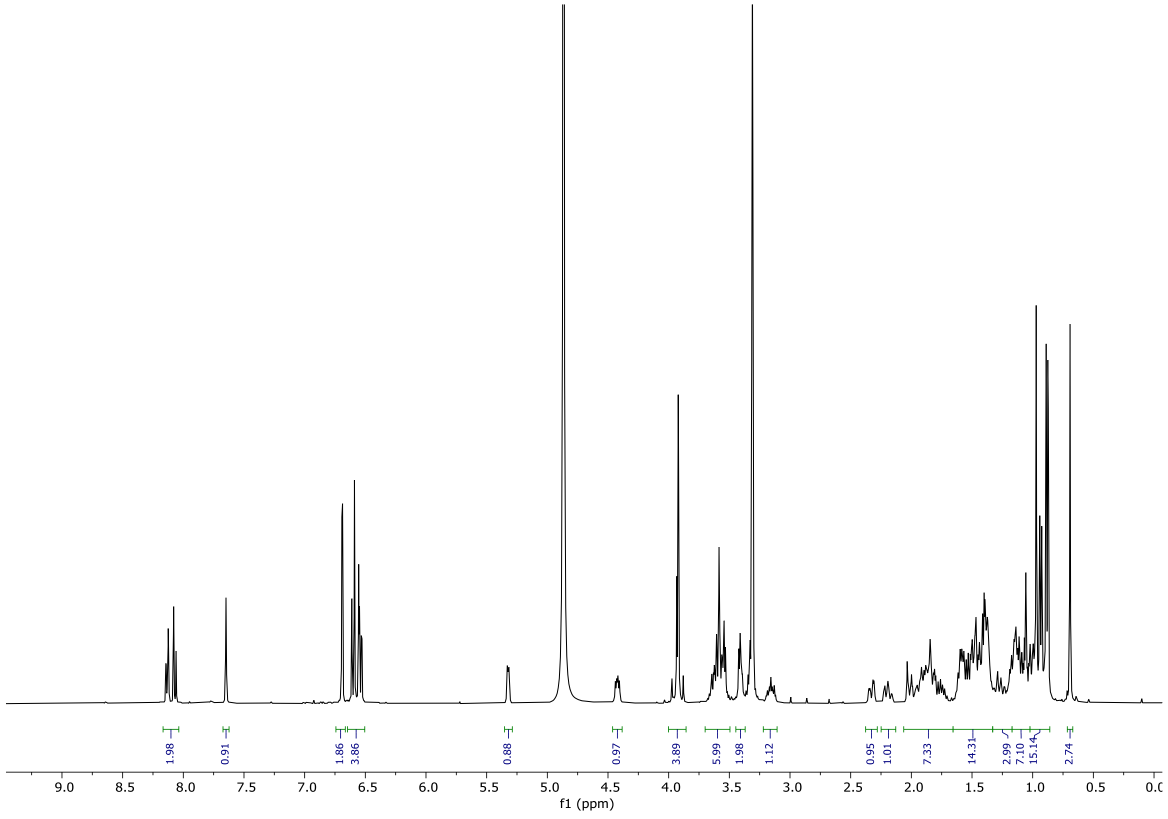

Figure 122. ^{1}H NMR (400 MHz, MeOD) spectrum of **18.**

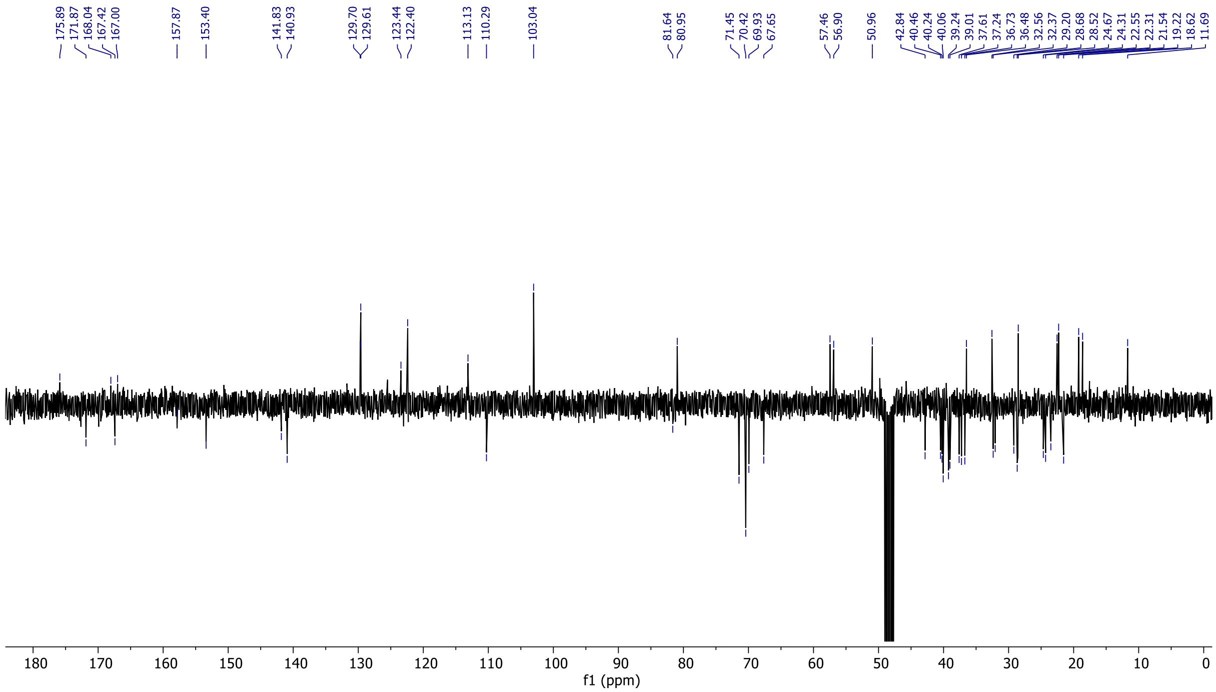

Figure 123. ^{13}C NMR (100 MHz, MeOD) spectrum of **18.**

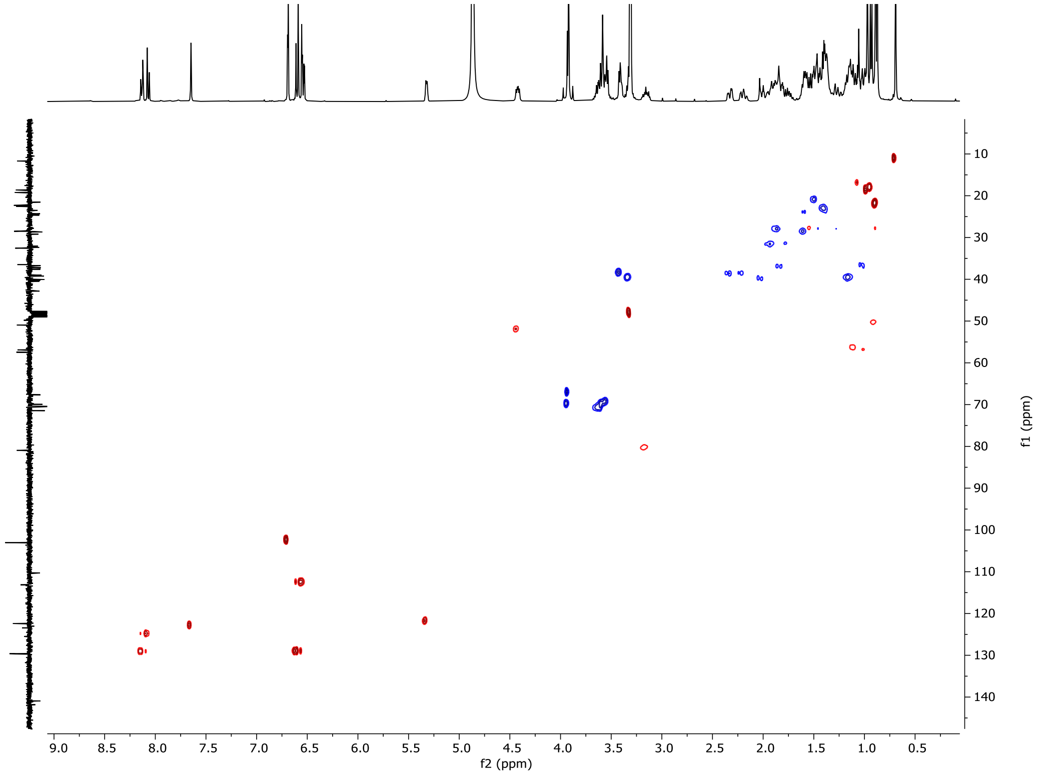

Figure 124. HSQC NMR (400 MHz, MeOD) spectrum of **18.**

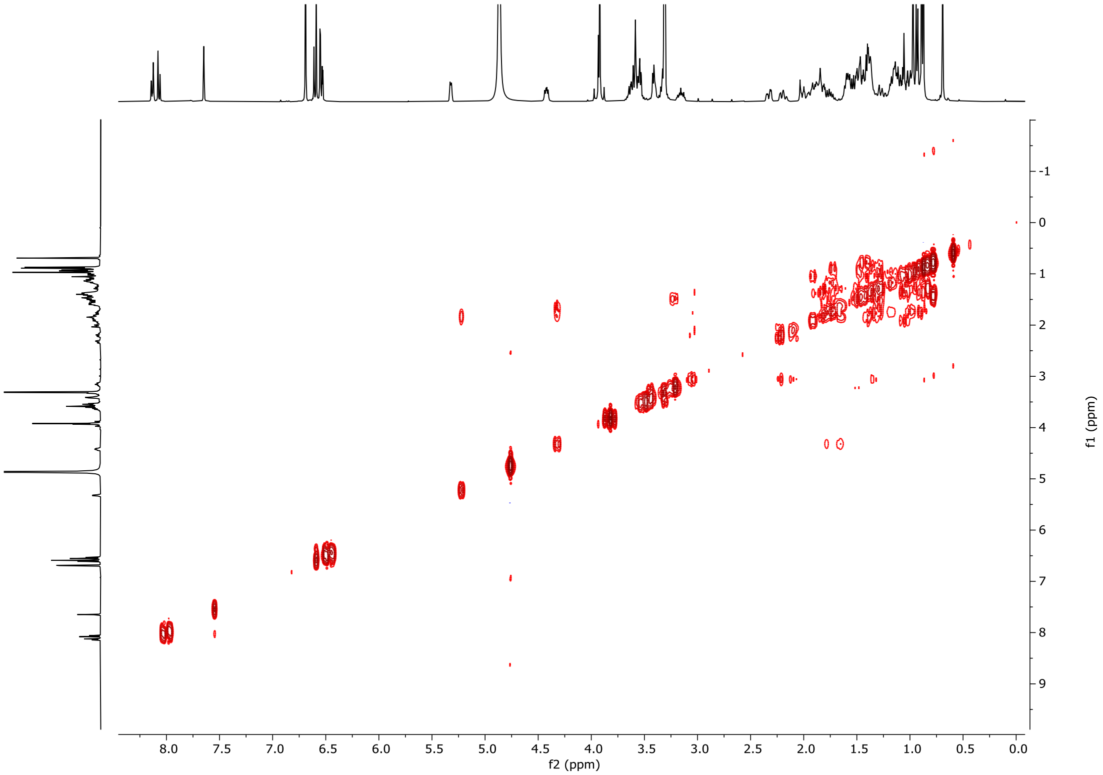

Figure 125. COSY NMR (400 MHz, MeOD) spectrum of **18.**

7. Materials and methods for liposomal formulation of synthetic glycolipids

LNPs were prepared according to the thin film hydration method followed by extrusion.[7] DSPC, Cholesterol and the corresponding glycolipid were mixed in $CHCl_3$/MeOH 10:1 at different molar ratios of each lipid (Table 3). Solvents were evaporated under reduced pressure in a rotary evaporator and the flask was placed under vacuum overnight to remove any residual solvent. MilliQ water was added and flask was vigorously shaken to hydrate the lipid film to a final lipid concentration of 11 mM over 1 h at 65°C. The suspension was sonicated (Sonorex; Bandelin, Berlin, Germany) for 2 min and then extruded (Avanti Polar Lipids, Inc., Alabaster, AL, USA) 21 times through stacked 400 nm and then through a 200 nm polycarbonate filters. The resulting milky solution was centrifugated at 10 000 rpm for ten minutes. Supernatant was collected and analyzed by RP-HPLC and pellet was resuspended in MilliQ water. Centrifugation was repeated two times. The resulting GLPN was stored at 4 °C. Formulations were characterized by size, size distribution, ζ potential and imaged by TEM. For fluorescent-labelled liposomes, synthetic fluorescent tag **18** was added to the original lipid mixture at a concentration of 1 molar % of the total lipids and the samples were protected from light during the preparation procedure.

Table 3. Composition of lipid nanoparticles

Entry	DSPC	Cholesterol	Glycolipids					
			**12**	**14**	**16**	**13**	**15**	**17**
**1**	60	40	0	0	0	0	0	0
**2**	55	40	**5**	0	0	0	0	0
**3**	50	40	**10**	0	0	0	0	0
**4**	45	40	**15**	0	0	0	0	0
**5**	60	35	0	0	0	**5**	0	0
**6**	60	30	0	0	0	**10**	0	0
**7**	60	25	0	0	0	**15**	0	0
**8**	45	40	0	**15**	0	0	0	0
**9**	60	25	0	0	0	0	**15**	0
**10**	45	40	0	0	**15**	0	0	0
**11**	60	25	0	0	0	0	0	**15**

7.1. Physicochemical characterization of formulations

The size, size distribution and ζ-potential of lipid nanoparticles were measured in a Malvern Instruments Nano-ZS Zetasizer equipped with a 632.8 nm, 4 mW HeNe laser (Malvern Panalytical, UK) at 25°C. All the samples were dissolved in MilliQ water (viscosity setting of 0.890 cP) at a ratio of 1:100 (*v/v*). The particle size and size distribution were determined using disposable plastic micro cuvettes (ZEN0040; Malvern Panalytical, UK) on a fixed angle of 173°. The particle size was reported as an average diameter in nm, while the particle size distribution was related to as polydispersity index (PDI). The ζ-potential was measured in MilliQ water using folded capillary cells (DTS1070; Malvern, Worcestershire, UK). The ζ-potential of the samples was automatically calculated based on the Smoluchowski equation[8] using the Zetasizer software version 7.1 (Malvern, Worcestershire, UK).

TEM measurements were carried out by Heike Runge from the Biomaterials Department Development of Mineralized Skeletal Materials group at Max Planck Institute for Colloids and Interfaces. LNPs solutions (5 µL) used for DLS measurements were stained with uranyl acetate (5 µL, 1% in water, *w/v*) for two minutes and placed on a 300-mesh copper TEM grids (Agar Scientific, Stansted, U.K.) After one minute of adsorption, the excess was blotted with a Whatman

filter paper. The samples were let to air-dry for 30 minutes. The images were acquired at 120 000× magnification using a TEM JEOL JEM-ARM200F. Microscope operating at 200 KV acceleration voltage and equipped with a Gatan OneView (4k x 4k).

8. Liposomal formulations

8.1. Conventional liposomes

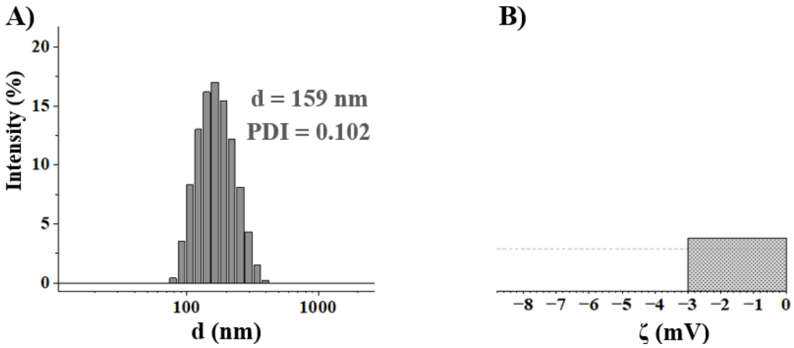

Figure 126. A) Size and size distribution by intensity of uncoated LNPs (Table 3, entry 1) determined by DLS (0.1 μM in H_2O, n = 3). **B)** Zeta potential measurements of uncoated LNPs (Table 3, entry 1) (0.1 μM in H_2O, n = 3). Values are the mean.

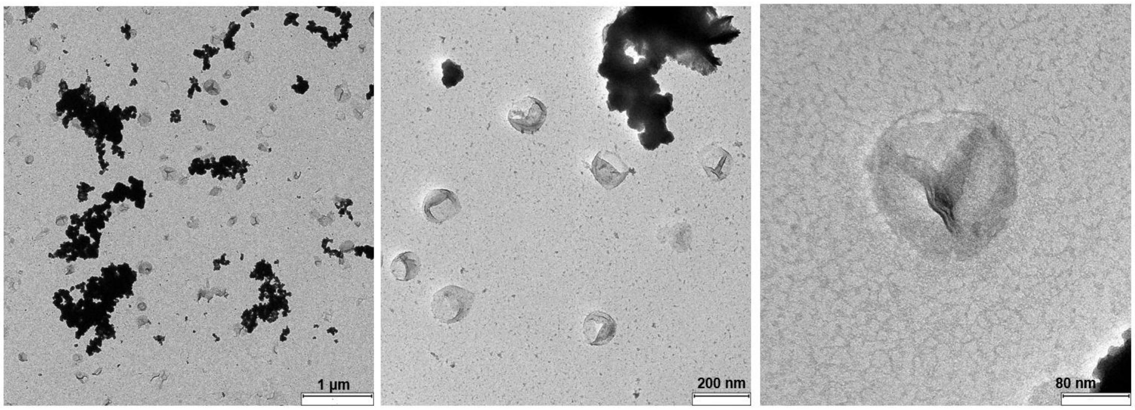

Figure 127. TEM images of conventional DSPC/Cholesterol liposomes (Table 3, entry 1).

8.2. α-(1→2)-Man-LNPs

Molar concentrations of 5–15% (Table 3, entry 2–4) for Pam_2-1,2-Man (**12**) were used to partially replace the total amount of DSPC used in the conventional formulation (Table 3, entry 1) and molar concentrations of 5–15% (Table 3, entry 6–8) for Cho-1,2-Man (**13**) were used to substitute the amount of cholesterol conventionally used (Table 3, entry 1).

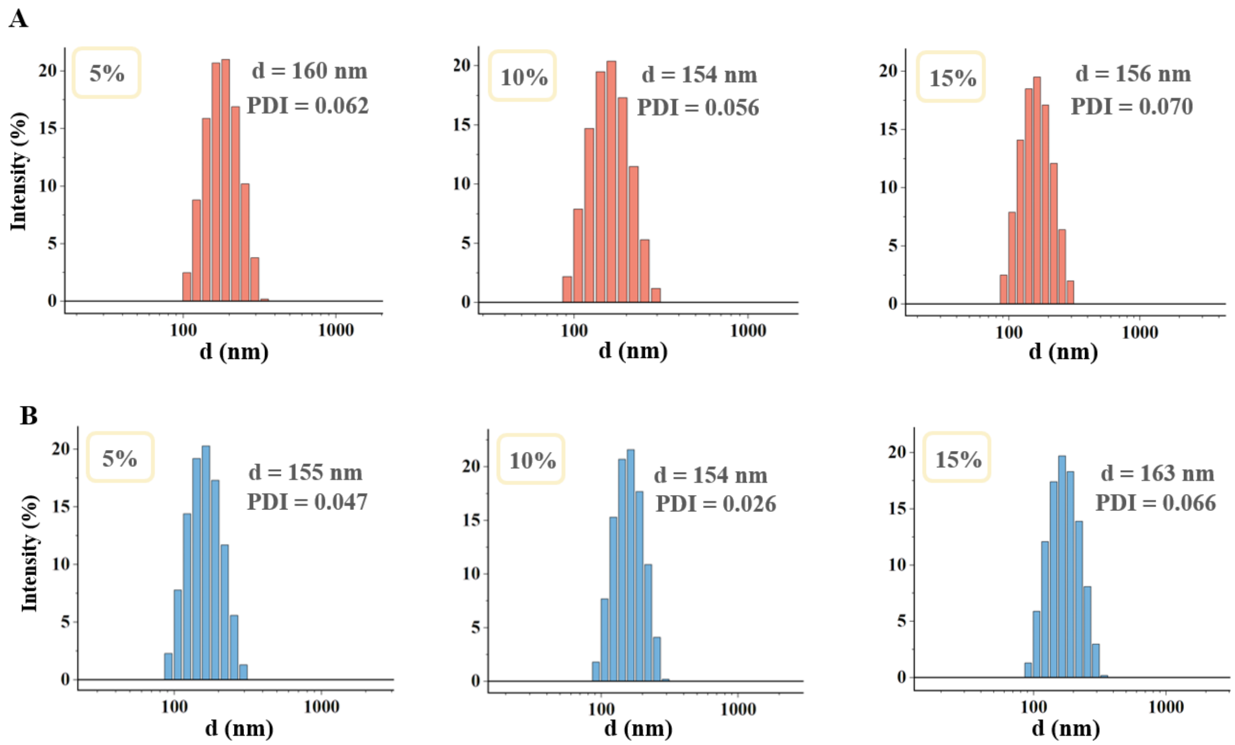

Figure 128. Size and size distribution by intensity of LNPs containing different molar percentages of **A)** Pam$_2$-α-1,2-Man (Table 1, entry 2–4) and **B)** Cho-α-1,2-Man (Table 1, entry 6–8) determined by DLS (100 μM in H_2O, n = 3). Values are the mean.

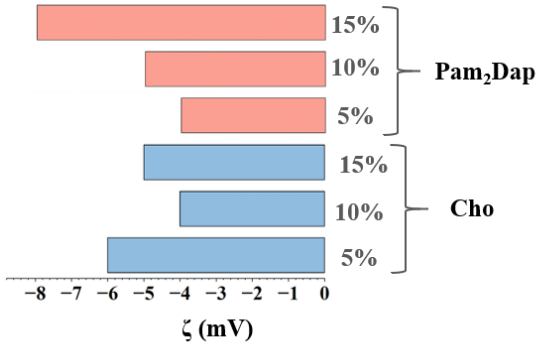

Figure 129. Zeta potential of MLNPs (Table 1, entry 2–8) (0.1 μM in H_2O, n = 3). Values are the mean.

8.2.1. ConA binding with mannosylated LNP

8.2.1.1. Agglutination study of mannosylated LNP with ConA by DLS

Crosslinking studies were conducted with the lectin Concanavalin A in 0.1 M HEPES buffer [(10 mM), NaCl (10 mM), $CaCl_2$ (1 mM) and $MnCl_2$ (1 mM)] at pH 7.4, as previously reported.[9,10] Mannosylated LNP (Table 3, Entry 2, 10 μL) were co-incubated with 1 mL of 0.1 M HEPES buffer

containing ConA (0.1 mg/mL). Size and size distribution were measured using a Zetasizer after specific time intervals (0, 15, 30, 60, 75 minutes).

8.2.1.2. Qualitative determination of lectin binding with mannosylated LNP

Exposition of mannosylated lipids on the lipid nanoparticles surface was qualitatively determined by measuring their recognition by ConA-FITC. The binding studies of entry 1 (negative control) and 2 (mannosylated LNP of least percentage of substitution for mannose lipid) with ConA-FITC were conducted in 0.1 M HEPES buffer [(10 mM), NaCl (10 mM), $CaCl_2$ (1 mM) and $MnCl_2$ (1 mM)] at pH 7.4 using confocal fluorescent microscopy. Mannosylated LNP (Table 3, Entry 2, 10 µL) were co-incubated with 1 mL of 0.1 M HEPES buffer containing ConA-FITC (0.1 mg/mL). Samples were visualized using a Leica SP8 microscope equipped with a hybrid detector with a laser (0.1%) at 496 nm.

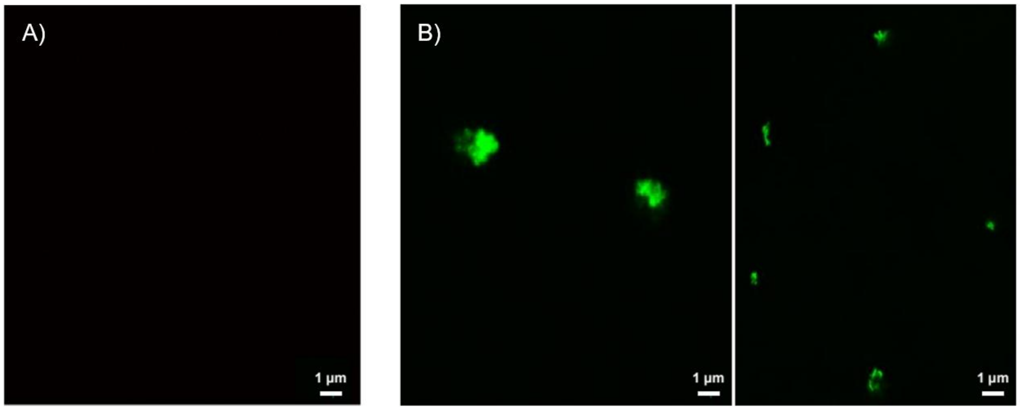

Figure 130. Fluorescent microscopy images of binding studies of ConA-FITC with **A)** Conventional liposomes (Table 3, entry 1) and **B)** LNPs coated with 5% Pam_2Dap- PEG_2-α-1,2-Man (Table 3, entry 2). Fluorescent signal indicates recognition of mannosylated structures by ConA-FITC.

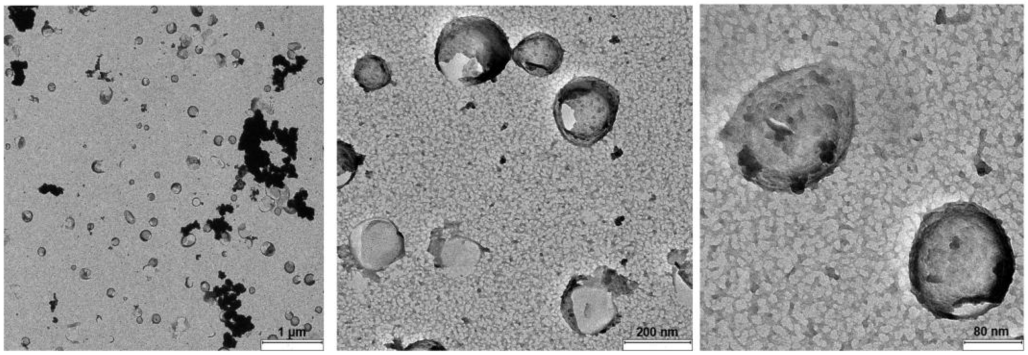

Figure 131. TEM images of LNPs coated with 5% Pam_2Dap- PEG_2-α-1,2-Man (Table 3, entry 2).

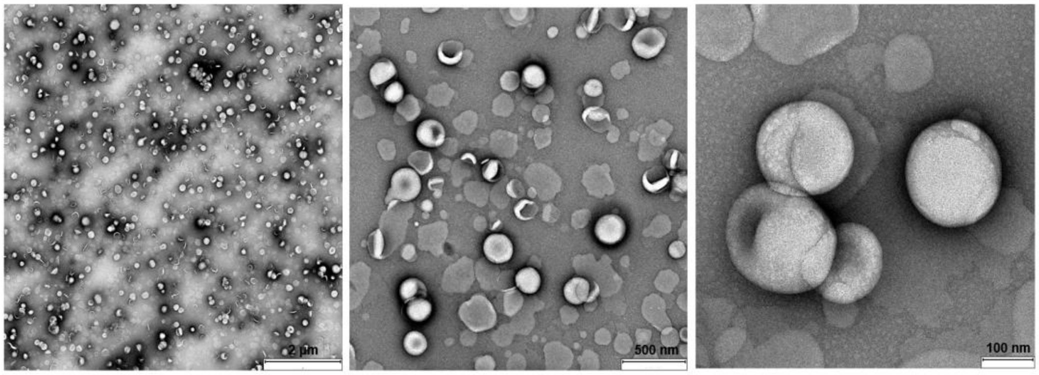

Figure 132. TEM images of LNPs coated with 10% Pam_2Dap- PEG_2-α-1,2-Man (Table 3, entry 3).

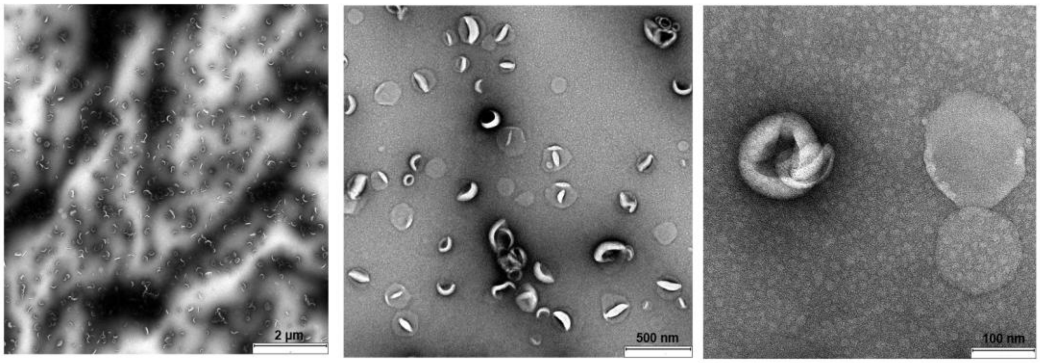

Figure 133. TEM images of LNPs coated with 15% Pam_2Dap- PEG_2-α-1,2-Man (Table 3, entry 4).

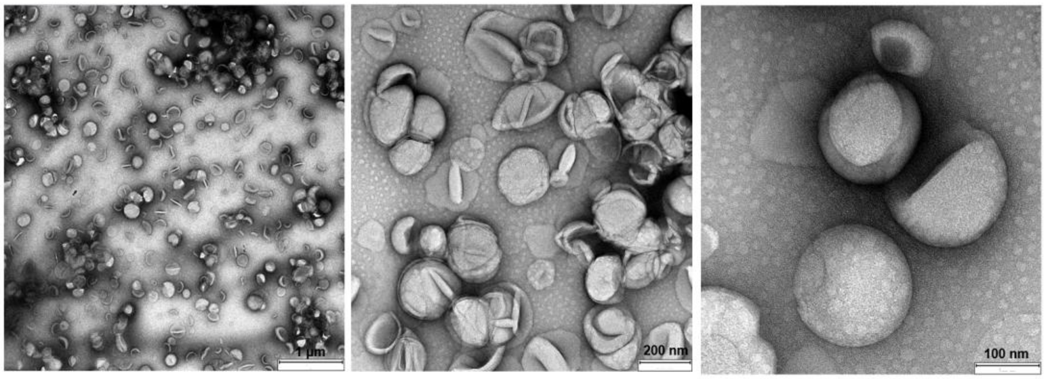

Figure 134. TEM images of LNPs coated with 5% Cho-PEG_2-α-1,2-Man (Table 3, entry 5).

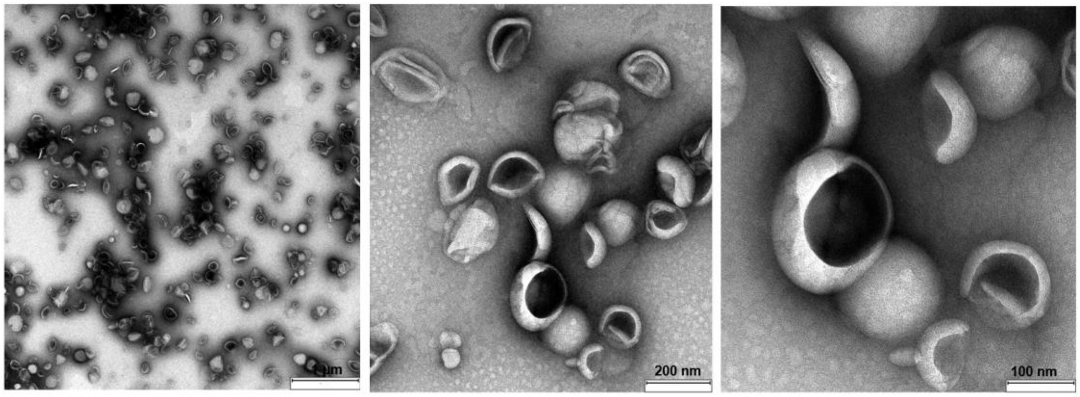

Figure 135. TEM images of LNPs coated with 10% Cho-PEG$_2$-α-1,2-Man (Table 3, entry 6).

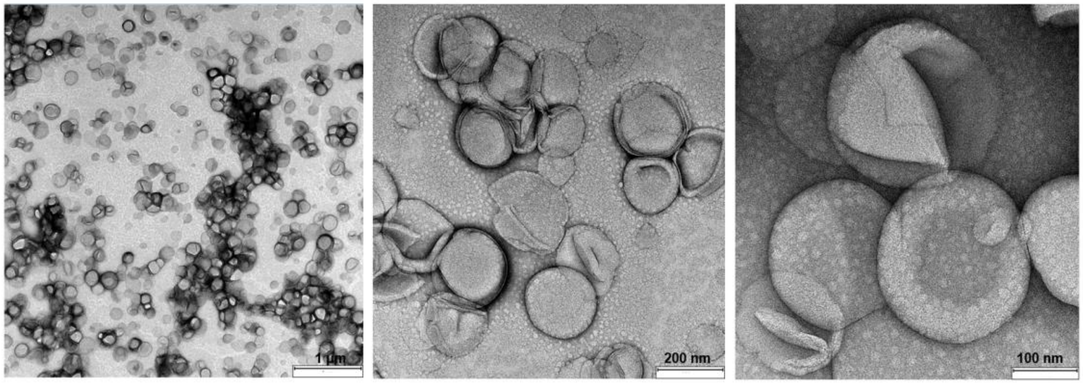

Figure 136. TEM images of LNPs coated with 15% Cho-PEG$_2$-α-1,2-Man (Table 3, entry 7).

8.3. α-(1→2)- Man α-(1→3)-Man-LNPs

Molar concentration of 15% (Table 3, entry 8) of Pam$_2$Dap-PEG$_2$-α-1,2, Man 1,3-Man (**14**) was used to partially replace the total amount of DSPC used in the conventional formulation (Table 3, entry 1) and molar concentration of 15% (Table 3, entry 9) of Cho-PEG$_2$-α-1,2, Man 1,3-Man (**15**) was used to substitute the amount of cholesterol conventionally used (Table 3, entry 1).

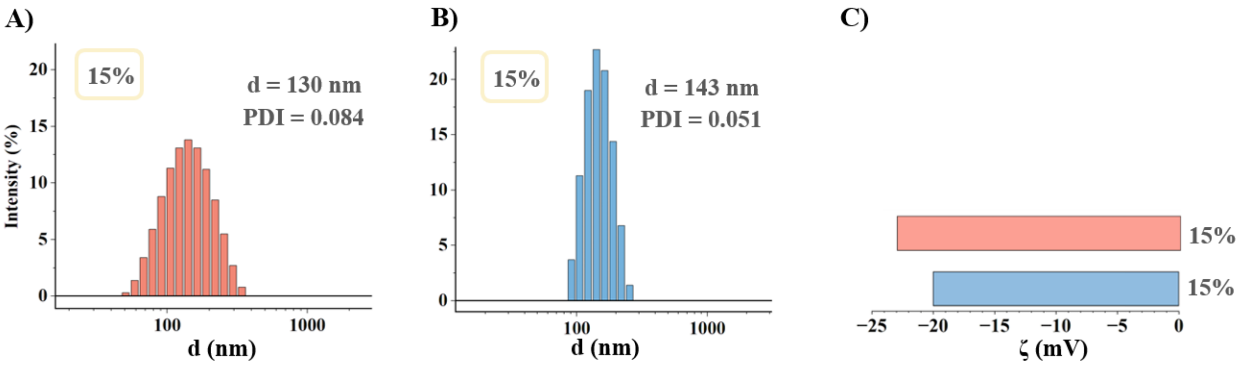

Figure 137. Size and size distribution by intensity of LNPs containing **A)** 15% of Pam_2- PEG_2-α-1,2, Man 1,3-Man (Table 3, entry 8) and **B)** Cho- PEG_2-α-1,2, Man 1,3-Man (Table 3, entry 9) determined by DLS. **C)** Zeta potential of MLNPs (Table 3, entry 8 and 9). (100 µM in H_2O, n = 3). Values are the mean.

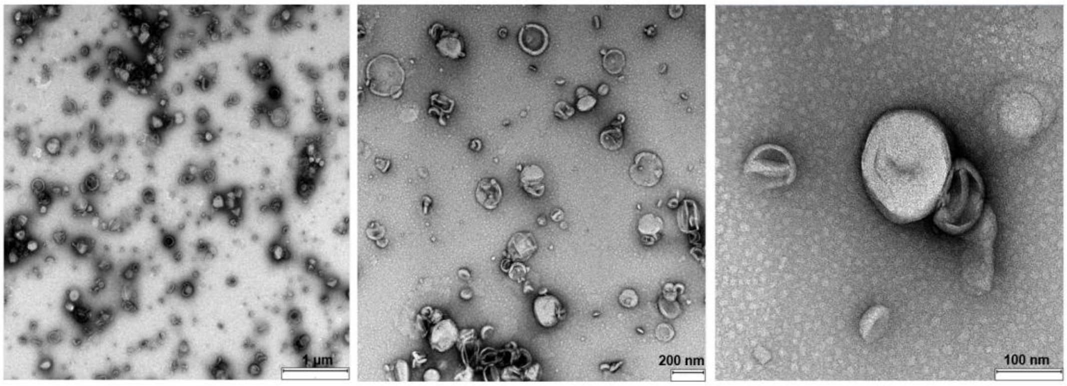

Figure 138. TEM images of LNPs coated with 15% of Pam_2- PEG_2-α-1,2,1,3-Man (Table 3, entry 8).

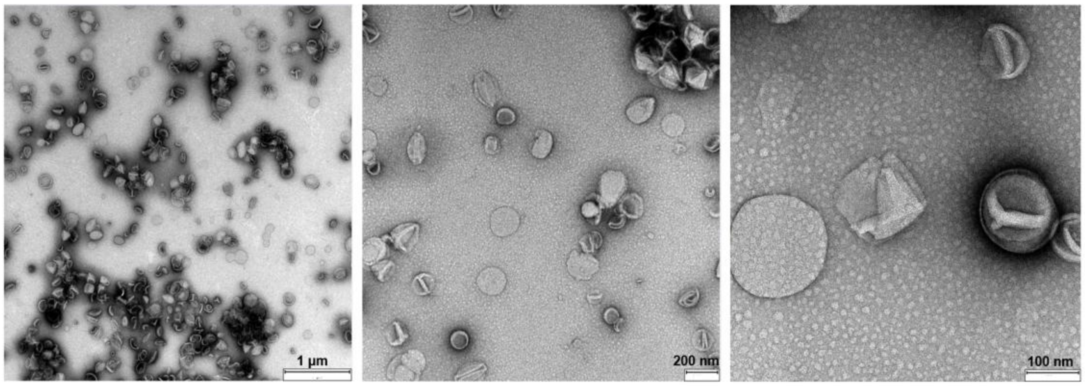

Figure 139. TEM images of LNPs coated with 15% of Cho- PEG_2-α-1,2,1,3-Man (Table 3, entry 9).

8.4. Gb3-LNPs

Molar concentration of 15% (Table 3, entry 10) of Pam_2-PEG_2-Gb3 (**16**) was used to partially replace the total amount of DSPC used in the conventional formulation (Table 3, entry 1) and molar concentration of 15% (Table 3, entry 12) of Cho-PEG_2-Gb3 (**17**) was used to substitute the amount of cholesterol conventionally used (Table 3, entry 1).

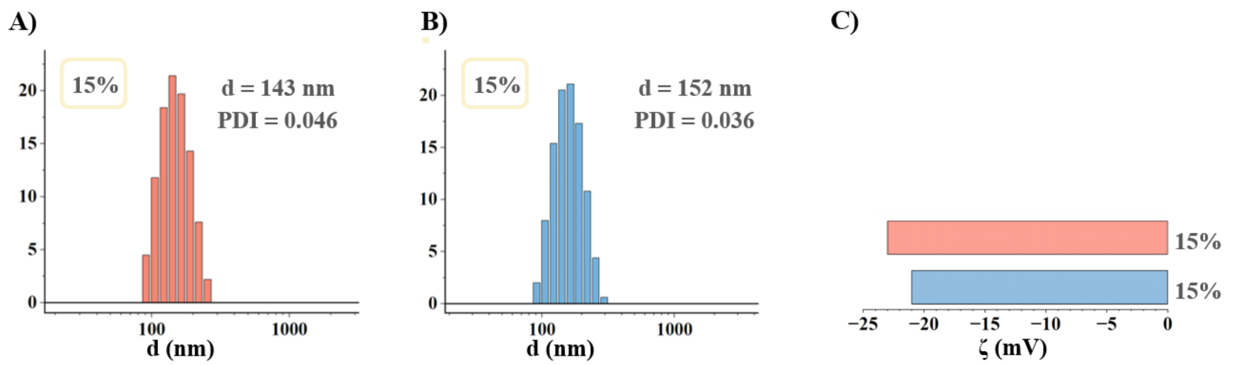

Figure 140. Size and size distribution by intensity of LNPs containing **A)** 15% of Pam$_2$-PEG$_2$-Gb3 (Table 3, entry 10) and **B)** of Cho-PEG$_2$-Gb3 (Table 3, entry 11) determined by DLS. **C)** Zeta potential of Gb3-LNPs (Table 3, entry 10 and 11). (100 µM in H_2O, n = 3). Values are the mean.

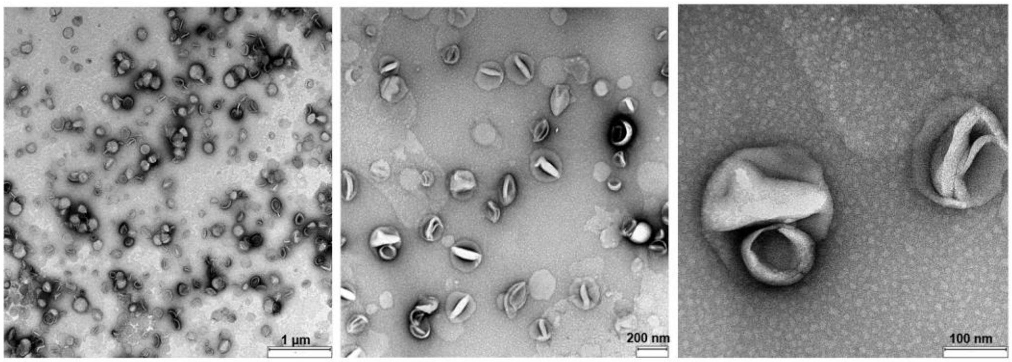

Figure 141. TEM images of LNPs coated with 15% of Pam$_2$- PEG$_2$-Gb3 (Table 3, entry 10).

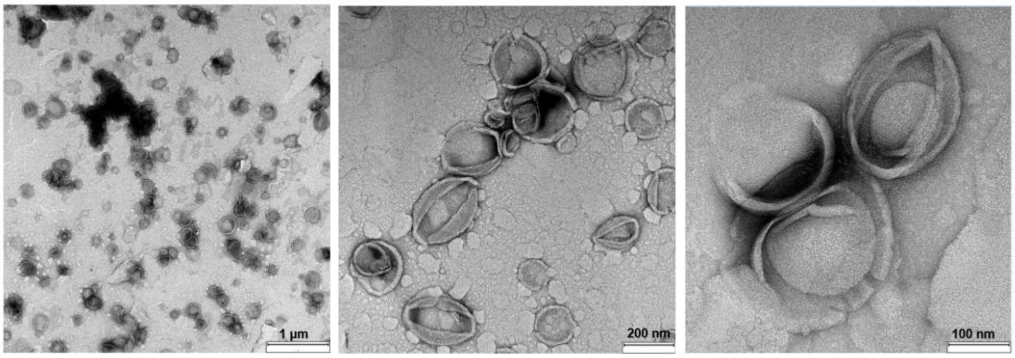

Figure 142. TEM images of LNPs coated with 15% of Cho- PEG$_2$-Gb3 (Table 3, entry 11).

9. Materials and conditions for cell-associated uptake of lipid nanoparticles by antigen presenting cell subsets assays

9.1. Sample handling

Peripheral blood samples were obtained as buffy coats from healthy donors (German Red Cross) and maintained at room temperature (RT) for up to 24 h until further processing. Peripheral whole blood mononuclear cells (PBMCs) were isolated by density gradient centrifugation using Ficoll (Bio & Sell GmbH)-filled Leucosep™ filtered tubes (Greiner Bio-One GmbH) according to manufacturer's protocol. Isolated PBMCs were either used immediately for co-incubation with 6-FAM labeled or unlabeled control liposomes to monitor the kinetics of APC uptake or subjected to monocyte enrichment to evaluate the liposome formulations.

9.2. Monocyte enrichment

Through negative selection, CD14+CD16- monocytes were magnetically enriched from the collected PBMCs using EasySep™ Human Monocyte Enrichment Kit (STEMCELL Technologies) according to manufacturer's protocol. The monocytes were washed and resuspended in pre-warmed RPMI 1640 complete (10% FCS).

9.3. Surface marker staining to evaluate liposome-induced activation of monocytes and associated cell toxicity

Enriched monocytes were seeded in a 96 U-bottom well plate at a concentration of 200,000 cells per well in 200 µL RPMI 1640 complete (10% FCS). Cells were incubated with a dilution series of each liposomal formulation, with a maximum concentration of 26.7 mM for 18 h at 37°C, 5% CO_2. The stimulated cells were washed and stained with Live/Dead ZombieRed and TruStain Fc blocker for 20 min at 4°C, followed by staining with the following fluorochrome conjugated antibodies: CD40 BV421, CD86 PE and HLA-DR APC (all manufactured by BioLegend). Residue antibodies were removed by a series of washing steps and cells were resuspended to a final volume of 100 µL PBS (2% FCS) and acquired on Beckman Coulter CytoFLEX flow cytometer using CytExpert software.

9.4. Monitoring kinetics of liposomal cell-associated uptake by APC subsets

Buffy coat-isolated PBMCs were seeded in a 96 U-bottom well plate at a concentration of $1x10^6$ cells per well in 200 µL RPMI 1640 complete (10% FCS). Cells were incubated with a dilution series of each liposomal formulation, with a maximum concentration of 400 nM for 2 h at 37°C, 5% CO_2. The stimulated cells were washed to remove unbound liposomes and stained with

Live/Dead ZombieUV and TruStain Fc blocker for 20 min at 4°C, followed by staining with the following fluorochrome conjugated antibodies: CD56 BV421, CD3 BV605, CD16 BV650, CD1c BV785, CD19 PerCP Cy5.5, CD123 PE-CF594, CD163 PE-Cy7, CD141 APC, CD88 Alexa Fluor 700 and HLA-DR APC-Fire750 (all manufactured by BioLegend). See figure 73 for representative FACS plots depicting the gating strategy to phenotype APCs. Residue antibodies were removed by a series of washing steps and cells were resuspended to a final volume of 100 µL PBS (2% FCS) and acquired on Beckman Coulter CytoFLEX flow cytometer using CytExpert software.

9.5. Data analysis and statistics

Flow cytometry data was analyzed with FlowJo v10 software, while graphical presentation and statistical analyses were performed using Microsoft Excel and Graph Prism 10. In flow cytometry analysis of monocyte activation and liposome-associated toxicity, frequencies of activated monocytes were background-subtracted using frequencies of non-specific activated monocytes in the unstimulated ddH$_2$O controls. Similarly, 6-FAM fluorescence signal was background-subtracted using autofluorescence from APCs alone.

10. Cell-associated uptake of lipid nanoparticles by antigen presenting cell subsets

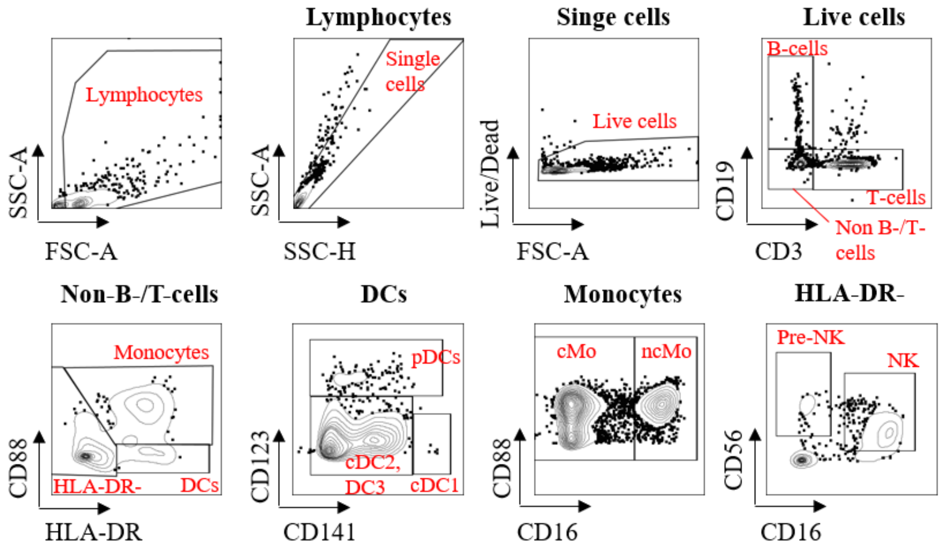

Figure 143. Representative FACS plot depicting the gating strategy to phenotype APCs.

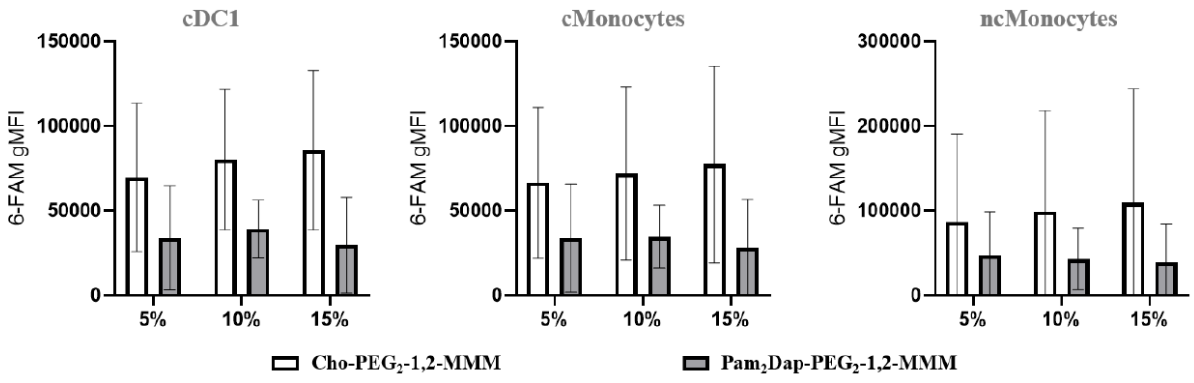

Figure 144. Internalization by APC subsets or surface association increases when increment coating molar concentration from 5 to 15% of α-1,2-trimmanose-containing lipids. Cholestanol-containing glycolipids showed increased uptake values by relevant markers in comparison with the use of pam$_2$dap-containing derivatives.

10.1. Innate cell cytotoxicity

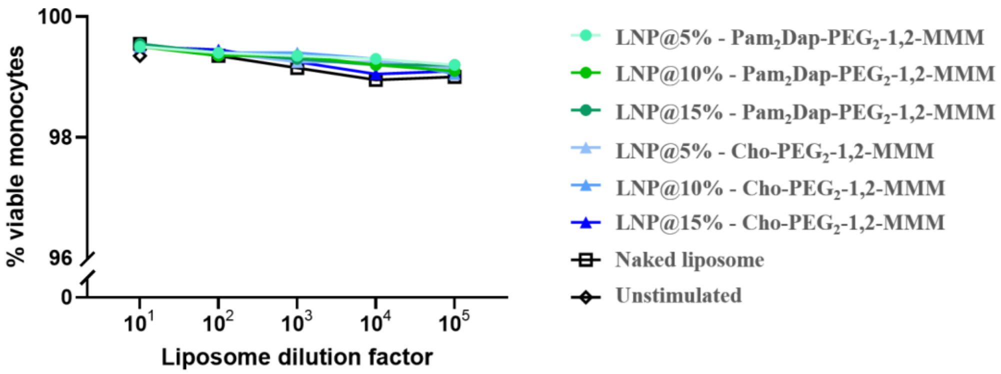

Figure 145. Percentage of viable monocytes after stimulation with LNPs at different concentration for 18 hours.

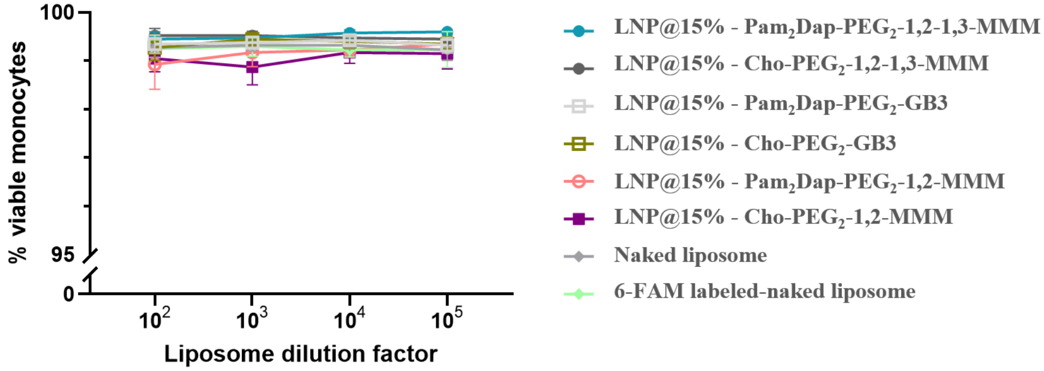

Figure 146. Percentage of viable monocytes after stimulation with LNPs coated with 15% of different glycolipids for 18 hours.

11. References

1. Pardo-Vargas, A., Delbianco, M. & Seeberger, P. H. Automated glycan assembly as an enabling technology. *Curr. Opin. Chem. Biol.* **46**, 48–55 (2018).

2. Singh, R. K., Reuber, E. E., Bruno, M., Netea, M. G. & Seeberger, P. H. Synthesis of oligosaccharides to identify an immunologically active epitope against Candida auris infection. *Chem. Sci.* **14**, 7559–7563 (2023).

3. Kröck, L. *et al.* Streamlined access to conjugation-ready glycans by automated synthesis. *Chem. Sci.* **3**, 1617–1622 (2012).

4. Greis, K. *et al.* The Influence of the Electron Density in Acyl Protecting Groups on the Selectivity of Galactose Formation. *J. Am. Chem. Soc.* **144**, 20258–20266 (2022).

5. M. Byrd, K. *et al.* Design and Synthesis of a Crosslinker for Studying Intracellular Steroid Trafficking Pathways. *Bioorg Med Chem* **23**, 3843–3851 (2015).

6. Gude, M., Ryf, J. & White, P. D. An accurate method for the quantitation of Fmoc-derivatized solid phase supports. *Lett. Pept. Sci.* 203–206 (2002).

7. Zhang, H. *Thin-Film Hydration Followed by Extrusion Method for Liposome Preparation. In: D'Souza, G. (eds) Liposomes. Methods in molecular biology. Humana Press, New York, NY.* vol. 1522 (2017).

8. Hunter, R. J. *Zeta potential in Colloid Science. Principles and Applications*. *ACADEMIC PRESS* (1986). doi:10.4159/harvard.9780674499171.prf.

9. Barre, A., Bourne, Y., Van Damme, E. J. M., Peumans, W. J. & Rougé, P. Mannose-binding plant lectins: Different structural scaffolds for a common sugar-recognition process. *Biochimie* **83**, 645–651 (2001).

10. Goswami, R. *et al.* Mannosylation of LNP Results in Improved Potency for Self-Amplifying RNA (SAM) Vaccines. *ACS Infect. Dis.* **5**, 1546–1558 (2019).
